# 14^th^ PSOGI-ISSPP International congress on Peritoneal Surface Malignancies*: Lyon, 26 – 28^TH^ September 2024

**DOI:** 10.1515/pp-2024-0031

**Published:** 2025-01-16

**Congress president**

Prof. Olivier Glehen

**Scientific committee**

Dr Aditi Bhatt

Dr Naoual Bakrin

Prof. Frédéric Bibeau

Prof. Olivier Glehen

Prof. Diane Goéré

Prof. Santiago González Moreno

Dr Vahan Kepenekian

Prof. Julien Péron

Prof. Marc Pocard

Prof. Beate Rau

Prof. Pascal Rousset

Prof. Victor Verwaal

Dr Laurent Villeneuve

Dr Wiebke Solass

Prof. Yanghee Woo

**Organizing committee**

Prof. Olivier Glehen

Dr Vahan Kepenekian

Prof. Julien Péron

Prof. Pascal Rousset

Prof. Frédéric Bibeau

Prof. Marc Pocard

Dr Naoual Bakrin

Dr Laurent Villeneuve

## Lung cancer with pleural metastasis treated by extrapleural pneumonectomy and hyperthermic intrathoracic chemotherapy (HITOC)

Frédéric Marchal^1^, Stéphane Renaud^2^, Philippe Guerci^3^

^1^Institut de Cancérologie de Lorraine - Vandoeuvre-les-Nancy (France),

^2^Department of Thoracic Surgery Université de Lorraine, INSERM U1256, CHRU NancyCHRU Nancy - Vandoeuvre-lès-Nancy (France),

^3^Department of CardioThoracic and Vascular Anesthesiology & Critical Care Medicine Université de Lorraine INSERM U1116, CHRU Nancy - Vandoeuvre-lès-Nancy (France)

**Background:** Lung cancer with pleural carcinomatosis has a poor prognosis and is often treated for palliative care. We report a case treated curatively with chemotherapy, cytoreductive surgery and hyperthermic intrathoracic chemotherapy (HITOC).

**Patients and methods:** The patient was 55 years old. Two tumors in left lung were diagnosed. Thoracoscopy revealed pleural carcinomatosis, with biopsies concluded to be a well-differentiated papillary adenocarcinoma. Chemotherapy was performed with Carboplatin Alimta Pembrolizumab. A second line of Taxol Avastin was instituted due to progression. The reassessment showed a good therapeutic response.

On 16/04/2024, a left pleuro-pneumonectomy with HITOC was performed. Once the specimen has been removed, 2 inflow drains were inserted the upper part of the thoracic cavity, and 2 outflow drains in the lower part of the thoracic cavity. The skin was closed. Cautious filling of the thoracic cavity with saline solution was started, with poor tolerance at the end of the procedure, with hypotension and bradychardia.

An outlet drain was opened to reduce intrathoracic pressure, and the procedure was carried out under favorable conditions. When the temperature reached 42°C, chemotherapy was inserted in the closed circuit of Performer HT (RanD, Medolla, Italy) with Cisplatin 142 mg during 90 min with renal protections by sodium Thiosulfate.

**Results:** On April 29, a videoassisted minithoracotomy was performed in the 4th space to evacuate blood clots. The patient was discharged for convalescence on D18. Since then, the patient has shown no signs of relapse. Pathological examination of the surgical specimen showed a good therapeutic response, with 4 millimetric foci 0.2 and 0.4 cm long, intraparenchymal and subpleural tumor.

**Conclusion:** Pleuropneumonectomy with HITOC is feasible. When filling the thoracic cavity after pleuropneumonectomy, it is important to evacuate intrathoracic air if a closed-chest procedure is performed, to avoid mediastinal compression (opening an outlet drain or placing a trocar in the scar).

## Prospective study of adjuvant oxaliplatin-based PIPAC with concurrent intravenous 5-fluorouracil and folinic acid after curative surgery for pT4a/b colon cancer

D. Baratti ^1^

^1^Department of Surgical Oncology, Fondazione IRCCS Istituto Nazionale dei Tumori, Milan, Italy.

**Background:** We conducted a prospective single center pilot study to assess feasibility and safety of adjuvant oxaliplatin-based Pressurized Intra-Peritoneal Aerosolized Chemotherapy (PIPAC) after curative surgery for pT4a/b colon cancer. This strategy takes advantage of pathological examination to optimize patient selection, and better drug diffusion and penetration to overcome the (potential) limitations of the postoperative time setting. We also hypothesized that concurrent intravenous 5-fluorouracil and folinic acid (FU/FA) could increase oxaliplatin effect without harm

**Patients and methods:** Ten patients with pT4a/b, N0-2, M0, R0 colon cancer were enrolled. PIPAC was performed within 4-8 weeks from primary surgery with oxaliplatin (92 mg/m^2^) and concurrent intravenous 5-FU/FA (400/20 mg/m^2^). Adjuvant PIPAC was considered feasible if the laparoscopic procedure can be completed in ≥9 patients, and postoperative stay will be ≤3 days in ≥6 patients. Adjuvant PIPAC was considered safe if maximunm one conversion to open surgery, one severe complication (NCI-CTCAE v.4 grade 3-5), and one readmission within 30 days occurred. The trial is registered with Clinicaltrials.gov.NCT06091683.

**Results:** Median age was 59 years (range 41-80). Median interval between primary resection and PIPAC was 6 weeks (range 3-7). The procedure was completed in all patients. Postoperative stay was ≤3 days in all but one patient. One patient had mild (grade 2) transaminase increase. No conversion, severe complication, death, or readmission occurred in the remaining patients. Metachronous peritoneal metastases (undetected at primary surgery) were discovered during PIPAC in one patient. The remaining patients are free of disease after a median of 19 months (range 10-35). Adjuvant systemic chemotherapy was not indicated for two patients (including the one with metachoronous peritoneal metastases), and started within 12 weeks from primary surgery for the remaining patients.

**Conclusion:** Adjuvant oxaliplatin-based PIPAC with concurrent intravenous FU/FA after curative surgery for pT4a/b colon cancer is feasible and safe. Preliminary oncological results are promizing.

## PIPAC in patients with peritoneal metastases from gastrointestinal tract (PIPOX01): an open label, non-comparative phase 1/2 dose escalation and expansion trial.

F. Dumont ^1^, V. Kepenekian ^2^, C. Passot ^3^, A.C. Ezanno-Manasterski ^4^, M. Pocard ^5^, J.L. Raoul ^6^, B. Lelièvre ^7^, J. Paul ^1^, E. Thibaudeau ^1^, G. Olivier ^2^

^1^Institut de Cancérologie de l’Ouest - Nantes (France),

^2^CHU Lyon - Lyon (France),

^3^Institut de cancérologie de l’Ouest - Nantes (France) - Nantes (France),

^4^Hopital d’instruction des armées Bégin - Saint Mande (France),

^5^CHU la pitié salpétrière - Paris (France),

^6^Institut de cancérologie de l’Ouest - Nantes (France) - Nantes (France) - Nantes (France),

^7^CHU Angers - Angers (France)

**Background:** Despite modern systemic chemotherapy, survival remains poor for patients with advanced isolated peritoneal metastases from the gastrointestinal tract. We aimed to assess the safety and efficacy of pressurized intraperitoneal aerosol chemotherapy (PIPAC) with oxaliplatin.

**Patients and methods:** We conducted a phase 1/2, open label, non-comparative, dose escalation and expansion trial of PIPAC with oxaliplatin in patients with a peritoneal cancer index (PCI) of more than 5, 13 and 15 for respectively a gastric, small bowel and colorectal primary cancer, and who had received at least three months of systemic chemotherapy. PIPAC cycle lengths were 4 to 6 weeks with systemic chemotherapy allowed 15 days after each PIPAC. PCI and oxaliplatin tumor concentration were assessed every PIPAC cycle. The main endpoints were tolerability, tumor response, and survival.

**Results:** Between 2017 and 2020, 34 patients were enrolled in three centers, in this phase 1/2 study, of whom 25 were evaluable at the recommended dose determined in the phase I trial (90 mg/m2 plus systemic 5-FU). Before inclusion, patients received a median of 2 [1-4] chemotherapy lines and had a median PCI of 22.5 [7-29]. At this dose, the safety profile showed acceptable tolerability. Eight patients (32%) had grade 3/4 treatment-related adverse events. Minor (grade 1/2) adverse events were mainly abdominal pain (n=19, 76%) and nausea (n=16, 64%). Median PFS was 6.1 months and median OS was 13 months.

**Conclusion:** In patients with advanced and refractory peritoneal metastasis, PFS of 6.1 months is encouraging. A prospective randomized phase II study is required.

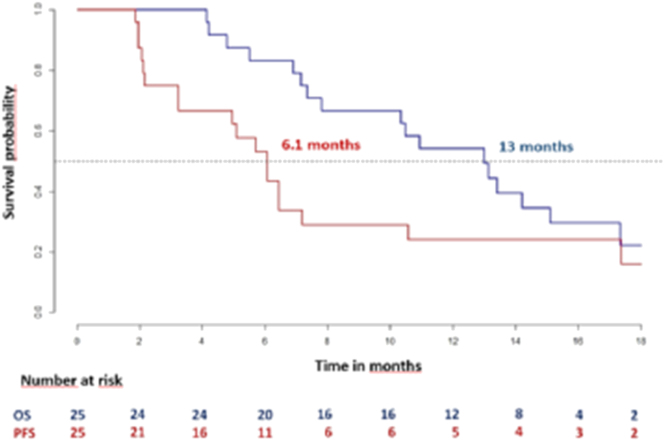



Figure: Overall and progression-free survival of patients

## The role of hyperthermia in intraperitoneal chemotherapy on abdominal tissue concentrations of cisplatin – insights from a porcine model

C. Harlev ^1^, M. Bue ^1^, E. Krogsgaard Petersen ^1^, A. René Jørgensen ^1^, B. Martin Bibby ^1^, A. Vibeke Schmedes ^2^, M. Stilling ^1^, L. Kjeld Petersen ^3^

^1^Aarhus University - Aarhus (Denmark),

^2^Vejle Sygehus - Vejle (Denmark),

^3^Odense University Hospital - Odensen (Denmark)

**Introduction:** In clinical studies cytoreductive surgery (CRS) combined with hyperterm intraperitoneal chemotherapy (HIPEC) utilizing cisplatin has revealed enhanced survival in patients with advanced ovarian cancer.

One potential mechanism behind the effect of HIPEC is increased local penetration of chemotherapy.This study aims to examine the effect of hyperthermia during intraperitoneal chemotherapy on local abdominal tissue concentrations of cisplatin during and after HIPEC and normothermic intraperitoneal chemotherapy (NIPEC).

**Patients and methods:** We used microdialysis to dynamically sample local tissue concentrations during and after HIPEC/NIPEC in a porcine model. All 16 pigs underwent cytoreductive surgery and were divided into two groups, receiving either CRS+HIPEC or CRS+NIPEC. Figure 1 shows the timeline of the experiment and sampling times. Table 1 shows sampling sites. The concentration of cisplatin in dialysate samples was determined by UPLC-MS/MS.

**Table:** 

Organ/tissue	Miicrodialysis catheter placement	Number of microdialysis catheters
Liver	1 mm	2
Rectum	1-1.5 mm (superficial) and 2.5-3 mm (profound)	2
Stomach	1-2 mm (superficial) and 4-5 mm (profound)	2
Peritoneum	1 mm	3

**Results:** There was no statistical significant difference between the maximal concentration or area under the curve between the two groups.

For both the HIPEC and NIPEC setup we found statistical significantly higher concentrations in the peritoneal compartments compared to all other compartments in terms of Cmax and AUC0-last

**Conclusion:** Applying hyperthermia during intraperitoneal chemotherapy did not enhance cisplatin penetration or concentration in abdominal tissue in our study. It remains to be established whether HIPEC confers benefits compared to NIPEC beyond drug penetration by inducing other cytotoxic effects such as promoting apoptosis or impairing DNA repair mechanisms in cancer cells.

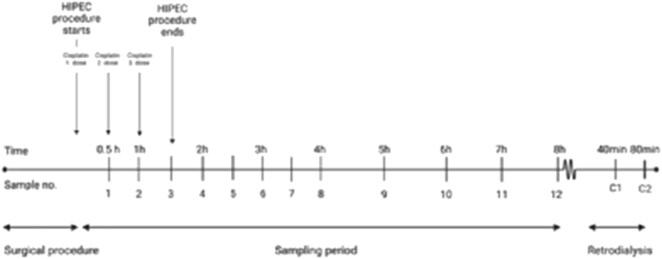



## Immediate second look after cytoreduction of colorectal or ovarian carcinomatosis. Invisible Gorilla effect?

L. Cosse ^1^, F. Dumont ^1^, M.F. Heymann ^1^, O. Kerdraon ^1^, C. Lemarie ^1^, T. Vignaud ^1^, C. Bourgin ^1^, C. Loaec ^1^, E. Thibaudeau ^1^

^1^Institut de Cancérologie de l’Ouest - Nantes (France)

**Background:** Completeness of cytoreductive surgery (CRS) is an important component for cure of peritoneal carcinomatosis (PC) from colorectal (CRC) or ovarian cancer. The aim of this study was to assess an immediate second look to check no residual nodules.

**Patients and methods:** Immediately at the end of CC0 procedure performed by an experienced surgeon, another experienced surgeon, blind for extend and type of disease, checked completeness of cytoreduction of first operator. Data were prospectively recorded.

**Results:** Between November 2021 to February 2024, 99 patients underwent CC0 procedures for PC from CRC or ovarian cancer with 41 patients with second look (feasibility rate 44.4.%) in one comprehensive cancer center. The origin of PC were CRC (n=24) or ovarian (n=17). During second look, 92 additional nodules were removed, of which 33 (33.3%) malignant. Additional malignant nodules of PC were found in 20 (50%) patients. Among the 4 experienced surgeons, no one had systematically performed real CC0 procedure. In univariate analysis, the risk factors to find additional malignant nodules were high median PCI (11.5 vs 8, p=0.03). Nor the operator, the prior surgical score, the preoperative chemotherapy, the mucinous histologic type or duration of the procedure had significant impact on the rate of new tumor nodules detected.

**Conclusion:** Rate of residual tumor nodules is dramatically high (50%) despite CRS considered complete by expert surgeon. Multiple and wellknowns limits of human attention are the first hypothesis of this result. Immediate second look or assistance for nodule detection are required to check completeness of CRS.

Number of patients with additional malignant nodul

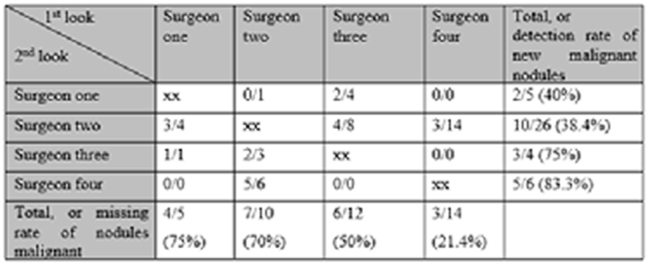



## Compliance and toxicity of intraperitoneal chemotherapy in metastatic colorectal and appendiceal cancer: Results of the randomized multicenter Intraperitoneal Chemotherapy After cytoReductive Surgery (ICARuS) trial

G. Karagkounis ^1^, N. Aguirre ^1^, K. Hester ^1^, M. Mohamed ^1^, E. Brady ^1^, M. Foote ^1^, A. Cercek ^1^, G. Nash ^1^

^1^Memorial Sloan Kettering Cancer Center - New York (United States)

**Background:** Intraperitoneal chemotherapy (IPC) is administered to reduce recurrence after Cytoreductive surgery (CRS) in select patients with colorectal (CRC) and appendiceal cancer (AC) presenting with peritoneal carcinomatosis (PC). However, direct comparisons between different IPC modalities are limited and their toxicity and compliance profiles remain incompletely defined.

**Patients and methods:** The Intraperitoneal Chemotherapy After cytoReductive Surgery (ICARuS) trial randomized patients with PC from CRC and AC who underwent CRS to either Hyperthermic Intraperitoneal Chemotherapy (HIPEC) with mitomycin C or Early Postoperative Intraperitoneal Chemotherapy (EPIC) with floxuridine. Patients were stratified by recent systemic chemotherapy and primary (AC vs. CRC). 30-day adverse events (AE) were graded by CTCAE V4.0.

**Results:** 285 patients were enrolled between April 2013 and May 2023 (75 CRC and 210 AC), with 145 randomized to HIPEC and 140 to EPIC. The average age was 56 years, and 59% were female. All HIPEC patients completed IPC treatment per protocol, whereas one patient declined planned EPIC after complete CRS. Adverse events per arm are detailed in the Table. There was no difference in length of stay (median HIPEC: 8d, EPIC: 9d, *p*=0.41) or ICU/stepdown admission (HIPEC: 12%, EPIC: 13%, *p*=0.95). Readmission was more frequent after EPIC than HIPEC (19% vs.10%, *p*=0.048). Reoperation was necessary in 8 patients (HIPEC: 2.1%, EPIC: 3.4%, *p*=0.50).

**Table:** 

Clinically significant adverse events in the two study arms by CTCAE v4.0				
	All Patients (n=285)	HIPEC (n=145)	EPIC (n=140)	p-value
**Any**	133 (47%)	55 (38%)	78 (56%)	**0.003**
**Gastrointestinal**	61 (21%)	25 (17%)	36 (26%)	0.08
**Cardiopulmonary**	36 (13%)	13 (9%)	23 (16%)	0.06
**Other**	87 (31%)	34 (23%)	53 (38%)	**0.008**
**Grade 3+**	97 (34%)	44 (30%)	53 (38%)	0.18
**Gastrointestinal Grade 3+**	46 (16%)	20 (14%)	26 (19%)	0.27
**Cardiopulmonary Grade 3+**	17 (6%)	8 (6%)	9 (6%)	0.74
**Other Grade 3+**	64 (23%)	27 (19%)	37 (26%)	0.11
**Mortality**	1 (0.4%)	0 (0%)	1 (0.7%)	0.49

**Conclusion:** Compliance with planned IPC after CRS was high regardless of approach. There was no difference between arms in the primary toxicity outcome of Grade 3+ AE, though overall AE and readmissions were more common in the EPIC arm.

## Deep epigastric lymph node involvement in patients with ovarian and colorectal peritoneal metastasis

A. El Asmar ^1^, C. Khaled ^1^, I. Veys ^1^, A. Hendlisz ^1^, M. Gomez Galdon ^1^, L. Verset ^1^, L. Polastro ^1^, A. Veron Sanchez ^1^, V. Donckier ^1^, G. Liberale ^1^

^1^HUB - Institut Jules Bordet, Université Libre de Bruxelles - Brussels (Belgium)

**Background:** The systemic dissemination of peritoneal metastases of ovarian and colorectal cancer (PMOC and PMCRC) from the intra to the extra-abdominal compartment has been previously described through the retroperitoneal lymph nodes (LN), and the diaphragmatic stomata. However, inferior deep epigastric LN (IDELN) basin has been recently described as an alternative lymphatic pathway for cancer dissemination. The objective of this study was to determine the incidence and conditions of involvement of these IDELN in the systemic spread, in patients with PMOC and PMCRC.

**Patients and methods:** This is a prospective monocentric nonrandomized controlled clinical trial conducted over a period of 3 years (December 10th 2020 – September 1st 2023). It includes patients with PMOC (NCT06237582) and PMCRC (NCT04966715), presenting for curative-intent cytoreductive surgery (CRS). For each patient, bilateral IDELN harvesting and histological analysis was performed.

**Results:** 40 patients were enrolled in the study, 20 patients from each group. The median peritoneal cancer index (PCI) for PMOC and PMCRC were 12 and 13 respectively. CC-0 was achieved in 16 patients in both groups. Out of the 20 patients with PMOC, 6 (30%) had positive IDELN, including 5 with high PCI (31, 28, 25, 17, 16). 4 had incomplete CRS (CC-1), and all exhibited high grade carcinoma, extensive pelvic involvement, and synchronous PM. In patient with PMCRC, 2 had positive IDELN (10%). These 2 patients had PCI of 25 and 15, both had unresectable disease, primary left colonic tumor with synchronous PM, and extensive pelvic peritoneum involvement.

**Conclusion:** The IDELN basin represents a drainage route for the peritoneum, and thus an alternative pathway for systemic dissemination in 30% and 10% of patients with PMOC and PMCRC, respectively. IDELNs were positive in patients with high grade disease, high PCI and extensive pelvic peritoneal disease.

## Impact of laparoscopic ultrasound during PIPAC directed treatment of unresectable peritoneal metastasis

M.S. Jørgensen ^1^

^1^Odense PIPAC Center, department of surgery, OUH, Denmark - Odense M (Denmark)

**Objectives:** Laparoscopic ultrasound (LUS) combines laparoscopy and ultrasound and it images peritoneum, liver and retroperitoneum. LUS has not been described in treatments with Pressurized Intraperitoneal Aerosol Chemotherapy (PIPAC). We present our experience with LUS in patients undergoing PIPAC directed therapy.

**Patients and methods:** Retrospective study of LUS findings from the prospective PIPAC-OPC2 trial. Outcome was changes in overall treatment strategy due to LUS findings.

PIPAC-OPC2 included 143 patients including 33 patients treated with electrostatic precipitation PIPAC. Nine patients were excluded due to primary non-access.

**Results:** During PIPAC 1, LUS was performed in 112 of 134 (84%) PIPAC procedures and changed overall treatment strategy in 1 patient due to detection of multiple liver metastases unseen by baseline CT. During PIPAC 2 and 3 LUS was performed in 59 of 104 (57%) and 42 of 78 (54%) PIPAC procedures, respectively. LUS changed overall treatment strategy in 1 patient during PIPAC 3 again due to detection of multiple liver metastases. Throughout PIPAC 1-3, LUS also detected pathological lymph nodes in 16 patients and focal liver lesions in another six patients of uncertain origin. No further examinations were performed in these patients and the overall treatment strategy was not changed according to the PIPAC-OPC2 protocol. One patient had a splenic capsule rupture related to the LUS itself. This was managed conservatively.

**Conclusion:** LUS may be safely performed during PIPAC directed therapy. However, LUS has limited clinical impact in patients scheduled for PIPAC and cannot be recommended as a routine procedure as part of performing PIPAC.

## Developing an AI tool for accurate radiological assessment of peritoneal metastases

L.J.S. Ewals ^1^, M. Welten ^1^, Y. Tong ^2^, C.H.B. Claessens ^2^, J.M.J. Piek ^1^, F. Van Der Sommen ^2^, M.J. Lahaye ^3^, I.H.J. De Hingh ^1^, M.D.P. Luyer ^1^, J. Nederend ^1^

^1^Catharina hospital - Eindhoven (Netherlands),

^2^Eindhoven University of Technology - Eindhoven (Netherlands),

^3^Netherlands Cancer Institute - Amsterdam (Netherlands)

**Background:** With emerging treatment options for peritoneal metastases (PM), accurate noninvasive methods are needed to assess the presence and extensiveness of PM. Our goal is to develop an Artificial Intelligence (AI) tool to assist radiologists in PM assessment on CT.

**Methods:** First, an AI model will be developed that divides the abdomen into 13 regions based on the Peritoneal Cancer Index (PCI). To achieve consensus on PCI region definitions on CT, a Delphi study was distributed among international experts (radiologists, surgeons, gynecologists) in the field of PM. By pragmatically approaching the surgical PCI region definitions, a first 3D prototype was designed (Figure). Experts evaluated all region’s structures/boundaries on a 5-point Likert scale using an online questionnaire. Consensus was defined as >75% agreement and <15% disagreement. As a proof-of-concept, regions 1-3 were segmented in 60 CT scans (50 training, 10 validation), whereafter a pre-trained AI model for abdominal organ segmentation was finetuned for segmenting these regions.

**Results:** In total, 45 of 88 experts participated and consensus was achieved for regions 0-2, 4, 6, and 8. Preliminary results on AI for segmenting regions 1-3 yielded a mean Dice score of 0.92, with minimal deviations from the ground truth (Figure).

**Conclusion:** Delphi round one achieved consensus on most regions for radiological PCI scoring. Further rounds aim to achieve consensus on the remaining structures/boundaries. The initial results of the developed AI model demonstrate that PCI region segmentation on CT is feasible; therefore, the next step will be training of AI models for all regions.

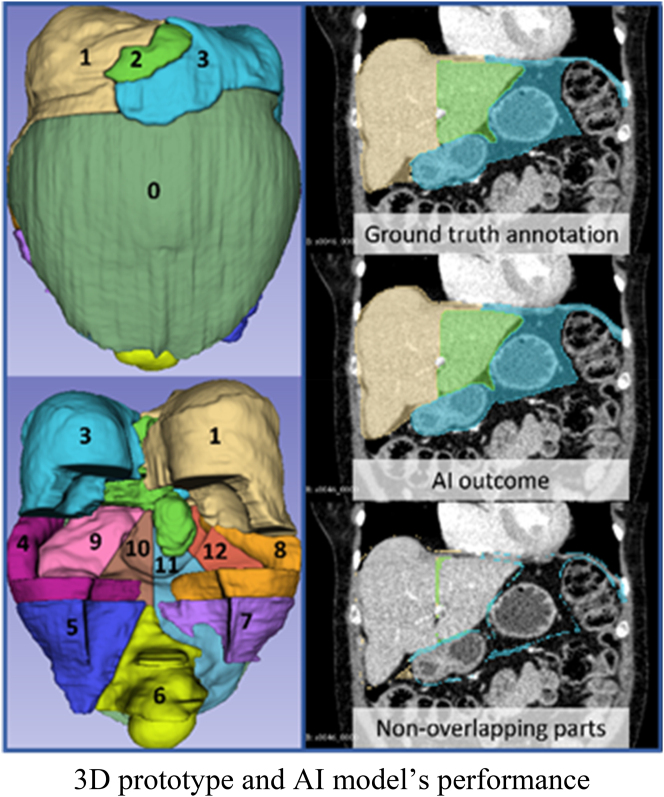



## Insights into the Clinical Prognosis of High-Grade Appendiceal Mucinous Neoplasms: Lymph Node Metastasis and Peritoneal Risks of HAMN

P. Dartigues ^1^, V. Kepenekian ^2^, C. Illac-Vauquelin ^3^, V. Verriele ^4^, J. Fontaine ^2^, I. Sylvie ^2^, A. Chevalier ^5^, S. Valmary-Degano ^6^, O. Glehen ^2^, N. Benzerdjeb ^2^

^1^Gustave Roussy Institute - Villejuif (France),

^2^CHU LYON SUD - Lyon (France),

^3^Claudius Regaud Institute - Toulouse (France),

^4^CRLCC Paul Papin - Angers (France),

^5^CHU l’Archet II - Nice (France),

^6^CHU Grenoble - Grenoble (France)

**Background:** High-grade appendiceal mucinous neoplasm (HAMN) is a relatively recent term used to describe a rare epithelial neoplasm of the appendix characterized by pushing-type invasion and high-grade cytologic atypia. Its implications regarding lymph node spread and the necessity of right colectomy are subjects of ongoing debate. All cases undergoing right colectomy for pseudomyxoma were included from the French reference network for rare peritoneal cancers (RENAPE) database to explore lymph node and peritoneal spread.

**Patients and methods:** We analyzed data from 440 patients diagnosed with low or high appendicular mucinous neoplasm (LAMN, n=240 / HAMN, n=33) or appendicular adenocarcinoma (AA, n=167) who underwent right colectomy with lymph node dissection within the RENAPE network.

**Results:** Nearly 60% of the patients were female (n=249), with a mean age of 56.6 years (range: 21-83). No difference of number of appendiceal wall rupture among the three subsets. Lymph node metastases were identified only in 16.2% of AA (27/167), whereas none were found among LAMN/HAMN cases. In terms of peritoneal metastasis, a significantly higher proportion of cases were classified as high grade with/without signet cells in HAMN (69.2%) compared to LAMN (15.6%) and AA (44.0%). In terms of peritoneal metastasis, a significantly higher proportion of cases were classified signicantly as high grade in HAMN and AA compared to LAMN. HAMN and AA with appendicular perforation experienced significantly more peritoneal recurrences than LAMN (p<0.001). In cases confined to the appendiceal wall, HAMN and LAMN confined to the appendiceal wall had a lower tendency for peritoneal recurrences than AA (p = 0.132).

**Conclusion:** Our data suggest that HAMN is more prone to peritoneal dissemination as high-grade expansion rather than nodal spread.

## Incidence of Pseudoprogression and Hyperprogression in Patients with Peritoneal Mesothelioma Treated with Ipilimumab and Nivolumab

O. Mitchell ^1^, F. Li ^2^, G. Porroga ^1^, M. Möller ^3^, A. Husain ^4^, M. Drazer ^1^, S. Armato ^2^, H. Kindler ^1^

^1^Section of Hematology/Oncology, Department of Medicine, University of Chicago - Chicago, IL (United States),

^2^Department of Radiology, University of Chicago - Chicago, IL (United States),

^3^Department of Surgery, University of Chicago - Chicago, IL (United States),

^4^Department of Pathology, University of Chicago - Chicago, IL (United States)

**Background:** The immune checkpoint inhibitors (ICI) ipilimumab and nivolumab (I/N) are standard treatment for pleural mesothelioma, but there is little data in peritoneal mesothelioma (PeM). In other cancers, ICI can produce an initial increase in tumor burden followed by regression (pseudoprogression) or accelerated (>50%) increase in tumor growth (hyperprogression). These phenomena have not been previously quantified in PeM. We therefore evaluated pseudoprogression and hyperprogression in PeM patients receiving I/N.

**Patients and methods:** Patients in a high-volume Mesothelioma Clinic were enrolled in an IRB-approved biorepository protocol. CT scans, obtained at baseline, 6 and 12 weeks after starting I/N, then every 3 months thereafter, were measured by a reference radiologist using RECIST 1.1. Statistical significance was determined via Fishers exact and Wilcoxon ranked sum tests.

**Results:** 32 PeM patients who received I/N between 5/2017 and 2/2024 were included. Characteristics: male 56%; median age 59 (26-77); epithelioid/biphasic/sarcomatoid: 81%/16%/3%; measurable disease 59%; PD-L1 >1%: 53%; probable/definite asbestos exposure 66%; germline mutation 27%. Of 10 patients (31%) with progression at initial reassessment, 8 had regression on subsequent imaging. Hyperprogression occurred in 11% of patients with measurable disease. No statistically significant differences in age, sex, histology, PD-L1 expression, asbestos exposure, somatic or germline mutations were observed between patients with and without pseudoprogression or hyperprogression.

**Conclusion:** In PeM patients treated with I/N, 80% of those with worsening disease at first reassessment had subsequent tumor regression, for an overall pseudoprogression rate of 25%. This relatively high incidence of pseudoprogression suggests that in the absence of significant clinical deterioration, PeM patients on I/N with possible progression at initial imaging should consider continuing treatment until subsequent imaging is performed. No statistically significant differences in clinical characteristics predicted either phenomenon. Ongoing analyses correlate incidence of pseudoprogression with objective response rate, response duration, and overall survival, and assess impact of hyperprogression on overall survival.

## Targeted Gene Therapy for Peritoneal Dissemination via MicroRNA Modulation: A Novel Approachch

Y. Kaeko ^1^, H. Ohzawa ^1^, R. Watano ^1^, H. Mizukami ^1^, N. Sata ^1^, H. Yamaguchi ^1^, J. Kitayama ^1^

^1^Jichi Medical University - Tochigi (Japan)

**Background:** In previous studies, we found that microRNA (miR)-29b levels in exosomes of peritoneal fluids were markedly reduced in patients with peritoneal metastasis (PM) of gastric cancer.

**Methods:** We engineered an adeno-associated virus encoding miR-29b (AAV-miR-29b) and assessed its impact on peritoneal mesothelial cells (PMC) in vitro. Utilizing a syngeneic model employing gastric (YTN16P) or pancreatic (PAN02) cancer cells, we administered a single intraperitoneal (IP) injection of 5 × 10^10 vector genomes of AAV-miR-29b and evaluated PM progression after 3-weeks.

**Results:** AAV-miR-29b was efficiently assimilated into PMC, leading to a significant upregulation of miR-29b expression within their exosomes. Stimulation with TGF-β1 (10 ng/ml) elicited mesothelail mesenchymal transition, augmenting their migratory capacity and tumor cell adhesion. However, these effects were entirely nullified by AAV-miR-29b. IP-injection of AAV-miR-29b resulted in a substantial increase in miR-29b levels within peritoneal tissue after 2 weeks. Notably, a single IP injection of AAV-miR-29b on day 0 or 3 significantly decreased the number of PM nodules in the mesentery, accompanied by marked inhibition of peritoneal fibrosis (P<0.05). While the inhibition was not statistically significant upon administration on day 7 alone, combination transfer with low-dose paclitaxel (PTX) (200 µg) significantly reduced the number of PM nodules, a response not observed with PTX alone.

**Conclusion:** A single IP administration of AAV-miR-29b effectively suppressed PM progression. Given the potent capability of miRNAs to modulate the tumor microenvironment through targeting multiple molecules, transfer of miRNAs to the peritoneum emerges as a novel gene therapy for PM prevention and treatment.

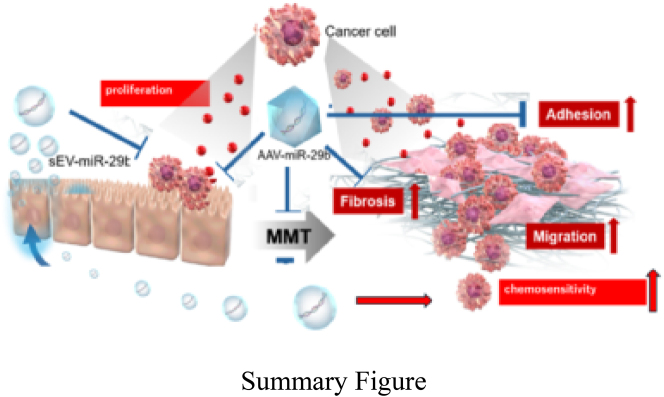



## Immune Profiles in Colorectal Cancer Peritoneal Metastases: Who could benefit from immunotherapy?

J. Sand ^1^, D. Ljungman ^1^, V. Verwaal ^2^, M. Quiding Järbrink ^3^, Y. Wettergren ^1^, E. Bexe Lindskog ^1^

^1^Department of Surgery, Institute of Clinical Sciences, Sahlgrenska Academy, University of Gothenburg. - Gothenburg (Sweden),

^2^Department of Clinical Sciences, Lund University, Skåne University Hospital. - Malmö (Sweden),

^3^Department of microbiology and immunology, Institute of biomedicin, Sahlgrenska Academy, University of Gothenburg - Gothenburg (Sweden)

**Background:** Patients with peritoneal metastases (PM) from colorectal cancer have a poor prognosis and additional treatment options need to be evaluated. For some patients with PM, immunotherapy might be a future treatment option. Previous research focused on surgical outcomes or aggregated data for all stage IV patients in chemotherapy studies. There is a gap in the understanding of the microenvironment of PM.

**Patients and methods:** Using an expression panel of immune related genes, we analyzed RNA from 61 patients who underwent cytoreductive surgery with hyperthermic intraperitoneal chemotherapy during 2014-2022 at Sahlgrenska University Hospital. Using nSolver, we calculated the differential expression of specific genes correlated to clinical data, adjusting for False Discovery Rate. Moreover, we performed cluster analysis, using a bagging simulation of 10’000 cycles.

**Results:** Median time to relapse was 12 months and 70% of relapses were distant metastases, not limited to the peritoneum. After 5 years 12% of patients were considered cured. We found no differences in gene expression related to age, gender, peritoneal cancer index, BMI, 12 months relapse-free survival or metachronous PM. The cluster analysis identified a subgroup comprising 13% of patients (cluster A), which showed increased presence of cytotoxic T-cells and decreased presence of macrophages. In addition, this subgroup showed higher expression of known T-cell exhaustion markers (TIM-3, TIGIT) and lower expression of their ligands (PD-L2, HVEM), and some proinflammatory markers (NF-κB, IKKβ, CD27). Cluster A did not have a statistically significantly longer time to relapse or cancer specific survival.

**Conclusion:** Research into distinguishing features of PM may provide novel insights. Our findings suggest that a subgroup of patients with PM have an increased number of cytotoxic T-cells in the tumor microenvironment, which may be exhausted or suppressed by microenvironment factors. Patients in this subgroup could be candidates for immunotherapy. Further investigation is required to confirm these results.

## Development and Implementation of a Novel Language Processing Solution to Measure Health-Related Quality of Life following Major Abdominal Surgery in Peritoneal Surface Malignancy

X.Z. Low ^1^, N.B. Shannon ^2^, T. Javed ^3^, N. Liu ^4^, M. Gandhi ^1^, M. Cai ^3^, C.J. Seo ^3^, C.A.J. Ong ^3^, C.S. Chia ^3^, J.S.M. Wong ^3^

^1^Duke-NUS Medical School - Singapore (Singapore),

^2^Department of Head and Neck Surgery, Division of Surgery and Surgical Oncology, National Cancer Centre Singapore and Singapore General Hospital, Singapore - Singapore (Singapore),

^3^Department of Sarcoma, Peritoneal and Rare Tumours (SPRinT), Division of Surgery and Surgical Oncology, National Cancer Centre Singapore and Singapore General Hospital, Singapore - Singapore (Singapore),

^4^Duke-NUS Medical School - Singapore (Singapore) - Singapore (Singapore)

**Background:** Patient reported outcome measures (PROMs) including health-related quality of life (HRQoL) provide rich information on patients’ perceptions of their well-being after surgery. However, widespread implementation of HRQoL questionnaires is challenging due to labour-intensive data collection. Natural language processing (NLP) has emerged as a promising solution that can rapidly organize and analyse large volumes of unstructured free clinical text from electronic medical records (EMR). We aim to develop an EMR-based NLP solution that can identify and measure dimensions of HRQoL in EuroQol EQ-5D-5L in peritoneal surface malignancy (PSM) patients undergoing major elective abdominal surgery.

**Methods:** We queried the SingHealth Integrated Health Information Systems (IHiS) Electronic Health Intelligence System (eHIntS) and extracted unstructured free clinical text of PSM patients during their surgical admission. Pre-processing and feature extraction were performed based on established NLP processes to transform unstructured text into a structured set of features suitable for development of a machine learning (ML) classification model. Cross-validation for hyperparameter optimisation of the model was performed within training data prior to assessment of model performance on set-aside test data. The model classified patient EQ-5D-5L dimension scores based on free clinical text and model performance was assessed against gold-standard scores of patient-reported EQ-5D-5L questionnaires.

**Results:** In our preliminary analysis, we analysed 4500 unstructured free clinical text documents of n=32 PSM patients between January 2020 and September 2023. After feature extraction, 123 structured n-gram features were used to train a Support Vector Classifier model to classify patient EQ-5D-3L ‘mobility’ dimension score. The model achieved sensitivity of 50% and specificity of 100% in the set-aside test data.

**Conclusion:** We have demonstrated feasibility of an NLP solution to evaluate dimensions of HRQoL from existing clinical free text documentation among PSM patients. Further refinement and validation of our NLP-HRQoL tool can lead to wider application among other surgical patients.

## Comparison of 30-day major complications defined by NCI-CTCAE versus Clavien-Dindo classifications in patients with colorectal peritoneal metastases undergoing cytoreduction

W. El Hout ^1^, L. Larby ^1^, S.T.O.D. Sarah T. ^1^, M.S.W. Malcolm ^1^, C.R.S. Chelliah ^1^, P.S. Paul ^1^, J.W. Jonathan ^1^, R.F. Rebecca ^1^, O.A. Omer ^1^, A.G.R. Andrew ^1^

^1^The Christie NHS Foundation Trust - Manchester (United Kingdom)

**Background:** The National Cancer Institute Common Terminology Criteria for Adverse Effects (NCI-CTCAE) and Clavien-Dindo classifications both categorise surgical-related complications into six classes from 0 (no complications), 1 and 2 (minor complications), 3 and 4 (major complications) and 5 (death). Both are commonly reported in the literature, but there are few direct comparisons between the two systems. In particular, the NCI-CTCAE system has specific criteria to capture medical complications, in addition to surgical complications. This study aims to compare major complication rates as classified by NCI-CTCAE and Clavien-Dindo classifications among colorectal cancer patients undergoing cytoreductive surgery (CRS) and HIPEC.

**Patients and methods:** We identified 500 patients with colorectal peritoneal metastases undergoing CRS-HIPEC in a UK national peritoneal oncology centre between January 2013 and December 2023 inclusive. We described 30-day major complication rates by both classifications and related these to length of CCU and hospital stays. For the NCI-CTCAE classification, we further classified major complications as surgical and medical.

**Results:** Median age at surgery was 62 years (range 21-82), with a mean Peritoneal Cancer Index of 7. Using NCI-CTCAE, the 30-day major complication rate was 38% (n=191), whereas using Clavien-Dindo, it was 11% (n=57). Notably, 70% of patients with a medical major complication per NCI-CTCAE were not classified as such by Clavien-Dindo. Median CCU stays were 2 days for both classifications, while median hospital stays were 13 days and 15 days, respectively. There was one 30-day mortality.

**Conclusion:** The 30-day major complications defined by NCI-CTCAE versus Clavien-Dindo classifications in patients with colorectal peritoneal metastases undergoing cytoreduction yield very different rates. Appreciation of these differences are required where surgeons are informing their patients regarding the rates and range of major complications that might occur in the early post-operative period and where researchers are meta-analysing complication rates across studies.

## Correlation of KELIM score with Pathological PCI and chemo response grade and its validation as a prognostic predictor of survival after interval cytoreduction in advanced high grade serous ovarian cancer.

S. Sinukumar ^1^, D. Damodaran ^2^, D. S ^2^, S. Piplani ^1^

^1^Jehangir Hospital - Pune (India),

^2^MVR Hospital - Calicut (India)

**Background:** The aim of the study was to correlate the KELIM Score with the pathological PCI and the CRG, and validate which of the three is a better prognostic marker in stage IIIC high grade serous epithelial ovarian cancer patients.

**Patients and methods:** In this retrospective multicentric study, all patients of Stage IIIC high grade serous carcinoma of ovary were included. Receiver operating curves (ROC) were applied to compare the prognostic value of pathological PCI (pPCI) with the KELIM score and the CRG in predicting OS and DFS. The correlation between KELIM, pPCI and the CRG was tested using the Pearsons correlation. Survival curves were calculated using the Kaplan-Meier test.

**Results:** From Jan 2018 to June 2023,171 patients undergoing interval CRS with or without HIPEC were included. There was a statistically significant correlation between the KELIM score, pathological PCI (r= -3.92, p- <0.001) and CRG (r=0.27, p=<0.001). ROC Curves, determined a pathological PCI value of 8 was taken as the cut-off value. On evaluating the factors affecting OS and DFS, KELIM score greater than 1 was associated with improved OS (p=0.001) and DFS (p=0.001) on both univariate and multivariate analysis, while pathological PCI with a cut off value of 8 showed improved DFS, 8, (p=0.001) in both univariate and multivariate analysis and an improved OS in univariate analysis alone (p=0.03).

**Conclusion:** The KELIM score correlates well with the pathological PCI. After interval CRS, Pathological PCI of 8 and above and KELIM score less than 1 are poor prognostic indicators of Overall survival and disease free survival.

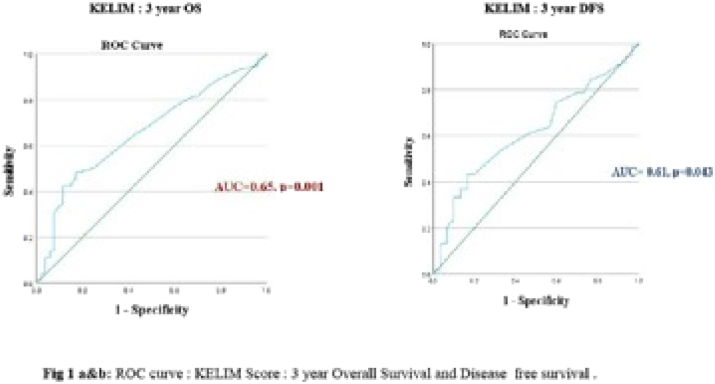



## Comparing doublet vs conversion neoadjuvant therapy prior to cytoreductive surgery and hyperthermic intraperitoneal chemotherapy in colorectal peritoneal metastatic disease

P. Cashin ^1^, P. Grönlund ^1^, H. Birgisson ^1^, W. Graf ^1^, L. Ghanipour ^1^

^1^Uppsala University Hospital - Uppsala (Sweden)

**Background:** Neoadjuvant chemotherapy (NAC) before cytoreductive surgery (CRS) plus hyperthermic intraperitoneal chemotherapy (HIPEC) is used selectively in Sweden for peritoneal metastases (PM) from colorectal cancer (CRC). This study aims to evaluate objective response rates, rates of conversion to CRS and HIPEC, and prognosis after NAC.

**Patients and methods:** Preoperative cases evaluated at the HIPEC multi-disciplinary therapy conference (MDT) at Uppsala University Hospital between June 2019 until December 2022 were screened for inclusion. The cohort was based on three groups, those enrolled for upfront surgery, those treated with double regimen NAC and those treated with NAC with conversion therapy (doublet plus targeted or triplet regimen). Objective response rate (ORR) and overall survival (OS) was compared. A subgroup analysis including patients with specific conversion request from the MDT was performed.

**Results:** 115 patients were included - 53 patients in the upfront, 35 in the doublet and 27 in the conversion group. The ORR in PM was 37.2% in the doublet group versus 37% in the conversion group (p>0.05). For those with conversion request from MDT the ORR in PM was 33.3% in the doublet subgroup (n=18) versus 34.7% in the conversion subgroup (n=23), p>0.05. 40% in the doublet group and 48% in the conversion group went through a complete CRS after being treated with NAC, p=0.61. For the conversion requested subgroup, it was 22% vs 39%, p=0.18. Median OS was 33 months in the upfront group, 16 months in the doublet group, and 20 months in the conversion group. Median OS after a complete CRS was 34, 35.5, and 40 months, respectively.

**Conclusion:** The ORR after 4 cycles of NAC was not improved by intensified conversion therapy. Conversion to complete CRS and HIPEC was not improved by conversion therapy either, albeit a large numerical difference was noted in the conversion requested subgroup.

## Combination of neo-adjuvant Checkpoint Inhibition and Dendritic Cell Therapy (MesoPher) with Cytoreductive Surgery and Hyperthermic Intraperitoneal Chemotherapy in patients with Peritoneal Mesothelioma: rationale and design of the IMMUNOPEC trial

M. Emmers ^1^, C. Verhoef ^1^, J. Aerts ^1^, E. Madsen ^1^

^1^Erasmus Medical Center - Rotterdam (Netherlands)

**Background:** Peritoneal mesothelioma (PeM) is an uncommon but aggressive malignancy with a poor prognosis.(1) While, cytoreductive surgery (CRS) and hyperthermic intraperitoneal chemotherapy (HIPEC) improves survival outcomes, (2, 3) the majority of patients is not eligible for this surgical treatment. Even when complete cytoreduction is obtained, recurrence rates are high. Dendritic cell therapy (DCT) in the form of MesoPher has been shown to be safe in patients with PeM in the adjuvant setting (4) and a synergistic effect between anti-PD-1 and DCT has been seen. (5) To enhance the effect of DCT and facilitate feasibility for CRS-HIPEC, the addition of checkpoint inhibitors (CPI) in a neo-adjuvant setting could be a promising approach.

**Patients and methods:** The IMMUNOPEC trial will be an open-label, single center, single-arm, interventional study targeting adult patients with epithelioid PeM who are eligible for CRS-HIPEC. The intervention includes a diagnostic laparoscopy to check eligibility for CRS-HIPEC followed by leukapheresis of which monocytes are used for the differentiation to dendritic cells. These dendritic cells will be pulsed with autologous mesothelioma antigens (MesoPher). Patients will receive two cycles of chemotherapy consisting of carboplatin and pemetrexed during 6 weeks after leukapheresis in which the vaccines are prepared. Two DCT vaccination will be administered where the first vaccinations will be combined with anti-PD-1 check point inhibition over 4 weeks. Thereafter, CRS-HIPEC will be performed and three bi-weekly DCT vaccinations will follow 8 to 10 weeks after surgery.

The primary objective is to evaluate the efficacy based on the progression free survival of (neo-) adjuvant combination treatment with CPI and DCT around CRS-HIPEC. Secondary objectives are to determine the lymphocyte infiltration in the tumor combined with activation and proliferation markers, systemic immune response and radiological response. This analysis will reflect the effect of the vaccines on tumor recognition and anti-tumor activity.

## Intraperitoneal Paclitaxel Chemotherapy Induces Eosinophil Recruitment to the Peritoneal Cavity: Implications for Tumor Response in Gastric Cancer Peritoneal Metastasis

M. Matsumiya ^1^, K. Takahashi ^1^, H. Miyato ^1^, K. Kurashina ^1^, S. Saito ^1^, H. Ohzawa ^2^, H. Yamaguchi ^1^, Y. Hosoya ^1^, N. Sata ^1^, J. Kitayama ^1^

^1^Department of Surgery - Shimotsuke (Japan),

^2^Department of Clinical Oncology - Shimotsuke (Japan)

**Background:** Intraperitoneal (IP) administration of PTX with systemic chemotherapy shows strong efficacy against peritoneal metastases (PM) from gastric cancer (GC). However, it is unclear how immune cells in peritoneal cavity are related with the response of PM.

**Patients and methods:** Single cell suspensions were obtained from ascites or peritoneal lavages from 45 patients with PM from GC. In 25 patients, cells were additionally obtained after the treatment with IP-PTX with SOX regimen. Samples were immunostained with mAbs to CD326 (EpCAM), CD45, CD11b, CD19, CD14, CD66b, CD163, CD3, CD8, CD4, CD56, CD193 as well as DAPI and FVS780, and the rates of various subtypes in CD45(+) leukocytes were analyzed with flowcytometry.

**Results:** Tumor leukocyte ratio (TLR) calculated as CD326(+) tumor cells divided by CD45(+) leukocytes markedly decreased after IP-treatment in all patients (p<0.0001). Consequently, the rate of lymphoid cells mostly decreased, while that of CD11b(+) myeloid cells significantly increased in peritoneal cavity (p=0.016). Among them, the rates of CD14(+) macrophages and CD66b(+)CD16(+) neutrophils remained relatively unchanged. In contrast, CD66b(+)CD16(-) CD193(+) eosinophils generally increased after the IP-chemotherapy from base line. The rate of eosinophils before treatment did not correlate with any clinical findings. However, in 18 patients who showed negative peritoneal lavage cytology (CY0) post-treatment, eosinophils markedly increased (M=0.44%, 0.01-17.8% vs M=16.4%, 0.01-43.9% p<0.0001). The difference was not significant in 7 patients with CY1 post-treatment. The mean fluorescence intensities of CD11b and CD63 in the peritoneal eosinophils tended to be higher than those in peripheral blood. Notably, patients with an eosinophil ratio of 2% or higher after 1 course of the combination chemotherapy showed longer overall survival than their counterparts with marginal significance. (n=16 vs n=9, p=0.076)

**Conclusion:** IP-PTX appears to recruit potentially activated eosinophils to the peritoneal cavity, which may enhance the anti-tumor effects of chemotherapy against PM. The mechanisms are currently under investigation.

## Intestinal obstruction-free survival as an end-point for peritoneal surface disease studies

J. Franko ^1^, P. Singh ^1^, N. Castellano ^1^, V. Le ^1^

^1^MercyOne DSM - Des Moines (United States)

**Background:** Meaningful clinical endpoint for studies of peritoneal carcinomatosis is debated. Treatment sequencing with systemic therapy makes overall survival an unreliable endpoint for surgical studies (see PRODIGE-7). We hypothesized that the burden of bowel obstruction symptoms among pmCRC patients may be greater and propose obstruction-free survival as a preferred intermediate outcome measure for studies of pmCRC.

**Patients and methods:** We examined a cohort of synchronous mCRC patients diagnosed and fully treated in our center from 2012 to 2022. We observed the frequency of intestinal obstructive symptoms requiring nasogastric decompression (NGT) and/or total parenteral nutrition (TPN) occurring more than 14 days after the initial therapy for mCRC. We assessed cumulative inpatient length of stay, number of admissions, and the effect of weight loss over time.

**Results:** There were 248 patients with available data (58 with pmCRC [PM+], 190 mCRC without peritoneal involvement [PM-]). The incidence of emergency room or hospital admission was similar (PM-: 3.8±4.2 versus 3.9±3.8 admissions among PM+, p=0.940), but the cumulative length of stay was longer among those with pmCRC (PM-: 15.8±15.2 versus 25.5±23.4 days among PM+, p=0.001). PM+ patients experienced more frequent need for NGT decompression and/or TPN than PM- (21/58, 36.2% versus 20/190, 10.5%; p<0.001).

**Conclusion:** Bowel obstruction requiring TPN and NGT is a more frequent cause of inpatient care among pmCRC patients than mCRC patients without peritoneal involvement. These data support obstruction-free survival as a meaningful intermediate endpoint for studies including peritoneal disease and future CRS-HIPEC studies.

## Single-cell RNA sequencing reveals unprecedented insights into the tumor immune microenvironment of colorectal cancer peritoneal metastases

S. Ernst ^1^, J. Demuytere ^1^, J. Haerinck ^2^, J. De Coninck ^2^, J. Taminau ^2^, E. Lebegge ^3^, D. Laoui ^4^, J. Van Ginderachter ^5^, G. Berx ^2^, W. Ceelen ^1^

^1^Laboratory of Experimental Surgery, Department of Human Structure and Repair, Ghent University - Ghent (Belgium),

^2^Molecular and Cellular Oncology Laboratory, Department of Biomedical Molecular Biology, Ghent University - Ghent (Belgium),

^3^Laboratory of Myeloid Cell Immunology, VIB Center for Inflammation Research, Vrije Universiteit Brussel - Brussels (Belgium) - Brussels (Belgium),

^4^Laboratory of Dendritic Cell Biology and Cancer Immunotherapy, VIB Center for Inflammation Research, Vrije Universiteit Brussel - Brussels (Belgium),

^5^Laboratory of Myeloid Cell Immunology, VIB Center for Inflammation Research, Vrije Universiteit Brussel - Brussels (Belgium)

**Background:** Current therapies for colorectal cancer (CRC) peritoneal metastases (PM) demonstrate limited efficacy, underscoring the necessity for new therapeutic strategies. Immunotherapies represent a promising avenue; however, a detailed characterization of the tumor immune microenvironment (TIME) is lacking. Consequently, there is an urgent need within the field for an in-depth analysis of the CRC PM TIME.

**Patients and methods:** From patients providing informed consent, fresh surgical CRC PM samples were obtained from three anatomical locations: the abdominal wall, the small bowel mesentery, and the greater omentum. Peritoneal tissue without macroscopic evidence of metastasis and, when possible, matched primary CRC samples were also collected. The resected tumor and peritoneal tissues were analyzed using single-cell RNA sequencing (scRNA-seq) and flow cytometry (FC).

**Results:** More than 140.000 single cells from ten patients have been sequenced. Manual annotation of clusters indicates a substantial immune infiltration, representing all major immune cell types, such as granulocytes, lymphocytes, and myeloid cells, with T cells and tumor-associated macrophages (TAMs) being the predominant leukocytes. Further subclustering of these immune cell populations reveals details in an unparalleled manner. Within the T cell compartment, eleven clusters can be identified ranging from exhausted and regulatory CD4 T cells to effector CD8 T cells. Subclustering TAMs reveal a bivalent origin and five functionally diverse subclusters. Multi-color FC confirms similar immune infiltration patterns at the protein level, with the exception of an increased abundance of neutrophils. Both FC and scRNA-seq suggest discrete anatomical location-dependent differences.

**Conclusion:** Altogether, this data will provide the field unprecedented insights into the TIME of CRC PM, unveiling promising targets that can steer future therapeutic directions. This analysis will be accompanied by multiplex immunohistochemistry, providing spatial information on the TIME, and Luminex-based secretome analysis.

## Diagnostic Performance of DW-MRI in Advanced Ovarian Cancer: First Results of the Dutch Prospective Multicenter Mission Trial

E. Berardi ^1^, W. Van Driel ^1^, K. Gaarenstroom ^2^, C. Lok ^1^, M. Lopez ^1^, P. Van Meerten ^2^, J. Van Nederend ^3^, M. Engbersen ^1^, R. Hermans ^3^, M. Lahaye ^1^

^1^The Netherlands Cancer Institute - Amsterdam (Netherlands),

^2^LUMC - Leiden (Netherlands),

^3^Catherina Hospital - Eindhoven (Netherlands)

**Background:** Achieving a complete cytoreductive surgery (CRS) is crucial in ovarian cancer patients, making preoperative assessment of surgical feasibility essential. MRI has shown promising results in predicting the peritoneal cancer index (PCI) and the extent of peritoneal disease. We aimed to prospectively determine the performance of MRI in predicting PCI and feasibility of complete CRS.

**Patients and methods:** The MISSION study (ClinicalTrial registry, NCT03399344) was a prospective, multicentre trial conducted in four Dutch referral hospitals. Inclusion criteria were aged > 18 years and newly diagnosed Stage III/IV ovarian cancer. Two readers scored the mrPCI. The predictive performance of DW-MRI for the PCI and resectability is investigated based on Youden’s index in terms of positive predictive value, negative predictive value and bootstrapped area under the receiver operating characteristic curve (AUC), as well as intraclass correlation coefficients for inter-observer reproducibility. The surgical PCI (sPCI) and result of CRS were used as reference standard.

**Results:** Between June 2018 and June 2023, 270 patients were recruited, of whom 220 (193 interval and 27 primary CRS) were eligible for the current analysis. The mrPCI and sPCI were strongly correlated (ICC=0.71, p<0.0001). The AUC for predicting a complete primary CRS or interval CRS were 0.9 and 0.83 respectively. A very strong correlation was found between both readers for the mrPCI (ICC=0.81, p<0.0001).

**Conclusion:** MRI is an accurate and robust tool for predicting the extent of peritoneal metastases and is able to predict whether complete CRS can be achieved in patients with advanced ovarian cancer.

## CRS-HIPEC following Neo-adjuvant Bidirectional Pressurized Intraperitoneal aerosol chemotheraphy (PIPAC) with systemic chemotheraphy In Primarily Advanced inoperable epithelial ovarian cancer

C. Rohit Kumar ^1^, S.P. Somashekhar ^1^, K.R. Ashwin ^1^, V. Ahuja ^1^, D. Patil ^1^, A.M. Fernandes ^1^, K. Agarwal ^1^, E. Shanbagh ^1^, S. Vivek ^1^, C.N. Patil ^1^

^1^Aster International Institute Of Oncology - Bengaluru (India)

**Background:** PIPAC has shown improved objective response rate with improved quality of life in combination with IV chemotherapy when compared to IV chemotherapy alone in salvage situations. The role of intraperitoneal chemotheraphy in various forms is growing day by day in improving outcomes of inoperable advanced peritoneal malignacy.

**Patients and methods:** Patients with primarily inoperable advanced epithelial ovarian cancer who could not undergo CRS-HIPEC were challenged with bidirectional PIPAC & IV chemotheraphy as a salvage situation after discussion in MDT. Patients who subsequently underwent CRS-HIPEC were analysed to study their clinical characteristics, extent of disease and peri-operative outcomes. PIPAC was given with Cisplatin 15mg/m2 and doxorubicin 3mg/m2. Intravenous chemotheraphy was given within one week of PIPAC. Each cycle was repeated once in 4-5 weeks.

**Results:** 120 PIPAC applications were done in 45 patients. 30 patients received 3 cycles of PIPAC with IV chemotheraphy given within one week of PIPAC. Out of 45 patients 28 underwent CRS-HIPEC subsequently. Mean age 54.5±10.74, PCI 15±5, duration of surgery 9.6±1.2 hrs. The mean drop in PCI who completed 3 PIPAC with IV chemotheraphy was 5±0.8. Out of 45 patients nearly 60% showed PRGS 1, 25% PRGS 2 and 15% PRGS 3. 52.4% had total peritonectomy, 12.7% had multivisceral resection, 55.8% had one bowel resections and stoma rate was 3.5%. Overall G3-G5 morbidity was 25.4% with major ones being post-operative intra-abdominal collection (21.8%), electrolyte imbalance (16.4%), pulmonary (16.4%) followed by hematological (12.7%). The 30 day mortality was 3.8%.

**Conclusion:** Neo-adjuvant PIPAC with Intravenous chemotheraphy might be a promising combination for inducing good response rates in advanced epithelial ovarian carcinoma. CRS-HIPEC following neo-adjuvant PIPAC + IV chemotheraphy is safe, feasible and tolerable. PIPAC repeated every 4-5 weeks along with systemic chemotheraphy provides a ideal scenario to monitor & assess response to loco regional and systemic chemotheraphy.

## Utility of Left Lateral Liver Lobectomy in Removing Retrohepatic Lesions during Cytoreductive Surgery for Low-grade Mucinous Carcinomatosis

A. Nikiforchin ^1^, A. Sardi ^1^, M.C. King ^1^, S. Iugai ^1^, V. Gushchin ^1^

^1^Mercy Medical Center - Baltimore (United States) - Baltimore (United States)

**Video URL:** https://drive.google.com/file/d/19YBcl9xgJqd1pB2IjShvRtTcEdc73DMz/view?usp=sharing

**Background:** Complete removal of tumor during cytoreductive surgery (CRS) is essential for a favorable prognosis in patients with peritoneal surface malignancies (PSM). Despite various surgical techniques, achieving a complete cytoreduction (CC) can be challenging when peritoneal lesions are spread throughout the upper abdomen, particularly in the retrohepatic space.

**Patients and methods:** Our step-by-step surgical video presents a complex CRS in the upper abdomen featuring a left lateral liver lobectomy as a practical way to access retrohepatic lesions and achieve CC.

**Results:** A 39-year-old male with a low-grade appendiceal mucinous neoplasm and extensive peritoneal disease presented to our PSM institution after unsuccessful treatment with multiple paracenteses and systemic chemotherapy. We recommended an attempt at CRS with hyperthermic intraperitoneal chemotherapy (HIPEC).

The surgery began with an anterior parietal peritonectomy and greater omentectomy, allowing us to assess the extent of the disease (PCI 34). After mobilizing the colonic splenic flexure, we performed a splenectomy, distal gastrectomy, bursectomy, and bilateral diaphragmatic peritonectomies. Palpated perihepatic and retrohepatic tumor implants extended from the porta hepatis to the left hepatic vein. To remove these difficult-to-reach lesions along the ligamentum venosum and around the first liver segment and the vena cava, we performed a left lateral lobectomy. With this approach, we could safely remove all these lesions and achieve a complete cytoreduction.

After finishing CRS (PCI 0, CC-0) with liver capsulectomy, bowel resections, and pelvic peritonectomy, we performed a 90-minute HIPEC (Mitomycin-C 40 mg, target temperature 41-43°C). The patient did not experience major postoperative complications and, at his 11-month follow-up, he had no signs of disease.

**Conclusion:** Left lateral liver lobectomy is a valuable addition to the CRS arsenal, allowing for a complete cytoreduction in the retrohepatic space when the tumor spreads along the ligamentum venosum, the first liver segment, and around the vena cava.

## Effects of center’s experience on outcomes of Cytoreductive surgery and HIPEC in rare peritoneal diseases

S. Kusamura ^1^, O. Glehen ^2^, Y. Yonemura ^3^, A. Sardi ^4^, D. Goere ^5^, P.H. Sugarbaker ^6^, M. Brendan ^7^, E.A. Levine ^8^, D. Morris ^9^, M. Deraco ^1^

^1^Fondazione IRCCS Istituto Nazionale Tumori Milano - Milano (Italy),

^2^Centre Hospitalo-Universitaire Lyon-Sud - Lyon (France),

^3^Nonprofit Organization to Support Peritoneal Surface Malignancy Treatment - Kishiwada (Japan),

^4^The Institute for Cancer Care, Mercy Medical Center - Baltimore (United States),

^5^Gustave Roussy Cancer Institute - Paris (France),

^6^Washington Hospital Center - Washington (United States),

^7^Basingstoke and North Hampshire National Health Service Foundation Trust - Basingstoke (United Kingdom),

^8^Wake Forest University Baptist Medical Cente - Winston-Salem (United States),

^9^University of New South Wales, St George Hospital - Sydney (Australia)

**Background:** This study aimed to investigate the impact of a center’s experience on outcomes following cytoreductive surgery (CRS) and hyperthermic intraperitoneal chemotherapy (HIPEC) for rare peritoneal diseases such as pseudomyxoma peritonei (PMP) and diffuse malignant peritoneal mesothelioma (DMPM).

**Patients and methods:** The analysis utilized the PSOGI/RENAPE databases, focusing on patients with DMPM or PMP treated with CRS with or without HIPEC. Cases with missing data, iterative procedures, and non-DMPM/PMP histologies were excluded. Kaplan-Meier plots and Cox model analyses were conducted, incorporating covariates such as patient demographics, disease characteristics, treatment specifics, and parameters related to center experience (case number and annual caseload). Outcomes assessed were overall survival (OS) and 90-day mortality. Continuous variables were transformed using restricted cubic splines. Analyses were performed separately for DMPM and PMP.

**Results:** From December 1989 to May 2023, 2,429 DMPM cases and 7,616 PMP cases were treated. For DMPM, the median center case number was 47 (IQR: 15-77), and the annual caseload was 8.4 (IQR: 3.1-10.7). For PMP, the median center case number was 120 (IQR: 35-298), and the annual caseload was 15.0 (IQR: 6.9-21.4).

Multivariate analysis showed that an increased annual caseload was independently associated with improved OS in a non-linear manner for both diseases. Specifically, for DMPM, the adjusted log hazard ratio (HR) for survival was significantly lower when the annual caseload exceeded 8 cases. For PMP, the adjusted log HR for survival was significantly lower when the annual caseload exceeded 32 cases. No correlation was found between center experience and 90-day mortality.

**Conclusion:** The analyses confirm the significant impact of a center’s experience on the outcomes of DMPM and PMP after CRS/HIPEC. Given the rarity of these diseases, centralization and large regional referral systems, as well as networking, are crucial to ensure high-quality treatment, as they enable centers to build the necessary experience to meet current standards.

## Utility of peritoneal lavage fluid for RNA profiling of peritoneal metastasis

S. Kulatheivam ^1^, L.L.M. Jakobsen ^1^, A. Ainsworth ^1^, M. Burton ^1^, C. Fristrup ^1^, M. Graversen ^1^, M.B. Mortensen ^1^, P. Pfeiffer ^1^, L.S. Tarpgaard ^1^, S. Detlefsen ^1^

^1^Odense University Hospital - Odense (Denmark)

**Background:** New methods for assessment of treatment response, prognostication and prediction of patients with peritoneal metastasis (PM) are needed. Utility of formalin-fixed and paraffin embedded (FFPE) sediments from peritoneal lavage fluids (PLFs) for transcriptomic analysis of PM has not been elucidated. Using PM from pancreatic cancer (PM-PC) for proof of concept, we aimed to: 1) Evaluate whether FFPE sediments from PLFs with PM are suitable for mRNA expression profiling, 2) identify dysregulated mRNAs related to >40 pathways involved in tumor biology in PM-PC vs. Controls, and 3) evaluate whether certain mRNAs hold prognostic value.

**Patients and methods:** PLFs from 19 PM-PC patients scheduled for Pressurized Intraperitoneal Aerosol Chemotherapy (PIPAC) and ascitic fluids from 16 benign ascites specimens (Controls) were included in this study. From PM-PC patients, only PLFs positive for malignant tumor cells were used. From FFPE blocks, RNA was extracted and used for expression profiling of 760 genes, using nCounter (NanoString) and the Tumor Signaling 360 Panel. Unsupervised clustering analysis, differential gene expression (DGE) in PM-PC vs. Controls, and, for the 20 most upregulated genes in PM-PC, DGE for short-term (STS) vs. long-term survival (LTS, defined as survival ≥ 15 months from PM-PC diagnosis) was performed. For survival analysis, KM curves, log rank test and Cox proportional hazards were calculated.

**Results:** For PM-PC vs. Controls, DGE identified 56 upregulated and 130 downregulated genes (false discovery rate (FDR) ≤ 0.05). In PM-PC STS vs. LTS, there was upregulation of *KRT17* (P=0.012). High expression of *KRT17* was negatively associated with survival (HR 4.4, P=0.0052).

**Conclusion:** PLF from PM-PC patients is suitable for transcriptomic analysis. Numerous cancer-related genes were upregulated in PM-PC vs. Controls. We found a negative association of *KRT17* with survival, which should be validated in future studies. The clinical value of RNA profiling using PLFs in PM should be explored further.

## Establishing an Onsite Surgical Training Centre at a Major Teaching University Hospital & National Peritoneal Malignancy Institute in Dublin, Ireland

M.F. Khan ^1^, D. Killeen ^2^, C. Shields ^1^, C. Thomspon ^1^, D. Brennan ^1^, J. Mulsow ^1^, R. Cahill ^1^

^1^Mater Misericordiae University Hospital - Dublin (Ireland),

^2^University College Dublin - Dublin (Ireland)

**Background:** Mater Misericordiae University Hospital was founded in 1861 and is one of the major teaching university hospitals in Ireland. It is also the national referral centre for peritoneal malignancy in the country. The Peritoneal Malignancy Insititute (PMI) itself was established in May 2013. A unique set up within the hospital was the availability of the original old surgical theatres. This space was transformed into an onsite surgical training centre. The centre offers a comprehensive range of models for training and research, including access to human cadaveric models at postgraduate level thanks to partnership with UCD Anatomy and Medical Council accreditation.

**Patients and methods:** Established in August 2017, the centre has hosted 100 cadaveric workshops, 50+ animal tissue wet labs, regular general and specialty-specific skills workshops with synthetic models and simulation trainers, device development and testing workshops, equipment demo and trialling sessions as well as weekly undergraduate simulation sessions and skills teaching throughout the academic year. Services have been provided to fourteen specialties and to allied health professions.

**Results:** Over 500 trainee surgeons and 160 consultant surgeons have benefitted from cadaveric workshops at the centre. The centre delivers regular study days and skills training in collaboration with RCSI, RCPI Institute of Obstetricians and Gynaecologists and the College of Anaesthesiologists of Ireland. Culminating in two very successful, Gyne Oncology lead workshops in peritoneal malignancy with visiting teams from Belfast & London, completed with high fidelity cadaveric surgical hand on training session and with live CRS & HIPEC cases to observe.

**Conclusion:** A unique opportunity has arisen to allow for “on-site” high fidelity training. This establishment can give access to surgical trainees and trainers alike to use its affiliated capabilities on a regular & frequent basis, especially in peritoneal malignancy amongst other subspecialities.

## Immune checkpoint inhibitors for patients with peritoneal metastases of dMMR colorectal cancer.

A.G. Aalbers ^1^, P. Snaebjornsson ^2^, M. Chalabi ^3^, K. Bolhuis ^3^, M. Lahaye ^4^, N.F. Kok ^1^

^1^Department of Surgery, Netherlands Cancer Institute - Amsterdam (Netherlands),

^2^Department of Pathology, Netherlands Cancer Institute - Amsterdam (Netherlands),

^3^Department of Medical Oncology, Netherlands Cancer Institute - Amsterdam (Netherlands),

^4^Department of Radiology, Netherlands Cancer Institute - Amsterdam (Netherlands)

**Background:** Immunotherapy has become the cornerstone of treatment for patients with mismatch repair deficient (dMMR) colorectal cancer. However, the efficacy of immune checkpoint inhibitors is lower in patients with metastatic as compared to localized colorectal cancer. Data on immunotherapy in patients with colorectal peritoneal metastases (CRPM) is scarce and further research is necessary.

Objectives: to compare the disease free survival (DFS) and overall survival (OS) of patients with dMMR CRPM in the following two treatment subgroups: CRS-HIPEC without perioperatieve checkpoint inhibition (group 1) and immune checkpoint inhibition with or without surgery (group 2).

**Patients and methods:** From our prospectively maintained tertiary referral centre database, all patients diagnosed with histopathologically proven dMMR CRPM were selected. These patients underwent CRS-HIPEC and/or immunotherapy. Mismatch repair (MMR) testing and mutational analysis was performed. DFS and OS were main outcomes.

**Results:** Of 41 patients included 26 patients underwent CRS-HIPEC and had no perioperative immune checkpoint inhibition treatment and 15 patients underwent either surgery with neoadjuvant immune checkpoint inhibition (n=6) or were treated with immunotherapy without surgery (n=9).

After a median follow up of 23.0 months, median DFS of patients without versus with immunotherapy was 10.0 (95% confidence interval [CI] 6.7-13.3) versus 39.0 months (95% CI 14.1-63.9, p=0.002). Corresponding median OS was 41.0 months (95% CI 5.9-76.1) versus not reached, all patients alive, respectively (p=0.021). In patients who had underwent surgery after neoadjuvant immune checkpoint inhibition therapy, heterogeneous histopathological responses were observed reflected by Mandard tumor regression grades varying from 1 to 4.

**Conclusion:** Immunotherapy prolongs survival in patients with dMMR CRPM. The optimal combination of checkpoint inhibitors (single vs. dual therapy), duration of immunotherapy and timing and necessity of adjuvant surgery is not clear yet. We would like to discuss our plan for a prospective registry for patients with dMMR CRPM at the meeting.

## Patient-derived Decellularized Extracellular Matrix Scaffolds to Develop an ex vivo Model of Pseudomyxoma Peritonei in a More Relevant Biological Context

L. Varinelli ^1^, M. Di Bella ^1^, D. Battistessa ^1^, F. Pisati ^2^, C. Paolino ^1^, G. Sabella ^3^, M. Guaglio ^4^, M. Deraco ^4^, M. Gariboldi ^1^, S. Kusamura ^4^

^1^Department of Experimental Oncology, Molecular Epigenomics Unit, Fondazione IRCCS Istituto Nazionale Tumori, via G. Venezian 1, 20133 Milan, Italy. - Milan (Italy),

^2^Cogentech Ltd. Benefit Corporation with a Sole Shareholder, via Adamello 16, 20139 Milan, Italy. - Milan (Italy),

^3^1st Pathology division, Department of Phatology and Laboratory Medicine, Fondazione IRCCS Istituto Nazionale dei Tumori di Milano, via G. Venezian 1, 20133, Milan, Italy. - Milan (Italy),

^4^Peritoneal Surface Malignancies Unit, Department of Surgery, Fondazione IRCCS Istituto Nazionale Tumori, via G. Venezian 1, 20133, Milan, Italy - Milan (Italy)

**Background:** Pseudomyxoma peritonei (PMP) is a rare tumor characterized by progressive accumulation of mucin within the abdominal cavity. Its pathophysiology might be connected to an immunosuppressive tumor microenvironment (TME) induced by the activated A2-adenosinergic axis. The impact of TME on response to current chemotherapies has been widely demonstrated. Using 3D-decellularized-Extracellular Matrix (3D-dECM) as a scaffold for cell growth makes it possible to recreate an in vivo-like environment to host patient-derived organoids (PDO) and other TME components to foster treatments development.

**Patients and methods:** PDO and cancer-associated fibroblast (CAF) models from PMP patients were developed and characterized in terms of 3D structures, immunohistochemical profile and genetic landscape. ECMs from the same patients were decellularized using a procedure based on detergents and enzymatic digestion. The main features of the obtained 3D-dECMs were characterized by immunohistochemistry. The 3D-dECMs were repopulated with PDOs and/or CAF and imaging and immunohistochemical approaches were used to demonstrate that the developed models maintained the main characteristics of PMP tissue.

**Results:** 3D-dECMs obtained from patients with PMP allowed the growth of PDOs, favoring the development of three-dimensional nodules that maintained the characteristics of in vivo PMP in terms of histology and morphology. Also, PMP-derived CAFs were able to grow onto the 3D-dECMs, colonizing the scaffolds alongside the sites previously occupied by the native stromal cells. Finally, 3D-dECMs repopulated with PMP-derived PDOs and CAFs reproduced the main clinical features of PMP diseases in terms of morphology and growth pattern.

**Conclusion:** The described ex vivo 3D model, obtained by combining patient-derived extracellular matrices depleted of cellular components with PMP-derived PDOs and CAFs to mimic the metastatic niche of the PMP disease, could be an innovative tool for developing new therapeutic strategies in a biologically relevant context, personalizing treatments, and increasing their efficacy.

## Prognostic value of DNA ploidy, stroma, and nucleotyping in colorectal synchronous peritoneal metastasis

X. Qin ^1^, W. Deng ^2^, K. Zhong ^3^, Y. Huang ^2^, H. Wang ^1^, Q. Qin ^1^

^1^Department of Colorectal Surgery, the Sixth Affiliated Hospital, Sun Yat-sen University - Guangzhou (China),

^2^Department of Pathology, the Sixth Affiliated Hospital, Sun Yat-sen University - Guangzhou (China),

^3^Department of Gastrointestinal, Shenzhen People’s Hospital - Guangzhou (China)

**Background:** Heterogeneity concerning survival in synchronous peritoneal metastasis from patients with colorectal cancer exists, thereby further classification is urgently required.

Objective: this study aimed to investigate the prognostic value of DNA ploidy, stroma-tumor fraction, and nucleotyping (PSN) in the prognosis of synchronous peritoneal metastasis.

**Patients and methods:** A total of 144 patients who underwent cytoreductive surgery for synchronous peritoneal metastasis from colorectal cancer at the Sixth Affiliated Hospital of Sun Yat-sen University from 2007 to 2019 were included. A novel scoring system on the basis of DNA ploidy (0 for diploid and 1 for aneuploid or tetraploid), stroma-tumor fraction (0 for ≤ 0.5 or 1 for >0.5), and nucleotyping of primary tumor (0 for >0.044 or 2 for ≤ 0.044) were produced by automated digital imaging. PSN classification was set as good (≤1 point ) or bad (>1 points).

**Results:** The median overall survival was 14.1 (95% CI, 12.2 – 20.4) months in the PSN-bad group and 24.3 (95% CI, 19.0 – 30.4) in the PSN-good group. The median progression-free survival was 6.8 (95% CI, 5.2 – 11.0) months in the PSN-bad group and 12.3 (95% CI, 8.5 – 16.2) in the PSN-good group.

**Conclusion:** PSN classification shows goog prognostic value for synchronous peritoneal metastasis from colorectal cancer.

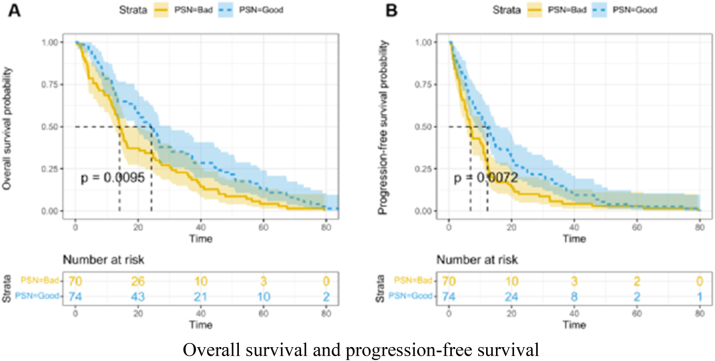



## The landscape of practice and training in the peritoneal surface malignancy field

C. Chia ^1^, M. Hubner ^2^, T. Dellinger ^3^, L. Bijelic ^4^, M. Alyami ^5^, S.P. Somashekhar ^6^, D. Cortes-Guiral ^7^, O. Sgarbura ^8^

^1^National Cancer Centre Singapore - Singapore (Singapore),

^2^Lausanne University Hospital - Lausanne (Switzerland),

^3^City of Hope Comprehensive Cancer Centre - California (United States),

^4^Centro Medico Teknon - Barcelona (Spain),

^5^King khalid Hospital - Najran (Saudi Arabia),

^6^aster international institute of oncology - Bangalore (India),

^7^Viamed Institute of Advanced Surgical Oncology - Madrid (Spain),

^8^cancer institute montpellier - montpellier (France)

**Background:** The field of peritoneal surface malignancy (PSM) has emerged and grown over the last 4 decades. Current practicing surgeons come from various subspecialties and there is no standardization in training. This survey aims to give an insight into the current landscape of surgeons in the field and how they were trained. We also delve into the parameters of an ideal peritoneal unit, surgeon and training.

**Patients and methods:** An online survey consisting of 24 questions was disseminated to practicing surgeons in the PSM field.

**Results:** There were a total of 208 respondents. 92.8% of the respondents were between the ages of 30-60. 75.4% were males. 62.8% were surgical oncologists with the other common subspecialties being colorectal, upper gastrointestinal, gynae-oncology. 46.4% were from Asia followed by 31.9% from Europe and 13.5% from South America. Only 35% did a fellowship in PSM but 86.5% felt that a fellowship was needed after residency. Majority felt that an expert centre should be able to offer all types of treatment options, should manage between 50-100 cases per year and perform 30-50 cytoreductive surgeries per year. 57% felt that a surgeon should be able to operate on all sites in the abdomen. 71.5% felt that some form of publication was necessary. The most common reason cited for choosing this field was because of the diversity of the surgery. Obstacles to the future included hyper-specialisation of surgery as well as work life balance for surgeons.

**Conclusion:** In this evolving field of PSM, the current practicing community is of the majority opinion that a proper fellowship in PSM is required. This should be actively looked into for future development of the field. The diversity and complexity is what attracted surgeons to this field but hyper-specialization could hinder the field’s future development and future training should take these needs into consideration.

## The influence of screening on colorectal peritoneal metastases in the Netherlands: The effect on incidence, treatment and survival, a synopsis of two Dutch cohort studies

L. Galanos ^1^, F. Van Erning ^2^, I. De Hingh ^1^

^1^Catharina hospital - Eindhoven (Netherlands),

^2^Netherlands Comprehensive Cancer Organisation - Utrecht (Netherlands)

**Background:** Colorectal cancer screening was implemented in 2014 to combat the elevating disease burden in the Netherlands. This abstract summarizes two retrospective Dutch cohort studies on the impact of screening on advanced-stage disease, comprising of colorectal peritoneal metastases (CPM).

**Patients and methods:** Data was used from the Netherlands Cancer Registry for both cohort studies and comprised of screen-eligible patients aged 55-75 years old. The first study compared screen-detected and clinically detected CPM patients, diagnosed in 2014-2020. Treatment was compared between screen-detected CPM patients and clinically detected CPM patients using χ2-tests. Overall survival (OS) was compared between both groups with the log-rank test. The second study provided an overview of the CPM incidence between 2009-2022. Crude rates of CPM incidence and cumulative CPM incidence were calculated and compared with the expected CPM incidence rates. Expected incidence was calculated based on the incidence rate prior to screening (2009-2013).

**Results:** The first study included 2773 synchronous CPM patients, 7% were detected by screening. It was shown that 28% of screen-detected patients underwent CRS-HIPEC versus 14% in the clinically detected group (p<0.001). OS was 20.0 months (IQR 9.7–51.7) in the screen-detected group versus 10.8 months (IQR 3.4–25.5) in the clinically detected group (p<0.001). The second trial observed that the CPM incidence shows an overall increase: 5.1 (2009) vs. 8.8 (2022) per 100,000 individuals. However, in the period after initiation of screening the observed CPM incidence stabilized, from 8.6 (2015) vs. 8.9 (2022) per 100,000 individuals. The observed and expected number of CPM differed significantly in the screen-eligible population (observed 6,437 individuals vs. expected 7,992 individuals; p<0.001).

**Conclusion:** Screen-detected CPM patients received more curative treatment and had better OS compared to clinically detected patients. Screening led to a stabilization in CPM incidence among screen-eligible patients. These findings confirm that screening helps to combat CPM.

## US Multicenter Dose-escalation Phase 1 trial of Mitomycin C Pressurized Intraperitoneal Aerosolized Chemotherapy in Combination with Systemic Chemotherapy for Appendiceal and Colorectal Carcinomatosis: Interim Results

M. Raoof ^1^, M. Fakih ^1^, P. Frankel ^1^, S. Chang ^1^, R. Whelan ^2^, D. Deperalta ^2^, A. Merchea ^3^, T. Dellinger ^1^

^1^City of Hope Cancer Center - Duarte (United States),

^2^Northwell Health - New York (United States),

^3^Mayo Clinic - Jacksonville (United States)

**Background:** PIPAC is a promising minimally invasive approach to treat unresectable colorectal/ appendiceal peritoneal metastases. Mitomycin C (MMC)-PIPAC in combination with systemic chemotherapy has not been previously investigated.

**Patients and methods:** In this US multicenter phase I study of MMC-PIPAC, patients who were unresectable after 4+ months of first- or second-line systemic chemotherapy were included. MMC-PIPAC was given every 6 weeks and systemic FOLFIRI was given every 2 weeks except the week of PIPAC. Dose escalation of MMC-PIPAC was performed at 4 dose levels – DL (7, 12.5, 19, and 25 mg/m^2^) in a 3+3 design. The primary endpoint was to assess the safety and to establish the recommended Phase 2 dose.

**Results:** A total of 17 patients (10 males: 7 females) have been enrolled with a median age of 55y (range 31-74y). Most common treatment-related toxicities were wound infection/ dehiscence (grade-2 in 4, and grade-3 in 1 patient), nausea/ vomiting (grade-2, 3 patients), and diarrhea (grade-2, 2 patients). One patient in DL-3 had a DLT (small bowel obstruction) and was taken off study. Nine patients on DL-1/2 who had at least 2 cycles of PIPAC were included in efficacy analysis. One patient had complete radiographic response, 1 had partial response and 7 had stable disease. Histologic laparoscopic, and CEA responses were observed in most patients (Fig 1).

**Conclusion:** MMC-PIPAC is safe at DL-2 in combination with FOLFIRI but DL-3 requires ongoing study to establish recommended phase 2 dose. At DL1 & 2, histologic, laparoscopic, radiographic and biomarker responses were observed in most patients.

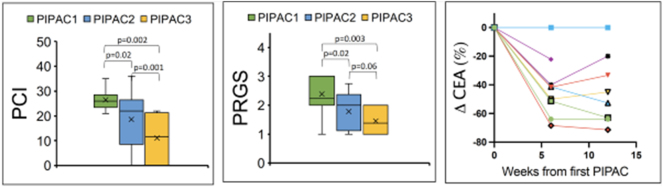



Laparoscopic. Histologic, and CEA Response.

## Rationale and Study Design of the KOV-HIPEC-02R: A Randomized, Multicenter, Open-label Phase III trial of Hyperthermic Intraperitoneal Chemotherapy in Platinum-Resistant Recurrent Ovarian Cancer

L. Myong Cheol ^1^, K. Ji Hyun ^1^, B. Jae Kyung ^1^, K. Uisuk ^1^, K. Junhwan ^1^, K. Jin Hee ^1^, P. Sang-Yoon ^1^

^1^National Cancer Center Gynecologic Cancer Center - Goyang-si, Gyeonggi-do (Korea, Republic of)

**Background:** Hyperthermic intraperitoneal chemotherapy (HIPEC) administered during interval cytoreductive surgery following neoadjuvant chemotherapy has shown to increase progression-free survival (PFS) and overall survival (OS) rates, as indicated by the OV-HIPEC-01 and KOV-HIPEC-01 trials. A recent meta-analysis (Kim SI, Kim JH, et al., GO 2023) demonstrated a survival benefit associated with HIPEC, particularly after recent chemotherapy exposure. Moreover, in ovarian cancer (OC), HIPEC is suggested to be effective in overcoming chemotherapy resistance.

**Patients and methods:** This trial (KOV-HIPEC-02) is a multicenter, open-label, 1:1 randomized, phase III trial that will enroll 140 patients with platinum-resistant recurrent epithelial ovarian cancer (NCT05316181). After cytoreductive surgery, patients undergo the HIPEC procedure at 41.5°C, with doxorubicin at 35mg/m^2^ and mitomycin at 15mg/m^2^. Enrolled patients receive non-platinum compound systemic chemotherapy until disease progression. The primary objective is to evaluate progression-free survival (PFS) between the HIPEC group and the control group. Secondary objectives include overall survival (OS), cancer-specific survival, and safety and quality of life. Considering a 3-year enrollment period, 2-year follow-up, and a statistical power of 80%, 140 patients are needed, accounting for a 10% dropout rate. As of May 31, 2024, 104 patients (74.2%) have been randomized.

**Results:** There are no available results at the time of submission.

**Conclusion:** The role of cytoreductive surgery and HIPEC in platinum-resistant recurrent ovarian cancer will be elucidated for the first time through this randomized trial (KOV-02R).

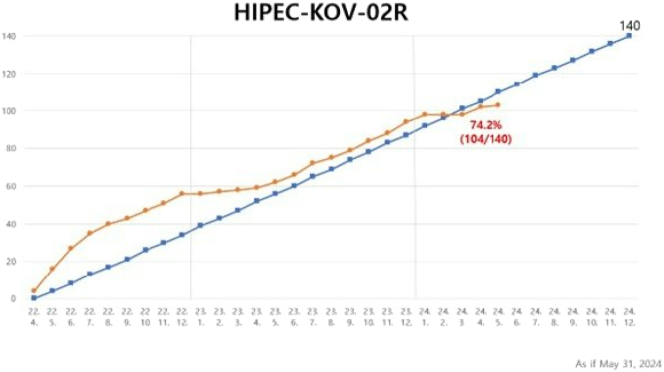



## Phase I PIANO Trial - PIPAC-Oxaliplatin and Systemic Nivolumab Combination for Gastric Cancer Peritoneal Metastases: Clinical and Translational Outcomes

H.L. Tan ^1^, W.P. Yong ^1^, D. Chia ^2^, C. Chia ^3^, J. Ong ^3^, W. Ceelen ^4^, W. Wouter ^4^, P. Tan ^5^, R. Sundar ^1^, J. So ^2^

^1^National University Cancer Institute, Singapore - Singapore (Singapore),

^2^Department of Surgery, National University Hospital, National University Health System, Singapore - Singapore (Singapore),

^3^National Cancer Centre, Singapore - Singapore (Singapore),

^4^Ghent University Hospital - Ghent (Belgium),

^5^Duke-NUS Medical School, Singapore - Singapore (Singapore)

**Background:** Pressurized intraperitoneal aerosol chemotherapy (PIPAC)-oxaliplatin (OX) induces direct DNA-damage and immunogenic cell death in patients who have gastric cancer with peritoneal metastases (GCPM). Combining PIPAC-OX with immune checkpoint inhibition remains untested. We conducted a phase I first-in-human trial evaluating the safety and efficacy of PIPAC-OX combined with systemic nivolumab.

**Patients and methods:** Patients with GCPM who had disease progression on at least first-line systemic chemotherapy were recruited across three centres in Singapore and Belgium. Patients received PIPAC-OX at 90mg/m2 6-weekly and i.v. nivolumab 240mg 2-weekly. Translational analyses were performed on tumor samples acquired during PIPAC procedures.

**Results:** 18 patients with GCPM were prospectively recruited. The PIPAC-OX and nivolumab combination was well tolerated with few treatment-related adverse events, although there was one grade 4 vomiting adverse event. The median decrease in peritoneal carcinomatosis index (PCI) was -5 (IQR: -12 to +1) and -7 (IQR: -6 to -20) and Peritoneal Regression Grade 1 or 2 was observed in 66.7% (6/9) and 100% (3/3) at 2nd PIPAC and 3rd PIPAC respectively. Translational analyses of GCPM samples showed that higher PCI scores correlated with increased T-cell exhaustion and PI3K-AKT MTOR signalling within adjacent normal peritoneum. Post-treatment samples were immune-infiltrated with increased T-cell proportions within peritoneal tumors.

**Conclusions:** The first-in-human PIANO trial of combination PIPAC-OX and nivolumab demonstrated safety and tolerability, as well as enhanced T-cell infiltration within peritoneal tumors. This trial sets the stage for future combinations of systemic immunotherapy with locoregional intraperitoneal treatments.

## The EPICH Trial: a multicenter pre-post study to implement ERAS Program for patients with peritoneal surface malignancies treated by Citoreductive Surgery and HIPEC

M. Robella ^1^, E. Pagano ^2^, M. Vaira ^1^, A. Evangelista ^2^, A. Cerutti ^1^, F. Borghi ^1^

^1^Candiolo Cancer Institute, FPO - IRCCS - Torino (Italy),

^2^Unit of Clinical Epidemiology, Azienda Ospedaliero Universitaria Città della Salute e della Scienza di Torino - Torino (Italy)

**Background:** Cytoreductive surgery and hyperthermic intraperitoneal chemotherapy (CRS/HIPEC) are complex procedures for the treatment of peritoneal surface malignancies. ERAS protocols aim to standardize preoperative, intraoperative and postoperative pathways to improve patient care.

Although ERAS guidelines specific to CRS/HIPEC have recently been published, evidence is still scarce and the program is not yet standardized in Italy. The aim of this study is to evaluate the impact of ERAS implementation in peritoneal cancer surgery in all Italian referral centers supported by an audit and feedback intervention.

**Methods:** Design: a multicenter pre-post study design.

During 12-month, approximately 300 patients will be included. In the first period (approximately 4 months, 100 expected patients) usual care will be described. In the second period (approximately 8 months, 200 expected patients) participating centers will apply an ERAS protocol defined and agreed upon by the centers. The study will involve the creation of a close-knit multidisciplinary team consisting of a surgeon, anesthesiologist, dietician, physiotherapist and nurse to apply and test the principles of the protocol.

Primary endpoint: length of stay (LOS) without outliers (>90°percentile).

Secondary endpoints: level of adherence to ERAS items, postoperative complications, intensive care unit LOS, reinterventions/readmissions rate, time to recovery of bowel function, quality of postoperative recovery as measured by dedicated questionnaire.

The sample of around 300 patients will allow to verify the hypothesis of reducing the average hospital stay (excluding outliers) from 14 to 12 days, assuming a standard deviation of 4 days (corresponding to an effect size of 0.5), with a one-tailed alpha error of 5% and a statistical power of 95%. Subgroups analyses will be performed according to peritoneal cancer index (PCI) and surgical complexity.

Estimated dates for completing accrual: September 2025. Results presented on January 2026.

The study protocol is being evaluated by the ethical committee of the coordinating center.

## Failure to intended adjuvant treatment impair prognosis in patients with gastric peritoneal carcinomatosis treated with cytoreductive surgery and HIPEC

J. Pinson ^1^, A. Grancher ^2^, P.E. Bonnot ^3^, J.J. Tuech ^1^, L. Villeneuve ^4^, V. Kepenekian ^4^, O. Glehen ^4^

^1^CHU de Rouen - Service de chirurgie oncologique et viscérale - Rouen (France),

^2^CHU de Rouen - Service d’oncologie digestive - Rouen (France),

^3^Centre chirurgical Lyon Mermoz - Lyon (France),

^4^CHU de Lyon - Service de chirurgie oncologique et viscérale - Lyon (France)

**Background:** Cytoreductive surgery (CRS) and hyperthermic intraperitoneal chemotherapy (HIPEC) are associated with complications, and disability that can prevent some patients from receiving subsequent adjuvant treatments. Inability to complete all intended adjuvant therapies might impair prognosis in survival terms. This study evaluated the impact of Return to Intended Oncological Treatment (RIOT) on survival as well as risk factors that could prevent RIOT in patients who underwent CRS and HIPEC for gastric carcinomatosis (GC).

**Patients and methods:** Outcomes for 178 patients who underwent CRS and HIPEC for GC and for whom adjuvant treatment was indicated, in two French institutions from 1989 to 2022, were examined. The log-rank test was used to compare survivals and Cox regression models to identify risk factors.

**Results:** Of the 178 patients whom RIOT was initially offered, only 108 (60.7%) could follow adjuvant therapy. Non-RIOT patients had higher ASA-score (p=0,026), less neoadjuvant therapy (73% vs 85%, p=0.043), more CC-score (14% vs 5.5%, p=0.047), more pT4 tumor (74% vs 55%, p=0,008). No differences were found concerning PCI, pN status or presence of signet ring cells. Return-to-theatre rate was higher for Non-RIOT patients (37% vs 11%, p<0.001) but grades 3-5 morbidity rate did not differ significantly (49% vs 35%, p=0.07). In multivariate analyses, risk factors associated with inability to RIOT were return-to-theatre (OR 8.3, p< 0.001), and pT4 tumor status (OR 3.1, p = 0.08). Patients unable to RIOT had shorter overall survival (OS) (median OS from HIPEC: 8 vs 19 months, p=0.004) but disease free survival did not differ significantly (6 vs 9 months, p = 0.2).

**Conclusion:** RIOT alter CRS and HIPEC improves survival in patients with GC. Pre- and intra-operative patient selection and improving post-operative care are major factors for increasing the RIOT rate and therefore survival.

## Surgery for Colorectal Peritoneal Carcinomatosis in no-expert Centers is associated with Tumor Persistence or Early Recurrence in Most Cases: A Bicentric Study of 106 Patients

B. Noiret ^1^, V. Kepenekian ^2^, M. Provost ^3^, G. Piessen ^1^, O. Glehen ^3^, C. Eveno ^1^

^1^Department of Digestive and Oncological Surgery, Claude Huriez University Hospital - Lille (France),

^2^Surgical Oncology Department, Hôpital Lyon Sud, Hospices Civils de Lyon, - Lyon (France),

^3^Surgical Oncology Department, Hôpital Lyon Sud, Hospices Civils de Lyon - Lyon (France)

**Background:** The addition of oxaliplatin-based HIPEC to cytoreductive surgery (CRS) for colorectal peritoneal metastases (CRPM) did not improve overall survival (OS) according to the PRODIGE 7 trial, raising concerns about a reduction in patient referral to expert centers favoring CRS alone in non-expert centers. The aim of our study was to evaluate the strategy of systematic second-look surgery in expert centers after CRS for CRPM performed in non-expert centers at a French national level.

**Patients and methods:** Data of CRPM patients treated with CRS alone between 2010-2022 in France were collected through databases of two HIPEC centers. Patients with colorectal cancer and histological evidence of peritoneal metastases (PM) after initial surgery in no-expert centers and then referred to the two expert HIPEC centers for CRS±HIPEC were included. Perioperative outcomes were evaluated in both surgeries. Survival outcomes were performed by Kaplan-Meier method.

**Results:** Among 106 patients (50.9% male, mean age 58.9±10.8 years), all tumors were classified as pT3-T4, with 87% having synchronous CRPM. Initial CRS in no-expert centers was performed urgently in 35% of cases, mainly due to occlusion or perforated tumor (62.1%). Intra-operative CRPM was discovered in 86% of cases with CRS described as complete in 63% of cases. CRPM recurrence was observed at reassessment of CT-scan and MRI in 39% and 48% of cases and in 88% during the second-look laparotomy at expert centers. The PCI was 6, with ≥3 organs resected in 33% of cases; and 10% of patients were deemed unresectable. 5-year OS and peritoneal-free survival was 40% and 35%, respectively.

**Conclusion:** CRS performed in no-expert centers, does not provide optimal treatment for CRPM, resulting in a residual disease or early recurrence rate of 88%. Referral to expert centers should be ensured from the diagnosis of CRPM to prevent worsening oncological outcomes.

## PSOGI 2024: 204 abstracts

- Artificial Intelligence and big data approaches in peritoneal surface malignancies (n=3)
- Basic science related to peritoneal surface malignancies (n=17)
- Diagnosis and staging of peritoneal surface malignancies (n=7)
- Educational on peritoneal surface malignancies (n=3)
- Intraperitoneal treatment for peritoneal surface malignancies (n=53)
- Open proposals linked to peritoneal surface maligancies management (n=11)
- Patient-centered care in peritoneal surface malignancies (n=15)
- Perioperative care (n=15)
- Peritoneal mesothelioma (n=8)
- Peritoneal metastases from colorectal cancer (n=41)
- Peritoneal metastases from gastric cancer (n=13)
- Peritoneal metastases of ovarian cancer (n=9)
- Pseudomyxoma peritonei (n=9)

## Artificial Intelligence and big data approaches in peritoneal surface malignancies

### PO 01 Deep learning algorythm for peritoneal carcinomatosis detection. Performance through analysis of laparoscopic video recording. #PO 01

#### Oral communication

F. Dumont^1^, A.B. Rahardjo^2^, C. Dumas^3^, T. Vignaud^4^, E. Tibaudeau^4^

^1^institut de cancerologie de l’ouest - Saint Herblain (France),

^2^intitut teknologo Sepuluh Nopember - Surabaya (Indonesia),

^3^insitut Mines Telecom Atlantiques - Nantes (France),

^4^Insittu de cancérologie de l’Ouest - Saint-Herblain (France)

**Abstract**

**Purpose:** Achieving competency in nodule detection of peritoneal metastasis (PM) is an essential component of PM management. Human assistance by deep learning system (DLS) can improve detection of malignant of tumor nodules. The aim of this study was to assess performance of preliminary DLS for tumor nodules detection in peritoneum.

**Methods:** Two surgeons specialized in peritoneal carcinomatosis, contoured via a specific computer program, all tumor nodules considered malignant on 5 videolaparoscopy of PM from colorectal cancer. The median PCI was 14 (2-20). DLS was developed from 1399 images of laparoscopy video of PM and used YOLO-based models. An augmentation process is carried out using Roboflow, allowing a dataset of 4197 images.

Performance of DLS was tested on laparoscopy video and compared to annotations of experts surgeons. Among 605 peritoneal tumors labeled by DLS and according the threshold of detection, the number of true positive, false positive and false negative ranged from 545 to 577, 26 to 62 and 28 to 68. The sensitivity ranged from 90 to 95% and the positive predictive value ranged from 90 to 95%. New reading of the five laparoscopy by one experimented surgeon was performed with help of dynamic algorythm detection in order to detect all malignant tumor. The result showed that 5.1% of tumor nodules were not seen by surgeon before being alerted by DLS.

**Conclusions:** First version of DLS show excellent sensitivity of nodules of PM and seems useful for human assistance of tumor nodule detection.

Laparoscic PM detected by artificial intelligency

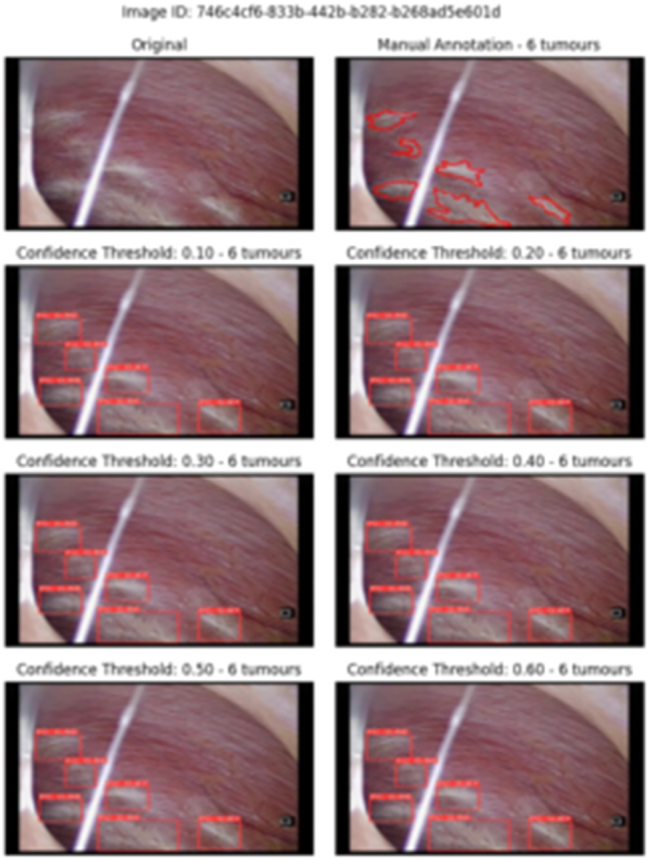



### PO 02 Employing Integrative Analysis to Identify Novel Targets in Ascitic Fluid of Peritoneal Metastasis: Building upon Paracrine Analysis Methodologies #PO 02

#### Oral communication or poster

A.J.Y. Ang^1^, Y. Liu^2^, Q.X. Tan^2^, J.W.S. Tan^2^, G. Ng^2^, C.Y.L. Chong^2^, W.Y. Guo^2^, J.S.M. Wong^3^, C.S. Chia^3^, C.A.J. Ong^3^

^1^Duke-NUS Medical School - Singapore (Singapore),

^2^Laboratory of Applied Human Genetics, Division of Medical Sciences, National Cancer Centre Singapore - Singapore (Singapore),

^3^Department of Sarcoma, Peritoneal and Rare Tumours, Division of Surgery and Surgical Oncology, National Cancer Centre Singapore - Singapore (Singapore)

**Abstract**

**Background:** Peritoneal metastases (PM) represent an advanced stage of cancer associated with limited treatment options and poor prognosis. Ascitic fluid accumulation, an oncogenic driver of PM, exacerbates patient symptoms and contributes to disease progression. Previous literature has identified certain paracrine factors present in ascitic fluid that drive this progression, however, these factors remain generally poorly characterized. A comprehensive understanding of these factors is crucial for identifying potential therapeutic targets and prognostic markers. We aim to investigate the significance of paracrine factors in ascitic fluid driving PM progression, which has the potential to inform the development of targeted therapies and personalized treatment approaches and improve patient outcomes and quality of life.

**Methods:** Through a review of the literature, multiple drivers of paracrine factors were found, and one starting gene was identified for the analysis. Following which, programmatic access of genomic pathway databases was undertaken to find all genes that were related to the starting gene. Proteomic and protein-sequencing databases were cross-referenced to derive a list of proteins that are secreted and enriched in ascitic fluid and peritoneal fluid. To identify prognostically significant factors, only proteins that had existing inhibitors and were prognostic in at least three different cancer subtypes were considered. The results were compared to our mass spectroscopy data to identify target therapeutic genes.

**Results:** We identified TNF-alpha as a prominent gene in the tumor-microenvironment of ovarian cancer. Through programmatic analysis, eight genes related to TNF-alpha were prognostic in at least three different primary cancer subtypes. Three of these genes (F2, MMP2, PLAT) were significant when cross-referenced to mass spectroscopy data from our centre.

**Conclusions:** Integrative analysis of genomic, proteomic, and transcriptomic data identified multiple paracrine factors that can improve the understanding of PM pathogenesis. In vivo, in vitro, and biochemical investigations are ongoing to validate these as therapeutic targets.

### PO 03 Initial Experience Mini-Invasive Robotic Cytoreduction and HIPEC for Peritoneal Carcinomatosis: A Promising Approach #PO 03

#### Oral communication

A. Bertolucci^1^, L. Piccini^1^, E. Rreka^1^, A. Greco^1^, M. Ferrari^1^, B. Musco^1^, P.V. Lippolis^1^

^1^Azienda Ospedaliera Universitaria Pisana - PISA (Italy)

**Abstract**

Peritoneal carcinomatosis represents a challenging condition, often necessitating comprehensive surgical interventions such as cytoreduction and hyperthermic intraperitoneal chemotherapy (HIPEC). Herein, we present the initial experience of cases treated with robotic cytoreduction and HIPEC. The patients demonstrated excellent postoperative outcomes, with an average hospital stay of 7 days and no postoperative complications. Notably, the Enhanced Recovery After Surgery (ERAS) protocol, seldom applied in traditional surgery for this condition, was fully implemented. Oncologically, the interventions achieved radical results, underscoring the potential of this approach in managing peritoneal carcinomatosis.

## Basic science related to peritoneal surface malignancies

### PO 04 Biomarkers of mesothelial cell EMT, carcinogenesis and mesothelioma invasiveness in experimental rat models #PO 04

#### Poster

D.L. Pouliquen^1^, A. Boissard^2^, C. Henry^2^, V. Verrièle^3^, M. Le Gall^4^, F. Guillonneau^5^

^1^Inserm U1307, CRCI2NA - Angers (France),

^2^Prot’ICO Facility, ICO - Angers (France),

^3^Dept Ana-Path, ICO, Inserm U1307, CRCI2NA - Angers (France),

^4^Protéom’IC Facility, Institut Cochin - Paris (France),

^5^Prot’ICO Facility, ICO, Inserm U1307, CRCI2NA - Angers (France)

**Abstract**

A collection of 26 experimental mesothelial cell lines established in immunocompetent rats after intraperitoneal induction with asbestos fibers was used to hierarchize candidate protein biomarkers involved in mesothelioma tumorigenesis and / or the epithelial-to-mesenchymal transition (EMT). Proteomic analysis aimed to discriminate between 22 preneoplastic cell lines showing epithelioid (G1 group, n = 10) or sarcomatoid morphology (G2 group, n =12), and 4 neoplastic cell lines (G3 group). 200 nanograms of peptides from each cell line lysate were analyzed by LC-MS/MS with a nanoHPLC hyphenated with a TIMS-TOF *Pro2* Bruker mass spectrometer. The comparison [G3 vs (G1 + G2)] identified 1299 differentially expressed proteins (*p*-values < 10^-4^) involved in the tumorigenic process, of which 36 exhibited additional differential abundances related to mesothelioma invasiveness. Among these 36 proteins, 23 were also involved in EMT (significant abundance changes in (G2 vs G1)). Dramatic decrease was observed for proteins encoded by *Gnai2* (Gα protein family involved in cell signaling transduction), *Swi5* (DNA homologous recombination), *Abraxas2* (apoptosis regulation), *Grn* / *Kxd1* (lysosome functions), and *Rpl9* / *Rps27a* (ribosomes). The neoplastic transformation was also characterized by increased abundance of proteins encoded by *Pdcd10* (apoptosis regulation), *Eif3f* (translation regulation), *Dnajc9* (DNA replication), *Mrto4* (mRNA metabolism), and *Cbr1* (carbonyl reductase). On the other hand, within the 13 remaining proteins of interest not additionally involved in EMT, 6 exhibited increased abondance related to tumorigenesis and invasiveness, encoded by *Lgals3* / *Cd47*, *Trip13*, *Fhl3*, *Ppip5k1* and *Tmx4*. In parallel, an Ingenuity Pathway Analysis of the most differentially expressed proteins in non-malignant transformations of peritoneal mesothelial cells, involving EMT in (G2 vs G1), identified candidate biomarkers encoded by *Cnn1*, *Csrp2*, *Loxl2*, *Tagln* (increase); and *Krt7*, *Tspan8* (decrease). All these data represent a valuable ground for identifying potential common markers of peritoneal surface malignancies found in patients.

### PO 05 Theoretical basis of comprehensive treatment for patients with peritoneal metastasis #PO 05

#### Oral communication

Y.Y.Y.Y. Yonemura^1^

^1^Kishiwada Tokusyukai Hopital - Kishiwada (Japan)

**Abstract**

(COMPT) designed to cure colorectal, and gastric cancer cancer patients with peritoneal metastasis (PM).

There are four curative scenarios following COMPT. Scenario A involves cases without micrometastasis (MM), where patients can potentially be cured by complete cytoreductive surgery (CCRS) alone. Similarly, if the residual number of MM is below the threshold level that can be completely eliminated by intraoperative hyperthermic intraperitoneal chemoperfusion (IOHIPEC), patients will be cured by CCRS plus IOHIPEC (Scenario C).

If neoadjuvant chemotherapy (NAC) reduces the MM burden below the threshold level, patients may then be cured by CCRS combined with IOHIPEC (Scenario D). If NAC completely eliminates MM, patients will then be cured by CCRS alone (Scenario F). Cure is defined as survival without recurrence for longer than 5 years after following COMPT.

Among 278 colorectal cancer (CRC)-patients with PM who underwent CCRS, the number of patients achieving a cure was 31 (11%), and the PCI was ≤ 12.

Among 10 patients treated with CCRS alone, and one patient with peritoneal cancer index (PCI) of 4 was cured (Scenario A). Four (29%) of 14 patients treated with CCRS plus IOHIPEC were cured. Four (7%) of 60 patients treated with NAC plus CCRS, were cured (Scenario F). Twenty-two (11%) of 195 patients treated with CCRS plus IOHIPEC after NAC were cured (Scenario C or D).

Among 278 gastric cancer (GC) patients with PM, no patient belongs to S-A, and 8 (8%) of 107 patients of S-F. Three (21%) of 36, and 14 (9%) of 156 patients in S-C and S-D are cured.

**Conclusions:** CRC and GC-PM-patients with a PCI ≤ 12 and PCI ≤ 10 could be cured with CCRS plus perioperative chemotherapy in 31 (11%, 31/278) and 25 (9%, 25/261).

### PO 06 Does the HyaRegen® gel used after PIPAC procedures have an effect on the progression of colorectal peritoneal metastases? #PO 06

#### Poster

M.L. Perrin^1^, S. Durand Fontanier^2^, C. Bassetti^1^, C. Yardin^1^, S. Bardet^1^, A. Taibi^3^

^1^Limoges University, CNRS research team, UMR 7252, groupe Biosanté, 87000, limoges, France - Limoges (France),

^2^Digestive Surgery Department, chu de limoges, Limoges University, CNRS research team, UMR 7252, groupe Biosanté, 87000, limoges, France - Limoges (France),

^3^Digestive surgery department, CHU de Limoges, CNRS research team, UMR 7252, groupe Biosanté, 87000, limoges, France - Limoges (France)

**Abstract**

The aim of this study was to evaluate the effects of the hyaluronic acid-based gel on tumor dissemination. First, we explored whether the survival of CT26 luciferase-expressing murine colonic tumor cells was correlated with the dose of HyaRegen® Gel, and we determined the half-maximal inhibitory concentration (the IC50) of the gel. Next, we performed an in vitro study of cell survival rates after gel application on day 0 and day 1. Finally, we intraperitoneally administered the gel to mice with immunocompetent BALB/c colonic peritoneal metastases. Tumor growth was regularly monitored using a bioluminescence assay. After all mice had been sacrificed on D21, the body weights and the volumes of intraperitoneal ascites were measured; the Peritoneal Carcinosis Index (PCI) and Ki-antigen 67 scores were calculated.The IC50 value was 70 µL of gel in a total volume of 100 µL. The cell survival rates on D4 were identical in the control group and the two groups that had been treated with gel. The bioluminescence levels over time were similar in the gel and control groups. The PCI scores were 35.5 ± 2.89 for the control group and 36 ± 2.45 for the gel group (p = 0.8005). The mean Ki-67 index percentages were 37.28 and 34.03 (p = 0.1971).This in vitro and in vivo study using a mouse model of immunocompetent metastatic peritoneal cancer did not reveal any pro- or anti-tumoral effect of HyaRegen® Gel. These findings indicate that the gel can be used to treat PIPACs with minimal apprehension.

In vivo analysis of HyaRegen® Gel effects

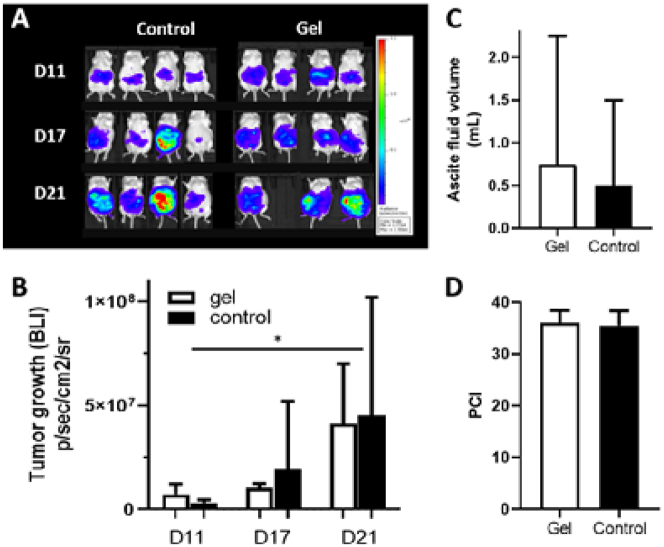



### PO 07 Is pressurized intraperitoneal aerosol chemotherapy (PIPAC) feasible in a rabbit model of gastric peritoneal metastases? #PO 07

#### Poster

S.M. Bardet^1^, M.L. Perrin^1^, C. Yardin^1^, S. Durand Fontanier^2^, A. Taibi^3^

^1^Limoges university, CNRS research team, UMR 7252, groupe BIOSANTE, 87000 LIMOGES - LIMOGES (France),

^2^Digestive surgery département, CHU de Limoges, Limoges university, CNRS research team, UMR 7252, groupe BIOSANTE, 87000 LIMOGES - LIMOGES (France),

^3^Digestive surgery département, CHU de Limoges, CNRS research team, UMR 7252, groupe BIOSANTE, 87000 LIMOGES - LIMOGES (France)

**Abstract**

The primary objective was to assess the safety and viability of repetitive PIPAC procedures in a rabbit model exhibiting gastric peritoneal metastases. The rabbits, as per the model by Pascal et al. (2017), were subjected to PIPAC treatments with serum physiological solutions on Days 8, 15, and 21. Preceding euthanasia on Day 26, a PIPAC procedure incorporating trypan-blue-dye was conducted.

Evaluation of the well-being status was carried out before each PIPAC session. Abdominal CT scans were performed prior to the first PIPAC and post the third PIPAC. During each PIPAC, ascites volume and Peritoneal Cancer Index (PCI) were quantified, alongside biopsy sampling from at least three nodules. Morbidity and mortality outcomes were documented. Histological scrutinization employing HES and Microscopic Multiphoton Imaging was undertaken.

The well-being score of all three rabbits decreased to below 2 points after the series of PIPAC interventions. A wound in the intestine was sutured without consequence, and no rabbit died during the experiment. Histological analysis revealed an increased presence of collagen in the peritoneum following PIPAC compared to control rabbits; however, no heightened tissue inflammation was observed in either the peritoneum or liver parenchyma. The distribution pattern of the blue dye post-PIPAC application displayed homogeneity across the parietal peritoneum, small intestine peritoneum, and colon while showing weaker diffusion in the diaphragmatic-cupola and Douglas’ cul-de-sac.

In conclusion, our findings indicate that PIPAC is both feasible and safe within a rabbit model featuring gastric peritoneal metastases. This model holds promise for evaluating novel chemotherapy protocols involving PIPAC applications.

Experimental protocol

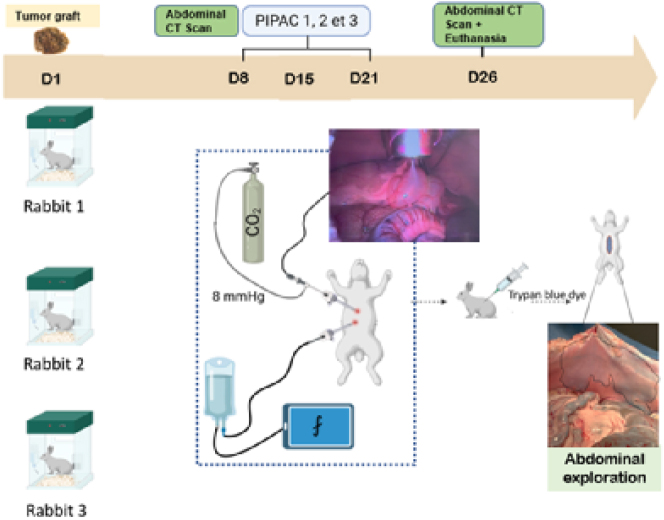



### PO 08 Preclinical evaluation of operational stability and drug delivery in a new device for hyperthermic intraperitoneal chemotherapy with negative pressure infusion capability #PO 08

#### Poster

S. Lee^1^, M.S. Kiyomiddinovna^2^, S.H. Oh^3^, S.H. Shim^4^, S.H. Lee^5^, J.W. Park^6^, S.J. Chang^7^, H.S. Kim^8^

^1^Keimyung University School of Medicine - Daegu (Korea, Republic of),

^2^Tashkent Medical Academy - Tashkent (Uzbekistan),

^3^Gacheon University College of Medicine - Incheon (Korea, Republic of),

^4^Konkuk University School of Medicine - Seoul (Korea, Republic of),

^5^Yonsei University Wonju College of Medicine - Wonju (Korea, Republic of),

^6^Seoul National University Hospital - Seoul (Korea, Republic of),

^7^Ajou University School of Medicine - Suwon (Korea, Republic of),

^8^Seoul National University College of Medicine - Seoul (Korea, Republic of)

**Abstract**

**Background:** We developed a new device for hyperthermic intraperitoneal chemotherapy (HIPEC) with negative pressure infusion, and evaluated its operational stability and drug delivery in pigs.

**Methods:** We performed HIPEC with a flow rate of 1,200 ml/min and a negative pressure of -20 mmHg in three pigs. First, we evaluated the induction time from the room temperature (22°C) to the body temperature (36°C) in the circulating solution, and then the target time from the body temperature (36°C) to the therapeutic temperature (42°C for inlets; 40°C for outlets). Second, we evaluated the temperature, flow rate and required negative pressure for the 90-min maintenance period. Finally, we evaluated the drug distribution and penetration depth in eleven abdominal regions by using 1% methylene blue.

**Results:** The induction time was 15.5 min, and the target times were 16.3 to 21.5 min for inlets and 20.3 to 25.8 min for outlets, showing no difference of the flow rate and a decreased in required negative pressure (Figure 1). Thereafter, there were no differences in time-dependent temperature, flow rates and required negative pressures for the maintenance period. Moreover, most of the peritoneum was intensely stained, and the penetration depth did not differ among the 11 regions (P=0.073).

**Conclusion:** The new device with negative pressure infusion capability may take about 30 to 35 min before implementation of HIPEC. Furthermore, there may be no differences in time-dependent temperature, flow rate and required negative pressure for the maintenance period, which may drive even drug delivery and penetration into the abdominal cavity.

Induction and target times for HIPEC

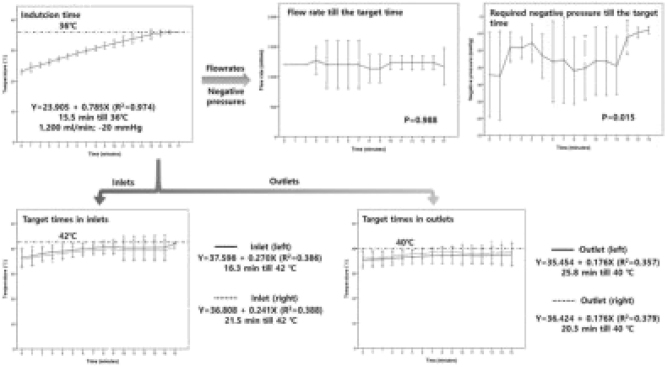



### PO 09 Preclinical evaluation of the pharmacokinetics, drug exposure and toxicity in rotational intraperitoneal pressurized aerosol chemotherapy using gemcitabine #PO 09

#### Poster

M.S. Kiyomiddinovna^1^, H.S. Kim^2^, S.H. Oh^3^, S. Lee^4^, G.W. Yim^5^, S.H. Shim^6^, J.W. Park^7^, S.J. Chang^8^, S.H. Lee^9^

^1^Tashkent Medical Academy - Tashkent (Uzbekistan),

^2^Seoul National University College of Medicine - Seoul (Korea, Republic of),

^3^Gacheon University College of Medicine - Incheon (Korea, Republic of),

^4^Keimyung University School of Medicine - Daegu (Korea, Republic of),

^5^Dongguk University College of Medicine - Goyang (Korea, Republic of),

^6^Konkuk University School of Medicine - Seoul (Korea, Republic of),

^7^Seoul National University Hospital - Seoul (Korea, Republic of),

^8^Ajou University School of Medicine - Suwon (Korea, Republic of),

^9^Yonsei University Wonju College of Medicine - Wonju (Korea, Republic of)

**Abstract**

**Background:** We evaluated the pharmacokinetics (PK), drug exposure, and toxicity of rotational intraperitoneal pressurized aerosol chemotherapy (RIPAC) using gemcitabine in pigs.

**Methods:** We sprayed gemcitabine of 10% and 30% of doses for intravenous chemotherapy (IC) in six pigs (cohort 1, n=3, 300 mg/m2; cohort 2, n=3, 1,000 mg/m2). We determined the safe dose of gemcitabine by comparing tissue concentrations, and hepatic and renal functions in two cohorts, and then compared the time-dependent plasma concentrations for the pharmacokinetics among two cohorts and IC (n=3) using the safe dose.

**Results:** Mean values of tissue concentrations were 1.3 to 11.2 times higher in cohort 2 than in cohort 1, with the similar findings that tissue concentrations were higher in the parietal peritoneum than in the visceral peritoneum. Moreover, we determined gemcitabine of 300 mg/m2 as the safe dose because cohort 2 showed a change of hepatic function after RIPAC. Cohort 2 showed the highest values of peak plasma concentration (Cmax), the time to Cmax (Tmax), the time taken for Cmax to drop in half (T1/2), the area under the curve from time zero to the time of last quantifiable concentration (AUClast), and AUC from zero to infinity (AUCinf) despite no difference in AUClast, AUCinf, and T1/2 between cohort 1 and IC using the safe dose (Table 1).

**Conclusion:** This preclinical study may suggest that gemcitabine of 300 mg/m2 can be considered as the starting doses for RIPAC in a phase I trial.

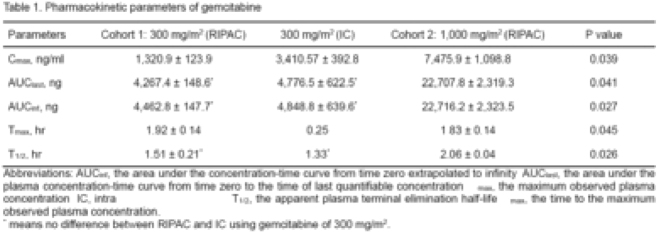



### PO 10 Preclinical evaluation of the pharmacokinetics, drug exposure and toxicity in rotational intraperitoneal pressurized aerosol chemotherapy using pemetrexed #PO 10

#### Poster

M.S. Kiyomiddinovna^1^, S.H. Oh^2^, S. Lee^3^, G.W. Yim^4^, S.H. Shim^5^, J.W. Park^6^, S.H. Lee^7^, S.J. Chang^8^, H.S. Kim^9^

^1^Tashkent Medical Academy - Tashkent (Uzbekistan),

^2^Gacheon University College of Medicine - Incheon (Korea, Republic of),

^3^Keimyung University School of Medicine - Daegu (Korea, Republic of),

^4^Dongguk University College of Medicine - Goyang (Korea, Republic of),

^5^Konkuk University School of Medicine - Seoul (Korea, Republic of),

^6^Seoul National University Hospital - Seoul (Korea, Republic of),

^7^Yonsei University Wonju College of Medicine - Wonju (Korea, Republic of),

^8^Ajou University School of Medicine, - Suwon (Korea, Republic of),

^9^Seoul National University College of Medicine - Seoul (Korea, Republic of)

**Abstract**

**Background:** We evaluated the pharmacokinetics (PK), drug exposure, and toxicity of rotational intraperitoneal pressurized aerosol chemotherapy (RIPAC) using pemetrexed in pigs.

**Methods:** We sprayed gemcitabine of 10% and 30% of doses for intravenous chemotherapy (IC) in six pigs (cohort 1, n=3, 90 mg/m2; cohort 2, n=3, 270 mg/m2). We determined the safe dose of gemcitabine by comparing tissue concentrations, and hepatic and renal functions in two cohorts, and then compared the time-dependent plasma concentrations for the pharmacokinetics among two cohorts and IC (n=3) using the safe dose.

**Results:** Mean values of tissue concentrations were 0.9 to 11.5 times higher in cohort 2 than in cohort 1, with the similar findings that tissue concentrations were higher in the parietal peritoneum than in the visceral peritoneum. Moreover, we determined pemetrexed of 270 mg/m2 as the safe dose because of no differences in hepatic and renal functions between the two cohorts. IC of 270 mg/m2 showed the highest values of peak plasma concentration (Cmax), the area under the curve from time zero to the time of last quantifiable concentration (AUClast), and AUC from zero to infinity (AUCinf), and the lowest value of the time to Cmax (Tmax) with no difference in the time taken for Cmax to drop in half (T1/2) when compared to the two cohorts (Table 1).

**Conclusion:** This preclinical study may suggest that pemetrexed of 270 mg/m2 can be considered as the starting doses for RIPAC in a phase I trial.

**Table 1.**
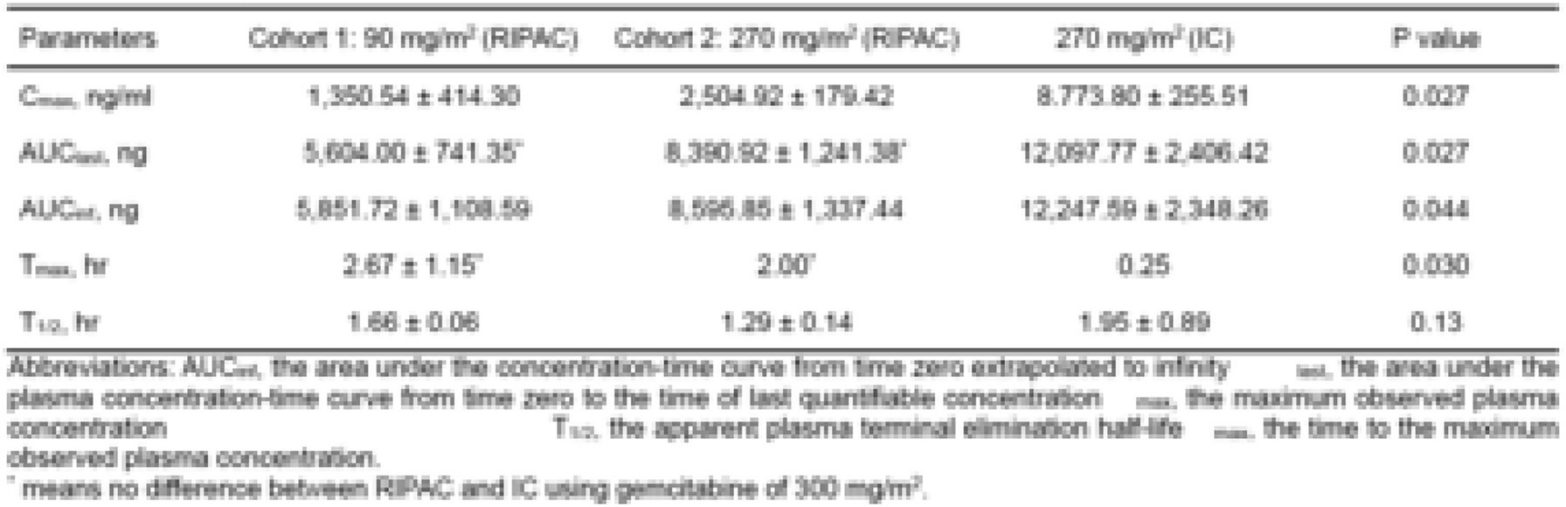
Pharmacokinetic parameters of pemetrexed

### PO 11 How HIPEC plays a role along with CRS in all settings of advanced Epithelial Ovarian Cancer: A pilot study on genetic point of view. #PO 11

#### Oral communication or poster

M.D. Ray^1^, A. Sharma^1^

^1^All India Institute of Medical Sciences - DELHI (India)

**Abstract**

**Background:** Regulatory T-cells (Tregs) play a crucial immunosuppressive role in ovarian cancer thereby causing progression and recurrences. On the other hand, Th17 cells help to activate B-cells, cytotoxic T-cells to kill the tumour cells. However, changes in Tregs population in patients with ovarian cancer after HIPEC in not yet known. HIPEC might have a definite role in ovarian tumour microenvironment.

**Methodology:** Immunological imbalance between Tregs and Th17 cells leads to cancer suppression. Our study aims to observe the variable expression pattern of Tregs and cytokine profile (CD4+, CD25+, CD127-, IL10+, Foxp3+) and Th17 cells (CD4+, CCR6+, IL17+, RORγ) in peripheral blood by flow cytometry and RT-PCR before and after the HIPEC (in 3 settings) in 1st and 4th week.

**Results:** Percentage of Tregs in Ovarian Cancer patients (n=30) were high compared to healthy controls before HIPEC and reduced remarkably 4 weeks after CRS and HIPEC. Th17 cells frequency was low before HIPEC but in patients after HIPEC, Th17 cells are increased after 4 weeks. There was highest expression of FOXp3 mRNA in recurrent group followed by upfront and interval. There was statistically significant decrease in FOXp3 at four weeks in three subgroups. There was an increase in ROR-ɣ and interleukin-17 in 4 weeks in upfront and interval groups

**Conclusion:** This basic research, exploring the role of FOXp3 Tregs and RORγ T-helper cells, indicated a potential role of biomarkers in understanding the prognosis of EOC patients and the impact of therapeutic interventions, like HIPEC, on outcomes.

Graphcal presentation of various expressions of FO

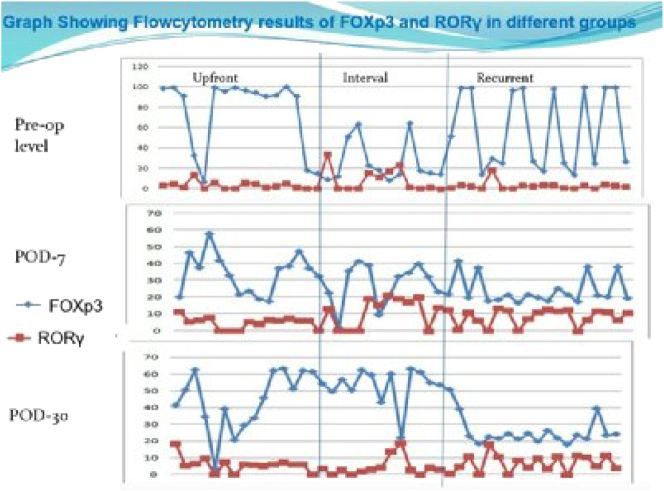



### PO 12 Expression profiling of 800 mRNAs in peritoneal metastasis from pancreatic cancer treated with systemic chemotherapy and PIPAC #PO 12

#### Oral communication

S. Detlefsen^1^, M. Burton^1^, A. Ainsworth^1^, C. Fristrup^1^, M. Graversen^1^, P. Pfeiffer^1^, L. Tarpgaard^1^, M. Mortensen^1^

^1^Odense University Hospital - Odense (Denmark)

**Abstract**

**Background:** Pressurized Intraperitoneal Aerosol Chemotherapy (PIPAC) is an experimental treatment in peritoneal metastasis from pancreatic cancer (PM-PC). Peritoneal quadrant biopsies (QBs) taken prior to each treatment are used for histological response evaluation using the Peritoneal Regression Grading Score (PRGS). Aim was to examine the mRNA profile of fibrosis due to response after systemic chemotherapy and PIPAC (Regression) compared to treatment-naïve PM-PC and chronic cholecystitis-related peritoneal fibrosis (Controls).

**Methods:** RNA was extracted from formalin-fixed and paraffin embedded (FFPE) QBs from PM-PC patients who had undergone systemic chemotherapy and PIPAC, showing histological response (PRGS 1, n=11), from peritoneal biopsies with treatment-naïve PM-PC (n=10), and Controls (n=10). Profiling of 800 mRNAs was performed (NanoString nCounter, IO360).

**Results:** Principal component analysis (PCA) and a heat map (with unsupervised hierarchical clustering) showed separation between PM-PC, Regression and Controls. In PCA, a few Regression cases clustered with Controls (Figure 1A&1B). Regression vs. PM-PC identified six upregulated and 197 downregulated mRNAs (FDR≤0.05), linked to TNFα signaling via NF-kB, G2M checkpoint, epithelial-mesenchymal transition, estrogen response, and coagulation (Figure 1C&1D). Regression vs. Controls identified 43 significantly upregulated mRNAs, linked to interferon-α response, and downregulation of 99 mRNAs, linked to TNFα signaling via NF-kB, inflammatory response, epithelial-mesenchymal transition, *KRAS* signaling, and hypoxia (FDR≤0.05) (Figure 1C&1D).

**Conclusions:** Dysregulation of mRNAs related to key tumor biological pathways was identified in Regression of PM-PC after systemic chemotherapy and PIPAC. Regression also showed significant transcriptomic differences from Controls. Future studies should explore whether mRNA profiling of QBs with PM holds prognostic or predictive value.

Unsuperv. clustering & differential RNA expression

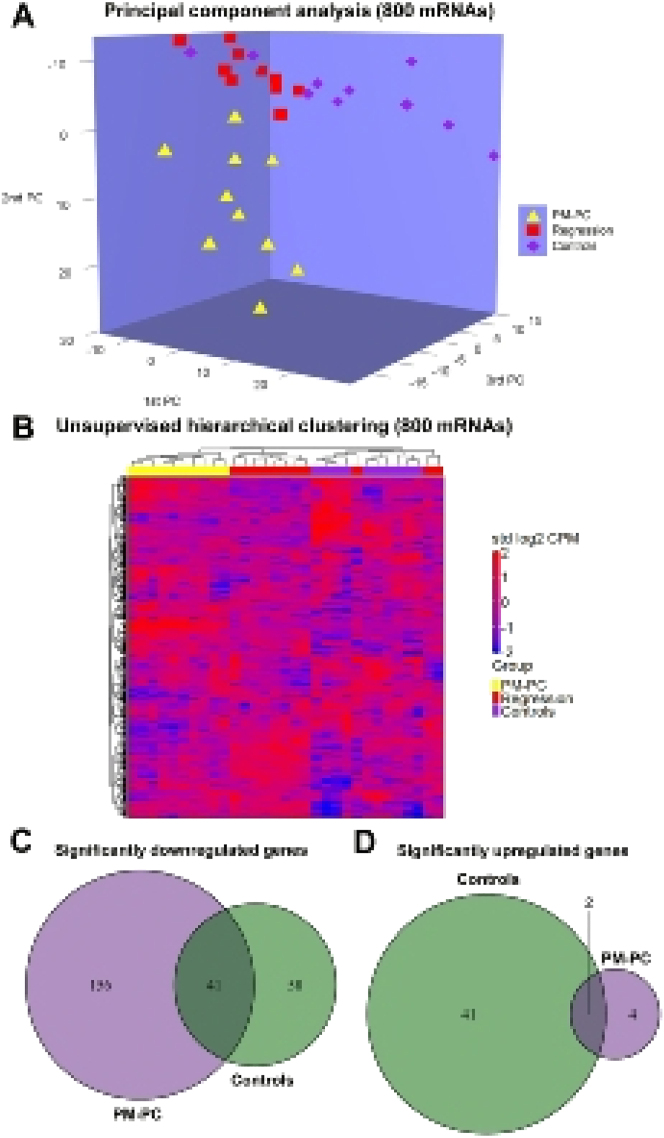



### PO 13 Interest of extracellular vesicles associated with PIPAC in a murine model of peritoneal carcinomatosis of colonic origin #PO 13

#### Oral communication or poster

P. Rozenbaum^1^, M. Thaury^1^, J. Branchu^2^, J. Volatron^2^, C. Crocheray^1^, F. Gazeau^3^, M. Pocard^1^

^1^INSERM U1275 - Paris (France),

^2^Everzom - Paris (France),

^3^CNRS UMR7057 - Paris (France)

**Abstract**

**Background:** Pressurized Intra Peritoneal Aerosol Chemotherapy (PIPAC) is an emerging technique that can be proposed to patients with peritoneal carcinomatosis. The development of PIPAC has led to the creation of new innovations, including the creation of drug carriers through bioengineering, which allow for the modification of chemotherapy (CT) capabilities with the aim of better penetration into tumor nodules and improved anti-tumor selectivity.

**Methods:** We worked on extracellular vesicles (EVs) in vitro and in a murine model of peritoneal carcinomatosis from colonic origin. First experiment studied the stability of EVs before and after aerosolization by PIPAC under real conditions, we used Nanoparticle Tracking Analysis to asses size and concentration. Second experiment studied the cytotoxicity, on CT26 cancerous cells, of an aerosol containing Mitomycin with and without EVs, we used AlamarBlue technique to asses cell viability at day 2 of treatment. Last experiment studied the efficacy, on carcinosis induced mices grafted at day 0 with CT26 cancerous cells, of an intra-peritoneal injection at day 6 and 8 of a Mitomycin solution with and without EVs, we used Peritoneal Cancer Index for assesment at day 10.

**Results:** First experiment showed no differences between the two groups in terms of concentration with p = 0,1231 and a slight increase of 7% in size between the two groups with a p = 0,0204. Second experiment showed a reduction of 11.2% in cell viability between the two groups with p < 0.0001. Last experiment showed a reduction of 73% in mean PCI between the two groups with p = 0.0237.

**Conclusions:** These results reinforce our intention to propose a phase I study to confirm the efficacy of the chemotherapy and EVs combination delivered by PIPAC in humans with peritoneal carcinomatosis from colonic origin.

### PO 180 Oxaliplatin penetration of peritoneum comparing Intravenous administration and intraperitoneal spraying method via a PIPAC and HPIPAC(hyperthermic PIPAC) #PO 14

#### Oral communication or poster

D.K. Lee^1^, H.H. Kim^2^, Y. Jung^1^, S.K. Bae^3^, M. Yoo^4^, C. Bang^5^, S. Lee^6^, C. Yoon^6^, D.Y. Kim^3^, J. Park^6^

^1^College of Pharmacy, Chung-Ang University - Seoul (Korea, Republic of),

^2^Chung-Ang University Gwangmyeong Hospital - Gwangmyeong (Korea, Republic of),

^3^College of Pharmacy, The Catholic University - Bucheon (Korea, Republic of),

^4^Asan medical center - Seoul (Korea, Republic of),

^5^Seoul National University Bungang hospital - Seongnam (Korea, Republic of),

^6^Graduate School of Public Health, Seoul National University - Seoul (Korea, Republic of)

**Abstract**

**Background:** We have deveolped a novel intraperitoneal drug delivery system, HPIPAC (Hyperthermic Pressurized Intraperitoneal Aerosol Chemotherapy), which combines the gas-based HIPEC (Hyperthermic Intraperitoneal Chemotherapy) and PIPAC (Pressurized Intraperitoneal Aerosol Chemotherapy) methods. In this study, we meticulously evaluated the penetration of oxaliplatin into the peritoneum using our HPIPAC system, comparing it with the systemic route and the PIPAC method in a swine model.

**Methods:** We administered 5 mg/kg of oxaliplatin to a pig weighing approximately 25 kg for each group. The blood and tissue samples were taken 20 minutes after completing each procedure. The degree of peritoneal invasion by oxaliplatin was examined using matrix-assisted laser desorption/ionization (MALDI) imaging. Ice-cold ethanol-based metabolism quenching halted metabolism without loss of oxaliplatin, and the distribution based on the signal of oxaliplatin’s biotransformed form, Pt(diaminocyclohexane)(Histidine), was observed through the application of a 2,5-dihydroxybenzoic acid as a matrix.

**Results:** The depth of peritoneum penetration was calculated based on the ratio of the area where the oxaliplatin signal was detected to the total area of the peritoneum (%). HPIPAC (54.53%) demonstrated higher penetration compared to PIPAC (41.37%) and IV (7.50%) methods.

**Conclusions:** Our HPIPAC method, which circulates heated carbon dioxide extracorporeally to maintain hyperthermia, necessitates the simultaneous extracorporeal circulation of intraperitoneal drugs. However, despite the lower drug exposure concentration, the HPIPAC method remarkably increased oxaliplatin delivery efficiency by approximately 13% compared to PIPAC.

oxaliplatin penetration of peritoneum

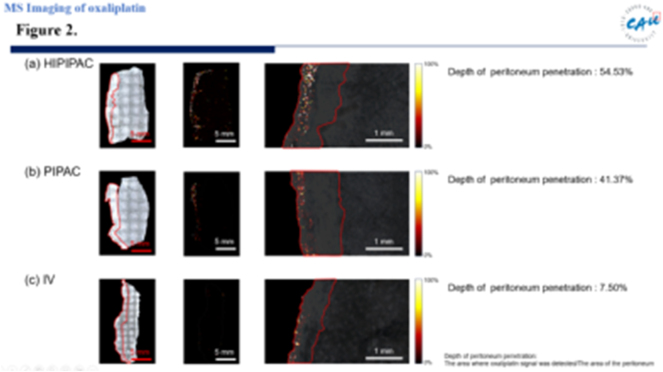



### PO 15 Oxaliplatin concentration in blood and tissues between systemic administration and intraperitoneal spraying methods including PIPAC and HPIPAC( hyperthermic PIPAC) #PO 15

#### Oral communication or poster

D.Y. Kim^1^, H.H. Kim^2^, S.K. Bae^1^, M. Yoo^3^, D.K. Lee^4^, C. Yoon^5^, S. Lee^5^, C. Bang^6^, Y. Jung^4^, S. Hwang^5^

^1^College of Pharmacy, The Catholic University - Bucheon (Korea, Republic of),

^2^Chung-Ang University Gwangmyeong Hospital - Gwangmyeong (Korea, Republic of),

^3^Asan Medical center - Seoul (Korea, Republic of),

^4^College of Pharmacy, Chung-Ang University - Seoul (Korea, Republic of),

^5^Graduate School of Public Health, Seoul National University - Seoul (Korea, Republic of),

^6^Seoul National University Bungang hospital - Seongnam (Korea, Republic of)

**Abstract**

**Background:** We have developed a novel intraperitoneal drug delivery system, HPIPAC (Hyperthermic Pressurized Intraperitoneal Aerosol Chemotherapy), which combines the gas-based HIPEC (Hyperthermic Intraperitoneal Chemotherapy) and PIPAC (Pressurized Intraperitoneal Aerosol Chemotherapy) methods using dual-flow nozzle. In this study, we analyzed the pharmacokinetic properties of oxaliplatin in the blood and tissues depending on the drug administration methods, which included intravenous administration, PIPAC, and HPIPAC in the swine model.

**Methods:** We administered 5 mg/kg of oxaliplatin to a pig weighing approximately 25 kg for each group. We collected blood sampling at 0, 2, 5, 10, 15, 30, and 45 minutes and 1, 1.5, 2, 3, 4, and 6 hours after oxaliplatin administration in each group. We harvested tissues (stomach, pancreas, spleen, small bowel, heart, kidney, liver, and peritoneum) after the final 6 hours of blood sampling. A liquid chromatography-tandem mass spectrometry (LC-MS/MS) method was performed to determine oxaliplatin in pig plasma and tissue homogenate samples. A protein precipitation procedure was applied for the sample pretreatment using acetonitrile as an extraction solution, and carboplatin was used as an internal standard.

**Results:** Between IV and PIPAC, plasma concentration was more than 100 times higher in IV delivery groups with statistical significance until 15 minutes after delivery. The same was observed for IV and HPIPAC with 10 times potency. HPIPAC had a higher concentration compared to PIPAC, and this trend was statistically significant until 15 minutes after initial delivery. Meanwhile, significant differences in tissue concentration were observed in the peritoneum and liver. HPIPAC showed five times higher concentrations in the peritoneum and the liver than IV and PIPAC, respectively.

**Conclusions:** HPIPAC showed less plasma and higher peritoneal concentration of oxaliplatin than the systemic route.

### PO 16 Comparison of the therapeutic efficacy of PIPAC Oxaliplatin versus PIPAC Cisplatin-Doxorubicin: experimental study in rabbit model with gastric peritoneal metastases #PO 16

#### Poster

A. Taibi^1^, M.L. Perrin^2^, C. Yardin^2^, S.M. Bardet^2^, S. Durand Fontanier^1^

^1^Digestive surgery, Limoges FRANCE, XLIM CNRS, UMR 7252, groupe BIOSANTE - Limoges (France),

^2^Limoges university, XLIM CNRS, UMR 7252, groupe BIOSANTE - Limoges (France)

**Abstract**

This study presents an experimental investigation of the feasibility in a rabbit model with gastric peritoneal metastases, evaluating the efficacy of PIPAC oxaliplatin (n=5) compared to PIPAC cisplatin-doxorubicin (n=5), and a group treated with physiological saline, on D8, D15, and D21. Assessment of the animals’ well-being score was undertaken prior to each PIPAC procedure. Abdominal CT-Scans were performed before PIPAC1 and after PIPAC3. During each PIPAC session, ascites volume and Peritoneal Cancer Index (PCI) were determined, along with biopsies from at least three nodules. Evaluation of Histological Response (HR) based on the Peritoneal Regression Grading Score (PRGS), were performed. Additionally, a detailed histological examination using Microscopic-Multiphoton-imaging was conducted.

The overall well-being score showed improvement in all groups (0.6 +- 0.55 for control group; 6 +- 2.24 for Cisplatin-Doxorubicin; and 7 +- 2.74, NS). The PCI score decreased solely in the Oxaliplatin group between PIPAC1 and PIPAC3 (11 +- 1.7 vs 8 +- 2.8 p=0.01). It remained stable in the Cisplatin-Doxorubicin group (11 +- 1.3 vs 11 +- 3.1 NS) but significantly increased in the control group (8 +-1.9 vs 21+-1.8 p=0.001). The PCI score post-PIPAC3 was notably elevated in the control group compared to the Oxaliplatin and Cisplatin-Doxorubicin groups (21+-1.8 vs 8+-2.8 vs 11+-3.1, respectively, p<0 .05). The median PRGS was recorded as being 2 for both Oxaliplatin and Cisplatin-Doxorubicin groups but significantly higher in the Control group (3.25 p=0 .0003). This experimental inquiry confirms the therapeutic impact of Oxaliplatin and Cisplatin-Doxorubicin administered via PIPACs on gastric peritoneal metastasis

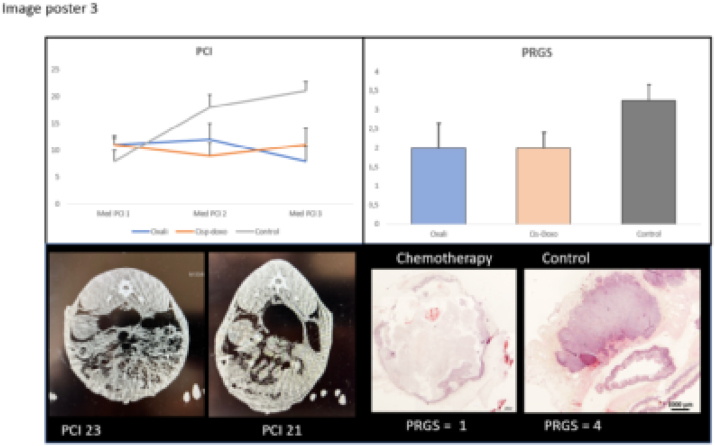



### PO 17 A new proteomic approach to detect and characterise microorganisms in Peritoneal Mucinous Carcinomatosis #PO 17

#### Oral communication or poster

A. Romero-Ruiz^1^, R. Pezzopane^1^, F.I. Bura^2^, M. Granados-Rodríguez^1^, A. Moreno-Serrano^2^, M. Torres-Martínez^2^, M.C. Vázquez-Borrego^2^, J. Alhama^1^, C. Michán^1^, Á. Arjona-Sánchez^3^

^1^Department of Biochemistry and Molecular Biology, University of Cordoba - Maimonides Biomedical Research Institute of Cordoba (IMIBIC) - Cordoba (Spain),

^2^Maimonides Biomedical Research Institute of Cordoba (IMIBIC) - Cordoba (Spain),

^3^Unit of Surgical Oncology, Department of Surgery, Reina Sofia University Hospital - Maimonides Biomedical Research Institute of Cordoba (IMIBIC) - Cordoba (Spain)

**Abstract**

**INTRODUCTION.** Pseudomyxoma peritonei (PMP) is a rare malignancy and the paradigm of Peritoneal Mucinous Carcinomatosis, which causes the continuous accumulation of mucus in the abdomen. The only available treatment involves complete cytoreductive surgery combined with hyperthermic intraperitoneal chemotherapy. Despite this aggressive management, relapses are common in PMP, leading to debilitating symptoms and a fatal end. It’s therefore important to look for ways to improve treatment and better understand the pathogenesis of the disease. In this context, microorganisms have previously been detected in PMP by genomic approaches, but data are limited. Our group aimed to develop the first method based on a “proteomic approach” to test and characterise the presence of microorganisms in mucinous tumour tissues.

**METHODS.** Samples obtained during CRS-HIPEC surgery were treated with our protein isolation protocol and then analysed by nanoHPLC-MS/MS. To identify and characterise the presence of microorganisms in the tumour tissue, a new human and microbial protein database was created.

**RESULTS AND CONCLUSIONS.** Our results demonstrate for the first time the presence of fungal proteins in mucin samples from PMP tumours (Figure 1). This work presents a novel proteomic and bioinformatic approach to identify the presence of microbiota. This is in line with the literature where different genera of bacteria and fungi have been identified by genomic approaches in different types of cancer. However, further research is needed to determine the role of this advance in the pathogenesis of mucinous tumours, to understand the mechanisms of oncogenesis and to develop better therapeutic strategies for these patients.

Frequency of proteins for each fungal family

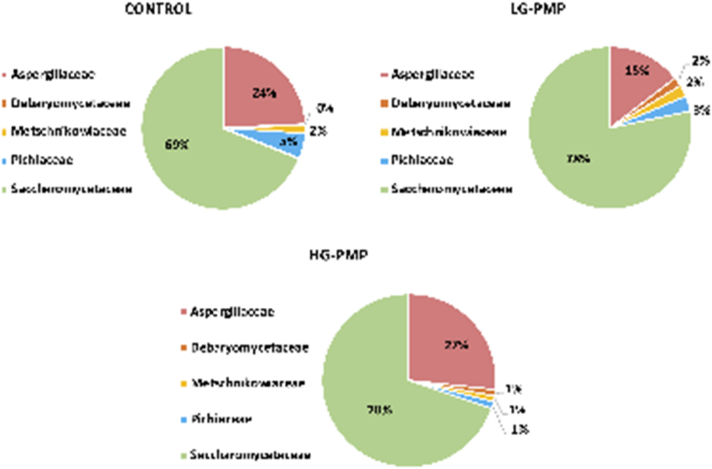



### PO 18 Differential tumor microenvironment (TME) changes in ovarian cancer (OC) responder versus non-responder to pressurized intraperitoneal aerosolized chemotherapy (PIPAC) – a single cell transcriptomics analysis #PO 18

#### Oral communication or poster

I. Bishara^1^, P. Cosgrove^1^, N. Ruel^2^, P. Frankel^2^, S. Yost^2^, S. Chang^2^, M. Raoof^2^, A. Bild^1^, A. Nath^1^, T. Dellinger^2^

^1^Beckman Research Institute of the City of Hope - Duarte (United States),

^2^City of Hope National Medical Center - Duarte (United States)

**Abstract**

**Background:** PIPAC is a promising treatment for select recurrent OC patients. No predictive biomarkers or clinical characteristics currently exist to predict response to PIPAC. We performed single cell RNA sequencing (scRNAseq) on serial peritoneal tumors from each PIPAC in an OC responder and non-responder, to characterize the interactions and evolution of cancer cells and normal cells in the tumor microenvironment to identify key response signaling.

**Methods:** One responder and one non-responder with recurrent OC were identified from a Phase I trial of PIPAC (cisplatin 10.5 mg/m2, doxorubicin 2.1 mg/m2 q6weeks; NCT04329494). The responder had an objective tumor response after 6 PIPAC cycles. The non-responder progressed with liver metastases after 2 PIPAC cycles. Tumor and normal peritoneal biopsies were collected at each PIPAC cycle, and scRNA-seq was performed using 10X Genomics Chromium Single Cell System. Cell types were annotated by SingleR and canonical cell type gene markers. Copy number alteration profiles of putative malignant cells were used to distinguish between malignant and normal cells. Pseudobulk samples were created for gene set and pathway enrichment analysis.

**Results:** From the temporal tumor compositions analysis, we observed a T-cell fraction reduction in the non-responder after two PIPAC cycles, while macrophage levels remained unchanged. Conversely, the responder experienced an increase in both T-cell and macrophage populations over six PIPAC cycles, indicating a more robust immune response to therapy. Pathway enrichment analysis for the responder displayed epithelial-mesenchymal transition (EMT), and oxidative phosphorylation pathways, while the non-responder revealed upregulation in cell cycle activity and beta-catenin signaling.

**Conclusions:** Serial evaluation of scRNAseq in a PIPAC responder vs. non-responder demonstrates differential TME changes with a trend towards increased T cell fractions associated with anti-tumor response. Clinical response to PIPAC was also associated with anti-neoplastic signaling through decreased invasive and metabolic pathways on pseudobulk pathway analyses.

### PO 19 A supramolecular injectable pH-responsive hydrogel tailor-made to improve intraperitoneal therapy: Results from preclinical evaluation in minipigs and rodent models for peritoneal metastases of colorectal and ovarian origin. #PO 19

#### Oral communication or poster

G.C. Van Almen^1^, P.P.K.H. Fransen^1^, A.G.W.E. Wintjens^2^, H. Braet^3^, K. Lenaerts^2^, W. Ceelen^4^, K. Remaut^5^, N.D. Bouvy^2^, I.H.J.T. De Hingh^6^, P.Y.W. Dankers^1^

^1^Dept. Of Biomedical Engineering, Eindhoven University of Technology and UPyTher BV - Eindhoven (Netherlands),

^2^Dept. Of Surgery, Maastricht University Medical Centre - Maastricht (Netherlands),

^3^Dept. Of Pharmaceutics, Ghent University and Cancer Research Institute Ghent - Ghent (Belgium),

^4^Dept. Of Human Structure and Repair, Ghent University and & Cancer Research Institute Ghent - Ghent (Belgium),

^5^Dept. Of Pharmaceutics, Ghent University and & Cancer Research Institute Ghent - Ghent (Belgium),

^6^Dept. Of Sugery, Catharina Hospital - Eindhoven (Netherlands)

**Abstract**

**Background:** The efficacy of currently used intraperitoneal chemotherapeutics is limited by the rapid clearance from the peritoneal cavity resulting in short drug exposure times. Hydrogels hold promise as drug delivery systems to improve local residence time of the therapeutic agent but are associated with difficulties in intraperitoneal administration and distribution. Here, we present a novel slow-release hydrogel that was specifically designed to overcome the topological challenges of the peritoneal cavity.

**Methods:** Unique supramolecular polymers were developed to create an injectable, pH-responsive hydrogel (UPy-PEG) that is well tolerated within the peritoneal cavity and has superior handling properties. Therapeutic efficacy and pharmacokinetics of drug-loaded UPy-PEG hydrogels was examined in rat models for peritoneal metastases of colorectal (CC531, WAG/Rij) and ovarian (SKOV3-IP2, athymic nude Hsd:RH-Foxn1rnu) origin using mitomycin C (MMC)- and paclitaxel (PTX), respectively. Next, translation to a clinically relevant scale was assessed after laparoscopic administration in minipigs.

**Results:** Both MMC- and PTX loaded hydrogels demonstrated a marked survival benefit as compared to intraperitoneally administered unformulated forms of MMC and PTX. Furthermore, MMC-loaded UPy-PEG demonstrated a favourable pharmacokinetic profile with prolonged release of MMC up to at least 6-8 hours and reduced plasma peak concentrations (110 vs 350 µg/L). Additionally, the anti-adhesive properties of UPy-PEG hydrogels proved beneficial for intraperitoneal application and significantly reduced the formation of peritoneal adhesions – even outperforming commercially used Hyalobarrier ® in an abrasion model in rats. Finally, clinical feasibility was demonstrated in minipigs using standard laparoscopic procedures and equipment, without clinical signs of discomfort or histopathological and haematological adverse effects up to 14 days.

**Conclusion:** UPy-PEG hydrogels can be administered conveniently and improve the efficacy of intraperitoneal therapy through optimal peritoneal distribution and prolonged drug exposure. These studies warrant further evaluation towards clinical application of drug-loaded UPy-PEG hydrogels in patients with peritoneal metastases.

### PO 20 Polymer Brush-Grafted Single-Atom Nanozyme with High Catalytic Activity, Tumor Targeting Ability, and Biocompatibility for Colorectal Peritoneal Metastasis Treatment #PO 20

#### Oral communication

X. Qin^1^, D. Miao^2^, R. Huang^1^, H. Wang^1^

^1^Department of Colorectal Surgery, the Sixth Affiliated Hospital, Sun Yat-sen University - Guangzhou (China),

^2^PCFM Lab, School of Chemistry, Sun Yat-sen University - Guangzhou (China)

**Abstract**

**Background:** Peritoneal metastasis from colorectal cancer (CRC) is a significant clinical challenge. Recent years have seen promising developments in the use of single-atom nanozymes as anticancer nanomaterials for treating CRC peritoneal metastasis.

**Objective:** This study aims to design a novel single-atom nanoenzyme for intraperitoneal treatment of this condition.

**Methods:** We prepared a carbon-based single-atom nanozyme, Fe-N-Csp, via pyrolysis and modified it with a polyacrylonitrile molecular brush through free radical polymerization to improve biocompatibility. Then, aminoethyl anisamide, known for tumor targeting, was grafted onto the polymer brush chain.

**Results:** Tests on mice of peritoneal metastasis with CT26 colon cancer cells showed that our functionalized nanoenzyme, FCPNA, retained catalytic activity and enhanced tumor targeting compared to Fe-N-Csp. Moreover, FCPNA demonstrated superior antitumor activity in vitro and in vivo. The combination of FCPNA with oxaliplatin significantly improved the anti-tumor effect, suggesting a synergistic interaction. After 14 days of treatment, the FCPNA group showed normal levels of inflammatory cells, indicating better biocompatibility compared to Fe-N-Csp.

**Conclusions:** In conclusion, we successfully synthesized FCPNA, a highly active, target-specific, and biocompatible single-atom nanoenzyme, which shows synergistic effects with oxaliplatin in treating CRC peritoneal metastasis.

Schematic overview

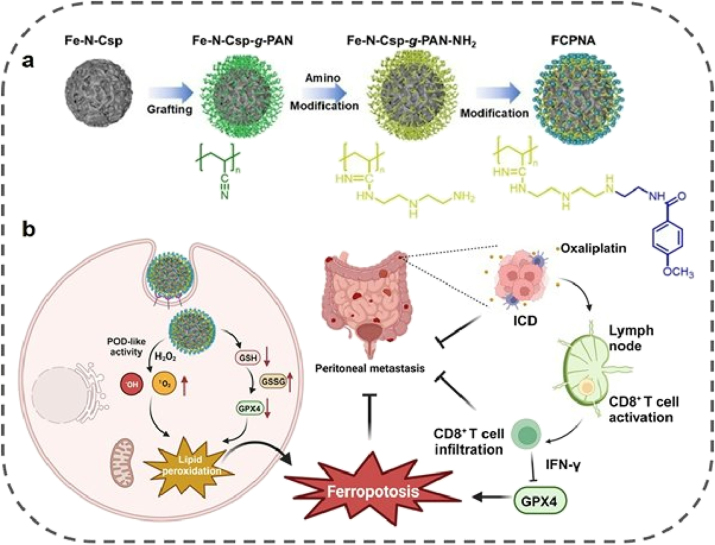



## Diagnosis and staging of peritoneal surface malignancies

### PO 145 Inter-reader agreement in calculation of the CT-PCI using e-PROMISE to evaluate colorectal peritoneal carcinomatosis #PO 145

#### Oral communication or poster

L. Vilcot^1^, W. Seye^1^, M. Auger^2^, F. Dumont^3^

^1^M.D. - Nantes (France),

^2^M.D. - Rennes (France),

^3^M.D., Ph.D. - Nantes (France)

**Abstract**

**Background:** CT is the reference in imaging for assessing peritoneal carcinomatosis and extraperitoneal lesions in colorectal cancer. The PeRitOneal MalIgnancy Stage Evaluation (PROMISE) internet application provides computer-assistance for producing standardized reports of the extent of peritoneal metastases and automatically calculating the peritoneal cancer index (PCI). The aim of this study is to evaluate inter-reader reproducibility in the calculation of PCI using it and to analyze the factors of disagreement.

**Methods:** This retrospective single-center study was based on a series of CT including abdominopelvic acquisition with contrast injection at venous time (70 sec) with the possibility of multi-planar reconstruction. Each radiologist (2 seniors and 1 junior) performed a pre- and post-neoadjuvant radiological PCI which were compared with both each other and the surgical PCI.

43 patients were included with a median age of 62 years. The tumor site was the right rectum for 19 patients, the left colon for 21 patients, and the rectum for 3 patients. The median number of neoadjuvant chemotherapies was 6. The intra-class correlation coefficient was average between the 3 readers (0.7) and better between the seniors. The main factors of disagreement were the description of tissue infiltration and the median size of the largest nodule (p<0.01). The junior radiologist tended to describe fewer nodules than the seniors but was borderline significant (p=0.06). The concordance between the radiological PCI of the three readers and the surgical PCI was poor regardless of the radiologist’s expertise in pre-chemotherapy and decreased in post-chemotherapy.

**Conclusion:** Using the e-PROMISE, the correlation for calculating the radiological PCI score was average between the three readers. However, the concordance was good between the experienced radiologists because the junior detected fewer carcinosis nodules and less tumor infiltration. The concordance between CT and surgical PCI was poor and affected by chemotherapy.

### PO 53 FDG-PET/CT as a method of patient selection and response evaluation in patients with peritoneal metastasis treated with Pressurized IntraPeritoneal Aerosol Chemotherapy. The PIPAC-OPC7 study #PO 53

#### Oral communication or poster

M. Graversen^1^, S. Roensholdt^1^, S. Detlefsen^2^, M.G. Hildebrandt^3^, P. Pfeiffer^4^, M.B. Mortensen^1^

^1^Odense PIPAC Center, department of Surgery, Odense University Hospital - Odense (Denmark),

^2^Odense PIPAC Center, department of Clinical Pathology, Odense University Hospital - Odense (Denmark),

^3^Odense PIPAC Center, department of Nuclear Medicine, Odense University Hospital - Odense (Denmark),

^4^Odense PIPAC Center, department of Clinical Oncology, Odense University Hospital - Odense (Denmark)

**Abstract**

**Background:** Pressurized IntraPeritoneal Aerosol Chemotherapy (PIPAC) is increasingly used in patients with peritoneal metastasis (PM). Many studies use RECIST or Peritoneal Cancer Index to evaluate treatment response, but this assessment remains challenging and lacks consensus. The histological Peritoneal Regression Grading Score (PRGS) is also often used and holds prognostic value with good reproducibility. Imaging with FDG-PET/CT is the cornerstone during response assessment in several cancer types, providing information on both morphological changes as well as qualitative and quantitative metabolic changes. This is the first study to evaluate the utility of FDG-PET/CT during PIPAC for patient selection and response evaluation in patients with PM.

**Methods:** A prospective pilot study will be conducted on 16 patients suffering from peritoneal metastasis originating from four primary cancers: gastric, pancreatic, ovarian, and colorectal cancer. Patients are treated in a series of three PIPACs; peritoneal biopsies are taken during each treatment and evaluated according to PRGS. The FDG-PET/CT scans will be completed at baseline and after PIPAC1 and 3. Patients with PET-negative PM at baseline are still included to investigate if PIPAC itself leads to PET-positive changes after PIPAC1.

**Results:** Inclusion started in March 2024. The primary objective is the number of patients with metabolic response according to FDG-PET/CT evaluated by PERCIST1.0 after PIPAC3. Secondary objectives include number of patients with metabolic response after PIPAC1, number of patients with PET-positive peritoneal surface after PIPAC1 despite negative baseline PET/CT (false positive/feasibility), and the number of patients where FDG-PET/CT (evaluated by PERCIST one-lesion) agrees with histological response according to PRGS after PIPAC1 and 3.

**Conclusions:** We hypothesize that FDG-PET/CT and PERCIST1.0 is feasible and can reflect metabolic response in patients with PM treated with PIPAC. This study may provide important data needed to plan future randomized controlled trials within the treatment of PM.

### PO 147 Patterns of preoperative tumour markers can predict resectability and prognosis of peritoneal metastases – a clustering analysis #PO 147

#### Oral communication

M. Enblad^1^, P. Cashin^1^, L. Ghanipour^1^, W. Graf^1^

^1^Department of surgical sciences, Uppsala university - UPPSALA (Sweden)

**Abstract**

**Background:** Prediction of open-close and long-term outcome is challenging and prognostic scores often include factors not known before cytoreductive surgery (CRS) and hyperthermic intraperitoneal chemotherapy (HIPEC). The aim was to analyse if patterns of preoperative tumour markers could aid in prediction of open-close and outcome of patients with pseudomyxoma peritonei (PMP) or colorectal peritoneal metastases (PM).

**Method:** All patients accepted for CRS and HIPEC for PMP or colorectal PM at Uppsala university hospital, 2013−2021, were included. Tumour markers CEA, CA19-9, CA125, CA72-4, and CA15-3 were clustered using the k-means algorithm and the average silhouette width determined the optimal numbers of clusters.

**Results:** Clustering of PMP (n=139) and colorectal PM (n=212) patients resulted in two clusters each. PMP-Cluster1 (n=132) had better prognosis, 16 (12%) open-close, median peritoneal cancer index (PCI) 19 (I.Q.R 8−29), and 1 (1%) with signet ring cells (SCs). PMP-Cluster2 (n=7) patients had poor prognosis, 3 (43%) open-close, PCI 37 (I.Q.R 36−39), and 3 (43%) with SCs. In colorectal-Cluster1 (n=189), there were 33 open-close (17%), PCI 11 (I.Q.R 6−21), and 19 (10%) with SC. Colorectal-Cluster2 (n=23) had poorer prognosis, 7 (23%) open-close, PCI 26 (I.Q.R 22−32), and 6 (26%) with SCs. Both PMP- and colorectal Cluster2 were characterized by at least four markedly elevated tumour markers of which CA72-4 and CA125 were elevated in almost all of these patients (Figure 1).

**Conclusion:** Elevation of several preoperative tumour markers is associated with increased risk of open-close, high PCI, SCs, and poor prognosis. CA72-4 and CA125 deserves increased attention.

Clustering of tumour markers

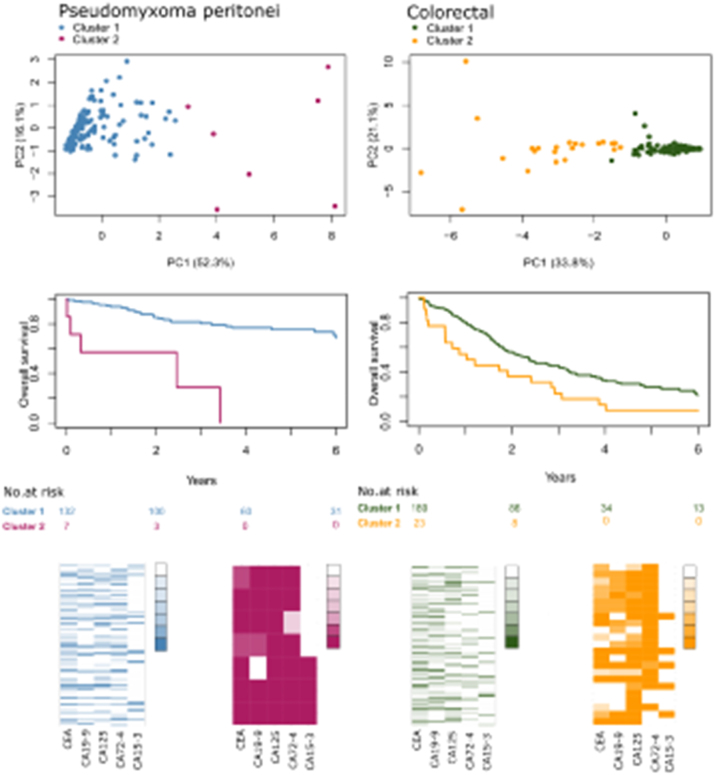



### PO 148 Peritoneal tumour DNA in peritoneal fluid: emerging tool for peritoneal metastases detection #PO 148

#### Oral communication or poster

A. Mariani^1^, H. Blons^2^, P. Laurent-Puig^2^, Z. Aziz^3^, G. Amira^2^

^1^Surgical oncology, HEGP - paris (France), ^2^Molecular oncology, HEGP - paris (France), ^3^Digestive oncology, HEGP - paris (France)

**Abstract**

**Introduction:** Circulating tumour DNA (ctDNA), is a promising biomarker that provides valuable information in the era of cancer detection. As ctDNA refers to DNA coming from cancer cells, it may be used to detect minimal residual disease, which is by definition occult malignancy not detected using conventional biological or imaging techniques. ctDNA was largely studied in the bloodstream however, cancer DNA fragments are present in non-blood body fluids. The aim of the study was to evaluate the feasibility and the clinical impact of tumoral DNA in peritoneal fluid (ptDNA) regardless of cytology.

**Methods:** Patients with proven and exclusive peritoneal metastases from ovarian, gastric and colorectal cancers were included. Peritoneal fluid was obtained from ascites when present or peritoneal washing when necessary. Another blood sample was collected for plasmatic ctDNA analysis, at the same time.

**Results:** 17 patients were included with gastric (n=4), colorectal (n=8) or ovarian (n=5) tumours. At the time of sampling, 85% of patients were already treated with chemotherapy. PtDNA was positive in 13 patients (76.4%). Ten patients had ascites (9 positive samples) and peritoneal washing was used for the 7 others (4 positive samples). Plasmatic ctDNA was positive in 3 patients (17.6%). Mutant allele fraction (MAF) and mutant copies per ml (MTc/ml) were higher in peritoneal fluid than in plasma (median MAF = 32% ; median MTc/ml = 142869 in peritoneal fluid vs median MAF = 2.6% ; median MTc/ml = 4343 ). Altogether, conventional cytology was positive in 41% of patients (n=5 /12, 5 samples not available) as compared to 75%, (9/12) for ptDNA in the 12 corresponding fluids.

**Discussion:** Here, we showed that ptDNA in peritoneal fluid had the best sensitivity for detecting tumoral activity into the peritoneum in comparison with conventional cytology or plasmatic ctDNA, independently of the presence of ascites.

### PO 149 Peritoneal carcinomatosis – or just gallstones? #PO 149

#### Poster

N. Goh^1^, Y.L. Wang^2^, C.C.H. Siew^1^

^1^Tan Tock Seng Hospital - Singapore (Singapore), ^2^Khoo Teck Puat Hospital - Singapore (Singapore)

**Abstract**

Mimickers of peritoneal carcinomatosis are occasionally encountered – indistinguishable on imaging with uninformative biopsy results. Differential etiologies include infections like tuberculosis and actinomycosis and other neoplastic processes like lymphoma. Due consideration given to alternative diagnoses can help rationalize exhaustive investigations and avoid major morbid surgeries.

We present a diagnostic dilemma wherein an elderly patient who had recent uneventful laparoscopic cholecystectomy was referred for evaluation of peritoneal carcinomatosis. During the cholecystectomy, the gallbladder was inadvertently entered with resultant localized spillage of pigmented gallstones that were aspirated by the end of surgery.

Following image guided core biopsy of a solid peritoneal lesion noted on computed tomographic imaging, the patient developed severe sepsis with persistent febrile episodes requiring hospital admission and broad-spectrum intravenous antibiotics. Histology returned as inflammatory infiltrates without evidence of malignancy and tumour markers (CEA and CA-125) were not elevated. Tissue culture results and tuberculosis tests (AFB smears) were negative.

The patient underwent repeat image guided biopsy which yielded culture results positive for Enterobacter clocae and again histology results negative for malignancy. Treatment consisting of a course of intravenous antibiotics was completed and repeat cross-sectional imaging demonstrated resolution of these lesions, consistent with an infective etiology.

Gallstone spillage during cholecystectomy is not uncommon and most patients remain asymptomatic. In the vast minority of patients who eventually develop symptoms, reported consequences include intra-abdominal abscess formation and fistula formation. The biopsy of such peritoneal lesions has shown to result in severe sepsis – a key consideration when performing biopsies in an outpatient setting.

CT scan - arrows depicting peritoneal lesions

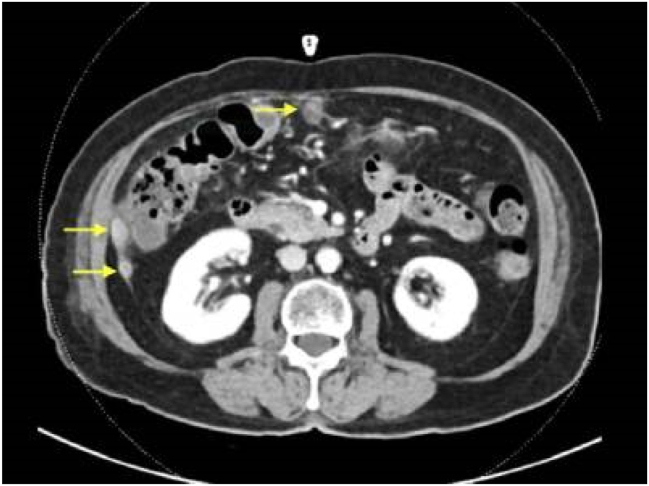



### PO 150 Retroperitoneal lymph node dissection – How I Do It #PO 150

#### Oral communication or poster

M.D. Ray^1^, G. Singh^1^

^1^AIIMS new delhi - NEW DELHI (India)

**Abstract**

**Background:** Retroperitoneal lymph nodes(RPLNs) are “safe haven” for chemoresistance tumor clones and potential site for relapse. Retroperitoneal lymph node dissection(RPLND) is an essential part of surgical practice to help in accurate staging of cervical, endometrial malignancies and therapeutically address malignancies of testis and ovary. RPLND is associated with acceptable complications like vascular injury, lymphatic leaks, infection, and intestinal fistula. In this paper we sought for techinque of RPLND to reduce complications and yield better outcome.

**Methods:** We introduce a RPLND technique which includes Zone based approach with Finger and Curtain dissection, practicing at a tertiary care centre in northern India.Retroperitoneum is divided into in 6 zones by three imaginary vertical and horizontal lines. Zone 2,3,6 comprises paraaortic lymph node and Zone 2,5,6 are high alert zones. Dissection of lymph node are done en block zone wise, focusing on vital structures in each zone. Fibrofatty tissue is raised as curtain ,while finger retracting and safeguarding vital structures by palpating and protecting them.

**Results:** A prospectively maintained database of patients undergoing RPLND using above technique from 2012 to 2023 were analysed. A total of 362 patients with mean age of 55.2yrs were included. Mean LN yield was 25.2. with a lymph node positivity rate of 49.2%. Mean blood loss was 403 ml. 30 (8.28%) patients developed complications.IVC injury was found in 6 patients (1.6%). 21(5.80%) patients developed lymphatic leak of which 13 (3.59%) had lymphatic ascites and 8 (2.4%) had chylous ascites. All of them were managed conservatively. No mortality was observed for this procedure.

**Conclusion:** RPLND by zone wise dissection with finger and curtain technique seems feasible, replicable and oncologically acceptable technique. Zone based dissection helps in prospective identification of nerves and fellow vessels with simultaneous safeguarding of vital structures.It helps in standardising the technique for better outcome and acceptable morbidity.

### PO 151 Should transmural recto-sigmoid invasion alone in ovarian cancer be considered as FIGO stage IV? -Opinion from tertiary cancer care centre in India #PO 151

#### Oral communication or poster

B. Bhukkal^1^, M.D. Ray^1^, V. Ravi^1^

^1^AIIMS - New Delhi (India)

**Abstract**

**Background:** Epithelial ovarian cancer (EOC) is the most common subtype of ovarian cancer. According to the FIGO staging system of ovarian cancers, any transmural bowel invasion is classified as stage IV disease. Transmural recto-sigmoid invasion is often due to the local progression of the disease and should not be included under stage IV disease.

**Methods:** Our study is a retrospective analysis of ovarian cancer database from 2012 to 2023 at the Department of surgical oncology at a tertiary cancer centre in India. A total of 476 patients with ovarian cancer were analyzed. Patients with stage IV disease were divided into two groups, in group A: those with rectosigmoid invasion without other features of stage IV disease, and in group B: those with parenchymal metastasis, pleural disease, transmural bowel invasion other than in recto-sigmoid and lymph nodal metastasis irrespective of recto-sigmoid invasion were included. The baseline characteristics, treatment, and survival outcomes of both groups were compared.

**Results:** In group A 31 patients and in group B 36 patients were included. Baseline and treatment characteristics in both groups were comparable with no difference, except for the number of patients who received NACT and surgical PCI scores. Mean (SD) recurrence-free survival (RFS) was 32.5 months (20.6) in group A and 17.2 months (13.4) in group B with HR 0.43 (95% CI 0.20 - 0.93); P=0.032. There was a statistically significant difference in overall survival (OS) between both groups, the mean (SD) OS in group A was 45.9 months (26.2), and in group B was 26.9 months (18.1) with HR 0.48 (95% CI 0.19 – 0.98); P=0.046.

**Conclusion:** Our study revealed that among stage IV epithelial ovarian cancers, a subset of patients only with resectable rectosigmoid involvement exhibited notably enhanced survival rates than other stage IV ovarian cancers when optimal cytoreduction was executed

## Educational on peritoneal surface malignancies

### PO 152 Feasibility and Efficacy of a Palliative Education Program for Peritoneal Surgeons and Trainees #PO 152

#### Poster

L.C.K. Wong^1^, W.K.D. Juan^1^, P.L. Koh^1^, S.H.X. Cheok^1^, M. Cai^1^, C.J. Seo^1^, C.A.J. Ong^1^, C.S. Chia^1^, J.S.M. Wong^1^

^1^Department of Sarcoma, Peritoneal and Rare Tumours (SPRinT), Division of Surgery and Surgical Oncology, National Cancer Centre Singapore and Singapore General Hospital, Singapore - Singapore (Singapore)

**Abstract**

**Background:** Patients with peritoneal surface malignancies (PSM) have advanced cancer and are candidates for palliative care (PC). However, there is a lack of PC awareness, knowledge, and training among peritoneal surgeons and surgical trainees, leading to suboptimal care. We therefore piloted a palliative education workshop in collaboration with a specialist PC team. We hypothesized that the workshop is feasible and efficacious in improving overall PC knowledge and awareness. Barriers to PC in surgical practices were also explored.

**Methods:** Specialist surgeons and surgical trainees were enrolled in a 3-day palliative education workshop. Feasibility was measured by completion rates and efficacy was evaluated using a mixed-method approach comprising of structured questionnaires and semi-structured qualitative interviews. Grounded theory concepts were applied to perform thematic analysis to explore barriers and facilitators to PC.

**Results:** The workshop was feasible with a full completion rate for 16 participants. 62.5% expressed minimal palliative experience or knowledge prior to the workshop. Barriers to PC delivery identified include misalignment of values and cultures, as well as deficiencies in skills such as communication and management of symptoms or psychosocial issues. There was an increase in knowledge scores pre- versus post-workshop, with a mean difference of 13.8 (95% confidence interval: 8.8–18.7, p < 0.01). At 9 months post workshop, 100% of respondents agreed/strongly agreed that the course helped to develop relevant palliative skills and knowledge, was applicable to daily clinical work, led to improved work performance and increased confidence in recognising PC needs, providing basic palliative support, as well as making appropriate referrals. 83% have shared knowledge and skills learnt with colleagues, of which all have experienced consequent improvements in team performance and processes.

**Conclusion:** PC education is feasible and efficacious. By addressing barriers to PC delivery, we can potentially improve the quality of care provided to PSM patients.

### PO 153 Synchronous peritoneal metastases from esophageal cancer in a nationwide cohort: an overview of the incidence, risk factors, treatment and survival. #PO 153

#### Oral communication or poster

R. Seuter^1^, L. Galanos^1^, M. Luyer^1^, A. Rijken^1^, R. Verhoeven^2^, I. De Hingh^1^, F. Van Erning^2^

^1^Catharina Hospital Eindhoven - Eindhoven (Netherlands),

^2^IKNL - Eindhoven (Netherlands)

**Abstract**

**Introduction:** This study aimed to provide insights into synchronous peritoneal metastases (PM) in esophageal cancer by analyzing incidence, risk factors, treatment and survival, using population-based data.

**Methods:** Netherlands Cancer Registry data were used to select esophageal cancer patients diagnosed between 2015–2020. Crude incidence rates and incidence trends for synchronous PM in esophageal cancer were calculated using the Revised European Standardized Rate (RESR) and the estimated annual percent change (EAPC). Logistic regression analyses identified factors associated with the presence of synchronous PM. Treatment of PM patients was investigated. Median overall survival (OS) for solitary PM, PM with systemic metastases, and systemic metastases only was calculated using the Kaplan-Meier method.

**Results:** The total study population consisted of 16,084 patients, of whom 672 (4.2%) were diagnosed with synchronous PM. The overall RESR of synchronous PM from esophageal cancer patients was 0.66 per 100,000 persons per year, increasing from 0.56 in 2015 to 0.75 in 2020 (EAPC +9.7%). Factors associated with the presence of synchronous PM were: distal tumor location, age <70, higher tumor/nodal stages, poor/undifferentiated tumor differentiation, adenocarcinoma morphology, and WHO performance status ≥2. Treatment primarily involved best supportive care (44%) and systemic therapy (31%). Median OS of all PM patients was 3.1 months (figure 1).

**Conclusions:** A rising incidence of synchronous PM was observed, affecting approximately one in 24 patients with esophageal cancer. With 3.1 months, median overall survival in synchronous PM is poor. Further research is required to improve diagnostics and to find more effective treatment strategies.

Figure 1.

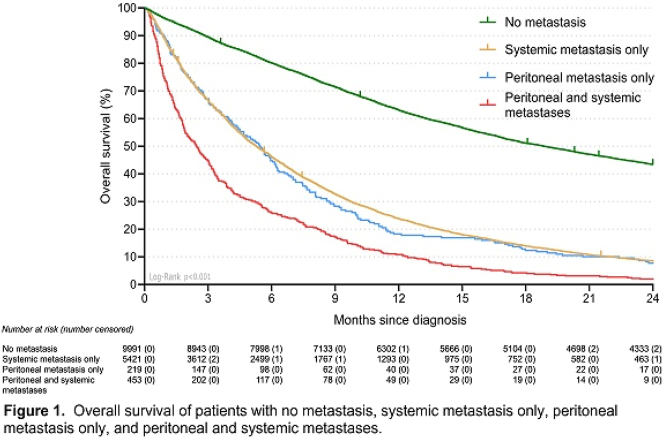



### PO 154 Influence of biennial fecal immunochemical testing (FIT) for colorectal carcinoma on the incidence of synchronous colorectal peritoneal metastases #PO 154

#### Oral communication or poster

L. Galanos^1^, A. Rijken^1^, M. Elferink^2^, N. Kok^3^, F. Van Erning^2^, I. De Hingh^1^

^1^Catharina hospital - Eindhoven (Netherlands),

^2^Netherlands Comprehensive Cancer Organisation - Utrecht (Netherlands),

^3^Netherlands Cancer Institute - Amsterdam (Netherlands)

**Abstract**

Biennial fecal immunochemical testing has been implemented to decrease incidence and mortality of colorectal cancer. In this study, we aim to assess the incidence of colorectal cancer with synchronous peritoneal metastases (CPM) before and after implementation of screening, in a nationwide cohort.

All patients diagnosed with CPM from colorectal origin between 2009-2022 were selected from the Netherlands Cancer Registry. Crude rates of CPM incidence and cumulative CPM incidence were calculated and compared with the expected CPM incidence and the expected cumulative incidence of CPM. Expected incidence was extrapolated from the incidence in the years prior to screening initiation (2009-2013).

Of 9,238 patients with CPM, 6,437 (70%) patients were eligible for screening (i.e. aged 55-75 years). For the total population, the observed CPM incidence shows an overall increase until screening initiation; from 3.6 (2009) to 4.4 (2014) per 100,000 individuals. Post-screening initiation an overall decrease in CPM incidence was observed; from 4.2 (2015) to 3.5 (2022) per 100,000 individuals. Within the screening eligible population, a similar initial increase in incidence of CPM was observed: 5.1 (2009) vs. 8.8 (2022) per 100,000 individuals. However, the period post-screening initiation the observed CPM incidence stabilized, from 8.6 (2015) vs. 8.9 (2022) per 100,000 individuals (figure1). The observed and expected number of CPM differed significantly in the overall Dutch population (observed 9,238 individuals vs. expected 10.440 individuals; p<0.001) and in the screen-eligible population (observed 6,437 individuals vs. expected 7,992 individuals; p<0.001).

An overall declining trend was observed regarding the CPM incidence in the Dutch population, since initiation of colorectal cancer screening. The observed incidence was lower as compared to the expected incidence, both in the overall Dutch population and in the screen-eligible population. These findings imply that CRC screening might result in a decrease of patients diagnosed with colorectal peritoneal metastases.

## Intraperitoneal treatment for peritoneal surface malignancies

### PO 21 Clinical evaluation of Icodextrin delivery as pressurized aerosol (PIPAC): Pilot study of antiadhesive - peritoneal effects #PO 21

#### Oral communication

X. Delgadillo^1^, A. Hytham^2^, P. Wuthrich^3^

^1^CMC Volta - La Chaux-de-Fonds (Switzerland),

^2^Carl Von Basedow-Klinikum Saalekreis - Merseburg (Germany),

^3^Clinique de Genolier - Les Muids (Switzerland)

**Abstract**

**BACKGROUND.-** There are currently few options for the prevention of metastatic peritoneal implantation of tumoral cells. Icodextrin 7,5% a glucose polymer derived from starch that is used as an osmotic agent solution used in peritoneal dialysis, has demonstrated to have prolonged activity against peritoneal metastasis (PM). **METHODS.-** Prospective study including patients with CRC-PM, receiving standard Icodextrin 7.5% solution by aerosolization through PIPAC deliery of fluids. Chi^2^ Test was used for analysis. **RESULTS.-** Between October 2021 to October 2023, four patients qualified & were included in the study. If few PM-adhesions were evidenced during laparoscopy, specific data was collected, to evaluate the Odds Ratio OR, & estimated results has been extrapolated with a concern that additional data would enhance overall results. 3 male & 1 female patients were included. They underwent 2 PIPAC Ico-7.5% aerosolization procedures using Capnopen® device. No complications, nor mortality. PCI ∼ 8 was obtained (6-10). Late follow up demonstrated no adhesions. **CONCLUSIONS.-** Icodextrin solution 7.5% offers the first feasible alternative to conventional dextrose solutions. It is effective, well tolerated and appears to be most useful in situations of reduced or inadequate UF with dextrose, including in high and high-average transporters.

Icodextrin 7.5% PIPAC delivery.

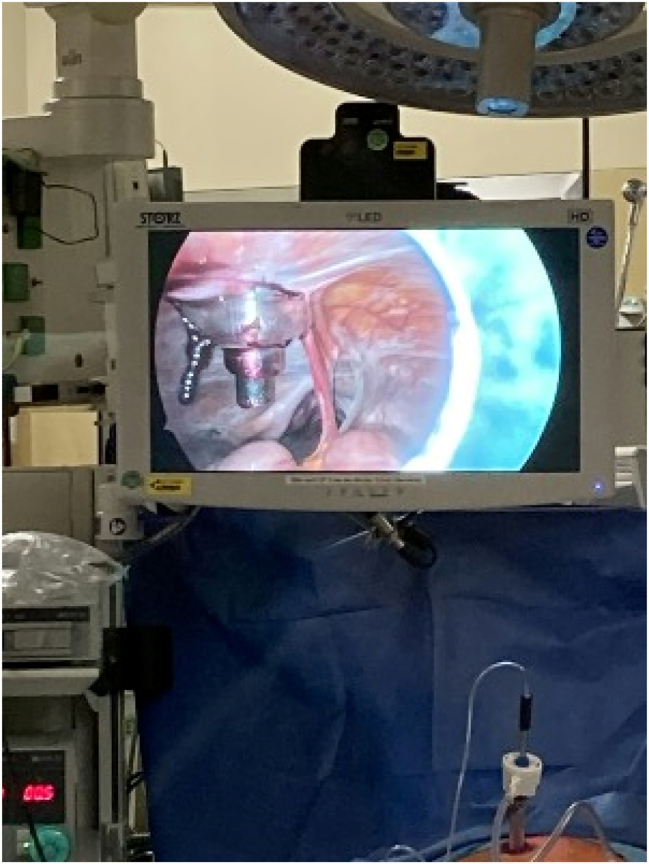



### PO 22 A multi-nozzle nebuliser does not improve tissue drug delivery during PIPAC #PO 22

#### Oral communication

I. Sautkin^1^, J. Weinreich^1^, M.A. Reymond^1^

^1^National Center for Pleura and Peritoneum, University Hospital Tübingen, Germany - Tübingen (Germany)

**Abstract**

**Introduction:** After PIPAC with the Capnopen® (Capnopharm, Tübingen, Germany), heterogeneous drug distribution was reported. To improve drug distribution, we developed a three-nozzle nebuliser (“Triplepen”) and compared the performance of both devices ex-vivo.

**Methods:** In physical experiments, Capnopen® and Triplepen with axial rotation were compared. The aerosol granulometry (MAD) was determined by laser diffraction spectrometry. The relative blue ink staining intensity and integrated density (RID) were analysed on a blotting paper with ImageJ® to assess the homogeneity of spatial distribution. Finally, we compared the tissue aerosol uptake in real-time and the tissue concentration of cisplatin and doxorubicin in the ex-vivo enhanced inverted bovine urinary bladder (eIBUB).

**Results:** To achieve the recommended upstream pressure >11bar, a flow of 3.4ml/s was needed for the Triplepen vs. 0.6ml/s for the Capnopen®, resulting in a shorter aerosolisation time (1.0 vs. 5.6min for 200ml, respectively). The aerosol droplet size (MAD) was smaller in the Capnopen® 35.6 (CI 34.5-36.6) vs. 43.2 (CI 28.0-58.4) (axial nozzle) and 42.3 (CI 39.3-45.3) and 45.5 (CI 37.6-53.5) µm (lateral nozzles) in the Triplepen. The spray pattern of the Triplepen had three spots but was inhomogenous. Based on the RID, the staining was more homogeneous with Capnopen® (p≤0.001). Real-time tissue drug uptake did not differ significantly between Capnopen® vs. Triplepen (p=0.07). Cisplatin tissue concentration was 39% less after aerosolisation with the Triplepen vs. Capnopen® (14.4±6.3 vs. 23.4±9.2ng/ml, p<0.001). Similarly, Doxorubicin tissue concentration was lower in the Triplepen group (1.3±1.2 vs. 1.5±1.2ng/ml, p=0.47).

**Conclusion:** The Triplepen did not provide the expected results. Cisplatin (p<0.001) and Doxorubicin (ns) tissue concentrations were lower with the Triplepen vs. the Capnopen®. There was no clear advantage of the Triplepen concerning the homogeneity of blue ink staining and DOX tissue concentration ex-vivo. Technical and medical equivalence between the Triplepen and the CE-certified device (Capnopen®) could not be verified.

### PO 23 The use of Cytoreductive Surgery (CRS) and Hyperthermic Intraperitoneal Chemotherapy (HIPEC) in pediatric peritoneal metastasis #PO 23

#### Oral communication

J. Spiliotis^1^

^1^European Interbalkan Medical Center - THESSALONIKI (Greece)

**Abstract**

**1. Background:** Peritoneal malignancies in children are rare, but are associated with poor outcome.

CRS and HIPEC have been applied to pediatric population in recent years. The role of this treatment remains controversial owing to the rarity of the disease and the limited number of cases.

**2. Methods:** A retrospective analysis from 2003 to 2023 identified 16 children with PM who underwent CRS and HIPEC from our group.

**3. Results:** The median age of the children was 14 years (range 8-16.4). The histological types included desmoid tumors (n=5), Wilms tumors (n=5), rhabdomyosarcoma (n=2) and other (n=4). The median peritoneal cancer index was 10.2 (range 6-28). There is 1 postoperative death. The median PCI 10.2 (range 6-28).

The median operation time was 6h (range 4-10). There is one postoperative death 1/16 (6.25%) due to massive pulmonary embolism in a colon cancer patients.

All patients were routinely placed with a Levine (gastric tube) after CRS and HIPEC which removed from 4th to 8th postoperative day. No evidence of renal failure or other metabolic complications.

The major complications according to the Clavien-Dindo were 3/16 (18.8%).

Postoperatively patients received chemotherapy (n=13) and radiotherapy (n=8) according to the different received molecular-targeted therapy. From the 14 patients 8 relapsed after CRS + HIPEC, tree of them in peritoneal cavity and five in Liver/or Lungs. The mean time of relapse disease is 2 years after CRS plus HIPEC.

**4. Conclusion:** CRS+HIPEC are safe and feasible in children with good selection of the cases.

### PO 24 A phase II study of intraperitoneal paclitaxel combined with gemcitabine plus nab-paclitaxel for pancreatic cancer with peritoneal metastasis. #PO 24

#### Oral communication or poster

N. Takahara^1^, Y. Yousuke^2^, H. Hironori^3^, S. Takashi^4^, I. Hiroyuki^5^

^1^Department of Gastroenterology, Graduate School of Medicine, The University of Tokyo - Tokyo (Japan),

^2^Department of Endoscopy and Endoscopic Surgery, Graduate School of Medicine, The University of Tokyo - Tokyo (Japan),

^3^Department of Chemotherapy, The University of Tokyo Hospital - Tokyo (Japan),

^4^Department of Hepato-Biliary-Pancreatic Medicine, The Cancer Institute Hospital, Japanese Foundation for Cancer Research - Tokyo (Japan),

^5^Department of Gastroenterology, Graduate School of Medicine, Juntendo University - Tokyo (Japan)

**Abstract**

【Background】 Peritoneal dissemination is a common metastatic form of pancreatic cancer and is an important prognostic factor. Given the promising results of intraperitoneal paclitaxel administration for peritoneal dissemination in various cancers, we developed a novel regimen of intraperitoneal paclitaxel combined with the standard treatment of gemcitabine/nab-paclitaxel for pancreatic cancer. Here, we report the safety and efficacy of this therapy in a phase II trial.【Methods】 This study was a multicenter, prospective, non-randomized trial involving patients with unresectable advanced pancreatic cancer and peritoneal metastasis who had not previously received chemotherapy. The primary endpoint was overall survival (OS), and the secondary endpoints included progression-free survival (PFS), tumor response, rate of negative peritoneal cytology, and safety. If the lower limit of the 80% confidence interval (CI) for median OS exceeded 7 months, the treatment was deemed worthy of further investigation in a phase III trial.【Results】 A total of 35 patients were enrolled from August 2019 to December 2022 (median age 63 years, male/female = 23/12, PS 0/1 = 19/16, liver metastasis/lymph node metastasis = 12/9, no ascites/minimal/moderate/severe ascites = 16/15/3/1). The median OS was 12.9 months (80% CI, 9.5–19.5), and the median PFS was 6.1 months (80% CI, 4.2–9.6). The response rate and disease control rate were 17.1% and 62.9%, respectively. A negative peritoneal cytology rate of 38.7% was achieved. Curative surgery was performed in 3 patients following a remarkable response to this treatment. The major grade 3/4 AEs were neutropenia (54.3%), leukopenia (37.1%), anemia (20.0%), febrile neutropenia (11.4%), and duodenal obstruction (8.6%). Ip-related AEs included port infection (8.6%) and obstruction (2.9%). All AEs were manageable and none were life-threatening.【Conclusions】 Intraperitoneal paclitaxel combined with gemcitabine plus nab-paclitaxel was safe and effective for pancreatic cancer with peritoneal metastasis. Further investigation through large-scale phase III trials is warranted.

### PO 25 Two-Stage Cytoreductive Surgery and Hyperthermic Intraperitoneal Chemotherapy for Peritoneal Surface Malignancies #PO 25

#### Oral communication or poster

G.L. Jazon^1^, M.P. Lopez^2^, M.G. Lim^3^, M.A. Zamora^4^

^1^Medical officer - General Surgery Resident - Manila (Philippines),

^2^Chief, Division of Colorectal Surgery - Manila (Philippines),

^3^Consultant, Division of Colorectal Surgery - Manila (Philippines),

^4^Fellow, Division of Colorectal Surgery - Manila (Philippines)

**Abstract**

**Purpose:** Cytoreductive surgery (CRS) with hyperthermic intraperitoneal chemotherapy (HIPEC) is an option for peritoneal surface malignancies (PSM), with outcomes relating to survival and quality of life. This is associated with increased morbidity that may impact on outcomes. We propose a two-staged approach—wherein CRS + HIPEC are done on separate dates.

**Methods:** Retrospective study was done who underwent a two-stage CRS-HIPEC from 2013 to 2023. Type of PSM, peritoneal carcinomatosis index (PCI), time interval to second surgery, operative times, length of stay and 30-day outcomes were obtained.

**Results:** Eleven patients underwent a two-stage CRS-HIPEC, with mean age of 53 years. The mean PCI was 11, with complete cytoreduction already achieved during the initial surgery in 7 (63%) patients—requiring only HIPEC on the second surgery. Further CRS, in the second surgery, was done prior to HIPEC in the other four.

The median duration of the first procedure was 366 minutes, the second was 340 minutes. Interval between the two surgeries was 6 days and the average total LOS was 16 days. No mortalities were reported.

**Conclusion:** The study showed that performing a two-stage procedure is safe with comparable oncologic and patient outcomes. We propose a wider use of a two-staged approach for CRS-HIPEC, not as bail-out procedure, but as a planned two-stage procedure—for patients with disease- and patient-related factors predictive of a potential volatile perioperative course. This strategy may benefit for complex peritoneal diseases with significant medical concerns.

### PO 26 Gastrointestinal Quality of Life after Cytoreductive Surgery and HIPEC – What is the real impact? #PO 26

#### Oral communication or poster

M.J. Madeira-Cardoso^1^, M. Peyroteo^1^, J. Mendes^2^, P. Pinto^1^, M. Marques^1^, A. Guimarães^1^, S. Alexandre^1^, F. Sousa^1^, M. Fernandes^1^, J. Abreu De Sousa^1^

^1^IPO Porto - Porto (Portugal), ^2^Centro Hospitalar do Médio Ave - Famalicão (Portugal)

**Abstract**

**Background:** Cytoreductive Surgery and HIPEC (CRS+HIPEC) is the only curative option for patients with peritoneal carcinomatosis (PC). CRS+HIPEC is a major procedure, consisting, many times, in a gastrointestinal (GI) resection. Both generic and GI quality of life (QoL) must be addressed as an outcome.

Objective: to analyze the impact of CRS+HIPEC in GI QoL.

**Methods:** Retrospective unicenter analysis of all patients submitted to CRS+HIPEC with curative intent between 2016 and 2020. Complete Cytorreduction Score > 1 was the only exclusion criteria. The Gastrointestinal QoL Index (GIQLI) was used to measure QoL. All surveys were performed by phone call.

The GIQLI total score varies from 0 to 144 (maximum score) and evaluates GI symptoms individually (ex. Abdominal pain, constipation, diarrhea, etc) in a scale from 0 to 4 (asymptomatic).

**Results:** 286 patients were submitted to CRS+HIPEC between 2016-2020 in our Center. 120 were alive at the time of the inquiries. 6 patients were excluded and 26 did not answered the phone. 88 patients (69% female; mean age 57±14 years) were included. 86% presented with ECOG 0 at the initial evaluation. 44% had the primary tumor located in the ileo-cecal appendix. Median PCI 11; IQR 39. 67% underwent GI resection (GIR).

Median GIQLI score 135; IQR 20. No/few GI symptoms were reported (median scores of 3 and 4 – asymptomatic). There was no difference in the prevalence of GI complaints between patients who were submitted to GIR (versus no GIR), except for abdominal pain, urgency, and diarrhea (p<0.005) – although more frequent among GIR, the complaints were mild.

**Conclusion:** CRS+HIPEC can significantly influence patients’ QoL apart from the known survival benefits. The GI impact of CRS+HIPEC is still unknown. In our study, CRS+HIPEC has apparently no negative impact on GI QoL, even after GIR. Prospective studies must be performed.

### PO 27 Assessment of the Efficacy of Chemotherapeutic Agents in Hyperthermic Intraperitoneal Chemotherapy (HIPEC) #PO 27

#### Poster

A.H. Mekkawy^1^, M.K. Rahman^1^, M. Breakeit^1^, K. Pillai^1^, S. Badar^1^, J. Akhter^1^, M. Dietz^2^, S. Barat^1^, S.J. Valle^1^, D.L. Morris^1^

^1^Mucpharm - Kogarah (Australia), ^2^Mucpharm - Rotterdam (Netherlands)

**Abstract**

**INTRODUCTION:** CRS-HIPEC is the mainstay treatment for peritoneal cancers. While CRS-HIPEC is often successful, some patients experience a rapid return of the cancer. Therefore, a method to identify any remaining viable tumor cells after treatment would be valuable. Flow cytometry, a technique used to analyze and differentiate cell populations, has the potential to serve this purpose. We propose investigating whether flow cytometry can be used to distinguish viable tumor cells following CRS-HIPEC treatment.

**METHODS:** A dedicated team performed CRS-HIPEC procedures according to local standards. During the procedure, peritoneal fluid was drained at three time points: at the beginning of surgery, before HIPEC perfusion, and after HIPEC. Cells were then concentrated from the fluid through a series of washes and centrifugation steps. Following in vitro culture, the cells were stained according to established protocols. Briefly, anti-tumor markers antibodies were utilized to gate for tumor cells. Annexin V versus Zombie dyes were employed to differentiate apoptotic and viable cells, respectively. Fluorescence minus one (FMO) control were used to define gate boundaries, and unstained cells served as negative controls. A Fortessa X20 flow cytometer was used for data collection.

**RESULTS:** This pilot study included five patients (appendix, peritoneum, gastric, colorectal, and peritoneum cancers, respectively) who were treated with CRS-HIPEC between October and December 2023. With this pilot study, we showed that the assessment of HIPEC drug efficacy on pre- and post-HIPEC samples is feasible. Data showed an increase of apoptotic cells by 3.9, 8.1, 12.8, 13.2, and 66.1%, respectively, after HIPEC treatment with MMC, Carboplatin, MMC & Cisplatin, MMC, and Cisplatin, respectively.

**CONCLUSIONS:** This pilot study will provide important information on the efficacy of HIPEC which is needed for the optimization of HIPEC and improvement of clinical outcomes. This technique also holds significant potential for research into new drugs and treatment regimens.

### PO 28 BromAc (Bromelain plus N-acetylcysteine) enhances Doxorubicin entry in ovarian and pancreatic cancer cells. #PO 28

#### Poster

M.K. Rahman^1^, A.H. Mekkawy^1^, K. Pillai^1^, M. Breakeit^1^, S. Badar^1^, J. Akhter^1^, S.J. Valle^1^, D.L. Morris^1^

^1^Mucpharm.Pty.Ltd - Sydney (Australia)

**Abstract**

**Introduction:** BromAc is the most potent mucolytic agent. The combination of BromAc and certain chemotherapeutics has a synergistic effect in a range of cancer cell line types. In this study we investigated one possible mechanism by which BromAc could sensitize cells to Doxorubicin.

**Methods:** Muc1-expressed human ovarian (SKOV-3 and CAOV-3) and pancreatic (PANC-1) cancer cells were first exposed to BromAc for 4 hours, then treated with Doxorubicin at various concentrations for 5-60 minutes. Then, the intracellular Doxorubicin concentration was measured using fluorescence spectrometry and microscopy. In another experiment, the viability was measured using SRB assay after 46 h of Doxorubicin treatment in post-BromAc treated cells.

**Results:** In PANC-1, 4 hours of BromAc exposure produced time-dependent Doxorubicin accumulation; BromAc increased the Doxorubicin expression in cultured intact cells from 5 to 15 minutes compared to the control. In CAOV-3, the concentration of intracellular Doxorubicin increased gradually with the rise of BromAc (0.001/0.5mg - 1/500mg) concentration; after exposure to BromAc (1/500mg), the uptake of Doxorubicin in the cells increased nearly 2-fold compared to cells treated with only Doxorubicin (p<0.05). However, in SKOV-3, BromAc enhanced cellular uptake of Doxorubicin to a varying extent with the changes in the concentration of BromAc.

The cell viability assay also corroborated our initial findings for CAOV-3; 4 hours of treatment with BromAc significantly improved the cell-killing effect of Doxorubicin compared to its treatment alone. In CAOV-3, the IC50 of Doxorubicin was 319 nM, but with pre-exposure to BromAc (0.01/5mg), the IC50 was reduced to 123 nM. BromAc also increases the sensitivity of PANC-1 and SKOV-3 to chemotherapeutics.

**Conclusion:** Taken together, the current findings suggest that pre-treatment with BromAc enhances intracellular chemotherapeutic delivery in pancreatic and ovarian cancer cell lines, which may be the cause of increasing sensitivity to chemotherapy.

### PO 29 Intraperitoneal chemotherapy improves 5-year survival for colon cancer #PO 29

#### Oral communication

S. Berkane^1^

^1^Faculté De Médecine D’alger. Service De Chirurgie Viscérale Et Oncologique. Hôpital De Bologhine. Ibn Ziri. Alger - Algiers (Algeria)

**Abstract**

**Summary:** Systemic chemotherapy (CS) has generally improved the survival of colon cancer (CC) and in particular stages III and IV. Does the addition of intraperitoneal chemotherapy (IPC) to radical surgery further improve results compared to CS alone? The objective of this study is to analyze the results in terms of locoregional and distant recurrences and survival after addition of IPC to CS after radical CC surgery. **Material and method:** all patients who underwent radical surgery associated with IPC or CS were included and divided into 2 groups. A group A: patients with CIP and group B: patients with CS. The following parameters were studied: age, sex, defect, TNM, metastatic sites, tumor markers, peritoneal cytology, 1- or 2-stage surgery, tumor recurrence, 5-year survival. **Results:** Out of a total of 705 patients, 227 met the inclusion criteria (32.2%). Groups A and B consist of 75 and 152 cases respectively. There was 18.7% (14/75) of recurrence in group A compared to 49.3% (75/152) for group B (p<0.001). Peritoneal and hepatic recurrences are significantly lower in group A compared to group B with respectively 05.3% (04/75) vs 18.4% (28/152) and 05% (04/75) vs 30% (41/152). Overall survival at 5 years is significantly higher for group A with 81% (61/75) vs. 57.2% (87/152) for group B (p<0.001). Likewise, a significantly better 5-year recurrence-free survival was noted in favor of group A, 81% (61/75) vs 52.3% (81/152) (p<0.001). **Conclusion:** our results provide an argument strongly in favor of the association of IPC with CS compared to CS alone after radical CC surgery. This association significantly increases overall survival at 5 years through a reduction in recurrence of peritoneal carcinomatosis and the occurrence of liver metastases.

### PO 31 Impact of resected peritoneal surface area and risk factors on acute kidney injury following CRS and cisplatin-based HIPEC. #PO 31

#### Oral communication or poster

P. Schredl^1^, J.P. Ramspott^2^, M. Weitzendorfer^1^, L. Kaiser^1^, J. Presl^1^, P. Tschann^3^, N. Rodemund^4^, D. Neureiter^5^, K. Emmanuel^1^, T. Jäger^1^

^1^Department of Surgery, Paracelsus Medical University Salzburg - Salzburg (Austria),

^2^Department for General, Visceral and Transplant Surgery, University Hospital Muenster - Muenster (Germany),

^3^Department of General and Thoracic Surgery, Academic Teaching Hospital Feldkirch - Feldkirch (Austria),

^4^Department of Anesthesiology, Perioperative Medicine and Intensive Care Medicine, Paracelsus Medical University Salzburg - Salzburg (Austria),

^5^Institute of Pathology, Paracelsus Medical University Salzburg - Salzburg (Austria)

**Abstract**

**Introduction:** Cytoreductive surgery (CRS) with hyperthermic intraperitoneal chemotherapy (HIPEC) using cisplatin may elevate the risk of postoperative kidney damage. The incidence is reported to range from 1% to 66%. The impact of the resected peritoneal surface on postoperative kidney function remains uncertain.

**Methods:** From a prospective HIPEC database, 63 patients who received cisplatin-based HIPEC between 2011 and 2022 were extracted. Clinical risk factors and their influence on postoperative kidney function were evaluated using univariate analysis. The resected and remaining peritoneal surface was calculated using the ’Salzburg Peritoneal Surface Calculator’ (SAPESUCA) software.

**Results:** The predominant tumor entities were ovarian cancer (51%), gastric cancer (28%), and mesothelioma (12%). The HIPEC was performed closed for 60 minutes at 41°C, with 75mg cisplatin/m^2^ body surface area (BSA), 15mg doxorubicin/m^2^ BSA. No periinterventional nephroprotection was provided in any of the cases. The median Peritoneal Cancer Index (PCI) score was 12 (range 1-31). Complete cytoreduction (CC0) was achieved in 86% of the cases. Postoperative acute kidney injury (AKIN) was observed in 22 patients (34.9%). Regarding the occurrence of postoperative kidney damage, neither age, PCI, nor tumor entity could be identified as risk factors. A positive correlation existed with the extent of resected visceral surface area (p=0.002).

**Conclusion:** In our patient cohort, AKIN occurs in 34.9% of cases following CRS and cisplatin-based HIPEC. This retrospective analysis demonstrated that the extent of peritonectomy is an independent risk factor for the occurrence of postoperative kidney damage and needs further validation.

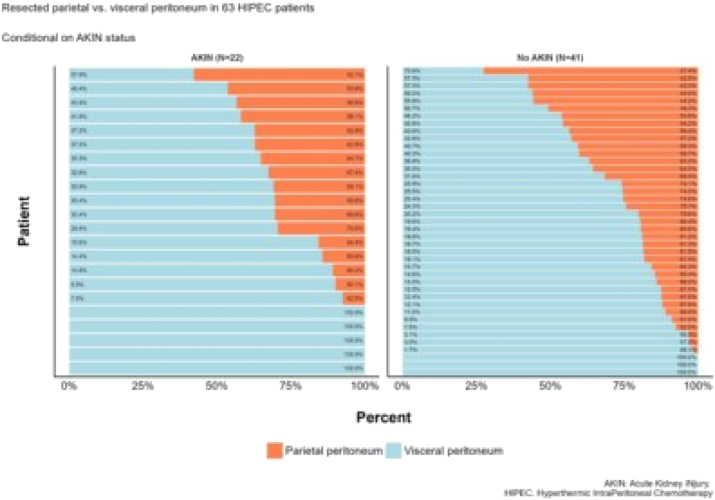



### PO 32 Data completeness within the International Society for the Study of Pleura and Peritoneum (ISSPP) PIPAC database #PO 32

#### Oral communication or poster

M.S. Jørgensen^1^

^1^Odense PIPAC Center, department of surgery, OUH, Denmark - Odense M (Denmark)

**Abstract**

**Background:** In 2020 PIPAC directed therapy reached stage 2b of the IDEAL recommendations. In this context a prospective international PIPAC database was launched in June 2020 by the International Society for the Study of the Pleura and Peritoneum (ISSPP). The ISSPP PIPAC database consists of six key elements (Patient, Consent, Treatment, Complications, Response evaluation and Follow-up) which are reported in the annual database report.

We investigated data completeness within the ISSPP PIPAC Database in order to provide a status and - if necessary, an improved strategy for data input and reporting.

**Methods:** A review of data completeness within each of the reporting centers was performed on March 1, 2024.

**Results:** Twenty-seven centers, 949 patients and 2455 PIPAC procedures were registered in the ISSPP database, but only 14 centers have included patients. Missing and incomplete data were observed in all six key elements except Consent. Most centers (10/14) had missing and/or incomplete Complications data, followed by Response evaluation (5/14), and Follow-up (4/14). Complication date was noted in 87% of all procedures and a similar rate was reported regarding type of complication. Dindo-Clavien grading was provided in 85%. Response evaluation was not performed in 353 procedures. PRGS- or non-PRGS response evaluation was missing in 230/2102 procedures (11%). Regarding follow-up, date of death was provided in 86% of the patients. Reasons for stopping PIPAC were available in 85%. In 16% of the patients where “other” reasons were assigned, this was not specified in details.

**Conclusion:** All major multi-center databases must accept incomplete data in the beginning, and this was confirmed for the majority of key elements within the ISSPP PIPAC Database. We will launch an improved and individual support program for active and new PIPAC centers to enrich future data sets.

### PO 33 Nephrotoxicity associated to cytoreductive surgery combined with cisplatin-based HIPEC for peritoneal malignant disease: systematic review and meta-analysissis #PO 33

#### Oral communication or poster

C. Grillo Marín^1^, C. Antón Rodríguez^2^, L. Prieto López^2^, G. Ortega Pérez^3^, S. González Moreno^3^

^1^Puerta de Hierro University Hospital - Majadahonda (Spain),

^2^Francisco de Vitoria University - Pozuelo de Alarcón (Spain),

^3^MD Anderson Cancer Center Madrid - Madrid (Spain)

**Abstract**

**Objective:** Cisplatin is frequently used in hyperthermic intraperitoneal chemotherapy (HIPEC) following cytoreductive surgery (CRS) for peritoneal carcinomatosis treatment of various origins. The main concern with intraperitoneal cisplatin administration is nephrotoxicity. Several reports exist on this topic with varying renal insufficiency assessment scales and incidences. Our objective is to conduct a systematic review and meta-analysis on cisplatin-related nephrotoxicity after HIPEC employing this drug.

**Methods:** A systematic literature review on cisplatin-based HIPEC following CRS for peritoneal carcinomatosis treatment was performed. Literature search utilized Medline, Cochrane, and Embase databases. Last search was conducted on October 23, 2023. PRISMA guidelines were used. The primary endpoint is the incidence of acute and chronic renal impairment post-cisplatin-based HIPEC with or without concurrent nephroprotective agents. Secondary endpoints include assessing the potential impact of various clinical variables on the primary outcome and critically appraising the different renal impairment scales utilized.

**Results:** This study includes 26 articles with a total sample of 1473 patients. Obtained results reflect an incidence of acute kidney injury (AKI) of 18.6% (95% CI: 13.6% – 25%) with a wide range of true effects of 3% - 59%. The incidence of chronic kidney disease is 7% (95% CI: 3% - 15.3%). With a wide range of true effects of 1% - 53%. The variables that statistically significantly influence these results are the scale used to measure renal insufficiency, as well as the use of nephroprotective agents and the presence of pre-existing renal disease. The rest of the analyzed variables did not show a relationship with the primary outcome.

**Conclusion:** The incidence of renal impairment following cisplatin-based HIPEC displays significant variability. Nephroprotective agent usage appears to aid in preventing cisplatin-induced renal failure. Reported renal failure incidences should serve as reference points for subsequent reports. Further prospective studies are warranted to establish optimal and standardized management protocols.

### PO 34 Presurized Intraperitoneal Aerosol Chemotherapy (PIPAC) in Urachal Adenocarcinomas: A multimodal treatment. #PO 34

#### Oral communication

X. Delgadillo^1^, J. Sandoval^2^, O. Misad^3^, C.A. Sedano^2^, H. Auris^2^, I.B. Qentasi^2^

^1^Centre Médico Chirurgical Volta - La Chaux-de-Fonds (Switzerland), ^2^Detecta Clinica - Lima (Peru), ^3^INEN - Surquillo - Lima (Peru)

**Abstract**

**Background:** Urachus, an allantois-cloaca embryonic remnant, broads intra-extra peritoneally, from the umbilicus to the bladder dome. Urachal Adenocarcinoma (UAC) an uncommon lesion (<0.05%) of all bladder neoplasias, sex ratio M1,6:1F, incidence between 50 to 60’s. *UAC* is rarelly diagnosed in early stages, when survival rate is ∼10 yr. In stage 4 survival rate is <1 yr. Surgery remains gold standard treatment as *UAC* is not radiosensitive. I.V. chemotherapy remains challenging because of limited vascularization of peritoneal metastasis. **Methods:** PIPAC emerges as a novel method used in the case of 61-y.o. patient, presenting lower back stabbing pain abdominally referred, unresponsive to painkillers. An abdominal MRI (***Picture 1.*** a.b.) showed an urachal tumor associated to important peritoneal dropsy. Immediate laparoscopy, complete urachal excision & total ascites drainage were performed (***Picture 2.***). Histopathology confirmed a very-low grade well-differentiated *UAC* glandular-type (***Picture 3.*** a.b.). Adjuvantly 1^st^ line i.v. chemotherapy of CIS+ 5-FU was initiated. After 6 weeks a 1^st^ PIPAC (CIS 30.0 mg/m^2^ + DOXO 6.0 mg/m^2^) was performed through a standard nebulizer (Capnopen®), Sugarbaker’s PCI was 14. **Results:** During 2^nd^-3^rd^ PIPAC the PRGS was twice 2/2, demonstrating *PM* regression. Radiological response was assessed by CT-Scan RECIST Score (***Picture 4.*** a.b.), letting us to schedule a further HIPEC after 6 weeks. **Conclusions:** PIPAC optimized peritoneal pharmacokinetics-biodistribution of chemotherapy not yet reported in *UAC* tumours. HIPEC remains the formal indication for overall survival improvement. We describe the 1^st^ PIPAC “*bridge*” in *UAC’s* multimodal PM treatment, with good tolerance, no toxicity & very good QoL.

MRI diagnosis, PIPAC procedure & PRGS evaluation.

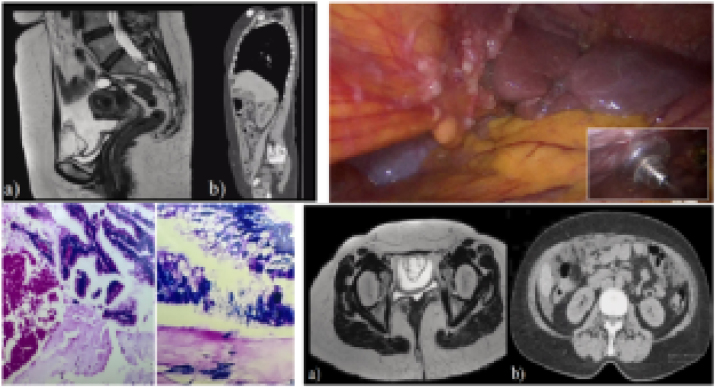



### PO 36 Effects of electrostatic precipitation during intraperitoneal aerosolized drug delivery: results from a realistic in vitro model #PO 36

#### Oral communication or poster

M. Rahimi-Gorji^1^, Y. Long^1^, C. Debbaut^2^, W. Willaert^1^, W. Ceelen^1^

^1^Laboratory of Experimental Surgery, Department of Human Structure and Repair, Ghent University - Ghent (Belgium),

^2^IBiTech – BioMMedA, Ghent University - Ghent (Belgium)

**Abstract**

**INTRODUCTION:** Intraperitoneal Aerosolized Drug Delivery is a novel treatment for patients with peritoneal metastases. The treatment consists of CO2 insufflation followed by aerosolization of anticancer drugs. This treatment results in a heterogeneous droplet distribution and limited tissue penetration. By adding electrostatic precipitation (EP), it may be possible to improve the homogeneity and penetration. In this study, we investigate experimentally droplet distribution and tissue penetration in a realistic in vitro model.

**METHODS:** In vitro experiments were performed (triplicate) using an aluminum model based on a CT scan of a patient (Fig. 1-a). A volume of 170 mL of methylene blue was nebulized. To enhance visualization of aerosol distribution, white tissue sheets were utilized to cover the model’s top surface. The electrical field was generated using a power supply (Alesi Surgical). After each experiment, the ventral surface was photographed to calculate the stained proportion. Fresh human normal peritoneum tissue samples were positioned at different sites in the model to allow quantification of penetration.

**RESULTS:** Fig. 1 illustrates the in vitro distribution of aerosols on the ventral surface. The addition of EP dramatically improved the spatial distribution of the aerosol, as evidenced by the extent of staining of the white tissue sheets (11.20% vs. 77.5%, Fig. 1-b). After cryo-sectioning and imaging of tissue samples, a significantly deeper tissue penetration of methylene blue by EP addition was observed (Fig. 1-c).

**CONCLUSIONS:** EP applied in a patient-based in vitro model of the peritoneal cavity shows a more homogeneous distribution and deeper tissue penetration of droplets.

Figure 1

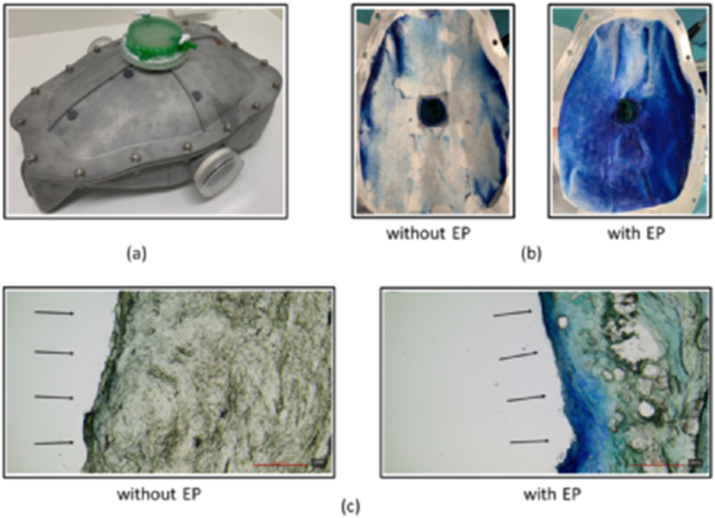



### PO 37 Collaborative Pressurized IntraPeritoneal Aerosol Chemotherapy (PIPAC) – Latin American (LATAM) Group: Initial experience & settings. #PO 37

#### Oral communication

X. Delgadillo^1^, L. Lay^2^, F. Perrota^3^, J. Sandoval^4^, R. Seitenfuss^5^, M. Uribe^6^

^1^Centre Médico Chirurgical Volta - La Chaux-de-Fonds (Switzerland),

^2^Instituto de Oncologia Roffo - Buenos Aires (Argentina),

^3^Oncologia Quirurgica Universidad Nacional de Asuncion - Asuncion (Paraguay),

^4^Detecta Clinica del Hospital Nacional Almenara-Irigoyen - Lima (Peru),

^5^Santa Rita Hospital da Santa Casa de Misericordia - Porto Alegre (Brazil),

^6^Clinica Meds. La Dehesa - Santiago (Chile)

**Abstract**

**Background.** PIPAC is a recent approach for intraperitoneal chemotherapy with promising results. We evaluate our initial experience, safety-feasibility and outcomes of PIPAC procedure in LATAM countries. **Methods.** The Collaborative PIPAC-LATAM Group, was founded in Huntington, L.A. (USA) during the 3^rd^ ISSPP International Congress held in 2022. Co-founder countries (Argentina, Bolivia, Brazil, Chile, Paraguay, Peru), collected data prospectively. Nevertheless, as PIPAC was available before October 2022, in some countries (Argentina/ Brazil), retrospective data harvesting has been obtained. **Results.** From 2017 to 2024, eleven procedures were performed with a classic nebulizer (Capnopen®) in Buenos Aires (36,4% ovarian, 45,5% digestive, 18,2% peritoneal’s). Brazil, had performed almost hundred PIPAC procedures since 2018, applying a native nebulizer (BioQuapp®) actually discontinued. Since 2022, around 15 different brazilian surgical teams perform PIPAC with a variety of up-coming devices. Paraguay has performed 4 PIPAC procedures using two different nebulizers (3-Capnopen® gastric /1-Regger® mesothelioma). A single PIPAC for ovarian PM has been performed successfully in Bolivia ( 2021) using Capnopen® device. Chile started PIPAC in 2021, performing 13 cases (30,8% colorectal, 23,1% upper GI, 23,1% gyneacological, 15,4% pseudomyxoma) using standard nebulizer. Patients with PM from various primary sites underwent 12 PIPAC procedures in Peru, nebulizing standard drug regimens but using 4 different devices. LATAM over all median hospital stay was 4,2 days, complications 1, 8%, mortality 0, 9%. HIPEC after PIPAC was performed in 19,9%. Colombia/Ecuador are pending on importation rules. **Conclusions.** We corroborate the feasibility, safety of PIPAC-LATAM, with very low morbidity-mortality, short hospital stay & good QoL.

General PIPAC LATAM Results

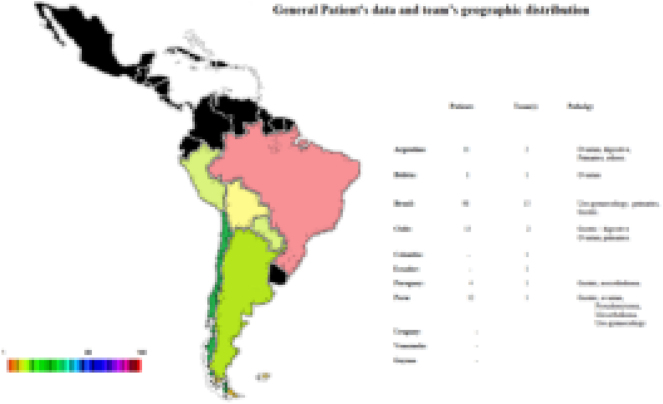



### PO 72 Neo-adjuvant intraperitoneal chemotherapy combined with systemic therapy prior to CRS and HIPEC for patients with resectable colorectal peritoneal metastases: Protocol of the multicenter, phase II, INTERACT-PLUS trial #PO 72

#### Oral communication or poster

J. Hamm^1^, L. Galanos^2^, P. Tanis^1^, C. Verhoef^1^, R. Mathijssen^1^, I. Hellemond^2^, J. Tuynman^3^, N. Kok^4^, I. De Hingh^2^, E. Madsen^1^

^1^Erasmus University Medical Center - Rotterdam (Netherlands),

^2^Catharina Hospital Eindhoven - Eindhoven (Netherlands),

^3^Amsterdam University Medical Center - Amsterdam (Netherlands),

^4^Netherlands Cancer Institute - Amsterdam (Netherlands)

**Abstract**

**Introduction:** The peritoneum is one of the most common metastatic sites in colorectal cancer patients. For patients with limited peritoneal metastases (PCI≤20), the only option with curative intent is cytoreductive surgery with hyperthermic intraperitoneal chemotherapy (CRS-HIPEC). Although CRS-HIPEC offers long-term survival and even curation for some, recurrence in the peritoneal cavity is common. It can be argued that the single administration of intraperitoneal (IP) chemotherapy, during CRS-HIPEC, might not be sufficient to eradicate intraperitoneal cancer cells. Previous research suggests that patients with peritoneal metastases (PM) may benefit from additional IP chemotherapy prior to CRS-HIPEC. Therefore, we designed this phase 2 trial investigating the feasibility of IP chemotherapy (irinotecan) in addition to systemic chemotherapy (mFOLFOX4- bevacizumab) prior to CRS-HIPEC in patients with resectable PM of colorectal origin.

**The secondary endpoints:** The secondary endpoints of this trial are histopathological and radiological treatment response, safety, the potential value of PET-CT in treatment response monitoring, quality of life (EQ-5D-5L, QLQ-C30, QLQ-CR29, iMTA productivity cost questionnaire, iMTA medical consumption questionnaire) and disease-free survival, which is assessed until three months after CRS-HIPEC.

**Methods:** The INTERACT-PLUS is a phase II, single-arm trial, conducted in four expert centers. The study population, comprising of 40 patients with limited, macroscopic PM, receive intravenous and IP treatment prior to CRS and HIPEC. According to standard of care in the Netherlands, patients undergo a diagnostic laparoscopy to evaluate the PCI-score. In case of a PCI-score between 1-20, a peritoneal access port for IP administration of irinotecan is placed. Simultaneously with IP irinotecan, patients receive intravenous 5-fluorouracil/leucovorin with oxaliplatin (mFOLFOX4) and bevacizumab. Depending on response and tolerability, patients receive a maximum of six cycles, the last two without bevacizumab.

**Trial registration and approval:** Submission to the Medical Research Ethics Committee (Rotterdam, the Netherlands) and the Clinical Trials Information System (CTIS) is currently ongoing.

### PO 73 Influence of splenectomy in patients with peritoneal metastases following cytoreductive surgery and hyperthermic intraperitoneal chemotherapy on postoperative complications, recurrence and survival. #PO 73

#### Poster

M. Rashed^1^, W. Graf^1^, M. Enblad^1^, P. Cashin^1^, H. Birgisson^1^, L. Ghanipour^1^

^1^Department of Surgical Sciences, Uppsala University - Uppsala (Sweden)

**Abstract**

**Background:** Cytoreductive surgery and hyperthermic intraperitoneal chemotherapy (CRS-HIPEC) is standard treatment for peritoneal metastases of various origins. Peritoneal metastases often involve the spleen, making splenectomy a common procedure in CRS-HIPEC. This study investigates postoperative complications and prognosis after splenectomy following CRS-HIPEC.

**Methods and patients:** Data from patients undergoing CRS-HIPEC during the period 2012-2022 was collected from a prospectively maintained HIPEC registry. Information about surgical complications related to splenectomy, overall survival and pattern of recurrence were registered. Results were then compared between splenectomised and non-splenectomised patients.

**Results:** In total, 437 patients underwent CRS-HIPEC or CRS during the study period, 118 patients were splenectomised and 319 were not. Median PCI was 26 in the splenectomy group and 8 in the control group (p<0.001). Local recurrence was more common in the left upper quadrant (p=0.005) and more grade 3-4 complications occurred compared to the control group (p=0.017). Patients undergoing a pancreas resection along with splenectomy (n=10) had a higher rate of postoperative complications such as amylase leakage and bleeding (p=0.004). The only factor independently associated with shorter time to recurrence was malignancy in the spleen specimen, HR 3.06 (1.05-8.95). In the univariate analysis, splenectomy was associated with negative overall survival HR1.56 (1.04-2.35) but not in the multivariate analysis.

**Conclusion:** Splenectomy during a CRS-HIPEC procedure does not increase the risk of postoperative complications such as amylase leakage and bleeding unless a simultaneous pancreatic resection is performed. Worse outcome in terms of survival and relapse after splenectomy may reflect a more advanced peritoneal tumor growth.

### PO 74 Phase 1 study of Pressurized IntraPeritoneal Aerosol Chemotherapy (PIPAC) and Electrostatic PIPAC (ePIPAC) with Paclitaxel for Peritoneal Carcinomatosis – PIPAC2 Study #PO 74

#### Oral communication or poster

R.J. Walsh^1^, D. Chia^2^, R. Sundar^3^, C. Yap^2^, A. Pang^2^, H.L. Tan^3^, A. Shabbir^2^, G. Kim^2^, W.P. Yong^3^, J.B.Y. So^2^

^1^1. Department of Haematology-Oncology, National University Cancer Institute, Singapore - Singapore (Singapore),

^2^2. Division of General Surgery (Upper Gastrointestinal Surgery), University Surgical Cluster, National University Hospital, Singapore; Division of Surgical Oncology (Upper Gastrointestinal Surgery), National University Cancer Institute, Singapore - Singapore (Singapore),

^3^1. Department of Haematology-Oncology, National University Cancer Institute, Singapore. - Singapore (Singapore)

**Abstract**

**Background:** Peritoneal metastases (PM) in solid organ malignancies carry a poor prognosis with limited treatment options. Response to standard systemic therapy is often disappointing, contributed in part by limited drug delivery to the peritoneum. Intraperitoneal (IP) drug delivery is therefore an attractive option with pressurised intraperitoneal aerosol chemotherapy (PIPAC) one such approach. Studies have suggested PIPAC enhances drug delivery over standard IP administration with high local, and low systemic drug concentrations reported. This may be further enhanced by adding electrostatic precipitation in a process termed ePIPAC. Here we study PIPAC/ePIPAC with paclitaxel in a prospective 2 arm study of patients with PM in a phase 1 3+3 dose escalation protocol. Interim analysis is presented.

**Methods:** Eligible patients with biopsy/cytology proven PM from a solid organ malignancy and progression after >/= 1 line of systemic therapy are enrolled to either the PIPAC or ePIPAC study arms. Patients with predominant extra-peritoneal disease were excluded. Patients undergo PIPAC/ePIPAC using paclitaxel at a 6 week interval. The dose of paclitaxel will be escalated from 15 to 60mg/m2 using a 3+3 design to determine maximum tolerated dose (MTD). Enrolment will begin with dose level (DL) 1 in the PIPAC arm. Should there be no dose limiting toxicity, recruitment at PIPAC 30mg/m2 and ePIPAC 15mg/m2 will occur concurrently in an alternating fashion. Pre- and post-treatment biopsies will be obtained for exploratory translational analysis.

**Results:** Trial recruitment is currently ongoing at DL2 (30mg/m2) for PIPAC and DL1 (15mg/m2) for ePIPAC. Therapy has been well tolerated with no related grade 3 or higher adverse events observed. Further outcome results will be presented.

**Conclusion:** Interim results of PIPAC/ePIPAC with paclitaxel suggest it to be feasible and well tolerated. Ongoing dose escalation aims to establish a MTD and recommended phase 2 dose.

### PO 75 Comprehensive picture of colorectal peritoneal metastasis- a single center experience #PO 75

#### Oral communication

V. Kesseler^1^, H. Teixeira Farinha^1^, M. Hübner^1^, C. Sempoux^1^, W. Solass^1^

^1^CHUV - Lausanne (Switzerland)

**Abstract**

**Introduction:** Metastatic colorectal cancer (CRC) is a significant cause of cancer-related deaths worldwide, often involving peritoneal metastases (CRC-PM). A new sequential intraperitoneal treatment-regimen allows for the first time in the history of CRC-PM to observe in real-time the effect of chemotherapeutics on tissue/tumor. This study aims to gather a comprehensive dataset of CRC-PM to enhance our understanding of this disease.

**Materials and Methods:** This retrospective cohort study was conducted at a single center (CHUV, Lausanne, Switzerland), including patients with CRC-PM treated with pressurized intraperitoneal aerosol chemotherapy (PIPAC) between 2015 and 2023. Data analysis combined morphological, molecular, and clinical features.

**Results:** The study comprised 45 patients with CRC-PM (23 males, 22 females), averaging 59 years old (range: 24 to 88 years). Primary tumor localization was evenly distributed with 22 cases right-sided (including 9 caecum, 6 transvers, and 2 appendix), and 23 left-sided (including 17 sigmoid and 3 rectum). Most women (59%) had left-sided CRC, while most men (56.5%) had right-sided CRC. Most cases were advanced (66% T4), with predominantly asynchronous metastases (66%). Mutations differed between right and left-sided CRC: KRAS (45.5% vs. 39%), MSI (22% vs. 17%), BRAF (13% vs. 4.3%); no mutations found (18% vs. 34.7%). A total of 84 PIPAC procedures were performed (average: 1.86 per patient, 3.90 biopsy per surgery). The average Peritoneal Cancer Index was 11.68 (range: 0 to 39), and the mean Peritoneal Regression Grading Score (PRGS) among patients who received multiple PIPACs was 2.05, showing a predominance of decreased PRGS between treatments (48% decreased, 26% increased, 26% no change).

**Conclusion:** This study compares right and left-sided CRC in CRC-PM, suggesting a positive response to PIPAC therapy, and highlighting its efficacy in achieving local disease control. CRC-PM remains heterogeneous, necessitating further research to gain a deeper understanding of the mechanisms underlying treatment response and resistance.

### PO 76 Phase I Trial of Intra-tumoral Lipopolysaccharide Injection in Peritoneal Tumors #PO 76

#### Oral communication

P. Wagner^1^, C. Lewis^1^, N. Dadgar^2^, H.Y. Park^1^, A. Omstead^1^, K.K. Xiao^1^, A. Zaidi^1^, A. Donnenberg^1^, V. Donnenberg^3^, D. Bartlett^3^

^1^Allegheny Health Network Cancer Institute - Pittsburgh (United States),

^2^Cleveland Clinic Foundation - Cleveland (United States),

^3^University of Pittsburgh - Pittsburgh (United States)

**Abstract**

**Background:** Intra-tumoral immunotherapy has emerged as a promising approach for treating advanced cancers. However, delivery challenges have restricted its exploration in intra-peritoneal tumors. We investigated the safety of intra-peritoneal tumor injection with Escherichia coli 0113-derived lipopolysaccharide (LPS), which stimulates immune cells through the toll-like receptor 4 (TLR4).

**Methods:** The Regional Immuno-Oncology Trial-1 (RIOT-1; NCT05751837) was a Phase I trial in which patients with peritoneal metastases from gastrointestinal malignancies underwent diagnostic laparoscopy, during which a single 1 μg dose of LPS was administered into a representative tumor, with saline injected into a second lesion as control. The primary outcome was safety of intra-abdominal tumor injection with LPS. Injected tumors were harvested during laparotomy 14 days post-injection, for biomarker assay of the tumor immune microenvironment.

**Results:** Intra-tumoral LPS and saline injection was feasible and safe in 12 enrolled patients, with no study-related adverse event observed in any patient. Immune biomarker analysis indicated a reduction in CD8 count in LPS-injected tumors (mean 556 vs. 291, p<0.0001), but not in saline (control)-injected tumors. CD3 counts did not change with injection of either LPS or saline.

**Conclusion:** Intra-peritoneal, intra-tumoral injection of the TLR4 agonist LPS was feasible and safe in this Phase I study, and was associated with a ∼50% reduction in the CD8 lymphocyte count within the tumor immune microenvironment-- a finding that will require further systematic characterization. Harvesting of injected tumors at subsequent laparotomy represents a window-of-opportunity concept to assess the promise of laparoscopically injected immunotherapeutic agents in peritoneal surface malignancies.

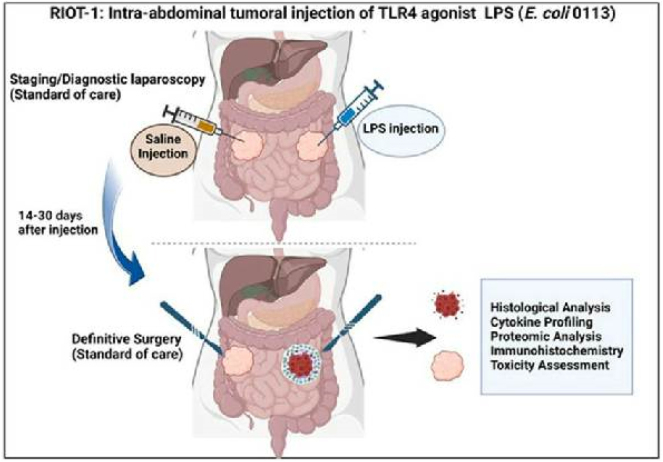



### PO 77 Intra-cavitary Tocilizumab Immunotherapy for Malignant Pleural Effusions and Ascites: Phase I Protocol Rationale and Preliminary Results #PO 77

#### Oral communication

P. Wagner^1^, V. Donnenberg^2^, H.Y. Park^1^, C. Lewis^1^, N. Dadgar^3^, K.K. Xiao^1^, A. Zaidi^1^, D. Bartlett^1^, A. Donnenberg^1^

^1^Allegheny Health Network Cancer Institute - Pittsburgh (United States),

^2^University of Pittsburgh - Pittsburgh (United States),

^3^Cleveland Clinic Foundation - Cleveland (United States)

**Abstract**

**Background:** Malignant ascites (MA) and pleural effusion (MPE) are late complications of advanced cancer with limited treatment options. Based on previous literature implicating the cytokine IL-6 as a central mediator in MA/MPE, the Regional Immuno-Oncology Trial-2 (RIOT-2) clinical protocol was developed to explore intra-cavitary delivery of the IL-6-receptor antagonist tocilizumab, as treatment for these conditions.

**Methods:** This phase I trial (NCT 06016179) is will assess the safety and pharmacokinetics of intra-cavitary tocilizumab administration to patients with MA/MPE. Eligible patients with MA/MPE undergo standard-of-care drainage catheter placement, followed by a starting dose of tocilizumab 0.5 µg/mL with dose escalation over four weeks. Primary endpoints are type/frequency of adverse events, with secondary pharmacokinetic and immunological endpoints.

**Results:** Two of the intended twelve patients have completed the ongoing study without study-related adverse events. Treated malignant ascites fluid contained peritoneal immune cells, mesothelial cells, and proliferating tumor, with cell analysis demonstrating polyfunctionality (IFNγ (83%) and IL-2 (2.4%) secretion) by expanded CD8+ T cells, and upregulated granzyme B and 4-1BB expression. Almost all tumor cells exhibited epithelial-mesenchymal transition (EMT; CTK+CD90+CD44+Vim+sECAD+sEpCAM-low). The fluid and plasma secretome, tocilizumab PK, and TCR and BCR repertoire will be determined in batch at study conclusion.

**Discussion:** IL-6 inhibition with intra-peritoneal tocilizumab is a rational mitigating treatment strategy for MA/MPE. The RIOT-2 study aims to assess the safety of intra-cavitary tocilizumab administration via indwelling catheters. In preliminary results, infiltrating T cells were polyfunctional, supporting their use in adoptive cellular therapy. Comprehensive translational biomarker analysis is planned at study conclusion.

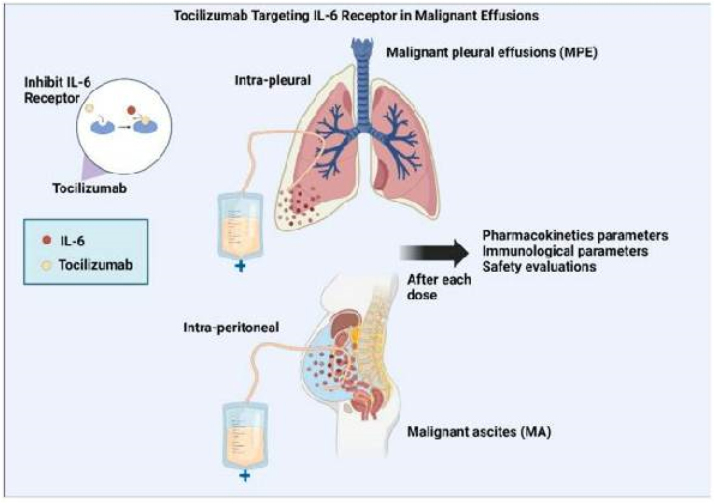



### PO 78 Pushing the Limits of Oncological Treatment in Elderly Patients: Results of an Aggressive Policy for Peritoneal Carcinomatosis Treated by Cytoreductive Surgery and HIPEC #PO 78

#### Oral communication

D. Benchimol^1^, R. Orgad^2^, S. Dolnikov^1^, M. Saleh^1^, D. Peleg^1^, L. Cooper^3^, H. Kashtan^2^, N. Wasserberg^1^

^1^Department of Surgery, Rabin Medical Center, Beilinson Hospital - Petah Tikvah (Israel),

^2^Department of Surgery, Samson Assuta Ashdod University Hospital - Ashdod (Israel),

^3^Department of Geriatric Medicine, Rabin Medical Center, Beilinson Hospital - Petah Tikvah (Israel)

**Abstract**

1. Peritoneal carcinomatosis (PC) has a dismal prognosis. Cytoreductive surgery and hyperthermic intraoperative chemotherapy (CRS+HIPEC) has improved survival with a high rate of perioperative mortality and morbidity. This study aims to assess outcomes of CRS+HIPEC for PC in adults older than 70 yo (OA).

2. A retrospective analysis of prospectively maintained database of patients operated for PC between June 2016 and January 2024, at a large-volume academic center.

The cohort was divided into two groups according to age (above and below 70 years). Baseline characteristics, oncological, and surgical variables were collected and compared between the groups. Statistical analysis was performed using Statistical Analysis Software (SAS) and a p<0.05 was considered significant.

3. Study cohort included 225 patients with an age range of 25-91 yo, of which 63 (28%) were ≥ 70 yo and 162 (72%) were < 70 yo. More patients in the OA group had cardiovascular co-morbidities (72% vs 44% p<.01), although there was no difference in American Society of Anesthesiology (ASA) score (p=0.4). Indication for surgery in both groups was peritoneal metastases of colorectal (>60%), gastric (8.3%) and ovarian (8.3%) malignancies.

Complete CRS+HIPEC was achieved in most patients in both groups (85% vs 84% p=0.9). Both groups had a similar Peritoneal Cancer Index (PCI) (8.6 vs 9.2 p=0.6) and in most cases completeness of cytoreduction (CC) score was 0 (74.2% vs 67.5% p=0.6). There were two perioperative deaths, one in each group. Complication rate was similar in both groups (42% vs 45% p=0.9). The rate of severe complications (Clavien-Dindo grade ≥3) was lower in the OA group (21% vs 45%) but this was not statistically significant (p=0.1). Survival was similar between the groups with an estimated 5-year survival rate higher than 40%.

4. Older adults, when carefully selected, can safely undergo CRS+HIPEC with comparable short and long-term outcomes.

### PO 79 The outcome of patients scheduled for CRS and HIPEC while only undergoing explorative laparotomy #PO 79

#### Oral communication or poster

V.V. Valdimarsson^1^, I.S. Syk^1^, V.V. Verwaal^1^

^1^Lund University - Lund (Sweden)

**Abstract**

**Background:** Some patients who are scheduled for cytoreductive surgery (CRS) and hyperthermic intraperitoneal chemotherapy (HIPEC) only undergo explorative laparotomy (open/close procedure) due to too extensive tumor spread (indicated by high Peritoneal Cancer Index (PCI)) or challenges in achieving complete cytoreduction (CC). This study aims to evaluate the surgical outcomes and overall survival of patients with peritoneal surface malignancy (PSM) who only undergo open/closed laparotomy.1

**Methods:** All patients scheduled for CRS and HIPEC in Malmö, Sweden between 2015-2023 but only undergoing open/close laparotomy were included. Patients without malignant diagnoses were excluded. Clinical and survival data were analyzed.

**Results:** A total of 30 patients underwent open/closed laparotomy only. Out of these patients, 21 (73.3%) had colorectal adenocarcinoma. Before the laparotomy, 17 (56.7%) patients had undergone diagnostic laparoscopies with a median PCI score of 14 (IQR 10 – 20). However, during the laparotomy procedure, the median PCI score was found to be 30 (IQR 26 – 34). The most common reason for not proceeding with CRS and HIPEC surgery was a high PCI score (63.3%) and small bowel involvement (16.7%). Three patients (9.9%) experienced serious postoperative complications (Clavien Dindo >3a) and one patient died during the first postoperative day. The median length of stay was 7 (IQR 5-13) days. After the explorative laparotomy, sixteen (76.2%) patients received palliative chemotherapy (median survival of 16.6 months), while five received none (median survival of 3.65 months). The median survival for the whole group was 10.4 (95% CI 6.4-17.9) months, and only one patient was alive after three years.

**Conclusion:** Patients diagnosed with PSM who undergo only exploratory laparotomy (open/close) have a poor prognosis. Exploratory laparotomy poses significant risks of serious postoperative complications, as well as a lengthy hospital stay. Improved diagnostic tools are urgently needed to help identify the right patients for CRS and HIPEC treatment.

### PO 80 Factors influencing survival for patients with initially unresectable peritoneal surface malignancies (PSM) treated with pressurized intraperitoneal aerosol chemotherapy (PIPAC): analysis of a large prospective cohort #PO 80

#### Oral communication

R. Orgad^1^, N. Bakrin^1^, B. Isabelle^2^, L. Villeneuve^2^, O. Glehen^1^, V. Kepenekian^1^

^1^Department of General Surgery and Surgical Oncology, Lyon Sud University Hospital, Pierre Benite, France - Lyon (France),

^2^EA 3738 CICLY, Lyon 1 University, Lyon, France - Lyon (France)

**Abstract**

**Background:** PIPAC a novel approach to deliver intraperitoneal chemotherapy, may improve treatment efficacy for PSM by enhancing drug exposure and diffusion to tumor cells. Safety, tolerance and promising survival results were already reported but the influence of the number of PIPAC procedures on prognosis has not been evaluated.

**Methods:** A retrospective analysis from a prospectively maintained database of all patients who underwent PIPAC treatment for unresectable PSM between January 2016 and January 2023

**Results:** A total of 346 patients underwent 1200 PIPAC treatments in the study period. Two-thirds of patients completed 3 or more PIPAC procedures. The overall median survival in our cohort was 12 months from 1st PIPAC procedure with a median follow up of 43 months. Patients that completed less than 3 PIPAC procedures had a significantly shorter median survival 5 months from 1st PIPAC vs 9 months for the patients that completed 3 procedures vs 16 months for patients that completed more than 3 procedures (p<0.001). Patients that converted to resectable disease having a curative CRS + HIPEC had the longest median survival of 37 months from 1st PIPAC and 52 months from diagnosis of PSM. We found that PSM origin and receiving 3 or more PIPAC treatments were independently correlated with better survival in the overall population, in the group of ulitmatley unresectable PSM and after propensity score weighting.

**Conclusions:** When 3 or more PIPAC procedures can be delivered in combination to systemic chemotherapy, survival is significantly improved. Its use should be validated by prospective studies.

Figure 1 - Kaplan Meier survival plots from 1st PI

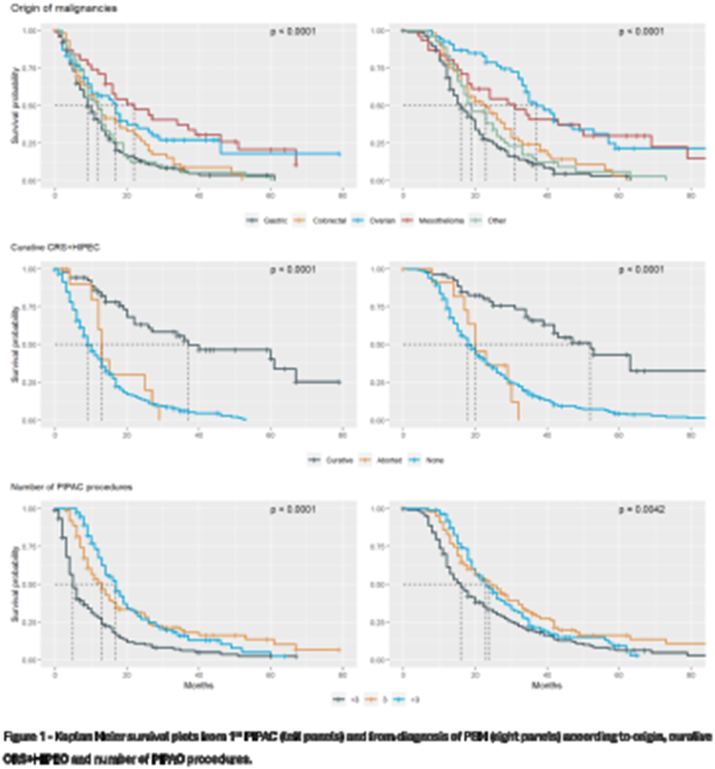



### PO 81 Advantages and Limitations of Minimally Invasive Cytoreductive Surgery and Hyperthermic Intraperitoneal Chemotherapy in Pseudomyxoma Peritonei of Appendiceal Origin #PO 81

#### Oral communication or poster

T. Tawantanakorn^1^, A. Methasate^1^, A. Trakarnsanga^1^, V. Chinswangwatanakul^1^, T. Akaraviputh^1^, T. Parakonthun^1^, J. Swangsri^1^, C. Phalanusitthepha^1^, T. Suwatthanarak^1^, C. Nampoolsuksan^1^

^1^Siriraj hospital, Mahidol university - Bangkok (Thailand)

**Abstract**

**Background:** Minimally invasive surgery provides enhanced recovery, reduced morbidity, and mortality. While the laparoscopic approach for cytoreductive surgery and hyperthermic intraperitoneal chemotherapy (CRS/HIPEC) remains challenging, the advantages are expected to improve postoperative outcomes.

**Methods:** A retrospective cohort study assessed the feasibility and safety of laparoscopic CRS/HIPEC in appendiceal mucinous neoplasm patients with a PCI score of 15 or less who underwent surgery at Siriraj Hospital between January 2011 and December 2022.

**Results:** Eleven patients underwent laparoscopic CRS/HIPEC, with 4 (57.14%) requiring conversion to open surgery. Seven successful laparoscopic CRS/HIPEC cases were compared to 41 cases in open surgery. The median peritoneal cancer index (PCI) score was lower in the laparoscopic group (1, range 0-5) compared to the open group (8, range 0-15, p 0.001). Lower and pelvic peritonectomy were more frequent in open CRS/HIPEC (p = 0.002, p = 0.034 respectively). It was discovered that conversion to open surgery was associated with a PCI of 12 or higher, along with peritoneal nodules located in the lower abdomen, deep pelvic cavity, or right subdiaphragmatic space, and multiple nodules in the small bowel and mesentery. Operative time and length of hospital stay did not significantly differ between groups, but laparoscopic surgery had lower estimated blood loss. The laparoscopic approach resulted in a faster median time to feed (3 days vs. 4 days, p = 0.034). Postoperative complications in the open CRS/HIPEC group were 19.5%, with one case classified as Clavien-Dindo Grade 3a. There were no complications in the laparoscopic group.

**Conclusions:** Laparoscopic CRS/HIPEC is safe and feasible in patients with appendiceal mucinous neoplasms, with benefits for improving postoperative bowel recovery. We advise preparing for potential conversion to open surgery, particularly in cases with a high PCI score and when the peritoneal nodule is located in the pelvic cavity.

### PO 82 Peritoneal Carcinomatosis in the Elderly: Too Old for Cytoreductive Surgery and Hyperthermic Intraperitoneal Chemotherapy (CRS/HIPEC)? #PO 82

#### Oral communication or poster

S. Yadegarynia^1^, A. Hernandez^2^, M. Montealegre^2^, P. Borowsky^2^, C. Cash^2^, K. Gomez^2^, P. Joshi^2^, D. Noe^2^, H. Bahna^1^, M. Moller^3^

^1^University of Miami/ JFK Medical Center - West Palm Beach (United States),

^2^University of Miami Miller School of Medicine - Miami (United States),

^3^University of Chicago Pritzker School of Medicine - Chicago (United States)

**Abstract**

**Introduction:** Cytoreductive surgery with hyperthermic intraperitoneal chemotherapy has been established as an effective treatment modality for patients with peritoneal malignancies with significant improvement in overall survival. There are recommendations against this procedure in elderly patients as its postoperative outcomes are still unclear. Therefore, we examined postoperative outcomes in patients 70 years of age or older.

**Methods:** We retrospectively analyzed a prospective two-institution database of patients with peritoneal malignancies undergoing CRS/HIPEC from 2011-2023. We analyzed outcomes including morbidity, mortality, and overall survival. We performed descriptive statistics to analyze population characteristics and Kaplan-Meier survival analysis for overall survival (OS), recurrence, and recurrence-free survival (RFS).

**Results:** Of the 12 patients identified, median age was 71 years, 67% were female, and 67% had an ECOG performance status of 0. Primary tumors included: appendiceal mucinous (33%), mesothelioma (16%), gastric (16%), and unknown primary (8%). Nine patients received neoadjuvant chemotherapy. Mean length of operation was 8.9 hours. Peritoneal cancer index ranged from 4 to 18 with 82% achieving a completeness of cytoreduction score of 0. Mean length of hospital stay was 10 days with mean SICU admission of 5 days. Seven patients experienced postoperative Clavien-Dindo grade 1 complication, with only one patient experiencing grade three. The median follow up was 25 months, 2-year OS rate of 100%, and total RFS of 50%. There were 2 deaths, 4 recurrences, and 50% of patients were alive with no evidence of disease at the end of the follow up period.

**Conclusion:** CRS/HIPEC seems to be a safe and feasible surgical option in select elderly patients with peritoneal carcinomatosis. Careful patient selection and implementation of proper postoperative care can reduce risk of complications. Given a rapidly aging population requiring advanced oncologic care, further studies are required to elucidate better predictors of outcomes and assess quality of life in this population.

### PO 83 Ex vivo imaging unravels the potential clinical utility of Cyph-11 in the detection of peritoneal metastases #PO 83

#### Oral communication or poster

X.Y.S. Ong^1^, M. Cai^1^, Q.X. Tan^1^, J.W.S. Tan^1^, C.J. Seo^2^, J.S.M. Wong^2^, C.S. Chia^2^, B.D. Gray^3^, K.Y. Pak^3^, C.A.J. Ong^1^

^1^Laboratory of Applied Human Genetics, Division of Medical Sciences, National Cancer Centre Singapore, 30 Hospital Boulevard, Singapore 168583, Singapore - Singapore (Singapore),

^2^Department of Sarcoma, Peritoneal and Rare Tumours (SPRinT), Division of Surgery and Surgical Oncology, National Cancer Centre Singapore, 30 Hospital Boulevard, Singapore 168583, Singapore - Singapore (Singapore),

^3^Molecular Targeting Technologies, Inc., West Chester, Pennsylvania 19380, United States. - Pennsylvania (United States)

**Abstract**

**Introduction:** Cytoreductive surgery (CRS) followed by instillation of hyperthermic intra-peritoneal chemotherapy (HIPEC) remains the only curative treatment for patients with peritoneal metastases (PM), where complete resection is known to be associated with the best overall outcomes. However, detection of residual tumour to facilitate removal of macroscopic disease is largely based on the operating surgeon’s discretion and could potentially be complemented with an objective imaging tool. Here, we performed ex vivo validation to evaluate the clinical utility of CypH-11 in detecting PM via topical administration.

**Methods:** The utility of CypH-11 was evaluated in 15 surgical patients diagnosed with peritoneal metastases. Matched normal and tumour tissues removed during CRS as part of standard therapy were systematically harvested for ex vivo imaging. Tissue subjected to the CypH-11 dye were imaged at intervals across a specified timing using IVIS Lumina III In Vivo Imaging System. Images were analysed using Aura Imaging Software. Formalin-fixed paraffin embedded tissue blocks were generated from the Cyph-11 treated specimen and subsequently sectioned for review by a pathologist.

**Results:** Observations of differential fluorescence signals across or within specimens were reported in 11 cases which then underwent review by a pathologist. Out of these 11 cases, histopathological assessment revealed that microscopic tumours were observed in 6 cases with PM that were derived from multiple histological subtypes, namely 4 colorectal, 1 peritoneal and 1 of endometrial origin. Our preliminary analysis suggests that the best clinical utility of CypH-11 could be in patients with colorectal malignancy, with the possibility of expanding its use to other histological subtypes.

**Conclusions:** Current data from this study serves as a proof of concept trial demonstrating the potential clinical use of CypH-11 in patients with PM and paves the way for its further development as an adjunctive tool to improve outcomes in surgical patients.

### PO 84 Quality of life and cost analysis of patients with unresectable peritoneal metastases after pressurized intra-peritoneal aerosol chemotherapy (PIPAC) #PO 84

#### Oral communication or poster

B. Paik^1^, M. Cai^1^, W.J. Fong^1^, W.S. Ong^1^, C.J. Seo^1^, J. Ong^1^, J. Wong^1^, C. Chia^1^

^1^Department of Sarcoma, Peritoneal and Rare Tumours (SPRinT), Division of Surgery and Surgical Oncology, National Cancer Centre Singapore and Singapore General Hospital, Singapore - Singapore (Singapore)

**Abstract**

**Background:** Unresectable peritoneal metastases (PM) portend dismal prognosis, often leading to distressing symptoms and poor health-related quality of life (HRQoL). Pressurized intra-peritoneal aerosol chemotherapy (PIPAC) has emerged as a promising solution to palliate symptoms, though costs and HRQoL data remains scarce.

**Methods:** We conducted a prospective cohort study including patients with unresectable PM undergoing consecutive PIPACs with systemic treatment between August 2020 and July 2023. Patient, tumor, and PIPAC related characteristics were recorded. HRQoL was measured using the 5-level EuroQol-5L (EQ-5D-5L). Quality adjusted life years (QALYs) gained were derived from EQ-5D-5L scores collected at each assessment point (before and after each PIPAC). Total healthcare costs included inpatient and outpatient costs.

**Results:** A total of 84 PIPAC were performed for 42 patients (mean=2.3,SD=1.4). 12(29%) patients had gastric, 22(52%) appendiceal/colorectal PM and 8(19%) other PM. No major adverse events occurred. Mean peritoneal cancer index (PCI) scores at 1st , 2nd and 3rd PIPACs were 17.8, 17.8 and 15.4 respectively. Mean EQ-5D-5L utility index was 0.69 at baseline and 0.81 after 3 PIPACs (mean change 0.11,p=0.012). Mean total healthcare cost, including PIPAC and systemic chemotherapy or immunotherapy was SGD$253,676 per patient. Mean cost per PIPAC was SGD$15,317 (6% of total costs). Figure 1 depicts costs attributable to surgery vs. others during each PIPAC admission. Overall mean QALY gained was 0.28; cost per QALY gained per patient was SGD$905,989.

**Conclusions:** Consecutive PIPACs in patients with unresectable PM were associated with improvement in HRQoL. PIPAC-related costs were a small proportion of total healthcare costs incurred.

Total PIPAC costs and costs breakdown per patient

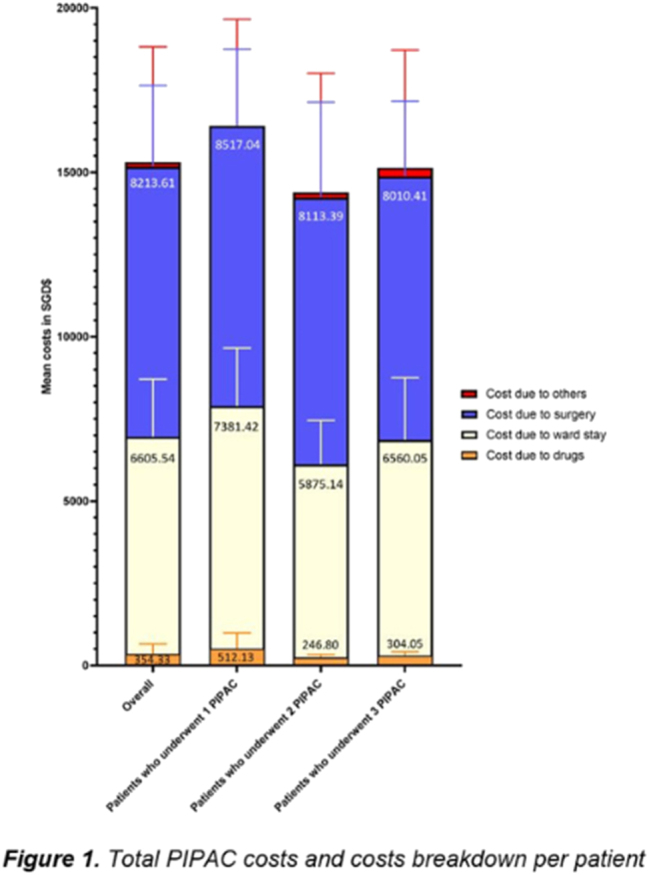



### PO 86 Advancing Intraperitoneal Drug Delivery: NIR-Mediated Specificity Enhancement for Studying Electric Field-Driven Nanoparticle Penetration #PO 86

#### Oral communication or poster

N. Saeed^1^, I.A. Okkelman^2^, R.I. Dmitriev^2^, S.M. Borisov^3^

^1^Lab of Experimental surgery, Ghent University, Belgium - GHENT, Belgium (Belgium),

^2^Tissue Engineering and Biomaterials Group, Ghent University, Belgium - GHENT, Belgium (Belgium),

^3^Institute of Analytical Chemistry and Food Chemistry, University of Technology, 8010 Graz, Austria - Graz, Austria (Austria)

**Abstract**

**Background:** Intraperitoneal drug delivery holds considerable therapeutic potential for combatting peritoneal metastases, yet its efficacy is often hindered by limited tissue permeability, impeding optimal drug penetration. To address this challenge, this study explores the application of electromotive drug administration (EMDA) as a means to enhance nanoparticle (NP) penetration across various peritoneal tissues.

**Methods:** Freshly excised peritoneal tissue fragments sourced from porcine, rat, and human donors were subjected to exposure with cationic amine-modified polymeric beads under conditions of EMDA (employing pulsed Direct current electrical stimulation) vs Passive Diffusion. After exposure, tissues were sectioned and evaluated under microscopy. Strong autofluorescence of peritoneal models limits the precise quantification and discrimination of NPs from autofluorescence (AF) inherent in the tissue microenvironment. To overcome this limitation, subsequent samples were treated with far-red emitting RL-100 nanoparticles doped with aza-BODIPY dye, facilitating analysis under FLIM microscopy. The nanoparticles were then optimized and characterized for use in peritoneal tissues.

**Results:** Initially, confocal microscopy faced challenges arising from the spectral overlap between conventional fluorescent beads and tissue autofluorescence. Notably, distinct differences in NP lifetime were observed between samples subjected to passive diffusion and those treated with EMDA. This disparity suggests the influence of the tissue milieu on NP fluorescence dynamics. This investigation underscores the utility of near-infrared NPs in conjunction with FLIM for accurate NP detection and differentiation from autofluorescence within peritoneal tissue models.

**Conclusion:** Differences in NP lifetime suggest potential avenues for further investigation into EMDA’s interaction dynamics with peritoneal tissues, offering insights into enhancing drug delivery efficiency.

Fig: FLIM Microscopy images of a. RL-100 NP s

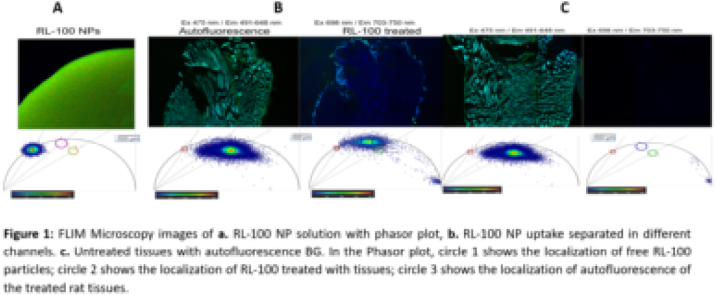



### PO 87 Preliminary data from randomized multicenter phase III trial PIPAC VEROne #PO 87

#### Oral communication or poster

F. Casella^1^, M. Bencivenga^1^, F. Meloni^1^, C. Puccio^1^, R. Rigoli^1^, I. Zacchi^1^, F. Filippini^1^, F. Tedone^1^, G. De Manzoni^1^

^1^General and Upper GI Surgery, University of Verona - Verona (Italy)

**Abstract**

**Background:** PIPAC VER-One is a prospective, randomized, multicenter phase III clinical trial that aims to evaluate the effectiveness of the use of PIPAC in combination with systemic chemotherapy in patients with gastric cancer and synchronous positive peritoneal cytology and/or limited peritoneal metastases (peritoneal cancer index [PCI] ≤6).

**Methods:** Six italian centers are included in the study. Currently have been randomized 12 patients: 6 in the arm A (control) treated with standard systemic chemotherapy and 6 in the arm B (experimental) treated with a bidirectional scheme including PIPAC and systemic chemotherapy. In this moment is being approved an amendment designed to put Progression Free Survival as primary endpoint (with the Secondary Resectability Rate) and the possibility of doing FLOT chemotherapy or associate immunotherapy (Nivolumab) to systemic chemotherapy in patients with PDL1 CPS≥5. With this amendment we have also specified the need to perform an exploratory laparoscopy in case of radiological suspicion of recurrence.

**Results:** 7 patients underwent Cytoreductive surgery (CRS) combined with hyperthermic intraperitoneal chemotherapy (HIPEC), 3 patients are still doing systemic therapy. No patient had disease progression but we had 2 drop-out, one due to logistical problems of the patient and another one due to worsening of patient’s general condition. All interventions achieved a R0 resection and only one patient had a severe complication (Clavien Dindo ≥ 3a).

**Conclusions:** From the first results, the study seems to be feasible and safe for patients. The inclusion criteria are very restrictive and we need to recruit 98 patients (including 10% of droup-out), so we think the clinical trial will last a total of 6 and a half years, considering a follow up of 3 years for each patient.

### PO 88 A single-center retrospective study of cytoreductive surgery and hyperthermic intraperitoneal chemotherapy for the treatment of peritoneal surface malignancies #PO 88

#### Oral communication or poster

K. Iliakopoulos^1^, L. Chardalias^1^, D. Politis^1^, K. Kordeni^1^, P. Metheniti^1^, D. Massaras^1^, G. Fragulidis^1^, J. Contis^1^

^1^2nd Department of Surgery, Aretaieion University Hospital, School of Medicine, National & Kapodistrian University of Athens - Athens (Greece)

**Abstract**

**Background:** Peritoneal surface malignancies (PSMs) are an heterogenous group of primary and metastatic cancers affecting the peritoneum, associated with poor prognosis. Cytoreductive surgery (CRS) combined with hyperthermic intraperitoneal chemotherapy (HIPEC) has radically changed the approach of patients with malignant tumors of the peritoneum. This study aims to present our recent experience with CRS and HIPEC, for PSMs in a single tertiary center.

**Methods:** Data were retrospectively collected from all patients with PSMs who underwent CRS and HIPEC in our department between 01/01/2020 and 31/12/2023. The standard HIPEC protocols were used according to type of PMS based on mitomycin, cisplatin and oxaliplatin agents through an open abdomen technique.

**Results:** During this period 53 patients (43 women and 10 men) underwent CRS and HIPEC. The median age of patients was 58 y.o. (range: 41-81). The histological type of the primary tumors was ovarian cancer (45%, N=24), colorectal cancer (19%, N=10), pseudomyxoma peritonei (15%, N=8), appendiceal mucinous neoplasm (13%, N=7), mesothelioma (4%, N=2), and 2 patients with uterine leiomyosarcoma. Completeness of cytoreduction (CC) score 0-1 (CC-0/1) was achieved in all patients prior to HIPEC. The median length of stay in the hospital was 10 days, while including 1 day of minimum stay in the intensive care unit (ICU). There was no 30-day mortality. Morbidity grade III-IV according to Clavien-Dindo Classification complication rate was present in 20% of patients. One out of 53 patients died due to the disease progression 18 months after the operation (brain metastases).

**Conclusions:** The current study demonstrated the results of treatment with CRS and HIPEC in a single tertiary educational center. Patients who underwent CRS and HIPEC for PSMs achieved moderate survival rates with acceptable postoperative morbidity and mortality risk while the method has radically changed the approach of patients with primary and metastatic malignant tumors of the peritoneum.

### PO 89 HSF-1/miR-145-5p transcriptional axis enhances hyperthermia intraperitoneal chemotherapy efficacy on peritoneal ovarian carcinosis #PO 89

#### Oral communication

R. Lo Dico^1^, M. Valle^1^

^1^IFO National Cancer Institut Regina Elena - Rome (Italy)

**Abstract**

Hyperthermic intraperitoneal administration of chemotherapy (HIPEC) increases local drug concentration and reduces systemic side effects associated with prolonged adjuvant intraperitoneal exposure in patients affected by either peritoneal malignancies or metastatic diseases originating from gastric, colon, kidney and ovarian primary tumors. Mechanistically, the anticancer effects of HIPEC have been poorly explored. Herein we documented that HIPEC treatment promoted miR-145-5p expression paired with a significant downregulation of its oncogenic target genes c-MYC, EGFR, OCT4 and MUC1 in a pilot cohort of patients with ovarian peritoneal metastatic lesions. RNA sequencing analyses of ovarian peritoneal metastatic nodules from HIPEC treated patients unveils HSF-1 as a transcriptional regulator factor of miR-145-5p expression. Notably, either depletion of HSF-1 expression or chemical inhibition of its transcriptional activity impaired miR-145-5p tumor suppressor activity and the response to cisplatin in ovarian cancer cell lines incubated at 42^o^ C. In aggregate, our finding highlight a novel transcriptional network involving HSF-1, miR145-5p, MYC, EGFR, OCT4 and MUC1 whose proper activity contributes to HIPEC anticancer efficacy in the treatment of ovarian metastatic peritoneal lesions.

### PO 155 Reviving HIPEC: enhancing survival rates with curative intent hyperthermic intraperitoneal chemotherapy for gastric cancer with peritoneal metastasis. #PO 155

#### Oral communication or poster

B. Joung^1^, W.J. Jeon^1^, D. Daniel^2^, D. Dani^3^, Y. Yanghee^3^

^1^Loma Linda University - Loma Linda (United States),

^2^UCSF Fresno - Fresno (United States),

^3^City of hope national cancer center - Duarte (United States)

**Abstract**

**Background:** The prognosis of gastric cancer with peritoneal metastasis (GC-PM) remains dismal. Despite the many studies evaluating hyperthermic intraperitoneal chemotherapy and cytoreductive surgery (HIPEC-CRS) for GC-PM in various treatment settings, its curative role in combination with systemic chemotherapy (SC) remains unclear.

**Method:** To determine the survival benefit of CRS-HIPEC in GC-OM, we conducted a systematic review and meta-analysis in accordance with the PRISMA guidelines on primary studies from January 1, 2010, to December 31, 2023. Inclusion criteria encompassed studies involving adults with GC and histologically confirmed PM who underwent curative- intent gastrectomy, D2 lymphadenectomy, and SC. Exclusion criteria comprised studies involving GC patients lacking PM involvement and HIPEC utilized solely for prophylactic purposes. The primary endpoint was overall survival (OS), with a focus on survival outcomes 1-year, 2-year, and 3-year intervals. A random effects meta-analysis of risk ratios was conducted using RevMan, with heterogeneity assessed utilizing I2.

**Result:** Out of 452 identified studies, we incorporated 5 studies involving a total of 524 patients for comparative analysis of survival outcomes between CRS-HIPEC and SC. Significantly superior OS was observed in patients undergoing CRS+HIPEC compared to those received SC alone (odds ratio (OR) 0.47, 95% CI [0.33-0.66], I2=25%, P<0.0001). Subsequent analysis revealed enhanced1-year OS (OR 0.56, 95% CI [0.32-0.97], I2=43%, p=0.04), 2-year OS (OR 0.41, 95% CI [0.22-0.75], I2=23%, p=0.004), and 3-year OS (0.35, 95% CI [0.14-0.84], I2=21%, p=0.02). The low heterogeneity (3%) observed amongst all the groups indicated that variations within the subgroups did not significantly impact the homogeneity of the dataset.

**Conclusion:** The incorporation of CRS and HIPEC alongside SC led to statistically significant enhancement in OS up to year 3 compared to SC alone in GC-PM. Additional investigations are warranted to assess the role of HIPEC in the curative setting, particularly in conjunction with emerging targeted therapeutic modalities.

### PO 156 Engineering and preclinical validation of an impaction-based PIPAC nebuliser (Capnotip®) with a spraying angle over 150° and improved aerosol homogeneity #PO 156

#### Oral communication

I. Sautkin^1^, H. Schoenfelder^1^, M. Reymond^1^

^1^R&D laboratory, Capnopharm GmbH, Tübingen, Germany - Tübingen (Germany)

**Abstract**

**Background:** Heterogeneous tissue drug distribution is reported after PIPAC. We engineered a 3rd-generation PIPAC nebuliser, based on impaction single-nozzle technology, to improve the homogeneity of tissue drug distribution. The performance of the new nebuliser (Capnotip®) was compared in vitro with that of the reference device (Capnopen®), (Capnopharm GmbH, Tübingen, Germany).

**Methods:** During aerosolisation, the flow is directed onto an impaction plate with optimised geometry, from which the aerosol is reflected. The Median Aerodynamic Diameter (MAD) of the aerosol droplets generated from distilled water, glucose 5 %, and silicon oil (5 cSt) was measured by laser diffraction spectroscopy (SprayTec™, Malvern, Kassel, Germany). The spraying angle was measured at the tip of the nebuliser and photodocumented. Aerosolisation patterns were depicted on 2D and 3D targets and compared between devices.

**Results:** The MAD was for distilled water, glucose 5 % and silicon oil 5-cSt with Capnotip® vs Capnopen®: 26.1 (CI-95% 24.4-27.9) vs 29.28 (26.6-32.4), 26.6 (25.5-27.7) vs 31.74 (31.5-32.0) and 35.5 (30.5-40.6) vs 26.74 (24.4-29.1) µm. The mean angle of aerosolisation with Capnotip® vs Capnopen® was 155° vs 57°. The aerosolisation patterns on the 2D and 3D targets were qualitatively superior with Capnotip® vs Capnopen®, with some residual inhomogeneity.

**Conclusion:** The Capnotip® is a 3rd-generation, single-nozzle PIPAC nebuliser with an angle of aerosolisation over 150°, allowing a superior surface spray coverage vs the Capnopen®. The MAD was comparable between devices (under 36 µm) with a narrow distribution range. Pharmacological measurements are ongoing on the ex vivo enhanced inverted urinary bladder model (eIBUB).

### PO 157 Intraperitoneal paclitaxel treatment combined with S-1+ Oxaliplatin for gastric cancer with peritoneal metastasis followed by conversion surgery #PO 157

#### Poster

S. Saito^1^, Y. Hironori^1^, H. Ohzawa^1^, K. Takahashi^1^, R. Kanamaru^1^, Y. Futo^1^, Y. Kaneko^1^, K. Kurashina^1^, Y. Hosoya^1^, J. Kitayama^1^

^1^department of surgery, Jichi Medical Uniersity - Shimotsuke (Japan)

**Abstract**

**Introduction:** Peritoneal metastases (PM) are the most frequent type of metastases and site of recurrence in patients with advanced gastric cancer (GC). Intraperitoneal (IP) administration of paclitaxel (PTX) has a great pharmacokinetic advantage to control peritoneal lesions.

**Objectives:** To determine the efficacy of and tolerance to combination chemotherapy using IP-PTX and systemic S-1/oxaliplatin (SOX) in patients with PM from GC.

**Methods:** Patients with GC laparoscopically diagnosed with macroscopic or microscopic PM were enrolled. PTX was administered IP at 40 mg/m2 on days 1 and 8. Oxaliplatin was administered IV at 100 mg/m2 on day 1, and S-1 was administered at 80 mg/m2/day for 14 consecutive days, repeated every 21 days. When the macroscopic shrinkage of peritoneal lesions was confirmed, gastrectomy with lymph node dissection was performed as conversion surgery.

When staging laparoscopy, exosomes were isolated from peritoneal fluid and expression levels of miR-21-5p, miR-223-3p, and miR-29b-3p determined.

**Results:** Ninety-six patients received SOX+ IP-PTX from 2016 to March 2024. Disappearance of malignant ascites rate was 73% and cytology turned negative rate was 70%. The 1-year overall survival (OS) rate was 70% with a median survival (MS) of 20 months. Gastrectomy was performed in 44 patients who had an excellent response to the therapy. Their 1- , 2-year and 5-year OS rates were 100%,71% and 40%, respectively with MS of 39 months.

The 1-year and 2-year OS rate of the patients who did not undergo gastrectomy was 43% and 18% with MS of 12months, which was significantly lower than in patients with gastrectomy.

Grade 3/4 toxicities included neutropenia, leukopenia, and anemia. There were no treatment-related deaths.

Overall survival of patients with high miR-21/miR-29b or miR-223/miR-29b ratios was significantly worse than in patients with low ratios.

**Conclusions:** IP-PTX and SOX combined with gastrectomy is a promising treatment strategy for GM with PM.

### PO 158 Clinical pathway and technique of Pressurized IntraThoracic Aerosol Chemotherapy (PITAC) directed therapy #PO 158

#### Oral communication or poster

P.S.H. Hansen^1^, C.A.L. Lindegaard^1^, S. Detlefsen^2^, L. Eckhoff^3^, M. Graversen^1^, M. Jelin-Klaric^3^, K.M. Jensen^4^, M.B. Mortensen^1^

^1^Odense PIPAC Center, Odense University Hospital, Denmark. Department of Surgery, Odense University Hospital - Odense (Denmark),

^2^Odense PIPAC Center, Odense University Hospital, Denmark. Department of Pathology, Odense University Hospital - Odense (Denmark),

^3^Department of Oncology, Odense University Hospital - Odense (Denmark),

^4^Department of Anesthesiology, Odense University Hospital - Odense (Denmark)

**Abstract**

**Background:** Pressurized IntraThoracic Aerosol Chemotherapy (PITAC) is a minimally invasive treatment platform for patients with malignant pleural effusion (MPE) and/or pleural metastasis (PLM). The rationale and technical details of PITAC are based on Pressurized IntraPeritoneal Aerosol Chemotherapy (PIPAC). However, there is no currently consensus on PITAC indications, techniques, methods for evaluating response, or follow-up. To advance PITAC to stage 2a of the IDEAL framework, we have defined a clinical pathway and performed a detailed description of the procedure to be used in the first PITAC phase-I study (PITAC-OPC5).

**Methods:** New PITAC clinical pathway and technique based on the available literature and our previous PITAC experience.

**Results:** Candidates for PITAC are identified at the PIPAC/PITAC MDT-conference and their clinical pathway include CT, blood tests, spirometry, chest ultrasound, disease mapping, and close follow-up. Patients are anesthetized, intubated in bed using video-assisted double-lumen endotracheal tube, and then positioned in prone position. After infiltrating the port site with local anesthesia, a 12-mm trocar is placed beneath the scapula under ultrasound guidance. A 5-mm trocar is positioned close to the spine. MPE is evacuated or pleural fluid lavage performed and send for evaluation. PLM is recorded according to a new PLM scoring system, and biopsies are taken. After careful review of the dedicated PITAC checklist, PITAC is performed using a certified nebulizer and standard PIPAC doses of chemotherapy. Chemotherapy-saturated air and fluid are evacuated after 30 minutes of passive diffusion, and the lung is expanded by manual ventilation. Port sites are inspected, closed in two layers, and infiltrated with local anesthesia. Patients are monitored in hospital for 24 hours. Biopsies are evaluated according to the proposed Thoracic Regression Grading Score (TRGS).

**Conclusion:** A specified clinical pathway and dedicated PITAC checklist were developed to implement PITAC, and is now used during the first phase-I study.

### PO 159 Safety and effect of Pressurized IntraThoracic Aerosol Chemotherapy (PITAC) directed therapy against malignant pleural effusion and pleural metastasis #PO 159

#### Oral communication or poster

P.S. Hansen^1^, S. Detlefsen^2^, L. Eckhoff^3^, M. Graversen^1^, M. Jelin-Klaric^3^, K.M. Jensen^4^, M.B. Mortensen^1^

^1^Odense PIPAC Center, Odense University Hospital, Denmark. Department of Surgery, Odense University Hospital, Denmark - Odense (Denmark) - Odense (Denmark),

^2^Odense PIPAC Center, Odense University Hospital, Denmark. Department of Pathology, Odense University Hospital, Denmark - Odense (Denmark),

^3^Department of Oncology, Odense University Hospital, Denmark - Odense (Denmark),

^4^Department of Anesthesiology, Odense University Hospital, Denmark - Odense (Denmark)

**Abstract**

**Background:** Malignant pleural effusion (MPE), with or without solid pleural metastasis (PLM), is a common and debilitating condition in several advanced cancer diseases. Pressurized IntraThoracic Aerosol Chemotherapy (PITAC) is a platform for repetitive nebulization of antineoplastic agents to the pleural cavity in these patients. PITAC was performed in humans for the first time in 2012 based on the technology and experience gained from Pressurized IntraPeritoneal Aerosol Chemotherapy (PIPAC). However, data on the safety and potential clinical effects of PITAC remain limited.

**Method:** Safety and potential effects of PITAC were evaluated using data from Odense PIPAC Center (OPC) collected between 2018-2021 alongside a review of the available literature.

**Results:** A total of 47 PITACs in 25 patients were available for evaluation. Data were heterogeneous and limited. Two major surgical complications were recorded: a lung lesion and bleeding after biopsy with PITAC completed in the latter. No other major surgical complications were registered, and the most frequent postoperative events were chest pain and urinary retention. Three patients were evaluated using the proposed histological Thoracic Regression Grade Score (TRGS), and complete response was seen in one patient. Other response evaluation was of limited value (e.g. CT-scans, cytology of MPE or pleural lavage fluid and lung ultrasound). Long-term follow-up did not reveal procedure related problems or complications, but again data were limited.

**Conclusion:** Due to significant variations in patient selection, the PITAC technique, complications during monitoring, and long-term follow-up, it is presently difficult to draw firm conclusions regarding safety and potential clinical outcome. PITAC remains in phase 1 of the IDEAL framework.

### PO 160 Implementation and evaluation of Pressurized IntraThoracic Aerosol Chemotherapy (PITAC) for patients with malignant pleural effusion - Protocol for the Danish phase I PITAC-OPC5 trial #PO 160

#### Oral communication or poster

P.S. Hansen^1^, S. Detlefsen^2^, L. Eckhoff^3^, C.W. Fristrup^1^, M. Graversen^1^, M. Jelin-Klaric^3^, K.M. Jensen^4^, M.B. Mortensen^1^

^1^Odense PIPAC Center, Odense University Hospital, Denmark. Department of Surgery, Odense University Hospital, Denmark - Odense (Denmark) - Odense (Denmark),

^2^Odense PIPAC Center, Odense University Hospital, Denmark. Department of Pathology, Odense University Hospital, Odense, Denmark. - Odense (Denmark),

^3^Department of Oncology, Odense University Hospital, Odense, Denmark - Odense (Denmark),

^4^Department of Anesthesiology, Odense University Hospital, Odense, Denmark - Odense (Denmark)

**Abstract**

**Background:** Pressurized IntraThoracic Aerosol Chemotherapy (PITAC) is a minimally invasive treatment platform for patients with malignant pleural effusion (MPE) and/or pleural metastasis (PLM). PITAC is based on Pressurized IntraPeritoneal Aerosol Chemotherapy (PIPAC), which has proven to be safe and feasible. Since 2012, 47 PITACs have been performed, but prospective data on feasibility, safety and potential local response are lacking. We present the first PITAC phase-I study protocol, which aims to monitor and evaluate patient and personnel safety and toxicity.

**Methods:** The prospective, controlled, phase-I study (PITAC-OPC5) aims to treat patients with MPE and/or PLM with PITAC. Although there are no data to support the estimated number of patients needed, previous experience suggests a non-access rate of 20%. Twenty patients with MPE will be included, each receiving two or more PITACs at four-week intervals. During video-assisted thoracoscopy, MPE is evacuated or pleural lavage performed, and the extent of visible PLM assessed. Pleural biopsies are collected, if possible, for histological response as per Thoracic Regression Grading Score (TRGS). Patients will be screened for intra- and postoperative complications related to PITAC. The primary outcome is the number of patients experiencing Clavien-Dindo≥3b or Common Terminology Criteria for Adverse Events (CTCAE)≥4 within 30 days. Secondary objectives include evaluating the extent of visible PLM, TRGS, cytological response, personnel safety, quality of life, and change in MPE volume.

**Results:** It is expected that PITAC is safe and feasible for patients and personnel. Positive results on the reduction of MPE have been published, why we expect this study to reduce MPE.

**Conclusion:** The results may have significant impact on the next clinical, technical, and scientific steps in the implementation of PITAC. Given the suboptimal treatment options for patients with MPE and/or PLM and the promising results so far, we find the implementation of PITAC ethically reasonable and sound.

### PO 161 First report of success for delivering Mitomycin C with a thermogel in a big animal model with positive pharmacology and surgical security #PO 161

#### Oral communication or poster

C. Mouawad^1^, A. Thouvenin^2^, P. Rozenbaum^1^, C. Pimpie^1^, V. Kemmel^3^, C. Eveno^4^, V. Boudy^2^, N. Mignet^2^, M. Pocard^1^

^1^Université de Paris, INSERM UMR 1275: CAP Paris Tech, Carcinomatosis Peritoneum Paris Technology; Hôpital Lariboisière, 2 rue Ambroise Paré, 75010 Paris, France - Paris (France),

^2^Université Paris Cité, CNRS, INSERM, UTCBS, Unité de Technologies Chimiques et Biologiques pour la Santé, F-75006 Paris, France - Paris (France),

^3^Laboratoire de Pharmacologie et Toxicologie Neurocardiovasculaire, UR 7296, Faculté de Médecine de Maïeutique et des Métiers de la Santé, Centre de Recherche en Biomédecine de Strasbourg (CRBS), 67085, Strasbourg, France - Strasbourg (France),

^4^CNRS, INSERM, UMR9020-U1277-CANTHER-Cancer, Université de Lille, CHU Lille, Lille, 59000, France - Lille (France)

**Abstract**

**Background:** We developed an alternative solution to deliver intra peritoneal chemotherapy using a poloxamer-based thermogel (TG) (P407, P188 and Alginate) with chemotherapy-carrying capacity and controlled drug release. Our goal is to offer a rescue TG Kit in case of high carcinomatosis risk, as an alternative to HIPEC procedure. Our previous study on a porcine model in 2022 validated the TG’s spatial distribution and drug-carrier characteristics, but showed a high risk of anastomotic fistula when associated with Oxaliplatin. After the HIPEC-T4 positive results with MMC, we tested the safety of TG associated with MMC on an animal model.

**Methods:** An in-vitro study was done to find the minimal MMC dose required for effective cytolytic effect on CT-26 cells. A murine model study (40 mice) evaluated the TG long-term histological effect and the TG efficacy on PCI decrease. A porcine model study evaluated the TG-MMC’s safety and pharmacodynamics after administering 50 mL of TG associated with 20 mg of MMC into 4 pigs of 30 kg each.

**Results:** Our in-vitro study showed the potent TG cytotoxicity of MMC on CT-26 cells, even at a low dose of 0.47 µmol. At 2 months, murine model results showed no negative effect on animal wellbeing, and a limited residual intra-peritoneal tissular inflammation after TG administration. Our porcine study’s early results showed the absence of anastomotic fistula at the 9th and 13th postoperative day after TG-MMC administration. None of the 10 bowel sutures showed dehiscence and the animal wellbeing was confirmed until autopsy.

**Conclusion:** Our early results validated the safety of administering TG-MMC concomitantly with the performance of digestive anastomoses. After the upcoming evaluation of its pharmacodynamics and PCI reduction characteristic, the association of TG and MMC could eventually offer a valid tool in the hands of surgeons confronted with locally advanced colorectal tumors.

### PO 162 Real-World Application of Cytalux to Illuminate Hidden Peritoneal Disease in Ovarian Cancer Surgery #PO 162

#### Poster

E. Baron^1^, R. Patterson^1^, J.A. Wernberg^1^, R. Sharma^1^

^1^Marshfield Medical Center - Marshfield, WI (United States)

**Abstract**

**BACKGROUND.** Cytalux is the first FDA-approved targeted imaging for highlighting occult peritoneal disease in ovarian cancer (OC). We present the first out-of-trial experience using Cytalux during cytoreductive surgery (CRS) for OC.

**METHODS.** We analyzed two OC cases with peritoneal dissemination. Cytalux was utilized before CRS to confirm tumor uptake and after to identify occult disease. The concordance between Cytalux-positive disease and final pathology was evaluated.

**RESULTS.** Case #1 had primary OC (PCI=21) treated with neoadjuvant chemotherapy (NACT). Case #2 had recurrent disease (PCI=9) after limited debulking + 4 years of Olaparib. In case #1, Cytalux highlighted 17 occult areas after CRS (16 peritoneal + 1 lymph node) with 14 pathologically confirmed (true positive rate – 82%). Among 16 Cytalux-positive peritoneal areas, 3 had no visible disease, and 13 had diminutive lesions upon close inspection. One area of post-NACT fibrosis was Cytalux-positive and later confirmed malignant. Two Cytalux-positive peritoneal areas without visible disease were fulgurated until signal loss but remained positive on final pathology. In case #2, Cytalux detected 2 additional areas: the appendix (malignant) and a small bowel segment (benign). The false-positive rate was 18% and 50% for cases #1 and #2 respectively.

**CONCLUSIONS.** Cytalux can identify occult disease and clarify questionable post-NACT fibrotic lesions in OC, but the false-positive rate is considerable. Loss of Cytalux signal after energy destruction of peritoneal lesions does not reliably confirm successful tumor elimination. While more real-world data are needed to define its optimal application, Cytalux may be an important CRS adjunct in OC.

Cytalux-illuminated peritoneal lesions

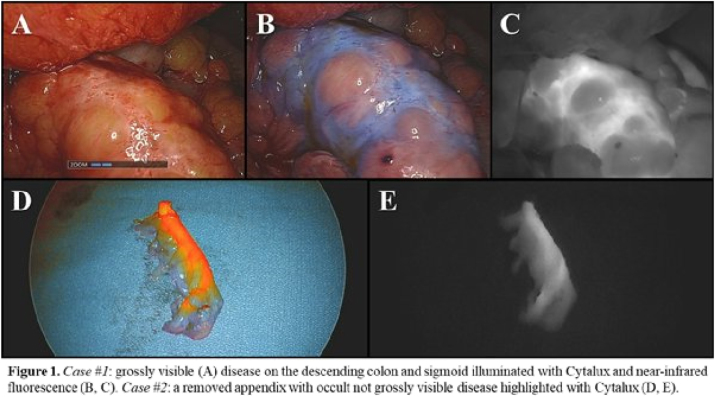



### PO 163 Experience of Cytoreductive surgery and HIPEC - A Multicentre retrospective study #PO 163

#### Oral communication

O. Al Hamdani^1^, M. Al Hosni^2^

^1^Oman Medical Speciality Board - Muscat (Oman),

^2^Sultan Qaboos Comprehensive Cancer Care and Research Centre - Muscat (Oman)

**Abstract**

**Background:** Cytoreductive surgery combined with Hyperthermic Intraperitoneal Chemotherapy (HIPEC) has gained international preference as the optimal surgical intervention for peritoneal carcinomatosis. This study delves into the outcomes of patients who underwent this procedure at three specialized centers in Oman, with a focus on postoperative median survival rates. The aim is to investigate the incidence and common causes of peritoneal metastasis in the Omani population while sharing insights and results derived from our experiences.

**Methodology:** Conducted retrospectively, the study spans from November 2012 to November 2023, utilizing electronic medical records of patients diagnosed with peritoneal metastasis who underwent CRS/HIPEC. Data collection occurred across three specialized centers in Oman, with plans to extend the study further for comprehensive analysis.

**Results and conclusion:** Over an 11-year period, our cohort comprised 130 patients, excluding 30 from the study. Intra-peritoneal therapy varied between centers, with one using Mitomycin C and two opting for oxaliplatin. No clear superiority was observed between the chemotherapy types, likely due to varied patient variables within the study. Our study also focused on peritoneal mets from colorectal cancers with a minority being from other origins.

The primary objective of our study was to present our experiences with cytoreductive surgery and Hyperthermic Intraperitoneal Chemotherapy (HIPEC) while also comparing our outcomes with those of international studies. By doing so, we aimed to contribute to the ongoing discussion regarding the standardization of approaches to this treatment method in our local practice.

### PO 164 Combined Nabpaclitaxel Pressurized IntraPeritoneal Aerosol Chemotherapy with systemic Nabpaclitaxel-Gemcitabine chemotherapy for pancreatic cancer peritoneal metastases: the interim analisys of Nab-PIPAC trial #PO 164

#### Oral communication

A. Di Giorgio^1^, F. Ferracci^1^, C. Lodoli^1^, F. Santullo^1^, L. D’agostino^2^, C. Bagalà^3^, E. Rodolfino^4^, D. Arciuolo^5^, G. Tortora^3^, F. Pacelli^6^

^1^Surgical Unit of Peritoneum and Retroperitoneum, Fondazione Policlinico Universitario A. Gemelli IRCCS - Rome (Italy),

^2^Catholic University of the Sacred Heart - Rome (Italy),

^3^Comprehensive Cancer Center, Fondazione Policlinico Universitario A. Gemelli IRCCS, Catholic University of the Sacred Heart - Rome (Italy),

^4^Dipartimento di Diagnostica per Immagini, Radioterapia Oncologica ed Ematologia, UOC RadiologiaAddomino-Pelvica, Fondazione Policlinico Universitario “A. Gemelli” IRCCS - Rome (Italy),

^5^Unit of Gynecopathology, Department of Woman and Child Health and Public Health, Fondazione Policlinico Universitario A. Gemelli IRCCS - Rome (Italy),

^6^Surgical Unit of Peritoneum and Retroperitoneum, Fondazione Policlinico Universitario A. Gemelli IRCCS, Catholic University of the Sacred Heart - Rome (Italy)

**Abstract**

**Background:** Current treatments for peritoneal metastases (PM) of pancreatic origin are largely ineffective. Pressurized Intraperitoneal Aerosol Chemotherapy (PIPAC) has emerged as a novel method for delivering anticancer drugs with promising efficacy against PM.A recent dose-escalation study identified the safe dose of Nabpaclitaxel.We present midterm results from Nab-PIPAC Trial, a phase-II study combining Nabpaclitaxel-PIPAC (Nab-PIPAC) and systemic Nabpaclitaxel-Gemcitabine chemotherapy for treating pancreatic PM (ClinicalTrials.govNCT05371223).

**Methods:** Patients with pancreatic PM underwent a treatment protocol comprising three combined courses. Each course included two cycles of intravenous Nabpaclitaxel-Gemcitabine and one cycle of Nabpaclitaxel-PIPAC at 112.5mg/m^2^. The study’s primary objective was to assess the Disease Control Rate(DCR) per RECISTv1.1 criteria, while secondary objectives included feasibility, safety, and pathological tumor response. Using Simon’s two-stage design in the first stage 12 patients were enrolled. If 6 or more showed DCR for ≥16 weeks, 26 patients will be enrolled in the second stage (power 80%, alpha0.1).

**Results:** From March 2022 to September 2023, 12 patients were enrolled, and 32 PIPAC were performed. Three patients dropped out (one due to infection, two due to disease progression).Nine patients (75%) completed the study, with DCR for ≥16 weeks (6 stable disease, 3 partial response). Histological tumor regression and PCI improvement were observed in 6 and 3 patients, respectively.There were no treatment-related deaths. The grade 3–4 post-operative complication rate was 3.1% (one patient required laparoscopy for suspected bowel perforation). One grade 4 toxicity was observed (neutropenia). Grade 3 hematological toxicities rate was 25% (1 platelet count decreased, 1 WBC count decrease, 1 neutropenia), Grade 3 non-hematological toxicities rate was 33,3% (3 wound dehiscence, 1 alopecia)

**Conclusion:** The study met its primary endpoint in 9 of 12 patients.Enrollment will proceed to the second phase. While preliminary results are promising for efficacy and safety, definitive conclusions will be drawn upon study completion.

### PO 165 The evolution of treatment in Peritoneal Surface Malignances: 700 patients treated in a single high-volume center. #PO 165

#### Oral communication or poster

M. Aulicino^1^, A. Di Giorgio^1^, F. Santullo^1^, F. Ferracci^1^, C. Abatini^1^, M. Attalla El Halabieh^1^, C. Lodoli^1^, C. Orsini^1^, G. D’annibale^1^, F. Pacelli^1^

^1^Chirurgia del peritoneo e retroperitoneo - roma (Italy)

**Abstract**

**Background:** The implementation of cytoreductive surgery(CRS), the development of techniques to deliver chemotherapy into the abdominal cavity(Hyperthermic Intraperitoneal Chemotherapy-HIPEC and Pressurized Intraperitoneal Aerosol Chemotherapy-PIPAC) and improved staging through pre-operative laparoscopy have allowed a significant prognostic improvement in patients with peritoneal surface malignancies(PSM). Therefore, we evaluated the outcomes of these surgical procedures and their evolution over the years with the increase of surgical expertise. **Method:** This retrospective study included 701 patients with PSM evaluated at the Multidisciplinary Tumor Board of the Fondazione Policlinico Agostino Gemelli and selected for diagnostic laparoscopy, PIPAC or CRS and HIPEC for a total of 887 procedures. **Results:** A total of 356 CRS were performed. The mOS/mDFS in months was: 52/22 in colo-rectal cancers, 18/13 in gastric cancers, 43/26 in mesotheliomas, 54/30 in ovarian cancers. The 5-year OS and DSF in Pseudomyxoma was 89% and 70%. Were performed 370 PIPAC on 184 patients. The mOS in months was: 16 in colo-rectal cancers, 9 in gastric cancers, 14 in pancreatic cancers and 6 in hepatobiliary tumors. An improvement in survival was found with increasing surgical expertise, particularly significant for PIPAC (mOS before-after 2021:6vs14; P:0.001). A total of 310 diagnostic laparoscopies were performed, of which 149 preliminary to CRS. Over the years, an increase in number of diagnostic laparoscopies performed has been associated with a reduction of laparotomies with unresectable disease. **Conclusion:** Although classically associated with a poor prognosis, therapeutic results for PSM treatment are progressively improving. Implementing existing treatment and identifying new ones is a future challenge to ensure the best treatment for this patients.

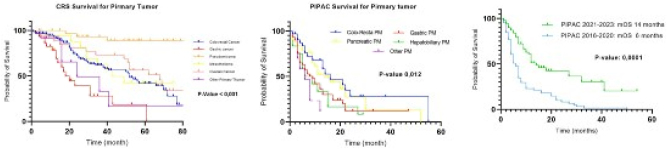



### PO 168 Rationale and Study Design of the KOV-HIPEC-04: A Phase III Randomized Controlled Trial in Primary Stage Three and Four Ovarian Cancer After Interval Cytoreductive Surgery (FOCUS) #PO 168

#### Oral communication or poster

M.C. Lim^1^, J.H. Kim^1^, J.K. Bae^1^, U. Kim^1^, J. Kim^1^, E. Woo^1^, H. Koh^1^, Y. Woo^1^, S.Y. Park^1^

^1^National Cancer Center - Goyang-si, Gyeonggi-do (Korea, Republic of)

**Abstract**

**Background:** The addition of hyperthermic intraperitoneal chemotherapy (HIPEC) during interval cytoreductive surgery increases progression-free and overall survival for patients with stage III ovarian cancer in two randomized controlled trials (OV-HIPEC-01 and KOV-HIPEC-01). This trial aims to identify the survival benefit of HIPEC in stage III & IV ovarian cancer in the era of maintenance therapy of bevacizumab and/or PARP inhibitors.

**Methods:** Ovarian cancer patients will be randomized at the time of interval cytoreductive surgery with achieving complete cytoreduction or cytoreduction with no more than 2.5mm size of residual disease to receive HIPEC (41.5 cisplatin 75mg/m2, 90 minutes) or not (control arm). After recovery from surgery, patients will receive postoperative platinum-based adjuvant chemotherapy followed by maintenance therapy with PARP inhibitor or bevacizumab. The primary objective of the trial is to evaluate OS in two groups. Secondary objectives are PFS, cancer-specific survival, time to first subsequent therapy (TFST), safety, CA-125 KELIM, and quality of life. Assuming that the enrollment period is 5 years and the follow-up period is 3 years, the total number of events required is 263. Based on the log-rank test, the total number of subjects required to prove HR 0.67 with a two-sided alpha of 0.05 and 90% power is 494. 520 patients are finally studied, considering 5% drop-out.

**Results:** There are no available results at the time of submission

**Conclusions:** The role of HIPEC during interval cytoreductive surgery will be discovered in stage III & IV ovarian cancer with this randomized trial (KOV-04) for the first time.

KOV-HIPEC-04

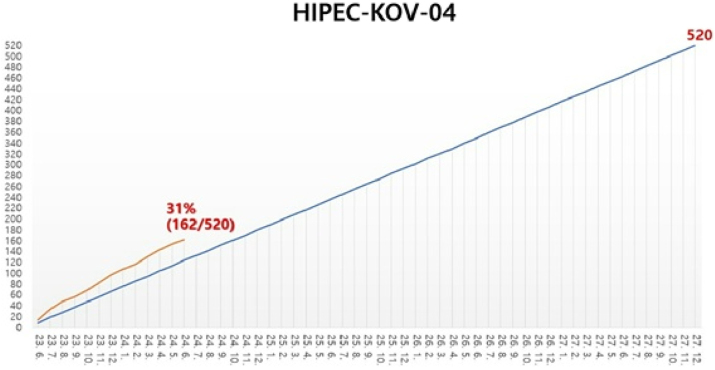



### PO 170 PIPAC. Starting a Program in Chile. Indications and Results. #PO 170

#### Oral communication or poster

M. Uribe^1^, C. García^1^, C. Luis^1^, R. Carlos^1^

^1^Clínica MEDS - Santiago (Chile)

**Abstract**

Aim. To report the experience of the interdisciplinary carcinomatosis team at MEDS Clinic in Santiago, Chile, indications and results. Methods. We report the data of all consecutive patients summited to PIPAC in our center. All cases were discussed in multidisciplinary committees. Results. We performed 25 PIPAC procedures in 15 patients, 12 women and 3 men. Median age was 57.1 years old (38-74). The origin was colon in 4, ovary in 3, pseudomyxoma, gastric and pancreas in 2 and breast and uterine in one. Median PCI was 14. Drugs used were Oxaliplatin plus Doxorubicin in usual doses. One patient with gastric cancer had 3 PIPAC, the first for cytoreduction with good results. She was planned for resectable surgery but she was lost of follow-up. One patient with pancreatic carcinomatosis had 3 PIPAC he was lost of follow-up. Currently he has metastatic disease. A 74-year-old female has had 3 episodes of PIPAC with excellent regional control of the carcinomatosis. One patient with colonic cancer was subjected to exploratory laparoscopy plus PIPAC. Was considered resectable and complete cytoreduction plus HIPEC was performed. For all patients in whom the indication of PIPAC was refractory ascites, this subsided (100%). Admission time was less than one day. The longest survival of palliative patients was 9 months. One patient with pancreatic carcinomatosis is alive, with metastatic disease after 4 years. Patients in whom we have done PIPAC in the last 6 months are alive with symptoms controlled. Comments. PIPAC is a procedure not widely diffused in our country. We report encouraging results. Is important to clearly decide the intention of treatment; cytoreduction with the objective of resection and probably HIPEC, or in a palliative setting. In our experience refractory ascites was controlled in 100% of the cases. We have no complications and a shorty admission time.

### PO 171 The Impact of KRAS Mutation Status on Postoperative Outcomes for Patients Undergoing Cytoreductive Surgery for Peritoneal Carcinomatosis of Appendiceal Origin #PO 171

#### Oral communication or poster

P. Borowsky^1^, M. Montealegre^1^, K. Gomez^1^, A. Hernandez^1^, C. Cash^1^, S. Yadegarina^2^, P. Joshi^3^, H. Bahna^2^, M. Moller^4^

^1^University of Miami - Miami (United States) - Miami (United States),

^2^University of Miami/JFK - Miami (United States),

^3^University of Miami - Miami (United States),

^4^University of Chicago - Chicago (United States)

**Abstract**

**Introduction:** KRAS mutations correlate with poor prognosis for intraabdominal malignancies. Less is known about how KRAS impacts postoperative outcomes following hyperthermic intraperitoneal chemotherapy (HIPEC) for peritoneal carcinomatosis of appendiceal origin (PCA). This study assessed the impact of mutant-type KRAS tumors on postoperative morbidity and mortality for patients undergoing HIPEC for PCA.

**Methods:** Patients were prospectively enrolled from 2017-2024. KRAS status was stratified into mutant versus wildtype groups. Sociodemographic and clinical differences between groups were compared using chi squared or t-tests. Logistic and linear regression analyses were performed to determine the impact of KRAS on postoperative outcomes, controlling for age, gender, race, ethnicity, tumor stage, grade, and peritoneal cancer index (PCI) score.

**Results:** 97 patients (32 [33%] mutant and 65 [67%] wildtype KRAS) were included. Mean age was 52.9 ± 10.6 years. Most patients were female, White, Hispanic, and had ECOG status of 0. 86 (89%) tumors were mucinous, 49 (57%) of which were low grade neoplasms and 61 (71%) of which presented with pseudomyxoma peritonei. Mean PCI score was 18 ± 10 (range 0-37). 58 (60%) patients had R0 resection. Mean intensive care unit (ICU) and hospital length of stay (LOS) were 5.2 and 11 days. Median follow up time was 36.9 (range 160.8) months. 25 (26%) patients had disease recurrence. Median recurrence-free and overall survival were 32.9 and 37.2 months. Overall, mutant and wildtype groups had comparable postoperative outcomes, with no differences in Clavien-Dindo classification or RR scores, ICU or hospital LOS, and recurrence or death rates.

**Conclusion:** Although KRAS mutations often imply poor prognosis, this study found that patients with KRAS-mutant PCA had comparable postoperative morbidity and mortality compared to those without when treated with HIPEC. This may imply therapeutic benefit from HIPEC for patients with KRAS-mutant PCA, potentially mitigating the adverse prognosis associated with this mutation.

### PO 172 The Impact of Peritoneal Carcinomatosis Index Reduction on Post-Operative Morbidity and Recovery in Patients with Peritoneal Surface Malignancy from Appendiceal Origin after CRS/HIPEC #PO 172

#### Oral communication or poster

C. Cash^1^, A. Hernandez^1^, M. Montealegre^1^, P. Borowsky^1^, S. Yadegarina^2^, K. Gomez^1^, H. Bahna^2^, M. Moller^3^

^1^University of Miami - Miami (United States),

^2^University of Miami/JFK - Miami (United States),

^3^University of Chicago - Chicago (United States)

**Abstract**

**INTRODUCTION:** Peritoneal Carcinomatosis Index (PCI) represents extent of peritoneal involvement during cytoreductive surgery (CRS) and hyperthermic intraperitoneal chemotherapy (HIPEC). PCI is a strrong prognostic indicator for outcomes in primary appendiceal neoplasms. We sought to evaluate the relationship between PCI reduction during CRS/HIPEC and post-operative morbidity in patients with primary appendiceal neoplasms.

**METHODS:** 276 patients were prospectively enrolled from 2002-2023 and underwent CRS/HIPEC. 98 had primary appendiceal neoplasms. Patient demographics, tumor characteristics, and perioperative outcomes were collected. Post-operative morbidity was represented by Clavien-Dindo (CDC) score. PCI reduction was calculated using the difference between PCI at exploration and post-operative PCI. Spearman’s Rho was used to assess the correlation between PCI reduction, CDC score, hospital length of stay (HLOS), and Intensive Care Unit length of stay (ICULOS). Linear regression was used to ascertain the magnitude of PCI reduction on HLOS, ICULOS, and CDC score.

**RESULTS:** The patient cohort consisted of 61 (62.2%) females and 35 (35.7%) males. Mean age was 52.8 ± 10.5 years. Appendiceal tumors consisted of 58.2% well-, 16.3% moderately-, and 15% poorly-differentiated. Median ICULOS and HLOS were 3 and 8 days. Mean PCI at exploration was 16.7 (range 2-37). Greater PCI reduction positively correlated with ICULOS (r=0.4, p<0.001) and HLOS (r=0.281, p=0.007), but did not correlate with CDC Score (r= 0.130, p= 0.214). There was a significant increase in ICULOS (β=0.305, CI=0.07 – 0.44, p<0.001) and HLOS with a greater PCI reduction (β=0.265, CI=0.058 – 0.442, p=0.011), but no significant increase in CDC Score (β=0.161, CI=-0.004-0.04, p=0.124).

**CONCLUSIONS:** Our findings suggest greater PCI reduction is a stronger indicator of recovery in patients with primary appendiceal neoplasm undergoing CRS/HIPEC, likely due to superior correlation to magnitude of surgical debulking. However, greater PCI reduction was not associated with increased post-operative morbidity.

### PO 173 Outcomes of Pressurized Intraperitoneal Aerosol Chemotherapy (PIPAC) for Unresectable Peritoneal Metastasis: Insights from a Single-Center Study #PO 173

#### Oral communication

J.P. Vieira De Sousa^1^, T. Bouça-Machado^1^, J. Nogueiro^1^, F. Gonçalves^1^, S. Meireles^2^, N. Tavares^2^, A. Costa^2^, B. Viamonte^3^, J. Lopes^4^, M. Aral^1^

^1^General Surgery Department, Local Health Unit of São João - Porto (Portugal),

^2^Department of Medical Oncology, Local Health Unit of São João - Porto (Portugal),

^3^Department of Radiology, Local Health Unit of São João - Porto (Portugal),

^4^Department of Pathology, Local Health Unit of São João - Porto (Portugal)

**Abstract**

**Background:** Pressurized intraperitoneal aerosol chemotherapy is a novel technique for treating unresectable peritoneal carcinomatosis, either alone or with systemic chemotherapy. This study aimed to evaluate the initial experience of PIPAC in a Portuguese center specialized in hyperthermic intraperitoneal chemotherapy.

**Methods:** A retrospective analysis of a PIPAC database was conducted from June 2022 to May 2024 at a tertiary hospital. Variables analyzed included primary tumor, ascites volume, PCI, chemotherapy regimen, pathological response, morbidity, length of hospital stay, and mortality.

**Results:** Fifty-six PIPAC procedures were performed on 23 consecutive patients with ECOG-PS≤2. Seventy percent were male and median age at first PIPAC was 65 years. Fourteen (58.3%) had gastric primary tumor and 5 (20.8%) had appendix tumor. Median PCI was 12 (range:3-39); 47.8% had at least three PIPAC procedures, and the mean PIPAC number was 2 (range:1-6). The non-access rate was 5.1%. Median hospital stay was 1 day (range:1–29). Six complications were recorded, with major complications (CTCAE3-4) occurring in four procedures (7.1%), and severe complications (Clavien–Dindo>3a) in three procedures (5.4%). There were two reoperations and two deaths within 30 days. Median overall survival (mOS) from diagnosis was 13.1 months (range:2.0-42.5), and 5.3 (range:0.4-16.3) from the first PIPAC. Excluding cases with high PCI (>26), mOS from diagnosis was 20.3 months (range:4.4-42.4). PRGS was used in 21.6% of PIPACs with a mean score of 2 (range:1-2). The primary reasons for discontinuing PIPAC treatment were disease progression (41.8%) or technical issues (20.8%). Two patients underwent curative surgery after a bidirectional approach achieving CC0.

**Conclusions:** PIPAC seems to be a feasible and safe treatment for peritoneal carcinomatosis, with low morbidity and short hospital stays. It is suitable for patients with peritoneal carcinomatosis not initially eligible for surgery to reduce tumor invasion or palliate symptoms, notably ascites. Contraindications are bowel obstruction and multiple intraabdominal adhesions.

### PO 174 Zero renal impairment post Cytoreductive surgery plus hyperthermic intraperitoneal chemotherapy with Cisplatin #PO 174

#### Oral communication

A. Abdulrahem^1^, A. Alyami^1^, M. Alzamanan^1^, S. Almowallad^1^, M. Fagihi^1^, Q. Alqurashi^1^, R. Alwadai^1^, A. Saihb^1^, M. Alhajlan^1^, M. Alyami^1^

^1^king khalid hospital - NAJRAN (Saudi Arabia)

**Abstract**

**Background:** Cytoreductive surgery combined with hyperthermic intraperitoneal chemotherapy (HIPEC) has been shown to provide benefits in the management of peritoneal Carcinomatosis . Cisplatin (CDDP) is one of the most frequently used drugs for peritoneal infusion but it renal toxicity and acute renal failure. The aim of the present study was to assess the impact of sodium thiosulfate (ST) as preventive method for renal impairment (RI) following HIPEC with CDDP.

**Methods:** This prospective study assessed the RI rates for all patients who underwent HIPEC with CDDP in king Khalid hospital Najran, Saudi Arabia between august 2019 and April 2024. All patients received an ST infusion at 9 mg/m2 prior to HIPEC and at 12 mg/m2 at the end of the procedure. RI was defined by postoperative serum creatinine >1.6 times elevation of baseline value.

**Results:** 39 patients underwent CRS and HIPEC with CDDP. 33 Ovarian (84.6%), 4 gastric (10.3%) , 2 multicystic mesothelioma (5.1%). (0%) developed RI

**Conclusion:** ST appears to be an effective drug for the prevention of the renal toxicity of CDDP used for HIPEC and should be used for all such procedures.

### PO 175 laparoscopic peritonectomy and HIPEC for the treatment of peritoneal metastasis #PO 175

#### Oral communication

M. Alzamanan^1^, A. Alyami^1^, A. Abdulrahem^1^, M. Fagihi^1^, S. Almowallad^1^, Q. Alqurashi^1^, R. Alwadai^1^, A. Saihb^1^, M. Alhajlan^1^, M. Alyami^1^

^1^king khalid hospital - NAJRAN (Saudi Arabia)

**Abstract**

**Background:** Minimal invasive surgery is an new era for CRS and Hipec. Less hospital stay , less pain and better QoL. Our aim is to present our experience on laparoscopic peritonectomy and laparoscopic HIPEC in a newly establish center.

**Methods:** A retrospective analysis for patients underwent laparoscopic CRS and HIPEC from march 2019 to April 2024 at the King Khaled Hospital (Najran, Saudi Arabia). The Hospital Data base include all clinical relevant data, surgical procedure, outcome criteria as overall survival and adverse events According to (CTCAE) version4.0. All procedures were done by well- trained surgeons.

**Results:** Excluding cases converted to open due to adhesions, 6 patients were analyzed: 4 of them were females and median age was 43 years. PM were from appendix, colorectal and ovarian cancer for 3, 2, and 1 patient respectively. Median PCI was 8 (3-18). Median time of surgery (excluding HIPEC time) was 8 hours (4.5-10.5). The median LOS in the hospital was 8 days . All patients underwent resection of the primary tumor, parietal peritonectomy. One of them total pelvic peritonectomy plus salpingo-oophorectomy with hysterectomy Two patients underwent bowel resection with anastomosis. Three patients had complications: grade IV (hemoperitoneum) grade III (intestinal obstruction) grade II (port site hernia). One patient died from covid-19 disease with severe pneumonia two months after surgery. The other five patients are alive without recurrence after a median follow-up of 18 months.

**Conclusion:** Total laparoscopic peritonectomy and HIPEC is feasible and safe, with shorter LOHS and the same or lower rate of complications compared to open CRS and the same oncologic results. In experienced centers it seems reasonable to offer our patients all the benefits of MIS when indicated

### PO 176 Initial experience with Robotic Assisted CRS-HIPEC #PO 176

#### Oral communication

A. Alyami^1^, M. Alzamanan^1^, A. Abdulrahem^1^, S. Almowallad^1^, M. Fagihi^1^, Q. Alqurashi^1^, R. Alwadai^1^, A. Saihb^1^, M. Alhajlan^1^, M. Alyami^1^

^1^king khalid hospital - NAJRAN (Saudi Arabia)

**Abstract**

**Background:** (CRS-HIPEC) as a treatment for peritoneal tumors has been accepted as a recommended therapy for select patients according to the latest guidelines from the NCCN. Traditionally, it has been performed through an open, midline incision. The minimally invasive surgical approach to CRS-HIPEC offers several benefits, including less post-operative pain, decreased blood loss, and shorter post-operative length of stay.

**Methods:** This is the first experience of Robotic Assisted CRS-HIPEC in the middle east for peritoneal tumors

We report 3 cases done in King Khalid Hospital in Najran, Saudi Arabia

**Results:** Between 8-2019/2-2024 , 3 Robotic CRS and HIPEC. 2 female and the age range between (31-50). low grade appendicular mucinous neoplasm with PMP for 2 patients and 1 colon PM . PCI range between (1-2) and the median duration of the surgery was 6 hours. Hospital; stay was 2 days for 2 patient and 7 days for the 3rd one . 1 grade III complication: subcutaneous emphysema and cellulitis secondary to chemotherapy that was treated simply with antibiotics and analgesia. Follow up , 29,25,and 2 months respectively: all of them are a live without recurrence

**Conclusion:** We present for the first time the feasibility of a Robotic-Assisted CRS-HIPEC in the middle east for peritoneal surface malignancies. Such approach need further prospective study to show the feasibility of Robotic-Assisted surgery for CRS-HIPEC.

### PO 177 pressurized intraperitoneal aerosol chemotherapy (PIPAC) for the management of unresectable peritoneal metastasis , experiance from Saudi arabia #PO 177

#### Oral communication

M. Alzamanan^1^, S. Almowallad^1^, A. Alyami^1^, A. Abdulrahem^1^, M. Fagihi^1^, Q. Alqurashi^1^, R. Alwadai^1^, A. Saihb^1^, M. Alhajlan^1^, M. Alyami^1^

^1^king khalid hospital - NAJRAN (Saudi Arabia)

**Abstract**

**Background:** PIPAC is a novel drug delivery system able to induce regression of peritoneal metastasis (PM) in the salvage situation. It is a recent approach with promising results for patients with non-operable peritoneal metastasis (PM). We aimed to evaluate survival and postoperative outcome of patients with unresectable PM from different origins treated with chemotherapy and PIPAC.

**Methods:** Retrospective analysis of a prospective maintained PIPAC database . PIPAC with Cisplatin 10.5 mg/m2 and doxorubicin 2.1 mg/m2 for Gastric and Ovarian PM, PIPAC Oxaliplatin 120 mg/m2 for Colorectal PM. Administrated over 30 min at 6 -week intervals . Outcome criteria , Overall survival and Adverse events according to (CTCAE) version4.0

**Results:** 56 PIPAC were done in 18 consecutive patients, 10 Patients were male (62.5%), PM origin was from Colorectal (6) Gastric (9) Ovarian (3) Median consecutive PIPAC procedures were 3 (1-7). At the first PIPAC, median age was 40 years (31-79) . Median PCI was 21 (10-39). All patients had systemic chemotherapy alternating with PIPAC. Six patients (33%) underwent more than 2 lines of pre-PIPAC chemotherapy

•Multiple Procedures. 14 Patients (77.8%) underwent 2 PIPAC, 11 Patients (61.1%) underwent 3 PIPAC , 6 Patients (33.3%) underwent 4 PIPAC , 4 Patients (22.2%) underwent 5 PIPAC , 2 Patients (11.1%) underwent 6 PIPAC , 1 Patient (5.5%) underwent 7 PIPAC. Overall and major complications CTCAE – III occurred in 3 procedures (5.3%) CTCAE -IV occurred in 1 procedure (1.7%). Median survival , Colorectal = 16 (8 – 69 ) months , Gastric = 20 (5 – 24) months Ovarian = 16 (15 - 48) months , 10 patients (55.5%) still alive on active treatment

**Conclusion:** PIPAC is safe and feasible in association with systemic chemotherapy for unresectable PM in newly established center. Survival data are encouraging and justify further clinical studies

### PO 178 Immediate peritoneal immediate chemotherapy (EPIC): an obsolete technic ? Analysis of series of 300cases. #PO 178

#### Oral communication

S. Berkane^1^

^1^Faculté De Médecine D’alger. Service De Chirurgie Viscérale Et Oncologique. Hôpital De Bologhine. Ibn Ziri. Alger - Alger (Algeria)

**Abstract**

**Introduction:** Early peritoneal immediate chemotherapy (EPIC) is a therapeutic approach which is associated with surgery for cancers of the peritoneal cavity, either locally advanced or metastatic. Purpose of the study, is the analysis of the immediate and long term results of this approach. **Material and method:** EPIC is used in our department as adjuvant, neoadjuvant or palliative (Symptomatic purpose) in the management of cancers of the peritoneal cavity. These cancerous diseases are represented by locally advanced and metastatic periotneal cancers. **Results:** We collected 300 cases. There were 176 women (58.7%) and 124 men (41.3%) with an average age of 48 years (16 to 89 years). The etiologies are represented by colonic, rectal, ovarian, gastric, appendicular, gallbladder and miscellaneous cancers. One hundred and forty-nine patients (49.7%) presented with peritoneal carcinomatosis and 151 (50.3%) had locally advanced disease. The adjuvant approach involved 140 patients (46.7%), the neoadjuvant approach in 80 patients (26.7%) and palliative in 80 patients (26.7%). Ninety-seven patients (17.7%) presented with complicated surgical outcomes, including 33 fatalities (11%). The most severe complications are represented by anastomotic fistula and renal and hematological complications in patients pretreated with systemic chemotherapy (65%). Eleven patients (03.7%) benefited from iterative remote CIPPI. One hundred and seventeen patients (39%) had complete control of their cancer after an adjuvant or neoadjuvant approach to CIPPI. Overall, survival at 3 years and at 5 years is respectively 125/250 (50%) and 104/252 (41.6%). **Conclusion:** In our experience, EPIC remains a useful technique both in the neoadjuvant approach, in the adjuvant approach and with palliative purposes (symptomatic: relieving the patient of their pain and ascites). It can also be used for peritoneal tumor recurrences, providing greater control of the cancerous disease.

## Open proposals linked to peritoneal surface maligancies management

### PO 179 Elucidating molecular mechanisms of Peritoneal Carcinomatosis in Colorectal Cancer through multi-omic analysis: integration of clinical and genomic profiling for enhanced detection of minimal residual disease #PO 179

#### Oral communication or poster

V. Martelli^1^, J. Vidal^1^, J. Badia-Ramentol^2^, J. Linares^1^, S. Salvans^3^, B. Bellosillo^4^, A. Calon^2^, M. Iglesias^4^, M. Pascual^3^, C. Montagut^1^

^1^Medical Oncology Department, Hospital del Mar, Hospital del Mar Research Institute, Universitat Pompeu Fabra, CIBERONC, Barcelona, Spain - Barcelona (Spain),

^2^Cancer Research Program, Hospital del Mar Medical Research Institute (IMIM), Barcelona, Spain - Barcelona (Spain),

^3^Department of Surgery, Section of Colon and Rectal Surgery, Hospital del Mar, Barcelona, Spain - Barcelona (Spain),

^4^Pathology Department, Hospital del Mar, Hospital del Mar Research Institute, Universitat Pompeu Fabra, CIBERONC, Barcelona, Spain - Barcelona (Spain)

**Abstract**

**Background:** Peritoneal carcinomatosis (PC) from colorectal cancer (CRC) represents a significant challenge due to its aggressive nature, complex treatment options, and the lack of strong prognostic and predictive biomarkers. Liquid biopsy is a minimally-invasive approach to explore real-time tumor molecular biology, assess minimal residual disease (MRD) and investigate tumor-derived factors in blood.

**Aim:** We aim to develop a multi-omic signature for MRD detection and relapse prediction by integrating circulating tumor DNA (ctDNA) analysis in peripheral blood and peritoneal fluid along with stroma-derived biomarkers after the analysis of cancer-associated fibroblasts (CAFs) derived from metastatic CRC (mCRC) patients with PC, candidates to cytoreductive surgery and hyperthermic intraperitoneal chemotherapy (CRS + HIPEC).

**Methods:** The study enrolled mCRC patients with PC undergone to CRS + HIPEC. Blood samples from peripheral vein, tumor draining vessels, and peritoneal fluid samples were collected for ctDNA analysis. Next-generation sequencing (NGS) analysis was performed in tissue samples from primary tumors. Fresh tissue was collected from primary tumors and peritoneal metastases to derive and characterize CAFs in search of tumor biomarkers.

**Results:** Overall, we have enrolled 30 microsatellite stable mCRC patients. The most frequent primary tumor location was right colon (N = 13; 43%), and intestinal-type adenocarcinoma the predominant histological type (N = 15; 50%). Tissue NGS revealed *RAS* mutations in N = 20 (67%) patients, followed by *TP53* mutations (N = 6; 20%). *BRAF* mutations occurred in two cases, with uncommon variants (*G506C*, *V600N*). Additional mutations were found in *PALB2* (N = 1; 3%), *POLE* (N = 1), and *HER2* (N = 1). We have currently derived 23 CAFs from 20 patients.

**Conclusions:** This open proposal addresses the need for improved prognostic markers in PC patients. Through advanced molecular techniques, we aim to better understand MRD after CRS and ultimately improve clinical outcomes in this challenging population.

### PO 180 Exposure assessment of oxaliplatin between systemic and intraperitoneal route including PIPAC and HPIPAC (hyperthermic PIPAC) using swine experimental model #PO 180

#### Oral communication or poster

S. Lee^1^, H.H. Kim^2^, D.Y. Kim^3^, S.K. Bae^3^, D.K. Lee^4^, Y. Jung^4^, M. Yoo^5^, C. Yoon^1^, C. Bang^6^, J. Park^1^

^1^Graduate School of Public Health, Seoul National University - Seoul (Korea, Republic of),

^2^Chung-Ang University Gwangmyeong Hospital - Gwangmyeong (Korea, Republic of),

^3^College of Pharmacy, The Catholic University - Bucheon (Korea, Republic of),

^4^College of Pharmacy, Chung-Ang University - Seoul (Korea, Republic of),

^5^Asan medical center - Seoul (Korea, Republic of),

^6^Seoul National University Bungang hospital - Seongnam (Korea, Republic of)

**Abstract**

**Background:** We have developed a novel intraperitoneal drug delivery system, HPIPAC (Hyperthermic Pressurized Intraperitoneal Aerosol Chemotherapy), which combines the gas-based HIPEC (Hyperthermic Intraperitoneal Chemotherapy) and PIPAC (Pressurized Intraperitoneal Aerosol Chemotherapy) methods. The study evaluates medical staff exposure to oxaliplatin, an antineoplastic drug, during peritoneal cancer treatments using three methods: Intravenous therapy (IV), Pressurized Intraperitoneal Aerosol Chemotherapy (PIPAC), and Hyperthermal Pressurized Intraperitoneal Aerosol Chemotherapy (HPIPAC) in swine models.

**Methods:** We administered 5 mg/kg of oxaliplatin around 25 Kgr swine for each group. Environmental samples (surface and air) were collected and analyzed for platinum, indicating oxaliplatin presence.

**Results:** Airborne platinum levels were mostly below the detection limit unless a leak occurred during surgery. Surface contamination was more common, especially on floors and injectors during PIPAC and hPIPAC, with detectable platinum on surgical gloves ranging from 2.7% in IV to 20.2% in HPIPAC. Platinum was not detected in the experimenter’s urine and blood.

**Conclusions:** The risk of inhaling oxaliplatin is low if there are no leaks. However, surface exposure suggests that medical staff are at risk, highlighting the need for precautionary measures despite the lack of specific legal exposure standards for oxaliplatin.

sampling site

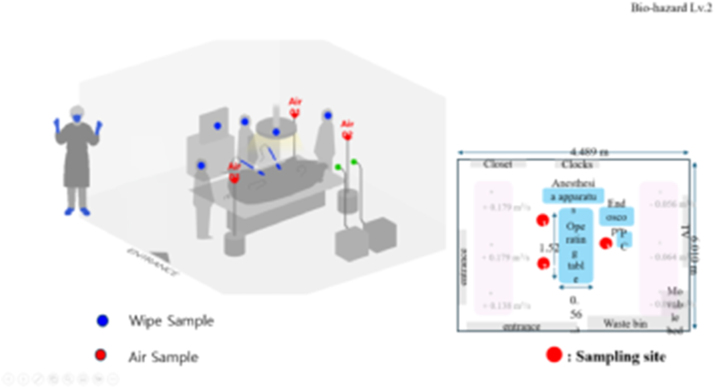



### PO 181 A multicenter phase I study of pressurized intraperitoneal aerosolized chemotherapy (PIPAC) nab-paclitaxel and cisplatin with systemic nab-paclitaxel in recurrent ovarian cancer patients - Trial in progress #PO 181

#### Oral communication or poster

T. Dellinger^1^, N. Ruel^1^, P. Frankel^1^, S. Yost^1^, S. Chang^1^, E. Wang^1^, J. Villella^2^, T. Dinh^3^, M. Raoof^1^

^1^City of Hope National Medical Center - Duarte (United States),

^2^Northwell Health - New York (United States),

^3^Mayo Clinic - Jacksonville (United States)

**Abstract**

**Background:** Nab-paclitaxel is a standard of care option for platinum-resistant recurrent ovarian cancer (PROC) patients. Intraperitoneal and intravenous (IV) combinations of cisplatin and paclitaxel are active regimens in first-line ovarian cancer treatment. The combination of PIPAC nab-paclitaxel and cisplatin, followed by systemic nab-paclitaxel has not yet been explored in ovarian cancer (OC).

**Methods:** This is a multicenter phase 1 trial (NCT04329494) evaluating safety and feasibility of PIPAC nab-paclitaxel and cisplatin in combination with systemic weekly nab-paclitaxel, in recurrent OC patients who are not candidates for cytoreductive surgery. Patients must be at least 6 months post first-line standard-of-care chemotherapy with no bowel obstruction. Treatment for each 28-day cycle consists of PIPAC cisplatin (15 mg/m2) and nab-paclitaxel (90 mg/m2) on Day 1, followed by IV nab-paclitaxel 100mg/m2 Day 8 and 15 (Fig. 1). A dose de-escalation plan is provided if the starting dose is not well-tolerated. Primary endpoints include dose-limiting toxicities and adverse events by CTCAE v5.0. Secondary endpoints include CT imaging Response Evaluation Criteria in Solid Tumors (RECIST), treatment response based on Intraoperative Peritoneal Carcinomatosis Index (PCI), and Pathologic Peritoneal Regression Grading Score (PRGS). Follow-up after treatment completion will be every 12 weeks. Statistical modeling and data analysis includes a safety lead-in incorporated into a two-stage Simon Optimal Design (IQ 3+3). Enrollment is currently ongoing.

**Conclusions:** This study will establish the feasibility of the combination of PIPAC (cisplatin and nab-paclitaxel) and systemic IV nab-paclitaxel in recurrent OC patients, and evaluate efficacy signal to determine a potential subsequent Phase II trial.

PIPAC + systemic therapy for OC patients

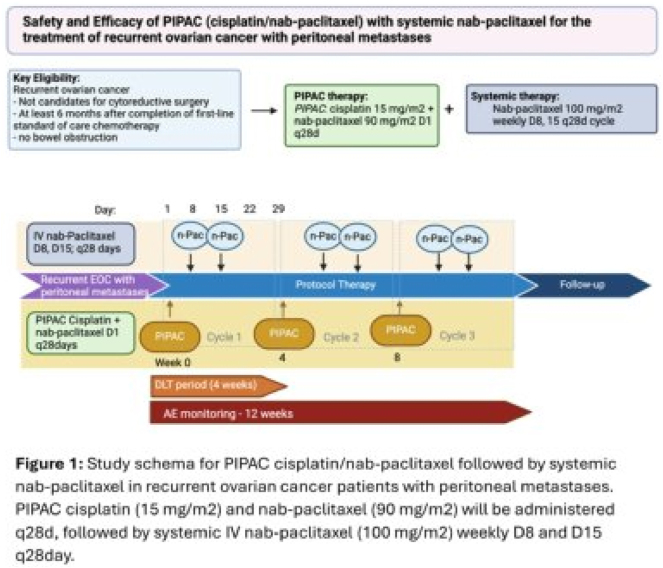



### PO 182 Urological Organ Resections in Cytoreductive Surgery and Hyperthermic Intraperitoneal Chemotherapy #PO 182

#### Oral communication

P.O. Özcan^1^, O.D. Duzgun^2^

^1^Istanbul University Cerrahpasa Medical Faculty - istanbul (Turkey),

^2^Umranıye Research and Training Hospital - istanbul (Turkey)

**Abstract**

**Background:** In cytoreductive surgery and hyperthermic intraperitoneal chemotherapy (CRS+HIPEC), involvement of urological organs (bladder, ureter) were previously considered inoperable. However, advancements in cancer surgery over the past decade have led to discussions regarding the feasibility of these organ resections in appropriate cases, which are now successfully performed in high-volumed centers. The aim of this study is to present the feasibility of urological organ involvement.

**Methods:** Cases of intraabdominal malignancies with peritoneal carcinomatosis (PC) who underwent CRS+HIPEC between May 2016 and March 2024 were retrospectively analyzed. Patients who underwent surgical interventions related to the urinary system were separated. Demographic data, diagnoses of urological resections and interventions performed, and postoperative morbidity-mortality were evaluated for these cases.

**Results:** Out of total 410 cases undergoing CRS+HIPEC, urological system interventions were performed in 72 (17.5%) cases. Preoperatively, 24 (33.3%) patients were diagnosed with ovarian cancer, 23 (31.9%) with colorectal cancer, 11 (15.2%) with gastric cancer, 7 (9.7%) with sarcomatosis, 6 (8.3%) with mesothelioma, and 1 (1.4%) case with cervical cancer. The mean Peritoneal Cancer Index (PCI) score was 10 (range 3-22). Complete cytoreduction (CC 0) was achieved in 66 (91.6%) cases, while 6 cases had a CC 1 score. A total of 63 cases underwent cystectomy (5 total, 58 partial). Among the cases undergoing partial cystectomy, ureteroneocystostomy was performed in 24 cases and ureteroureterostomy in 9 cases. Four cases underwent total nephrectomy and 1 case underwent partial nephrectomy. Urological complications of Clavien–Dindo grade 3 or higher were observed in 7 (9.7%) cases. Urinary leakage was detected in 6 cases, one case developed bladder necrosis. . Mortality occurred in 2 (2.7%) cases due to bladder ischemia and pulmonary embolism.

**Conclusions:** Urological organ involvements and reconstructions in CRS+HIPEC can be performed with acceptable morbidity-mortality rates in high-volumed experienced tertiary centers.

### PO 183 It is what the surgeon does not see that kills the patient #PO 183

#### Oral communication or poster

P. Sugarbaker^1^

^1^Washington Cancer Institute - Washington (United States)

**Abstract**

**Background:** There is a variable number of colon cancer patients, estimated at approximately 10%, who present with advanced disease. If these patients are treated by the current conventional standard of care, the likelihood for treatment failure is extremely high. These are not patients with known disseminated disease but patients who are at high risk for recurrent disease unless special treatments are initiated preoperatively and intraoperatively. Clinical features of these patients are shown in Table 1.

**Methods**

**Table:** 

Evaluation	Score
***Preoperative CT with oral contrast***	
Bowel wall invasion more than 5 mm beyond muscularis propria (T3 or T4)	25%
Lymph node or vascular invasion	25%
Tumor diameter ≥5 cm or encroachment on adjacent organs or structures	25%
***Tumor markers CEA, CA19-9, or CA-125***	
Any tumor marker >3 times the upper limit of normal	25%
More than one tumor marker in the abnormal range	25%
***Colonoscopic findings***	
Aggressive histology by biopsy	25%
Circumferential involvement of the bowel wall	25%
***Symptoms***	
Symptoms present including palpable mass, pain, obstruction, localized perforation or weight loss	25%

**Results:** Patients identified at a high-risk score of ≥ 100% require neoadjuvant chemotherapy. At the time of surgery, a thorough exploration of all occult peritoneal spaces for metastatic disease needs to be performed. A modified cytoreductive surgical procedure along with the colon resection is performed. This includes the greater omentum, ovaries and Fallopian tubes in postmenopausal women. Peritonectomy is used to create a shroud around the tumor so that all peritoneum that has been in direct contact with the tumor surface is resected. If peritoneal metastases are documented at any site, HIPEC should be included as part of the treatment package.

**Conclusions:** Patients at high risk for recurrence will have an improved outcome with proper preoperative evaluation, preoperative neoadjuvant chemotherapy, and a revised intraoperative management strategy.

### PO 184 Potential role of Intraoperative Peritoneal Cytology in decission-making for the indication of HIPEC administration #PO 184

#### Oral communication or poster

S. González-Moreno^1^, G. Ortega-Pérez^1^, I. López-Rojo^1^, S. Núñez-O’sullivan^1^, O. Alonso-Casado^1^, J.F. García-García^2^, A. Teijo-Quintans^2^, L. Castella-Bataller^2^, C. Grillo-Marin^3^, P.H. Sugarbaker^4^

^1^Department of Surgical Oncology. Peritoneal Surface Oncology Program. MD Anderson Cancer Center. Madrid (Spain) - Madrid (Spain),

^2^Department of Pathology. MD Anderson Cancer Center. Madrid (Spain) - Madrid (Spain),

^3^Department of General Surgery. Puerta de Hierro-Majadahonda University Hospital. Madrid (Spain) MD Anderson Cancer Center España Foundation. Madrid (Spain) - Madrid (Spain),

^4^Program in Peritoneal Surface Malignancy. Washington Cancer Institute. Washington, DC (USA) - Washington, DC (United States)

**Abstract**

**Background:** The target of Hyperthermic IntraPeritoneal Chemotherapy (HIPEC) are peritoneal free cancer cells. Peritoneal cytology is a prognostic factor in established peritoneal carcinomatosis. Indications for HIPEC have been the subject of consensus statements and clinical trials. Unnecessary HIPEC may add morbidity. We hypothesize that the intraoperative availability of a peritoneal cytology report may be of help in guiding the decision to administer HIPEC or not in cases at high risk of microscopic tumor spillage.

**Methods:** We perform peritoneal cytology upon abdominal opening in every case, either on ascitic fluid or on a peritoneal lavage. We irrigate the abdominal cavity with 500 cc of normal saline, agitate, and aspirate in all four quadrants. The aspiration canister is sent to the Pathology lab for cytological analysis. Intraoperative cytology specimen processing involves a regular hematoxylin-eosin stain, and reading is available within 1 hour; for delayed reading, a Papanicolau stain is additionally performed.

**Results:** We report 3 cases at high risk of having intraperitoneal free cancer cells where intraoperative peritoneal cytology report was instrumental and factored in our decision, together with clinical context, to proceed or not with the administration of HIPEC. A positive peritoneal cytology made the indication to proceed with HIPEC and viceversa. Correlation of intraoperative and delayed readings was 100%.

**Conclusions:** A large cohort and long-term follow up are needed to validate this strategy of intraoperative peritoneal cytology -guided HIPEC in patients at high risk of microscopic-only peritoneal dissemination. Further refinement of intraoperative cytology technology to increase its yield is warranted.

**Table:** Table 1. Results

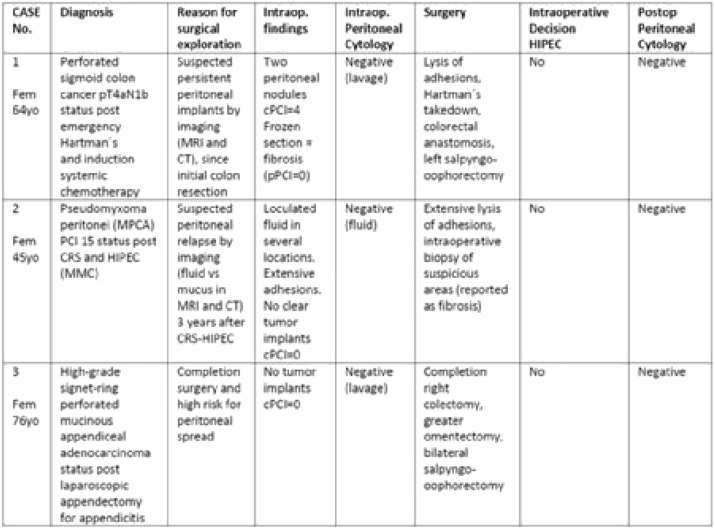



### PO 185 The Interplay of Complete Cytoreduction, HIPEC and Fibrosis in High-Grade Goblet Cell Adenocarcinomas of the Appendix with Peritoneal Carcinomatosis #PO 185

#### Oral communication or poster

P.K. Koukoutsidi^1^, T.S. Schnelldorfer^1^, M.D. Goodman^1^

^1^Tufts Medical Center - Boston (United States)

**Abstract**

**Background:** High-grade goblet cell adenocarcinomas (GCA) of the appendix behave more aggressively compared to low-grade GCA and are associated with poor prognosis. Patients with peritoneal carcinomatosis (PC) from a high-grade GCA can be referred for cytoreductive surgery (CRS) with hyperthermic intraperitoneal chemotherapy (HIPEC). In our case series, we assessed the survival of patients with high grade appendiceal GCA and PC, who underwent either CRS/HIPEC or tumor debulking.

**Methods:** A prospectively maintained database of patients treated at the Division of Surgical Oncology from 2007 to 2022 was reviewed retrospectively. Selection criteria were the histology of the primary tumor and scheduling to undergo CRS/HIPEC for PC. We calculated median overall survival from the diagnosis of PC, with focus on the attainment of complete cytoreduction (CC0/CC1) and HIPEC administration.

**Results:** 385 records were screened and 21 cases of GCA were identified. Thirteen (13) patients had a diagnosis of high-grade GCA with PC. 7/13 patients received CC/HIPEC, and 6/13 patients underwent tumor debulking. In the presence of PC, the median overall survival was 53 months for the patients who underwent CC/HIPEC and 20 months for the patients who received debulking. Of note, in the debulking group, 5/6 patients had tumors that intraoperatively exhibited characteristics of infiltration, thickening and fibrosis, confirmed by the pathology report, which rendered CC unsuccessful. In contrast, within the CC/HIPEC group, pathology reported localized fibrosis in only 2/7 patients and the achievement of CC was not impeded.

**Conclusions:** Our results demonstrate that CC/HIPEC may improve overall survival in patients with high grade GCA with PC. Notably, the presence of fibrotic tissue might affect CC success. Considering the potential impact of fibrosis on surgical outcomes, pathological assessment of fibrosis preoperatively could guide cytoreduction strategies and treatment choices in patients diagnosed with high-grade appendiceal GCA.

### PO 186 Preliminary study on feasibility of intraperitoneal continuous aerosolized fluids delivery to perform CIPAC (Continuous IntraPeritoneal Aerosolized Chemotherapy). #PO 186

#### Oral communication

M. Vaira^1^, J. So^2^, M. De Simone^3^, R. Gelmini^4^, F. Roviello^5^, O. Glehen^6^

^1^PSM Unit. General, Oncological and Emergency Surgery Unit - University Hospital of Modena - Modena (Italy),

^2^Head & Senior Consultant, Division of Surgical Oncology, Assoicate Director National University Cancer Institute, Singapore. - SINGAPORE (Singapore),

^3^PSM Program, Clinica Santa Caterina da Siena, GVM Group, Care and Research. - Torino (Italy),

^4^Head of General, Oncological and Emergency Surgery Unit - University Hospital of Modena - Modena (Italy),

^5^Head of General Surgery and Surgical Oncology, acting Director Department of Medicine, Surgery, and Neurosciences, University of Siena. - Siena (Italy),

^6^Head of General and Oncologic Surgery Department in Centre Hospitalier Lyon Sud. - Lyon (France)

**Abstract**

**Background:** HIPEC is an hyperthermic, continuous, drugs circulation performed after CRS with intent to cure peritoneal carcinomatosis (PC). PIPAC is a palliative treatment for PC, never requires CRS; it’s a laparoscopic aerosolisation of drugs, obtained by pressure, lasts for 30 mins. Drug insufflation time is 3-4 minutes over 30 minutes of the procedure. Encouraging results to shift from palliative to curative intent are reported. Single nozzle and multi nozzle PIPAC devices are described. Single nozzle shows better drugs penetration in a limited area while multinozzle ones have homogeneous distribution, but lower tissue penetration.This feature seems to be related to drugs-inflation timing, more than to pressure issues. Aim of this work is to verify if a continuous inflation, by a multinozzle device, is feasible into a cavity.

**Methods:** We designed a prototype multinozzle device operated by gas ( not by interventional radiology system) to aerosolize fluids. The system was built to perform a continuous aerosolized inflation for 30 minutes (duration of a standard PIPAC procedure. We tested the distribution of patent-blue inflated continuously into a reversed cow bladder (to mimick the intraperitoneal cavity) analyzing the blue-painted area reached by patent blue.

**Results:** Preliminary tests show that continuous aerosolized infusion is feasible, the system worked for 30 minutes and the distribution of patent blue was homogeneous into the bladder samples taken.

**Conclusions:** Multinozzle devices show better spatial aerosol distribution, but lower drugs penetration into tissues than single nozzles ones. This study was conceived to try to maintain good spatial distribution of multinozzle devices and overtake their (time-related) low drugs penetration issues by continouus aerosol inflation. Further tests are ongoing to assess if both homogeneous distribution and tissue penetration can be achieved by multinozzle continuous aerosolized inflation, in order to perform, in the future, CIPAC ( Continuous IntraPeritoneal Aerosolized Chemotherapy).

### PO 187 Overcoming Resource Constraints: Implementing a Peritoneal Surface Malignancy Program in an LMIC Setting #PO 187

#### Oral communication or poster

A. Souadka^1^, O. Lahnaoui^1^, A. Benkabbou^1^, M.A. Majbar^1^, B. El Ahmadi^2^, Z.H. Belkhadir^2^, S. Boutayeb^3^, R. Mohsine^1^

^1^Surgical Oncology Department, National Institute of Oncology, University Mohammed V, Rabat, Morocco. - Rabat (Morocco),

^2^ICU and anesthesiology Department, National Institute of Oncology, University Mohammed V, Rabat, Morocco. - Rabat (Morocco),

^3^Oncology department, National Institute of Oncology, University Mohammed V, Rabat, Morocco. - Rabat (Morocco)

**Abstract**

**Background:** The implementation of comprehensive cancer care programs in low-middle income countries (LMICs) faces numerous challenges due to limited resources and infrastructure constraints. This study explores the specific challenges encountered while implementing a peritoneal surface malignancy (PSM) management program in such a setting.

**Methods:** The program, initiated in 2021, aimed to integrate multidisciplinary care pathways for PSM patients from diagnosis through long-term follow-up. We documented and analyzed challenges related to infrastructure, personnel training, equipment availability, and interdepartmental coordination. Impact on program implementation and adaptations made in response to these challenges were also evaluated.

**Results:** Key challenges included inadequate surgical and postoperative facilities, delays in the procurement of essential medical supplies, and limited access to specialized training for staff. These factors led to variations in the adherence to intended protocols and delayed the roll-out of certain program components. Adaptive strategies included the development of in-house training sessions, adjustments in resource allocation, and increased reliance on digital technology for patient management and team communications.

**Conclusion:** The implementation of a structured PSM management program in an LMIC revealed significant systemic and operational challenges. Identifying and addressing these issues was crucial for program adaptation and continued development. The lessons learned from this experience provide valuable insights for similar healthcare settings facing comparable challenges.

### PO 188 Evaluating Peritoneal Surface Malignancy Management in North Africa: Outcomes from a Multi-Center Retrospective Audit #PO 188

#### Oral communication or poster

A. Souadka^1^, A. Makni^2^, M. Abid^3^, A. Souadka^4^

^1^Surgical Oncology Department, National Institute of Oncology, University Mohammed V, Rabat, Morocco. - Rabat (Morocco),

^2^Surgical Oncology Department, Batna Cancer Institute, Batna, Algeria.Surgical Department A, Rabta Hospital, Tunis, Tunisia. - Tunis (Tunisia),

^3^Surgical Oncology Department, Batna Cancer Institute, Batna, Algeria. - Batna (Algeria),

^4^Surgical oncology department , private center , Rabat, Morocco - Rabat (Morocco)

**Abstract**

**Introduction:** The management of peritoneal surface malignancies (PSM) poses significant challenges, particularly in regions with limited resources. This study presents a comprehensive audit of the first and only four centers managing PSM in North Africa, focusing on the implementation of cytoreductive surgery (CRS) with or without hyperthermic intraperitoneal chemotherapy (HIPEC).

**Methods:** We retrospectively analyzed data from 374 patients treated for various PSM at centers in Rabat (private and national institute), Tunis, and Batna from 2015 to 2020. The malignancies included colorectal carcinomatosis (158 cases), pseudomyxoma peritonei (147 cases), ovarian carcinomatosis IIIB-IV (43 cases), gastric carcinomatosis (22 cases), and mesothelioma (3 cases). We evaluated the extent of disease using the Peritoneal Cancer Index (PCI), with a mean score of 12. The outcomes measured were intervention length, hospital stay, major morbidity (Clavien-Dindo >3b), mortality, overall survival (OS), and disease-free survival (DFS).

**Results:** Of the total, 83 patients underwent CRS+HIPEC, while the remainder received CRS only. The mean length of surgical interventions was 296 minutes, and the average hospital stay was 11.37 days.The major morbidity rate among the patients was approximately 17.1%, and the mortality rate was about 4.8%. The survival outcomes, including OS and DFS rates, were calculated and will be discussed.

**Conclusion:** The implementation of a PSM management program in North Africa has shown promising short-term surgical and oncological outcomes despite resource limitations. This audit highlights the critical role of dedicated surgical teams and the adaptation of complex oncological procedures in low-middle income settings. The experience serves as a model for similar regions striving to improve care for patients with advanced malignancies.

### PO 189 A systematic review and meta-analyses on the efficacy of stenting for gastric outlet obstruction with peritoneal carcinomatosis #PO 189

#### Oral communication

M.D. Bin Massuryono^1^, T.H.J. Yim^2^, J.W.S. Tan^3^, C.S. Chia^4^, J.C.A. Ong^4^

^1^Department of Sarcoma, Peritoneal and Rare Tumours, Division of Surgery and Surgical Oncology, National Cancer Centre Singapore - Singapore (Singapore) - Singapore (Singapore),

^2^National University of Singapore - Singapore (Singapore),

^3^Laboratory of Applied Human Genetics, Division of Medical Sciences, National Cancer Centre Singapore - Singapore (Singapore),

^4^Department of Sarcoma, Peritoneal and Rare Tumours, Division of Surgery and Surgical Oncology, National Cancer Centre Singapore - Singapore (Singapore)

**Abstract**

**Background:** Management of malignant gastric outlet obstruction (GOO) with peritoneal carcinomatosis (PC) is challenging due to the advanced disease phase and the associated frailty of patients. Management of GOO with palliative surgery such as a gastrojejunostomy is associated with significant mortality and morbidity. Due to its efficacy and safety, stenting with self-expandable metallic stents (SEMS) is increasingly utilised. However, its role in PC is unclear due to the risk of multifocal obstruction and decreased gastrointestinal motility.

**Methods:** A systematic review and meta-analysis were conducted to clarify the role of SEMS for GOO in PC. Inclusion criteria include (1) Adult patients with GOO; (2) A group/sub-group with PC; (3) Intervention with SEMS placement; (4) Reporting of relevant outcomes e.g. success, reintervention. 10 of the 2223 studies identified were included. A descriptive synthesis and meta-analyses were conducted.

**Results:** The outcomes of 1242 patients, 578 of whom had PC, were obtained. Stenting in the absence of PC is associated with higher technical success (OR= 1.84, 95% CI 1.06-3.20) and clinical success compared to that in no PC (OR = 2.08 95% CI 1.36-3.18).

Nonetheless, stenting in PC demonstrated high technical and clinical success rates of 97.5% and 78.6%, respectively. The absence of PC is not a significant predictor of reintervention (OR = 1.29, 95% CI 0.41-4.13) and stent patency duration.

**Conclusion:** Given its high efficacy despite PC, stenting can be considered as an alternative to surgery in treating PC-associated GOO. Surgery can still be pursued if stenting fails, avoiding significant morbidity and mortality.

Forest Plots of Technical and Clinical Success of

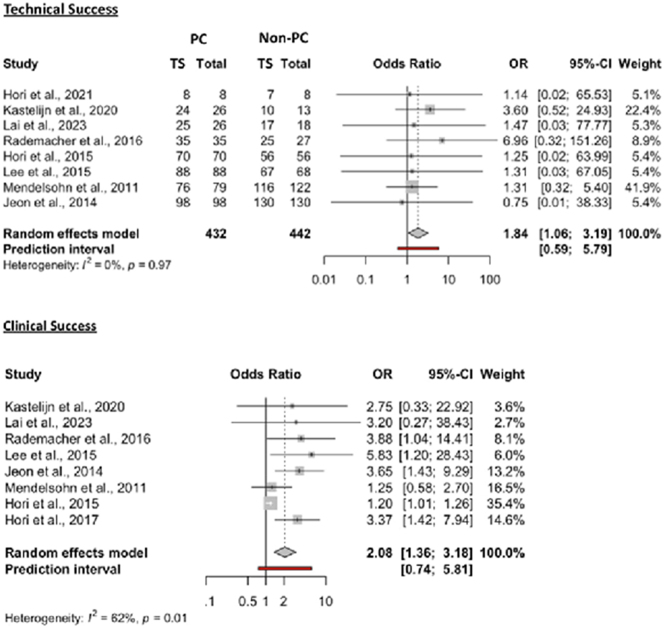



## Patient-centered care in peritoneal surface malignancies

### PO 38 Palliative Care Education for Surgical Oncologists: A Systematic Review of Current Curriculum and Educational Outcomes #PO 38

#### Poster

S.H.X. Cheok^1^, B. Paik^1^, P.L. Koh^1^, C.A.J. Ong^1^, C.J. Seo^1^, C.S. Chia^1^, M. Cai^1^, S.M.J. Wong^1^

^1^National Cancer Center - Singapore (Singapore)

**Abstract**

**Introduction:** Ensuring holistic management of surgical oncology patients with advanced malignancies such as peritoneal carcinomatosis requires consideration of palliative care. Despite the recognized importance of integrating palliative care into surgical training, gaps persist in education and practice. This systematic review evaluates the design, implementation, and efficacy of palliative education programs (PEPs) for surgeons, aiming to delineate a more effective curriculum for primary palliative care training among surgical oncologists.

**Search Strategy and Selection Criteria:** Following PRISMA guidelines, a comprehensive search across MEDLINE, EMBASE, and the Cochrane Library was conducted till February 2023, using combinations of both MeSH and non-MeSH keywords. We identified 10 studies, comprising 571 surgeons, that described the implementation, content coverage, and outcomes of PEPs for surgical residents, fellows, or surgeons. Quality assessment was performed using NIH tools, with findings synthesized narratively due to heterogeneity.

**Main findings:** The review identified significant variability in PEP designs, content, and outcome measures, with most programs showing improvements in palliative care knowledge and communication skills. However, the lack of standardized curriculum and methodological rigor across studies complicates the establishment of best practices. Despite this, there was a noted improvement in surgeons’ comfort with end-of-life discussions and palliative care principles, underscoring the potential impact of targeted education.

**Conclusion:** Our review reveals that the current design, implementation, and assessment of existing PEPs for surgeons vary widely between institutions and lack methodological rigor. The findings highlight an urgent need for a standardized, comprehensive PEP for surgical oncologists that incorporates multidisciplinary approaches. Such a program should not only aim to improve knowledge and communication skills but also foster a positive shift in attitudes towards palliative care

### PO 39 The Impact of Neighborhood-Level Disadvantage on Treatments and Outcomes in Newly Diagnosed Appendix Cancer #PO 39

#### Oral communication or poster

V. Kovalik^1^, A. Sardi^1^, M.C. King^1^, S. Iugai^1^, L.F. Falla-Zuniga^1^, C. Nieroda^1^, V. Gushchin^1^

^1^Mercy Medical Center - Baltimore (United States)

**Abstract**

**Background:** Peritoneal surface malignancy institutions (PSMI) admit appendix cancer (AC) patients for cytoreductive surgery and hyperthermic intraperitoneal chemotherapy (CRS/HIPEC) from various residential areas (RA). We assessed RA deprivation and CRS/HIPEC outcomes.

**Methods:** This retrospective cohort study, using a prospective PSMI database (1998-2023), included US-based AC patients with peritoneal metastases treated with initial CRS/HIPEC. We evaluated geospatial distribution and area deprivation index (ADI), categorizing patients by within-state ADI decile: high (H-ADI, 7-10), moderate (M-ADI, 4-6), and low (L-ADI, 1-3). Peritoneal cancer index (PCI), non-definitive treatments (NDT), incomplete cytoreductions (ICR), and complications graded via the Clavien-Dindo Classification were compared. Cox regression estimated hazards of RA deprivation for 10-year overall survival (OS) with 95% confidence interval (CI).

**Results:** Overall, 369 patients were included: 95 (25.7%) H-ADI, 123 (33.3%) M-ADI, and 151 (40.9%) L-ADI. Patients had comparable age (p=0.399), disease burden (PCI>20 range: 68.4%–74.6%, p=0.531), and prior NDT rates (range: 48.4%–49.7%, p=0.703). ICR rates were 17.9%, 11.4%, and 9.9% in H-ADI, M-ADI, and L-ADI, respectively (p=0.175). Patients from disadvantaged RA (H-ADI) had more grade-3/4 complications (24.2% H-ADI vs 21.1% M-ADI or 11.9% L-ADI, p=0.030) and 90-day readmissions (30.9% H-ADI vs 21.1% M-ADI or 17.4% L-ADI, p=0.048). The 90-day mortality was 3.2%, 2.5%, and 1.3% for H-ADI, M-ADI, and L-ADI, respectively (p=0.612). H-ADI and M-ADI hazard ratios for OS were 1.64 (95%CI: 0.98, 2.72) and 1.56 (95%CI: 0.97, 2.50), respectively.

**Conclusion:** AC patients from disadvantaged neighborhoods are more susceptible to major complications and readmissions after CRS/HIPEC despite comparable disease burden and NDT history at presentation.

Distribution of Residential Areas

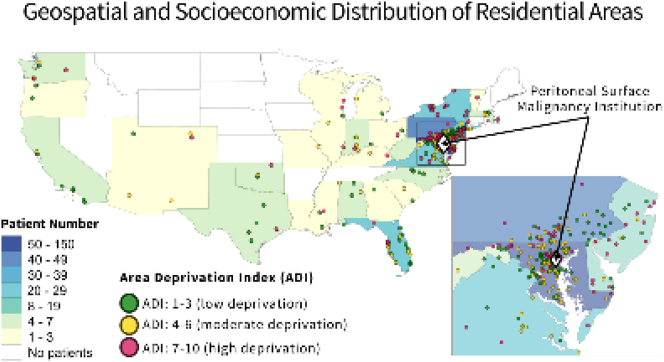



### PO 40 An Integrated Prehabilitation and Serious Illness Care Program in Patients with Peritoneal Surface Malignancy: A Prospective Pilot Study #PO 40

#### Oral communication or poster

A.T.I. Ng^1^, W.J. Fong^1^, P.L. Koh^2^, M. Babu Ramalingam^3^, M.Z. Cai^1^, C.J. Seo^1^, C.A.J. Ong^1^, S.C. Chia^1^, S.M.J. Wong^1^

^1^Department of Sarcoma, Peritoneal and Rare Tumours (SPRinT), Division of Surgery and Surgical Oncology, National Cancer Centre Singapore and Singapore General Hospital, Singapore - Singapore (Singapore),

^2^Department of Sarcoma, Peritoneal and Rare Tumours (SPRinT), Division of Surgery and Surgical Oncology, National Cancer Centre Singapore and Singapore General Hospital, Singapore - Singapore (Singapore) - Singapore (Singapore),

^3^Department of Rehabilitation Medicine, Singapore General Hospital, Singapore - Singapore (Singapore)

**Abstract**

Title

An Integrated Prehabilitation and Serious Illness Care Program in Patients with Peritoneal Surface Malignancy: A Prospective Pilot Study

**Background:** Peritoneal surface malignancies (PSM) are advanced malignancies associated with debilitating symptoms and constitute life limiting serious illnesses. The management of PSM is challenging and often requires complex surgeries. Multi-modal prehabilitation via physiotherapy and immunonutrition has been shown to prevent functional decline after surgery and improve peri-operative outcomes. Serious illness conversations (SIC) has also been shown to improve goal concordant care.

We hypothesize that an integrated prehabilitation and serious illness care program in PSM patients undergoing cytoreductive surgery (CRS) with or without hyperthermic intraperitoneal chemotherapy (HIPEC) is feasible and can improve patient-oriented outcomes.

**Method:** We conduct a prospective study including patients with PSM undergoing CRS with or without HIPEC at the National Cancer Centre Singapore between September 2023 and January 2024. The integrated program was considered feasible if there was more than 50% compliance to prehabilitation and SIC. Patient-oriented outcomes include 1) 5-level EuroQol-5 Dimension Instrument (EQ-5D-5L) questionnaire and 2) delivery of goal-concordant caredetermined by concordance in life priority survey administered. Both questionnaires were administered at baseline and at 1-month after surgery.

**Results:** Among 39 PSM patients, 74% were compliant to prehabilitation and 44% received SIC. 5.1%(n=2) received CRS and 61.5% (n=24) CRS-HIPEC for PSM. Mean EQ-5D-5L utility scores improved by 0.07 (0.87 baseline vs 0.95 post-surgery, 95% CI 0.01–0.1, p = 0.02).

Goal-concordant care was achieved in 74%. Prior to surgery, 90% of patients expressed their key priority was to achieve “cure of condition” while 79.5% prioritized “preservation of function”, followed by “prolonging life” (64%), relief of symptoms (38.5%) and establishing a diagnosis (2.6%).

**Conclusion:** A integrated prehabilitation and serious illness care program is feasible and can play a role in improving patient-oriented outcomes and the delivery of goal-concordant care.

### PO 41 The textbook outcome in ovarian cancer: an interesting tool for comparing results between Surgical Peritoneal Units #PO 41

#### Oral communication

S. Carbonell-Morote^1^, P. Cascales^2^, A. Arjona^3^, E. Gil-Gómez^2^, G. Gómez-Dueñas^4^, I. Caravaca^5^, A. González-Gil^2^, V. Aranaz^2^, J. Ramia^1^, F.J. Lacueva^5^

^1^Hospital universitario Dr. Balmis Alicante - Alicante (Spain),

^2^Hospital Virgen de la arrixaca - Murcia (Spain),

^3^Hospital Reina Sofía - Cordoba (Spain),

^4^Hospital reina sofia - Cordoba (Spain),

^5^Hospital General universitario Elche - Elche (Spain)

**Abstract**

**Introduction:** Patients who achieve the textbook outcome(TO) have an ideal, uneventful postoperative course. Obtaining TO has also been related to better survival in cancer patients. Information on TO in patients undergoing surgery for peritoneal carcinomatosis due to ovarian cancer is very scarce. The objective of this study is to investigate TO in patients with carcinomatosis of ovarian origin who underwent interval surgery with/without HIPEC(TOOC) and its impact on survival.

**Methods:** A multicenter retrospective observational study was conducted on a prospective basis between January 2010 and January 2015. The inclusion criteria were patients >18 years old, with ovarian cancer and peritoneal carcinomatosis, undergoing elective surgery(with/without HIPEC) after response to neoadjuvant therapy. The criteria used to establish TOOC were: i)no major Clavien-Dindo complications, ii)no mortality, iii)non-prolonged stay(less than p75=10 days), iv)complete cytoreduction(CC-0), and v)no readmission within 30 days.

**Results:** 365 patients were included; TOOC was achieved in 204(55.9%) patients. Complete CC-0 cytoreduction was obtained in 312(85.5%). Seven patients(1.9%) died. Seventy-one patients(19.5%) presented major complications(≥IIIa). The readmission rate was 9.3%, and 24.9% of patients had a prolonged stay. The parameter with the most significant negative impact on TOOC achievement was the length of hospital stay. Multivariate analysis confirmed PCI and post-surgical ileus as independent predictors of TOOC. Survival analysis showed that patients who achieved TOOC achieved better overall survival(41 versus 27 months)(p<0.0001).

**Conclusion:** TO is a simple and valuable management tool to evaluate and compare the results obtained in different centers after surgery for peritoneal carcinomatosis of locally advanced ovarian cancer. Achieving TO had survival benefits.

### PO 42 Measurement of survival according to textbook outcome and PCI in patients with interval surgery for carcinomatosis of ovarian cancer #PO 42

#### Poster

S. Carbonell-Morote^1^, P. Cascales^2^, A. Arjona^3^, G. Gómez-Dueñas^3^, A. González-Gil^2^, E. Gil-Gómez^2^, I. Caravaca^4^, V. Aranaz^4^, J. Ramia^1^, F.J. Lacueva^4^

^1^Hospital General Universitario Dr. Balmis Alicante - Alicante (Spain),

^2^Hospital Virgen Arrixaca - Murcia (Spain),

^3^Hospital Reina Sofía - Córdoba (Spain),

^4^Hospital General universitario Elche - Elche (Spain)

**Abstract**

**Introduction:** Patients who achieve the textbook outcome (TO) have an ideal, uneventful postoperative course. Obtaining TO has also been related to better survival in cancer patients. Information on TO in patients undergoing surgery for peritoneal carcinomatosis due to ovarian cancer is very scarce. The objective of this study is to investigate survival based on TO and PCI in patients with carcinomatosis of ovarian origin who underwent interval surgery with/without HIPEC.

**Methods:** A multicenter retrospective observational study was conducted on a prospective basis between January 2010 and January 2015. The inclusion criteria were patients >18 years old, with ovarian cancer and peritoneal carcinomatosis, undergoing elective surgery (with/without HIPEC) after response to neoadjuvant therapy. The criteria used to establish TOOC were: i) no major Clavien-Dindo complications, ii) no mortality, iii) non-prolonged stay (less than p75=10 days), iv) complete cytoreduction (CC-0), and v) no re-entry within 30 days.

**Results:** 365 patients were included; TOOC was achieved in 204(55.9%) patients. CompleteCC-0 cytoreduction was obtained in 312(85.5%). Seven patients(1.9%) died. Seventy-one patients(19.5%) presented major complications(≥IIIa). The readmission rate was 9.3%, and 24.9% of patients had a prolonged stay. The parameter with the most significant negative impact on TOOC achievement was the length of hospital stay. Survival analysis showed that patients who achieved TOOC achieved better overall survival (41 versus 27 months) (p<0.0001). When performing stratification according to PCI<10; 10-20 and >20, it was observed that patients who reached TO had better survival statistically significant, regardless of PCI.

**Conclusion:** TO is a simple and valuable management tool for evaluating and comparing the results obtained in different centers after surgery for peritoneal carcinomatosis of locally advanced ovarian cancer. Achieving TO has survival benefits in any PCI.

### PO 44 Does Incisional hernia reducible in patients undergoing Cytoreductive Surgery and HIPEC: An observational clinical study from a tertiary oncology center, India #PO 44

#### Oral communication or poster

M.D. Ray^1^, A. Kumar^1^

^1^All India Institute of Medical Sciences - New Delhi (India)

**Abstract**

**Background:** Incisional hernia (IH) is an unwanted and bothersome complications after Cytoreductive Surgery (CRS) and Hyperthermic Intra Peritoneal Chemotherapy (HIPEC). The frequency of occurrence of IH among patients with peritoneal surface malignancies treated with CRS and HIPEC remains unexpectedly high in various Studies. Our study aimed to analyze the incidence of IH in our cohort of patients and identify the factors associated with occurrence of IH and found the ways to reduce it.

**Methods:** After Ethical Clearance, we retrospectively analyzed the data from a prospectively maintained structured computerized comprehensive database from January 2013 to December 2023 for 360 patients who had undergone CRS and HIPEC. All patients were followed-up for a minimum period of two years with physical examination. Radiological imaging as per requirement basis and IH was documented. Identified the factors associated with IH.

**Results:** Within two years of CRS and HIPEC, 25 patients (6.9%) out of 360, developed IH. Indicated an annual incidence rate of 3.5%. The mean duration of hospitalisation was 8.4 + 4.13 days. 52 (14.4%) had early post-operative surgical complications like surgical site infection. The development of IH in our series was significantly associated with the occurrence of early post-operative surgical complications, (48% vs 12%, p = 0.001), mainly Clavien-Dindo II (16% vs 7%, p = 0.001), and III (44% vs 4%, p = 0.001), post chemotherapy status (72% vs 87%, p = 0.045) and bowel anastomosis (32% vs 11%, p = 0.002).

**Conclusion:** The low incidence of IH following CRS and HIPEC in our patient cohort, as compared to the existing literature, can be attributed to a combination of factors, including meticulous surgical techniques and application of abdominal binder post operatively, especially in obese patients. Identification of significant factors for developing IH and tackling them meticulously, reduce the incidence.

### PO 45 Cytoreductive Surgery Plus Hyperthermic Intraperitoneal Chemotherapy (CRS+HIPEC) Leads to Sustained Long-term Quality of Life in Patients with Peritoneal Surface Malignancies #PO 45

#### Oral communication or poster

M. Montealegre^1^, K. Gomez^1^, P. Borowsky^1^, A. Hernandez^1^, C. Cash^1^, S. Yadegarynia^2^, P. Joshi^1^, H. Bahna^2^, M. Moller^1^

^1^University of Miami - Miami (United States),

^2^University of Miami/ JFK Hospital - Miami (United States)

**Abstract**

**Introduction:** CRS+HIPEC is used to treat peritoneal surface malignancies. This surgery, however, is associated with extensive morbidity, potentially impacting patient satisfaction. This study assessed the long-term impact of CRS+HIPEC on patient-reported quality of life (QoL).

**Methods:** Patients undergoing CRS+HIPEC were prospectively enrolled from 2017-2024 and completed the 26 item World Health Organization (WHOQOL-BREF) QoL questionnaire in the pre- and/or post-operative periods. Questionnaires assessed functionality in physical (PF), psychological (PSY), social (SOC), and environmental (ENV) domains. Mean pre- and post-operative domain scores were compared using t-test analyses.

**Results:** 161 of 274 patients were alive at time of assessment, of which 69 (43%) completed QoL analyses. 41, 11, and 17 patients completed postoperative, preoperative, and both surveys, respectively. Mean patient age was 53 ± 12 years. Most patients were white, Hispanic, female, had primary appendiceal malignancy, and ECOG status of 0. Mean preoperative PCI score was 15 (range 0-39). 47 (68%) patients had R0 resection. Mean hospital length of stay was 11 days. Median follow up time was 23.3 months (range 27 days-145.5 months). Median time from surgery to postoperative QoL survey completion was 30.1 months (82 days-147.5 months). Overall, mean PF, PSY, and ENV scores were higher postoperatively, although this was only significant for PSY scores (p= 0.037). SOC scores remained stable pre- and post-operatively. For patients completing both pre- and post-operative surveys, pre- and post-operative PF, PSY, and SOC scores did not differ (p> 0.05); ENV scores were higher preoperatively (p= 0.009).

**Conclusion:** Even more than 30 months postoperatively, patients undergoing CRS+HIPEC can sustain stable long-term QoL. This study includes to our knowledge the longest follow up time for assessment of QoL after CRS+HIPEC. The results suggest patients should be counseled that QoL remains stable, or potentially improved, after CRS+HIPEC despite the known morbidity associated with this procedure.

### PO 46 Building a Peritoneal Surface Malignancy Unit: diagnosis, treatment and palliation of peritoneal carcinomatosis #PO 46

#### Oral communication or poster

S. Guerrero-Macías^1^, M.E. Manrique-Acevedo^1^, D. Viveros-Carreño^1^, C. Bonilla^1^, M. Pierre^1^, C. Cruz^1^

^1^Cancer Treatment and Research Center (CTIC) - Bogotá (Colombia)

**Abstract**

**Background:** Since 1995, Dr. Sugarbaker has proposed and described the treatment of carcinomatosis with cytoreductive surgery. However, over the years, various strategies have been developed to optimize the management of the patients with peritoneal malignancies throughout their disease process. International groups dedicated to the study and treatment of peritoneal surface malignancies (PSM) propose that the management of these patients should be guided and supported by a multidisciplinary team with training in oncology, for the benefit of patients with this diagnosis.

**Methods:** We describe the processes involved in establishing a multidisciplinary peritoneal malignancy team encompassing the diagnostic, therapeutic and palliative scenario of patients with peritoneal carcinomatosis. Our institution protocols are based on pre-habilitation, patient selection, perioperative care and research, aligning with recommendations set by the international organizations dedicated to the study and management of PSM.

**Results:** The PSM Unit of the Cancer Treatment and Research Center (CTIC) is located in Bogotá, Colombia. It has been operational for 10 months, the team is integrated of surgical, clinical, and therapeutic support professionals and includes 8 protocols for its different stages of care. Diagnostic procedures were initiated in July 2023, followed by therapeutic procedures in August 2023. To date, 52 diagnostic procedures (precision laparoscopies), and 28 therapeutic procedures (cytoreduction with and without HIPEC) have been performed. Additionally, the use of intraperitoneal therapies with palliative intent (NIPEC and PIPAC) is currently under development. There are three prospective studies in the recruitment phase.

**Conclusions:** The groups dedicated to managing PSM must adhere to professional, technical, and research standards to ensure the comprehensive care of patients with peritoneal carcinomatosis. Despite considerable challenges and barriers in our country, our team is convinced of the benefits of the program. We are determined to persevere in our goal of ensuring that more patients receive the appropriate treatment for this disease.

### PO 47 Opportunistic Cholecystectomy during cytoreductive surgery for secondary peritoneal malignancies #PO 47

#### Oral communication or poster

S. Guerrero-Macías^1^, D. Viveros-Carreño^1^, M.E. Manrique-Acevedo^1^, C. Cruz^1^

^1^Cancer Treatment and Research Center (CTIC) - Bogotá (Colombia)

**Abstract**

**Background:** Gallstone disease is one of the most common pathological entities in the daily practice and in emergency surgery. Consequently, cholecystectomy stands as one of the most commonly performed procedures in Colombia. However, a persistent debate surrounds its prophylactic role in elective surgery. This debate gains particular significance among patients undergoing cytoreductive surgery (CRS), as additional abdominal procedures in such cases may carry a heightened risk of morbidity due to intra-abdominal adhesions and the potential for visceral injuries. This report aims to assess postoperative morbidity associated with opportunistic cholecystectomy at the time of CRS.

**Methods:** Retrospective cohort study of patients who underwent CRS in the Peritoneal Surface Malignancies Unit of the Luis Carlos Sarmiento Angulo Cancer Treatment and Research Center (CTIC) in Bogotá, Colombia since its opening in August 2023. Postoperative outcomes among patients who underwent opportunistic cholecystectomy at the time of CRS with or without HIPEC are described.

**Results:** Over an 8 months period, 27 patients underwent to CRS with or without HIPEC. 66.6% for ovarian cancer, 22.2% gastric cancer, 6 % colon and appendiceal cancer and 5.2% other neoplasms. Cholecystectomy was performed in 24 patients (88.8%) and it was an opportunistic procedure in the 95.8%. There were no minor or major complications related to de procedure. There was no effect on length of hospital stay, postoperative morbidity or hospital readmissions in these patients.

**Conclusion:** Opportunistic cholecystectomy is a safe procedure in patients undergoing CRS with or without HIPEC and is not associated with increased morbidity or hospital length of stay according to historical data. While the benefits of this procedure is not yet elucidated, it may be considered to avoid potential future complication during surgeries for biliary gallstone disease.

### PO 48 Complications and mortality rate of cytoreductive surgery with hyperthermic intraperitoneal chemotherapy: Italian Peritoneal Surface Malignancies Oncoteam results analysis #PO 48

#### Oral communication

R. Lo Dico^1^, F. Carboni^1^, M. Valle^1^, M. Vaira^2^, M. Robella^2^, P. Sammartino^3^, D. Biacchi^3^, M. Deraco^4^, L. Martin-Romano^4^, D. Baratti^4^

^1^IFO National Cancer Institut Regina Elena - Roma (Italy),

^2^Candiolo Cancer Institute, FPO – IRCCS, Candiolo, Torino, Italy - Torino (Italy),

^3^Cytoreductive Surgery and HIPEC Unit, Department of Surgery “Pietro Valdoni”, Policlinico Umberto I, Sapienza University of Rome - Roma (Italy),

^4^Peritoneal Surface Malignancy Unit, Department of Surgery, Fondazione IRCCS Istituto Nazionale Tu-mori, - Milano (Italy)

**Abstract**

**Background:** Cytoreductive surgery with hyperthermic intraperitoneal chemotherapy may significantly improve survival for selected patients with peritoneal surface malignancies, but it has always been criticized due to the high incidence of postoperative morbidity and mortality.

**Methods:** Data were collected from 9 Italian centers with peritoneal surface malignancies expertise within a collaborative group of the Italian Society of Surgical Oncology. Complications and mortality rate were recorded and multivariate Cox analysis was used to identify risk factors.

**Results:** The study included 2576 patients. The procedure was mostly performed for ovarian (27.4%) and colon cancer (22.4%). Median peritoneal cancer index was 13. Overall postoperative morbidity and mortality rates were 34% and 1.6%. A total of 232 (9%) patients required a surgical reoperation. Multivariate regression logistic analysis indentified the type of perfusion (p=<0.0001), body mass index (p=<0.0001), number of resections (p=<0.0001) and colorectal resections (p=<0.0001) as the strongest predictors of complications; whereas the number of resections (p=<0.0001) and age (p=0.01) were the strongest predictors of mortality.

**Conclusions:** Cytoreductive surgery with hyperthermic intraperitoneal chemotherapy is a valuable option of treatment for selected patients with peritoneal carcinomatosis providing low postoperative morbidity and mortality rates, if performed un high-volume specialized centers.

### PO 49 What is the proposed strategy offer by the different RENAPE unit after complete LAMN (Low grade mucinous appendicular tumors) resection in a primary care center: need for a new consensus ? #PO 49

#### Oral communication

M. Pocard^1^, A. Taibi^2^, O. Sgarbura^3^, D. Goere^1^, O. Glehen^4^, F. Dumont^5^, C. Brigand^6^, K. Amroun^7^, K. Abboud^8^, N. Renape^9^

^1^université Paris Cité - paris (France),

^2^Université de Limoges - Limoges (France),

^3^Unicancer - Montpellier (France),

^4^université de Lyon - Lyon (France),

^5^Unicancer - Toulouse (France),

^6^université de strasbourg - Strasbourg (France),

^7^université de Reims - Reims (France),

^8^université de Saint Etienne - Saint Etienne (France),

^9^INCA - INCA Paris (France)

**Abstract**

**Background:** Incidental resection is the most frequent situation for discovering an appendiceal tumor. Pathologists have made progress and diagnosis of LAMN is increasingly proposed, even in primary care hospitals. In France, in such situations the case is referred to a reference center within the French network – RENAPE. The Guidelines lack precision in certain situations and some teams adopt a noncompliance attitude, including a therapeutic de-escalation based on active monitoring using MRI. We planned a prospective study to analyze the different strategies proposed by the different RENAPE centers.

**Method:** Questionnaire based on simple clinical situations after incidental complete LAMN resection was addressed. 90% of centers responded.

**Results:** All centers required: RENARAD – pathological expert – new analysis – and a post appendectomy MRI. Majority (80%) analyze CEA, CA19-9 and CA 125, others only analyze CEA. In case of resected R0 LAM, with no abnormality during surgery and pathology (no mucus outside), biology and MRI, 50% of the center do not perform any follow-up, 50% proposed a follow-up until for one during 10 years, based on MRI. In case of limited perforation, observed by mucus outside during surgery or on pathological examination, but with normal CT and MRI: majority (80%) proposed a follow-up based on MRI at 6 month, and after every year; 10% proposed a laparoscopy at 3 months, and 10% a laparoscopic HIPEC procedure. In case of perforated LAMN, mucin completely aspirated during surgery and suspected implant of MRI: 60% proposed a cytoreductive surgery and HIPEC (for some robotic or laparoscopy) from 3 to 6 months, 30% consider a new laparoscopy to decide the strategy and 10% proposed an active follow-up.

**Conclusions:** Various strategies have been proposed for perforated LAMN highlighting the necessity to expand the data base analysis to propose new guidelines.

### PO 50 Pilot study on electronic patient reported experience and positive psychiatry outcome measures during PIPAC directed therapy #PO 50

#### Oral communication or poster

S. Hald Nielsen^1^, A.M. Ølholm^1^, C. Fristrup^1^, M.I.C.H.A. Bau Mortensen^1^

^1^Odense PIPAC Center - Odense (Denmark)

**Abstract**

**Background:** Pressurized Intraperitoneal Aerosol Chemotherapy (PIPAC) provides local treatment effect in patients with peritoneal metastasis (PM) without compromising their quality of life. Data on patients reported experience measures (PREM) and positive psychiatry outcomes (PPO) are limited.

**Methods:** Prospective electronic evaluation of PREM and PPO in patients scheduled for PIPAC. PREM focused on patients/relatives’ experience (21 questions, Likert scale) during first outpatient contact and subsequent PIPAC treatment. PPO was measured before first PIPAC (baseline) and after each PIPAC procedure using the WHO-5 questionnaire (0-100, 100 = best score). The mean score in the general population is 68, and PPO scores ≤50 triggered two additional questions (Major Depression Inventory, MDI-2) and clinicians were alerted.

**Results:** Twenty-six consecutive patients (14M/12F) were evaluated with PREM during first outpatient visit. Eighteen patients went on to have ≥ 1 PIPAC and were evaluated by additional PREM and PPO. All patients responded to both first and second PREM as well as all the PPO questionnaires. Two different PREM questions were unanswered in 2 patients. At least 80% responded very positive (highest or 2-highest rating) to 19/21 PREM questions. The two questions with lower scores concerned contact and treatment information after discharge. Mean PPO score was 80 (range 52-96) at baseline, and 73 (32-100), 67 (8-96) and 72 (56-88) after 1, 2 and 3 PIPAC, respectively. Low scores alerted the clinicians in four cases (4/18, 22%) leading to contact to the patient.

**Conclusion:** An electronic evaluation of PREM and PPO during PIPAC treatment was feasible, and preliminary data showed a high patient and relative’s satisfaction, and that PPO scores remained high during PIPAC treatment.

### PO 51 Systematic review of endpoints in clinical trials evaluating cytoreductive surgery and hyperthermic intraperitoneal chemotherapy #PO 51

#### Oral communication

A. Almog^1^, O. Sgrabura^2^, D. Delia^3^, M. Mohammad^4^, M. Martin^5^

^1^Chaim Sheba Medical Center - Ramat-Gan (Israel),

^2^Institut Régional du Cancer Montpellier - Montpellier (France),

^3^Hospital Universitario Principe de Asturias - Madrid (Spain),

^4^King Faisal Specialist Hospital and Research Center, Riyadh, Saudi Arabia - Riyadh (Saudi Arabia),

^5^University Hospital of Lausanne - Lausanne (Switzerland)

**Abstract**

**Background:** Clinical endpoints need to be objective, measurable and pertinent to allow for scientific evaluation of new treatments and for optimal clinical decision-making. This is of particular importance for major surgeries for metastatic disease.

**Methods:** This is a systemic review of clinical endpoints of phase II and III clinical trials on cytoreductive surgery (CRS) and hyperthermic intraperitoneal chemotherapy (HIPEC). Major scientific databases were queried by use of pre-defined MESH terms for prospective clinical studies published and registered in English language from 2000-2024. Descriptive statistics focused on primary and secondary endpoints.

**Results:** Systematic search revealed 3708 hits, and 175 papers were reviewed in detail to identify a total of 37 eligible clinical studies (n=36 curative intent, total n= 3952 patients), 12 and 25 were phase II and III studies, respectively. Fourteen were multi-center and 23 were single center studies. Overall survival was primary endpoint in 11 (29.7%) publications, secondary in 12 (32.4%) and neither in 14 studies. QoL or PROMs/PREMs were primary endpoint in 2 of the studies. The most frequently chosen primary and secondary endpoints were neither of the above: Disease free survival (DFS) 3 (8%) and 6 (16.2%), progression free survival (PFS) 7 (19%) and 4 (10.8%), relapse free survival 5 (13.5%) and 0, other (i.e. complications, toxicity, cost, etc.) 16 (43.2%) and 24 (64.8%) respectively.

**Conclusions:** Surrogate endpoints and early outcome measures were the most frequent endpoints in clinical trials on CRS/HIPEC. Patient and public involvement is needed to define the optimal endpoints for future studies in surgical oncology.

### PO 52 Peritoneal Mesothelioma Patient Experience with long term survival (10+ years) after CRS, HIPEC and adjuvant Chemotherapy #PO 52

#### Oral communication

S. Babin^1^, L. Fourcaud^2^

^1^author/patient - Bordeaux (France),

^2^physical therapist/co author - Bordeaux (France)

**Abstract**

Female patient diagnosed in 2013 at the age of 45 with epithelioid peritoneal mesothelioma. Treated with CRS, HIPEC and adjuvant chemotherapy (1 cycle cisplatin/ Pemetrexed, 3 cycles carboplatin/ Pemetrexed) in a specialized hospital in France.

In the months and years following treatment, the patient suffered 7 intestinal obstructions requiring hospitalization of an average of 8 days. Treatment was medical and successful on each occasion: nasogastric tube, IV steroids, IV anti spasmodic, IV morphine and nothing by month until return of flatulence and bowl movements. Reintroduction of liquids then solids was gradual over several days.

Patient was obligated to learn, question and experiment with different diets, lifestyle habits, medical and paramedical treatments to manage and avoid further intestinal blockages and digestive pain.

As a result of years of experimentation,the patient has been occulsion free since 2018 and maintains a decent quality of life as long as a strict management of lifestyle practices are respected: low fiber diet, strict rest after meals, exercise avoiding direct abdominal contractions, physical therapy on the abdomen (Busquet Method and facia therapy), stool softeners, hot water bottle application to abdomen, as well as stress management.

In conclusion, this patient has experience and knowledge to share with medical professionals which could be beneficial to the overall health and wellbeing of patients faced with peritoneal disease and treated with CRS and HIPEC. This patient’s experience could potentially help other patients to avoid serious, life threatening post-treatment complications with easy to implement lifestyle changes.

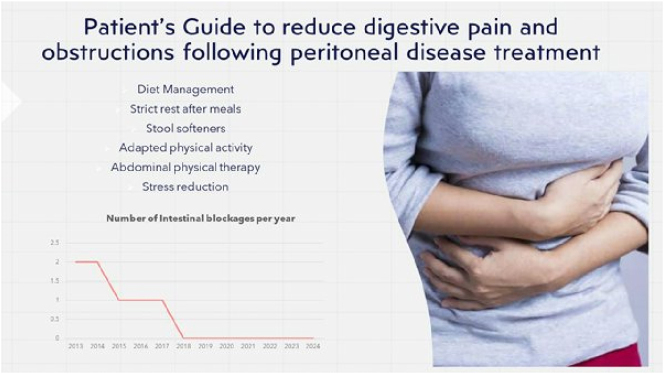



### PO 54 DEFYING LIMITS: A Case Report on the Experience of a Low Resource Hospital in the Philippines on its First Cytoreductive Surgery and Heated Intraperitoneal Chemotherapy #PO 54

#### Oral communication

L.C. De Leon^1^, J.A. Banzuela^1^, R. Sarmiento^1^, O. Bisquera Jr.^1^

^1^RIZAL MEDICAL CENTER - PASIG CITY (Philippines)

**Abstract**

**Background:** Pseudomyxoma peritonei (PMP) is a rare condition characterized by mucinous tumor cells within the peritoneal cavity, typically originating from the appendix. Treatment involves cytoreductive surgery (CRS) and hyperthermic intraperitoneal chemotherapy (HIPEC), which improve survival but are complex and costly. Few institutions in the Philippines have the expertise and resources to manage PMP. We present the first experience of a low-resource hospital in PMP management.

**Method:** A 64-year-old female with PMP from Low-grade Appendiceal Mucinous Neoplasm (LAMN) underwent extensive surgery and adjuvant chemotherapy but developed hepatic and splenic lesions. She was prepared for CRS and HIPEC with a Mitomycin-based regimen. Patient agreed to shoulder the expenses for the HIPEC machine and medication. Due to limited resources, extensive planning and permits were necessary, and a specialist surgeon was invited to assist. Intraoperatively, patient was closely monitored by a multidisciplinary team. Renal artery monitoring was also done. There was temporary cessation of HIPEC due to tubing leak, but it was completed after resolving the issue.

**Results:** Total operative time was 12 hours. The patient had intraoperative hypotension requiring low-dose inotropic support and received two units of blood. Postoperatively, she was transferred to the Surgical Intensive Care Unit (SICU), extubated the next day, and had an uneventful recovery. She progressed from general liquids to a full diet by postoperative day 5. Early rehabilitation was facilitated, and she received two additional units of blood. A superficial surgical site infection was managed, and all drains except one were removed before discharge. The patient continues to recover at home and will be closely monitored for complications, recurrence, or disease progression.

**Conclusions:** This case highlights the successful management of PMP in a low-resource Philippine hospital through CRS and HIPEC. Despite significant challenges, the patient’s recovery underscores the feasibility and potential for such complex treatments in similar settings.

## Perioperative care

### PO 190 Prehabilitation in Peritoneal Surface Malignancy Patients undergoing Cytoreductive Surgery and Hyperthermic Intra-peritoneal Chemotherapy: A Qualitative Research Study #PO 190

#### Oral communication or poster

N.H. Soh^1^, X. Zhong^1^, W.J. Fong^1^, P.L. Koh^1^, M. Cai^1^, C.J. Seo^1^, J.C.A. Ong^1^, C.S. Chia^1^, J.S.M. Wong^1^

^1^Department of Sarcoma, Peritoneal and Rare Tumours (SPRinT), Division of Surgery and Surgical Oncology, National Cancer Centre Singapore and Singapore General Hospital, Singapore - Singapore (Singapore)

**Abstract**

**Background:** Prehabilitation comprising of exercise therapy, psychological support and nutritional supplementation has been shown to improve peri-operative outcomes among patients undergoing major abdominal surgery. We piloted a structured prehabilitation program for patients with peritoneal surface malignancies (PSM) planned for cytoreductive surgery and hyperthermic intra-peritoneal chemotherapy (CRS-HIPEC). In this study, we aim to explore the overall experience during prehabilitation among these patients.

**Methods:** The sample was drawn from a prior prospective cohort study exploring the efficacy of prehabilitation among PSM patients undergoing CRS-HIPEC. English-speaking patients who were fully compliant to the prehabilitation program were recruited. Interviews were recorded, transcribed, and analyzed using Malterud’s principles of systematic text condensation. We present interim findings of our analysis.

**Results:** Among five recruited patients, there were, 3 colorectal, 1 appendiceal and 1 urachal PSM. All patients expressed that confidence in their primary peritoneal surgeon who had reinforced the importance of prehabilitation before CRS-HIPEC was a strong motivator for participation and compliance. Most also experienced a sense of “feeling cared for” through the structured program and remained motivated throughout it. Three patients expressed reluctance in engaging in the psychological aspects of the program and preferred to draw emotional support directly from their caregivers.

**Conclusion:** Reinforcement by the primary peritoneal surgeon is key to ensuring participation and compliance to prehabilitation programs. Further analysis to data saturation will elucidate richer themes to identify barriers and facilitators to prehabilitation among PSM patients.

### PO 191 Feasibility and Efficacy of An Integrated Tertiary-Primary Care Team in Peritoneal Surface Malignancy Patients Following Major Abdominal Surgery #PO 191

#### Oral communication or poster

S. Bek^1^, W.J. Fong^1^, T. Javed^2^, P.L. Koh^1^, M. Tan^3^, C. Mingzhe^1^, S. Chin Jin^1^, J. Ong^1^, C. Chia^1^, J. Wong^1^

^1^Department of Sarcoma, Peritoneal and Rare Tumours (SPRinT), Division of Surgery and Surgical Oncology, National Cancer Centre Singapore and Singapore General Hospital - Singapore (Singapore),

^2^Clinical Trials and Research Centre, Division of Surgery and Surgical Oncology, Singapore General Hospital - Singapore (Singapore),

^3^Department of Family Medicine and Continuing Care, Singapore General Hospital - Singapore (Singapore)

**Abstract**

**INTRODUCTION:** Major abdominal surgery for peritoneal surface malignancies (PSM) can be associated with post-operative sequelae that can prolong length of stay (LOS) and increase rates of emergency readmissions. Community services including home care delivered by primary care physicians and community nurses can ease transition back to the community following surgery. We trained a community team in post-operative care and piloted an integrated tertiary-primary care service. We hypothesize that this integrated care service is feasible and efficacious in PSM patients.

**METHODS:** We conducted a prospective cohort study including PSM patients following cytoreductive surgery (CRS) with or without hyperthermic intraperitoneal chemotherapy (HIPEC) or palliative surgery. Suitable patients were identified by the integrated tertiary-primary care team and enrolled into the intervention arm. These included those requiring post-operative wound, stoma, drain care, or parenteral nutritional support. Feasibility was measured by the number of dropouts and rates of emergency readmissions in the intervention arm. Efficacy was measured using LOS and absolute reductions in LOS i.e, planned discharge minus actual discharge dates. We built regression models to evaluate the impact of the integrated care service on LOS outcomes.

**RESULTS:** There were 160 PSM patients over a 1.5-year duration. We enrolled 17 patients into the integrated care service for stoma (N=7), wound (N=7), drain (N=5), and nutritional support (N=1). Dropout rate was 17%. Mean LOS was 17.8 vs. 27.7 days in the intervention vs. control arm (mean difference -9.9, 95%CI -19.7– 0.01; p=0.05). Mean absolute reductions in LOS in the intervention arm was 11.4 days (range 5–21). Readmission rates were 0.6 vs. 0.4 in intervention vs. control arm (mean difference 0.2, 95%CI -0.1–0.6, p=0.5).

**CONCLUSION:** An integrated tertiary-primary care service both feasible and associated with improvements in LOS amongst PSM patients.

### PO 192 The Impact of Body Mass Index on Postoperative Outcomes in Older Adults after Major Surgery #PO 192

#### Poster

J.K.T. Tan^1^, W.J. Fong^1^, H. Abdul Kadir^2^, C.J. Seo^1^, M. Cai^1^, C.A.J. Ong^3^, S.C. Chia^1^, H.R. Abdullah^4^, S.M.J. Wong^1^

^1^Department of Sarcoma, Peritoneal and Rare Tumours (SPRinT), Division of Surgery and Surgical Oncology, National Cancer Centre Singapore and Singapore General Hospital, Singapore - Singapore (Singapore),

^2^Health Service Research Unit, Singapore General Hospital, Singhealth Tower, 10 Hospital Boulevard, Singapore 168582, Singapore - Singapore (Singapore),

^3^Department of Sarcoma, Peritoneal and Rare Tumours (SPRinT), Division of Surgery and Surgical Oncology, National Cancer Centre Singapore and Singapore General Hospital, Singapore; 3Laboratory of Applied Human Genetics, Division of Medical Sciences, National Cancer Centre Singapore, Singapore - Singapore (Singapore),

^4^Department of Anaesthesiology, Singapore General Hospital, Outram Road, Singapore 169608, Singapore - Singapore (Singapore)

**Abstract**

**BACKGROUND.** The ageing population has become a pertinent phenomenon in countries across the globe. This has a detrimental impact on postoperative morbidity, straining healthcare resources. Some studies suggest body mass index (BMI) can function as a screen for preoperative fitness and malnutrition in a range of surgical procedures. However, literature on the specific impact of BMI on early postoperative outcomes on the elderly surgical population is sparse. We hypothesize that BMI is associated with poor post-operative outcomes among older adults undergoing major surgical procedures.

**METHODS.** We utilized an institutional database, PASAR (Perioperative Anesthesia Subject Area Registry) to identify older adults (aged ≥ 65) who underwent major surgical procedures (HCUP definition) at a single institution between January 2020 December 2021. Low BMI was defined as < 18.5kg/m2. Multivariable regression models were used to determine if BMI was independently associated with 30-day postoperative mortality and overall inpatient length of stay (LOS), and LOS in high dependency (HD) and intensive care (ICU).

**RESULTS.** Of 6549 older adults studied, 3.7% (N=244) had low BMI. Overall, mean BMI was 25.8kg/m2, 30-day mortality was 0.1 %, mean hospital LOS was 9.0 days, mean HD stay 21.8 hours and mean ICU stay 8.6 hours. Comparing older adults with low vs. normal BMI, low BMI was associated with longer overall LOS (mean LOS 14.79 days vs 9.63 days, coefficient 3.72 SE 0.84, p<0.001) and HD stay (mean LOS 42.38 vs 24.88 hours, coefficient 11.99 SE 5.15, p=0.02). There was no difference in ICU LOS (10.12 vs 9.67 hours, coefficient 0.83 SE 5.62, p=0.9) and 30-day mortality (OR 1.24, 95% CI 0.98-1.79).

**CONCLUSION.** Low BMI is associated with prolonged LOS and HD stay after surgery in the geriatric surgical population. These findings can be incorporated into risk stratification models for early identification of at-risk patients to optimize outcomes.

### PO 195 Phase angle in patients with peritoneal carcinomatosis undergoing cytoreductive surgery after a prehabilitation program. #PO 195

#### Poster

J.J. Macia^1^, S. Lario-Pérez^2^, C. Lillo-García^1^, I. Caravaca^1^, F. López-Rodríguez^3^, V. Aranaz^4^, L. Sánchez-Guillén^3^, A. Moya-Martínez^5^, M.I. Tomás Rodríguez^6^, F.J. Lacueva^4^

^1^Oncological Abdominal and Pelvic Surgery Unit. General Surgery Department. University General Hospital of Elche - Elche (Spain),

^2^General Hospital Obispo Polanco - Teruel (Spain),

^3^Colorectal Unit. General Surgery Department. University General Hospital of Elche. Center for Translational Research in Physiotherapy. Pathology and Surgery Department. Miguel Hernandez University of Elche - Elche (Spain),

^4^Oncological Abdominal and Pelvic Surgery Unit. General Surgery Department. University General Hospital of Elche. Center for Translational Research in Physiotherapy. Pathology and Surgery Department. Miguel Hernandez University of Elche - Elche (Spain),

^5^Statistical Department. FISABIO. University General Hospital of Elche. Spain - Elche (Spain),

^6^Center for Translational Research in Physiotherapy. Pathology and Surgery Department. Miguel Hernandez University of Elche - Elche (Spain)

**Abstract**

**Background:** Phase angle (PA) has been used to assess nutritional status, body composition, complications and survival in patients with cancer. Lower PA value negatively correlates with malnutrition and systemic inflammation. Patients with peritoneal carcinomatosis often have a poor nutritional status and surgery is associated with severe morbidity. Multimodal prehabilitation may improve physical status but the benefit for these patients remains unknown. We assessed the PA after a home-based prehabilitation in patients with peritoneal carcinomatosis undergoing cytoreductive surgery (CRS) and HIPEC.

**Methods:** A prospective study of patients with peritoneal carcinomatosis following a home-based prehabilitation program was carried out. PA was estimated by bioelectrical impedance analysis (BIA) before prehabilitation and one-day before surgery (1-DBS). C-reactive protein was also assessed previous CRS. Postoperative morbidity was registered according to the Clavien-Dindo classification.

**Results:** Fifty-nine patients were included. Women were more prevalent (81.4%) and peritoneal metastasis from ovarian origin accounted for 52.5%. Sarcopenia was found in 29 (49.2%) patients and CC0 or CC1 was achieved in 51 (86.5%) patients. HIPEC was administered to 35 (59.3%) patients. Clavien II to V occurred in 28 (47.5%) patients. The median PA at the 1-DBS was 5.3º (4.8-5.8), higher in comparison with the PA at the baseline measurement 5º (4.6-5.6) (p=0.003). A subsequent analysis by sex confirmed the significant increase both in women 5.1º (4.8-5.7), p=0.01; as well as in men 5.8º (5.1-6.5); p=0.05. Clavien-Dindo II to V postoperative complications were more frequent in patients with a lower PA (p= 0.038). No significant correlation was found between PA and C-reactive protein.

**Conclusions:** PA increased significantly after the home-based prehabilitation, and a higher PA after prehabilitation was related with a fewer Clavien-Dindo II to V postoperative complications. No correlation was found between preoperative PA and C-reactive protein levels.

### PO 196 The outcome of ERAS Pathways Implementation with Cytoreductive Surgery (CRS) and Hyperthermic Intraperitoneal Chemotherapy (HIPEC) in patients with Peritoneal Metastasis. Multi Centres Experience #PO 196

#### Oral communication or poster

W. Abdel Gawad^1^, B.A. Al Dokany^1^, A.S. Zeweta^2^, A. Al Mutawa^3^, M. Samy^4^

^1^National Cancer Institute (NCI)-Cairo University-Egypt. KAH-Ministry of National Guard Medical Affairs Al Ahsa-Eastern Province - Cairo (Egypt),

^2^Ismalia Cancer Centre - Ismailia (Egypt),

^3^King Abdul Aziz Hospital-Ministry of National Guard Health Affairs (MNGHA) - Al Ahsa (Saudi Arabia),

^4^National Cancer Institute - Cairo (Egypt)

**Abstract**

**Introduction:** Implementation of ERAS has been positively reflected on remarkable decrease of surgical morbidity, length of hospital stays and financial expenses.

**Objectives:** The aim of this study was to evaluate the outcome of ERAS pathway with CRS and HIPEC regarding postoperative morbidity , mortality & hospital stay.

**Methods:** A total of 788 patients were enrolled at three Cancer centres, ERAS pathway was implemented from 2016-2023 (n =638), compared retrospectively to 150 patients in the pre-ERAS phase from 2011-2016. Age ranged (28-68) years - mean of 55.6 y ,M/F ratio was 1:4.5.Disease sites was; Ovarian(52%) , colorectal (23%), Appendix (14%) & PMP(6%) &Gastric (5%) . PCI range was (5-26) -mean (14.7). Completeness of Cytoreduction, CCR 0 (200) , CCR 1 (121) & CCR 2 –(84) patients respectively.

**Results:** In the Pre and post ERAS Implementation, IV fluids: mean 30.5 L, compared to 17.1 L (P<o.oo2). LOS was 10.2 days compared to 8.3 days, SSI & sepsis of 17 % & 12 % respectively (P < 0.02). The Surgical morbidity according to Clavin-Dando scoring system; grade I-III were evident in 55.3 % & in 40 %, in class IV 30.2% & 10 % respectively. The mortality rate was 3.5 % & 2.2 % respectively.

**Conclusion:** ERAS for CRS and HIPEC has showed a remarkable significant outcome with respect to relative risk reduction of 30% for III/IV complications, decreased LOS by 25%, decrease in total IVF and narcotic use with no increase in 30-day readmissions or mortality rate.

### PO 197 High dose iron infusion (ferric isomaltose/ derisomaltose) before CRS-HIPEC procedure: improvement in patients outcomes after surgery #PO 197

#### Poster

T. Reis^1^, I. Gomes^2^, M. Torres^1^, M. Reis^3^, M. Reis^3^

^1^OSWALDO CRUZ HOSPITAL - RECIFE (Brazil),

^2^UFPE-CLINICS HOSPITAL - RECIFE (Brazil),

^3^Health College of Pernambuco - RECIFE (Brazil)

**Abstract**

**Background:** Anemia implicate a worse prognosis in patients submitted to major surgeries. Surgery Guidelines suggest to treat iron disorders before surgery, to improve clinical outcome

**Methods:** Analysis of 25 consecultive surgical patients submitted to CRS-HIPEC, from Jan2021 to April 2024, after a single high dose iron infusion with 1000mg up to 2000mg (20 mg/kg) of ferric isomaltose/derisomaltose before surgery (Group A). These patients were compared with another consecultive 25 patients who did not receive iron infusion (Group B). Data was represented as median (IQR) and the Wilcoxon test was performed to compare primary outcomes: blood transfusion rates, time of hospital stay and hemoglobin levels after 30 days. Significance level adopted was 5% (p < 0.05). Statistical analysis was performed in R software package

**Results:** All primary outcomes (time of hospital stay, blood transfusion rates and hemoglobin levels after 30 days) were statistically significant and different between the groups that received a single high dose iron infusion (Group A) and those that did not receive (Group B).

**Conclusion:** A single dose of iron infusion before CRS-HIPEC was statistically significant in clinical outcomes, reducing hospital stay and blood transfusion rates, also rising hemoglobin levels after 30 days.

### PO 198 Evaluating the Impact of Indocyanine Green on assessing bowel perfusion in Cytoreductive Surgery with Hyperthermic Intraperitoneal Chemotherapy #PO 198

#### Oral communication or poster

C. Rohit Kumar^1^, S.P. Somashekhar^1^, K.R. Ashwin^1^, A.M. Fernandes^1^, K. Agarwal^1^, D. Patil^1^, E. Shanbagh^1^, V. Ahuja^1^

^1^Aster International Institute Of Oncology - Bengaluru (India)

**Abstract**

**Background:** Anastomotic leak after cytoreductive surgery (CRS) with hyperthermic intraperitoneal chemotherapy (HIPEC) is a significant concern. Studies have shown that ICG-based detection of bowel vascularity is feasible and has reduced anastomotic leak rates in minimally invasive surgery.

**Methods:** Patients who underwent CRS+HIPEC and had at least one bowel anastomosis were included. ICG was given intravenously to assess the bowel perfusion. The line of anastomosis was marked by surgeon initially before giving ICG. The perfusion transition area was named Line A if it was proximal,line B if it co incided , Line C if its distal to surgeon marked line. All variables pre, intra and post op were recorded in HIPEC registry. The leak rates for this cohort was compared with historic cohort of the institution.

**Results:** From 2021 to 2023, 64 of 104 patients underwent CRS+HIPEC and had at least one bowel anastomosis. A total of 94 anastomoses were performed, with a mean of 1.2 anastomoses per patient (range 1-2). Median age was 55 years, 35.5% underwent upfront CRS and the most common histology was serous epithelial ovarian cancer (62.3%). The median Peritoneal Cancer Index was 16±2. All patients ICG was clearly able to show line of vascularity for anastomosis. In 5 (7.8%) the line was proximal and in 13 (20.3%) it was distal. Only 1 patient developed an anastomotic leak. The incidence of anastomotic leaks and bowel complications was lower in the ICG cohort compared to the historic cohort (4.8% vs. 1.7%; p > 0.05).

**Conclusion:** ICG fluorescence is a simple, safe & useful technique with minimal added intraoperative time for accurate determination of the resection margin of the viable bowel and helps to reduce anastomotic leakage with subsequent improvement of outcome. ICG is easy to use logistically and provides important vascular information at low surgical expense.

### PO 199 The Impact of Multidisciplinary Team Discussion on Surgical Decision Making in Patients with Peritoneal Surface Malignancies: Model for Multi-Institutional International Cooperation #PO 199

#### Oral communication or poster

W. Abdel Gawad^1^, B. Al Dokanny^1^, A.S. Zeweta^2^, M. Samy^3^, A. Al Ghamedi^4^, F. Aldayel^4^, E. Al Mansor^4^, A.R. Al Mutawa^4^

^1^National Cancer Institute-Cairo University/-KAH -Ministry of National Guard-KSA - Cairo (Egypt),

^2^Ismalia Cancer Centre - Ismailia (Egypt),

^3^National Cancer Institute - Cairo (Egypt),

^4^KAH-Ministry of National Guard - Al Ahsa (Saudi Arabia)

**Abstract**

**Introduction:** The implementation of multi-disciplinary approach in cancer care has shown a decrease in variability between physicians’ recommendations, errors and provides a clear treatment plan according to guidelines.

**Aim:** The purpose of the study is to evaluate the impact of MDT discussion on patient selection for CRS and HIPEC in patients with peritoneal surface malignancy.

**Methods:** The study was conducted from 2019 - 2023. PSM patients (780) were prospectively enrolled in a weekly MDT conference. Disease sites were: gynaecological & colorectal malignancies diagnosed at the NCI, Cairo & Ismalia Cancer Centre (Egypt) and KAH-Saudi Arabia. Presentation through designed template with adequate corium of the members’ attendance & a final recommendations were also reported.

**Results:** Most referrals for MDT conference were from surgical oncology (342 patients, 76%), radiation oncology (54 cases, 12%), medical oncology (29 cases, 6.4%) and radiology (25, 5.6%). None of these patients had prior CRS and HIPEC. A single specialty Clinic evaluated 195 Patients (25%) out of the 780 patients and decided for CRS and HIPEC ( 118 gynecological & 77 CRC) patients. MDT conference reviewed the 195 patients, only 20 patients (9.7%) were deemed appropriate candidate for CRS & HIPEC with 70% financial saving.

**Conclusion:** The diverse clinical expertise of the MDT members is essential and critical in identifying patients with PSM who would benefit the most from CRS & HIPEC. This is important for achieving positive impact on patients’ prognosis and quality of life ,cancer service efficiency and cost effectiveness in countries with limited resources.

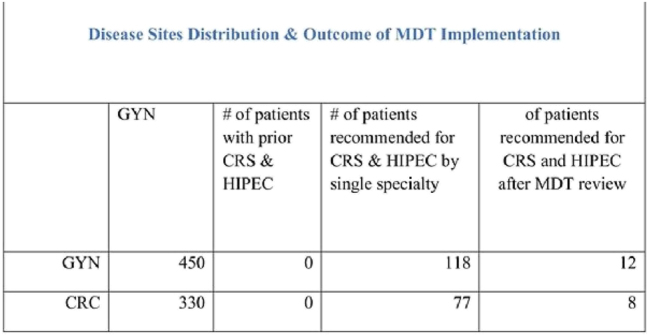



### PO 200 Assessment of Role of Enhanced Recovery After Surgery (ERAS) Protocols in Patients undergoing Cytoreductive Surgery with Hyperthermic Intraperitoneal Chemotherapy (HIPEC) - A Prospective Feasibility Study #PO 200

#### Oral communication or poster

P. Das^1^, A. Balasubramanian^1^, P. Subramani^1^

^1^JIPMER - Pondicherry (India)

**Abstract**

**Introduction:** Cytoreductive surgery (CRS) with hyperthermic intraperitoneal chemotherapy (HIPEC) attributes to higher postoperative morbidity. Incorporation of enhanced recovery after surgery (ERAS) protocols in perioperative management can be promising in reducing postoperative morbidity and accelerating recovery.

**Materials and methods:** Patients requiring cytoreductive surgery with HIPEC were prospectively assigned to undergo predefined prehabilitation and perioperative protocols. The objective was to assess the feasibility of protocol adherence and evaluate the effect of ERAS-protocol on perioperative recovery and early postoperative morbidity.

**Results:** Total 16 patients were included in the trial. Mean age was 48.5 + 10.82 years. Out of included cases recurrent ovarian carcinoma was most frequent (75%), followed by appendiceal mucinous neoplasm (12.5%) and primary peritoneal carcinomatosis (12.5%). Median Peritoneal Carcinomatosis Index (PCI) was 12. Complete cytoreduction was achieved in 75% cases. Median duration of epidural analgesia requirement was 48 hours. Median number of postoperative days for starting oral feed and removal of nasogastric tube was 2; for urinary catheter removal was 3 and abdominal drain removal was 7. Median length of stay in the intensive care unit was 4 days and Median length of postoperative hospital stay was 10 days. Four patients (25%) developed Calvien-Dindo Grade III-IV morbidity. Adherence to preoperative and intraoperative protocols was 100% and 93.8% respectively; 43.75% cases had adherence to postoperative protocols.

**Conclusion:** Adoption of ERAS protocol for patients undergoing CRS and HIPEC has potential for faster postoperative recovery with reduced morbidity. The implementation of postoperative protocols was associated with lower adherence than other perioperative approaches

### PO 201 Enhanced Recovery After Surgery (ERAS) Implementation in Cytoreductive Surgery (CRS) and Hyperthermic IntraPEritoneal Chemotherapy (HIPEC): Insights from Italian Peritoneal Surface Malignancies Expert Centers #PO 201

#### Oral communication

M. Robella^1^, A. Sommariva^2^, M. Tonello^2^, F. Borghi^1^, M. Vaira^1^

^1^Candiolo Cancer Institute, FPO - IRCCS - Torino (Italy),

^2^Unit of Surgical Oncology of the Esophagus and Digestive Tract, Veneto Institute of Oncology IOV-IRCCS - Padova (Italy)

**Abstract**

**Background:** Cytoreductive surgery (CRS) combined with Hyperthermic Intraperitoneal Chemotherapy (HIPEC) is a complex procedure that involves extensive peritoneal and visceral resections followed by intraperitoneal chemotherapy. The Enhanced Recovery After Surgery (ERAS) program aims to achieve faster recovery by maintaining pre-operative organ function and reducing the stress response following surgery. A recent publication introduced dedicated ERAS guidelines for CRS and HIPEC with the aim of extending the benefits to patients with peritoneal surface malignancies.

**Methods:** A survey was conducted among 21 Italian centers specializing in peritoneal surface malignancies (PSM) treatment to assess adherence to ERAS guidelines. The survey covered pre/intraoperative and postoperative ERAS items and explored attitudes towards ERAS implementation.

**Results:** All centers completed the survey, demonstrating expertise in PSM treatment. However, less than 30% of centers adopted ERAS protocols despite being aware of dedicated guidelines. Preoperative optimization was common, with variations in bowel preparation methods and fasting periods. Intraoperative normothermia control was consistent, but fluid management practices varied. Postoperative practices, including routine abdominal drain placement and NGT management, varied greatly among centers. The majority of respondents expressed an intention to implement ERAS, citing concerns about feasibility and organizational challenges.

**Conclusions:** The study concludes that Italian centers specialized in PSM treatment have limited adoption of ERAS protocols for CRS±HIPEC, despite being aware of guidelines. The variability in practice highlights the need for standardized approaches and further evaluation of ERAS applicability in this complex surgical setting to optimize patient care.

### PO 202 Impact of body mass index on surgical and oncological outcome after Hyperthermic Intraperitoneal Chemotherapy (HIPEC) #PO 202

#### Oral communication or poster

A.C. Ezanno^1^, O. Poudevigne^1^, J.L. Quesada^2^, J. Abba^3^, B. Malgras^4^, B. Trilling^5^, P.Y. Sage^5^, M. Pocard^6^, C. Arvieux^6^, F. Tidadini^5^

^1^Department of digestive surgery, Begin Military Teaching Hospital - St Mandé (France),

^2^Clinical Pharmacology Unit, INSERM CIC1406, Grenoble Alpes University Hospital - Grenoble (France),

^3^Department of Digestive and Emergency Surgery, Grenoble Alpes University Hospital - Grenoble (France),

^4^Department of digestive surgery, Begin Military Teaching Hospital, - St Mandé (France),

^5^Department of Digestive and Emergency Surgery, Grenoble Alpes University Hospital, - Grenoble (France),

^6^Department of digestive surgery,La Pitié Salpêtrière Hospital, - Paris (France)

**Abstract**

**Background:** Complete cytoreductive surgery with Hyperthermic intraperitoneal chemotherapy (HIPEC) is the standard treatment for patients with peritoneal metastases (PM). In this retrospective observational two-center study, we assessed the impact of patients body mass index (BMI) on surgical and oncological outcomes.

**Methods:** Between 2017 and 2021, 144 patients with PM (all etiologies) were included. Morbimortality at day-30, overall survival (OS) and free-recurrence-survival (RS) were compared according to the patients BMI. The patients were divided into 2 groups (BMI<25, and BMI≥25).

**Results:** The median OS was 71.3 months [63-71.5] and significantly different between groups (p=0.025). The median RFS was 26.8 months [20-35.3] and similar for both groups (p=0.267). OS and RFS were similar after stratification by histology. Multivariate analysis using cox model adjusted on PCI score identified BMI<25 (HR = 2.53 [1.10-5.80]) and male sex (HR = 2.34 [1.11– 4.92]) as predictors of poorer OS. Pseudomyxoma histology (HR = 0.21 [0.07 – 0.63]) identified as a protective factor and ASA score>2 (PH = 2.08 [1.21–3.60]) and number of resection (1.25 [1.09 – 1.43]) as a risk factor of FRS. Complication rates at day-30 were similar between two groups; p=0.094. BMI≥25 group had more digestive fistula (p=0.05) and more readmission at day-90 (p=0.007) but not more reintervention (p=0.723).

**Conclusion:** Morbidity at day-30 was similar for BMI<25, and BMI≥25 patients. Readmissions at day-90 were more frequent in high-BMI group. BMI<25 is deleteriously associated with mortality. BMI, sex, neo-adjuvant chemotherapy and the use of Mitomycin-C in HIPEC were related to OS.

### PO 203 Monocentric experience of interventional radiology approach in treatment of postoperative complications after complete cytoreductive surgery +/- HIPEC #PO 203

#### Oral communication or poster

M. Neuberg^1^, P. Beunon^1^, E. Fernandez De Sevilla^1^, L. Benhaim^1^, L. Tselikas^1^, M. Gelli^1^, I. Sourrouille^1^

^1^Gustave Roussy - Villejuif (France)

**Abstract**

**Introduction:** Management of peritoneal malignancies is based on complete cytoreductive surgery (CRS) +/-HIPEC, which is associated with high postoperative morbidity rates. Intraabdominal complications may be treated using a conservative approach based on interventional radiology (IR) to avoid surgical reoperation, with unknown success rate.

**Objectives:** Assessment of efficacy of IR postoperative treatment of intraabdominal complications after CRS+/-HIPEC.

**Methods:** All consecutive patients who received an IR postoperative treatment of intraabdominal complications after CRS+/-HIPEC (2010-2023) were included in this retrospective monocentric study.

**Results:** We included 101 patients who underwent CRS+HIPEC (75%,n=76), CRS alone (23%,n=23) or CRS+CIPPI (2%,n=2), for peritoneal metastases (50%, n=51), pseudomyxoma peritonei (35%, n=35) or peritoneal mesothelioma (15%, n=15). Median age was 55yo [46;61], and median PCI 13 [6;23]. We performed 164 IR procedures (mostly percutaneous drain (n=153), but also arterial embolization (n=6), ppercutaneous pyelostomy (n=2) or pleural drains (n=2)). Repeated IR procedures were performed in 41 patients (41%), with 2 procedures in 24 (24%), 3 procedures in 12 (12%) and 4 procedures in 5 (5%).

We had to reoperate 55 patients in the postoperative course, but only 25 (25%) of these reoperations occurred after IR procedure, assessing failure of this approach. In univariate analysis, predictive factors of failure of IR procedure were multiple anastomosis during CRS (p=0.037), splenectomy (p=0.025), use of 2 intraperitoneal drugs (p=0.006) and performance of >3 IR procedures (p=0.023). Neither morphological characteristics of intraabdominal collections (number, size, localization) nor fluid characteristics (aspect, bacteriological analysis) appeared as predictive factors. We identified no independent predictive factor in multivariate analysis. Two postoperative deaths occurred in patients with IR procedure failure (1 respiratory failure, 1 hemorrhagic shock).

**Conclusions:** IR postoperative treatment success rate of 75% is encouraging. Failures do not seem to be predictable based on morphological and clinical characteristics.

### PO 204 Impact of the SCODA Program (Surgical Complication Optimization through Diet and Activity) on Outcomes in Cytoreductive Surgery with or without HIPEC #PO 204

#### Oral communication or poster

A. Souadka^1^, O. Lahnaoui^1^, A. Benkabbou^1^, M.A. Majbar^1^, A. Ghannam^2^, Z.H. Belkhadir^2^, R. Mohsine^1^, A. El Fassi^2^, B. El Ahmadi^2^

^1^Surgical Oncology Department, National Institute of Oncology, University Mohammed V, Rabat, Morocco. - Rabat (Morocco),

^2^ICU and anesthesiology Department, National Institute of Oncology, University Mohammed V, Rabat, Morocco. - Rabat (Morocco)

**Abstract**

**Background:** The SCODA Program, a low-cost prehabilitation initiative involving structured physical activity and nutritional strategies, aims to optimize outcomes for patients undergoing cytoreductive surgery with or without HIPEC. This program, especially significant in settings with limited resources, includes elements designed to address common perioperative challenges, such as blood shortages.

**Methods:** In this retrospective study of a prospective database, we compared two cohorts of 83 patients (who underwent cytoreductive surgery with or without HIPEC) each, before and after implementing the SCODA Program, which includes 90-minute daily walks, a hypercaloric diet, and systematic iron supplementation to mitigate the effects of blood shortages. We evaluated the impact on pulmonary complications, major complications (grade 3b), ICU stay durations, and perioperative transfusion rates.

**Results:** The introduction of the SCODA Program led to significant reductions: pulmonary complications decreased from 11 to 2 (p=0.021, 81.82% reduction), major complications from 18 to 8 (p=0.055, 55.56% reduction), median ICU stay from 5 days to 1.5 days (p<0.001, 70% reduction), and transfusions from 17 to 7 patients (p=0.047, 58.82% reduction).

**Conclusion:** The SCODA Program, by leveraging low-cost interventions such as enhanced nutrition and exercise along with strategic iron supplementation, significantly improved surgical outcomes. Its successful implementation highlights the potential of cost-effective prehabilitation measures to enhance patient recovery and manage resource constraints effectively, particularly in the context of blood shortages.

### PO 205 Cutaneous panniculitis post hyperthermic intraperitoneal chemotherapy with mitomycin C (MMC-HIPEC), a rare and unrecognized side effect #PO 205

#### Oral communication or poster

E. Ribereau-Gayon^1^, O. Glehen^1^, O. Harou^1^, D. Dalle^1^, C. Theillac^1^, B. Reynaud^1^, S. Debarbieux^1^

^1^Hospices Civiles de Lyon - Lyon (France)

**Abstract**

**Background:** Mitomycin (MMC) is an alkylating cytotoxic chemotherapy agent known for causing skin side effects after cutaneous extravasation. We report six new cases of previously undescribed delayed cutaneous toxicity.

**Methods:** This retrospective, single-center study included patients who developed skin lesions within three months post-MMC-HIPEC. We collected their clinical, biological, histological data and clinical evolution.

**Results:** Six female patients treated between 1989 and 2024 (2800 procedures) presented with skin lesions appearing on average 1.5 months after MMC-HIPEC, located on the flanks and/or inguinal regions around drain sites. Lesions were erythematous, inflammatory, painful, and sclerotic, with one case of ulceration. CRP was high in all cases and 4 patients had fever. Biopsies performed in 3 cases revealed septal or mixed panniculitis with neutrophil predominance, and vascular alterations in two cases. Microbiological tests were negative, except for one case in which Alternaria grew in culture (considered as a sample contamination). Antibiotics (n=5) or antifungals (n=1) did not alter the initial course. Corticosteroid therapy in three patients led to significant improvement within 24-72h, with no recurrence after a six-week tapering. The remaining three patients improved spontaneously.

**Conclusions:** This is the first report of inflammatory panniculitis post-MMC-HIPEC initially suspected as infectious dermohypodermitis. Previous reports described genital necroses primarily in men, about two months post-MMC-HIPEC, and delayed cutaneous toxicity from intravenous MMC, presenting as ulcers and necrotic eschars. This adverse effect seems rare. The persistence of MMC in tissues for over 30 days may explain the delayed onset.

### PO 206 Safety and Efficacy of Total Parental Nutrition in Patients with Peritoneal Surface Malignancies undergoing Palliative Gastro-intestinal Surgery #PO 206

#### Oral communication or poster

M.V. Tan^1^, W.J. Fong^2^, E. Salazar^3^, C.C.M. Cheah^3^, M.Z. Cai^2^, C.J. Seo^2^, J.C.A. Ong^2^, S.C. Chia^2^, J.S.M. Wong^2^

^1^National University of Singapoore, Yong Loo Lin School of Medicine - Singapore (Singapore),

^2^Department of Sarcoma, Peritoneal and Rare Tumours (SPRinT), Division of Surgery and Surgical Oncology, National Cancer Centre Singapore & Singapore General Hospital - Singapore (Singapore),

^3^Department of Gastroenterology and Hepatology, Singapore General Hospital - Singapore (Singapore)

**Abstract**

**Background:** Palliative gastrointestinal surgery is performed in up to 40% of patients with advanced peritoneal surface malignancies (PSM), who are often malnourished and prone to poor post-surgical outcomes. Total parenteral nutrition (TPN) has been used to optimise nutrition and outcomes in curative PSM surgeries, but its safety and efficacy in palliative PSM patients are understudied. We aim to evaluate TPN’s safety and efficacy in improving nutritional states of PSM patients undergoing palliative gastrointestinal surgery.

**Methods:** A retrospective review was conducted on PSM patients undergoing palliative gastrointestinal surgery for bowel obstruction between April 2020 to March 2024. TPN’s safety was measured by TPN-related complications. Efficacy was measured by comparing changes in serum albumin(g/dL) peri-operatively using one-way analysis of variance. Baseline albumin was measured on the day of index surgical admission, pre-operative albumin was measured one day before surgery and post-operative albumin was measured at discharge.

**Results:** 93 PSM patients underwent palliative gastrointestinal surgery. 41%(N=38) received peri-operative TPN; 18%(N=17) received TPN pre- and post-operatively, and 24%(N=21) post-operatively. Median peri-operative TPN duration was 10 days(range=3-141). 16%(N=6) experienced TPN-related complications, including line-related infections(N=3) and minor electrolytes derangements(N=3). Figure 1 depicts serum albumin trends. Pre-operative albumin levels were higher in patients without TPN compared to those with pre- and post-operative TPN and post-operative TPN only(32.7 vs. 31.4 vs. 29.6, p=0.097). Post-operatively, all groups had no difference in albumin levels(32.4 vs. 31.4 vs. 32.2, p=0.882).

**Conclusion:** Peri-operative TPN is safe and is associated with maintenance of nutritional state among malnourished PSM patients undergoing palliative gastrointestinal surgery.

Trend of serum albumin comparing 3 patient groups

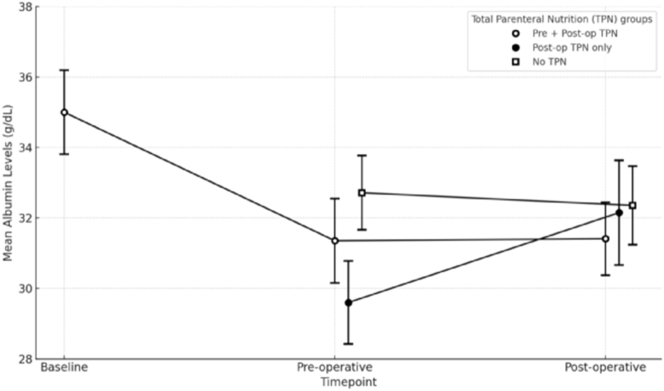



## Peritoneal mesothelioma

### PO 55 Bromelain and N-acetylcysteine combined with hyperthermic chemotherapy in mesothelioma cell lines #PO 55

#### Oral communication or poster

M. Dietz^1^, S. Badar^1^, J. Akhter^1^, S. Valle^1^, D. Morris^2^

^1^Mucpharm Pty Ltd., Sydney, NSW 2217, Australia - Sydney (Australia),

^2^Mucpharm Pty Ltd., Sydney, NSW 2217, Australia; - Sydney (Australia)

**Abstract**

**Background:** Peritoneal mesothelioma (PeM) is a rare malignancy with a poor prognosis. Recurrence rates after cytoreductive surgery with hyperthermic intraperitoneal chemotherapy (CRS-HIPEC) are high. Local additional therapy with bromelain and N-acetylcysteine (NAC) could improve the efficacy of HIPEC. This preclinical in-vitro study aimed to determine the efficacy of bromelain and NAC alone and combined with chemotherapeutic agents in mesothelioma cell lines.

**Methods:** YOU and MSTO-211h cells were seeded in 96-well plates. After 24 hours of incubation cells were exposed to the drugs for two hours at 37°C or 42°C. The treatment regimens consisted of bromelain, NAC, bromelain with NAC, MMC, paclitaxel, or the relevant combinations. After 72 hours of incubation, sulforhodamine B (SRB) assays were performed to determine the cell viability.

**Results:** Single-agent treatment with bromelain demonstrated a cytotoxic effect on YOU and MSTO-211h cells in a dose-dependent manner. For NAC, a cytotoxic effect was only observed at higher concentrations. The combination of bromelain with NAC increased cytotoxicity compared to single-agent treatments. Both YOU and MSTO-211h cells were sensitive to MMC and paclitaxel, and combination with bromelain and NAC resulted in an increase in cytotoxicity.

**Conclusions:** Bromelain and NAC increases the efficacy of MMC and paclitaxel at hyperthermia in two mesothelioma cell lines. Future studies are needed to confirm these results in vivo. If the addition of bromelain with NAC would increase the efficacy of HIPEC this could improve microscopic cytoreduction and would reduce the risk of disease recurrence.

### PO 56 Optimizing the pathological diagnosis of diffuse malignant mesothelioma #PO 56

#### Oral communication

F.L. Nava^1^, S. Kusamura^1^, M. Shimonovitz Moore^2^, M. Millione^1^, D. Baratti^1^, M. Guaglio^1^, A. Cabras^3^, G. Colletti^1^, M. Deraco^1^

^1^Fondazione IRCCS Istituto Nazionale Tumori Milano - Milano (Italy),

^2^Tel Aviv Medical Center - Tel Aviv (Israel),

^3^Mater Olbia Hospital - Olbia (Italy)

**Abstract**

**Background:** Diffuse Malignant Peritoneal Mesothelioma (DMPM) presents diagnostic challenges due to its rarity and intra-tumoral heterogeneity, impacting treatment choices and prognosis. This study aimed to evaluate factors influencing diagnostic accuracy in identifying sarcomatoid components and defining Ki67.

**Methods:** The concordance between preoperative and post-CRS-HIPEC histotypes definition and Ki67 assessment was calculated with k-index on our retrospective single center series of 98 DMPM. The influence of biopsy type (core-needle vs. surgical), number (single vs. multiple) and specimen volume (n° of blocks) on the concordance of histotype and Ki67 was evaluated by Fisher’s exact test. Sensitivity (Se), specificity (Sp), positive-predictive-value (PPV), and negative-predictive-value (NPV) of core-needle vs. surgical biopsy in identifying sarcomatoid components and Ki67 assessment were calculated.

**Results:** Ki67 assessment resulted in being moderately concordant (k-index=0.4) between preoperative and post-CRS-HIPEC pathological assessment, while the histotype discrimination (epithelioid vs. sarcomatoid in DMPM) showed an optimal concordance (k-index=0.8). No difference in histotype or Ki67 emerged between core-needle and surgical biopsies (p-value=0.60 and p-value=1.00, respectively). The accuracy of preoperative pathology in defining histotype and Ki67 was not influenced by the number of biopsied sites (p-value=0.16, histotype; p-value=0.50, Ki67), nor by their specimen volume (p-value=0.30, histotype; p-value=0.60 Ki67). Surgical biopsy showed Se=80.0%, Sp=98.0%, PPV=80.0%, and NPV=98% in recognizing the sarcomatoid components and Se=88.0%, Sp=79.2%, PPV=81.5%, and NPV=86.4% in assessing Ki67. Core-needle biopsy demonstrated Se=50%, Sp=100.0%, PPV=100.0%, and NPV=60.0% in defining the sarcomatoid component; because of the small sample size, the accuracy in Ki67 estimation could not be verified.

**Conclusions:** DMPM diagnosis remains challenging. While surgical biopsy outperformed core-needle biopsy in terms of Se and NPV, the difference was not substantial. Core-needle biopsy could be considered when the tumour nodule is easily accessible by interventional radiology, but careful consideration is warranted given the sample size limitations of the patient cohort.

### PO 57 Epithelioid malignant mesothelioma discovered following peritonitis: About a case #PO 57

#### Poster

E. Jmal^1^, O.S.M.A.N. Osman^1^, F. Ennaceur^1^, N. Feriani^1^, W. Mkhinini^1^, M. Hedfi^1^

^1^Zaghouan regional hospital - Zaghouan (Tunisia)

**Abstract**

**BACKGROUND:** Malignant mesothelioma is a rare growth of mesothelial cells strongly associated with asbestos exposure.Epithelioid mesothelioma is a form of malignant mesothelioma made up of epithelioid cells.It is the most common mesothelioma cell type.The diagnosis of peritoneal mesothelioma is suspected when a peritoneal carcinomatosis is fortuitously discovered without an obvious primary tumor.

**CASE REPORT:** A 77-year-old male patient, with no medical history, was admitted for increased abdominal pain and diarrhea for two weeks. He denied any systemic chronic disease and previous surgeries.On physical examination, he had a low-grade fever. A generalized abdominal tenderness was also reported.A complete blood count revealed a white cell count of 34650/uL and CRP of 248. Abdominal ultrasound examination showed moderate effusion.A CT- SCAN showed peritonitis with peri-splenic and peri-hepatic effusion.

An emergency exploratory laparotomy was conducted. Intra operative assessment revealed a suspicious effusion of great abundance and an abscess at the root of the mesentery with whitish micronodules in the visceral and parietal peritoneum.A biopsy was performed and has shown an epithelioid malignant mesothelioma.Our patient had a cytoreductive surgery.No visceral resections were required. He did not received an intraperitoneal chemotherapy.

Postoperative outcome was uneventful. The patient is currently under observation

**CONCLUSION:** Peritoneal mesothelioma is a rare primary tumor of serous membranes, which habitually carries a poor prognosis. It is generally discovered in the advanced phase of the disease, due to the variety of clinical and radiological findings, which worsens its prognosis even further. Diagnostic laparoscopy with biopsy and immunohistochemistry are considered the gold standard for the diagnostic’s confirmation.

Patients diagnosed with epithelioid mesothelioma are often eligible for more treatment options than the other cell types. These tumors typically respond well to treatment. The primary treatments are surgery, chemotherapy and immunotherapy. However, many factors can affect a patient’s specific treatment plan, especially the type of mesothelioma.

### PO 58 Long-Term Survival in MPM Patients Treated with CRS+HIPEC: A Retrospective Study of Two Treatment Centers #PO 58

#### Oral communication or poster

X.L. Liang^1^, Y. Li^2^

^1^+8615030413619 - Beijing (China),

^2^+8618612709123 - Beijing (China)

**Abstract**

**Objectives:** To explore the survival benefit factors of malignant peritoneal mesothelioma (MPM) patients after cytoreductive surgery (CRS) plus hyperthermic intraperitoneal chemotherapy (HIPEC), and apply conditional survival (CS) to provide dynamic survival prediction.

**Methods:** Data of 212 MPM patients who underwent CRS+HIPEC at two centers from 2015 to 2024 were retrospectively analyzed. Median overall survival (OS) for the entire cohort was 28.5 months, with 25th and 75th percentiles at 16.0 and 47.7 months. Patients were divided into LTS group (≥48.0 months) and short-term survival (STS) group (≤16.0 months) according to OS. CS is the probability of surviving y years after already surviving for x years, which is calculated by the formula CS_(x|y)_=S_(x+y)_/S_(x)_. Univariate and multivariate analyses were performed to explore the favorable factors of MPM patients with LTS. CS and Kaplan-Meier were applied to assess the postoperative survival probability.

**Results:** 90 patients were enrolled; 53 (58.9%) of them were LTS and 37 (41.1%) were STS. In LTS and STS groups, median OS was 110.3 (48.5-272.7) *vs.* 11.0 (3.0-15.4) months. Univariate analysis revealed 14 factors (*P*<0.05) with statistically significant differences: age, surgery history, Karnofsky performance status, pathological types, tumor vascular emboli, lymphatic metastasis, Ki-67 index, preoperative CA125 level, peritoneal cancer index (PCI), completeness of cytoreduction, bleeding volume, red blood cell (RBC) transfusion, ascites and severe adverse events (SAEs). Multivariate analysis identified that PCI≤20, less RBC transfusion and no SAEs were LTS independent prognostic factors. 5-year CS increased over time from 27% at 0-year to 84% at 4-year. The survival curve seem to flatten at 5 years after surgery.

**Conclusions:** PCI≤20, less intraoperative RBC transfusion, and no SAEs are independent factors for LTS in MPM patients. The probability of achieving 5-year OS after CRS+HIPEC for MPM patients increases with each additional year survived. Some patients may achieve clinical cure after postoperative year 5.

### PO 59 Cytoreductive Surgery and Hyperthermic Intraperitoneal Chemotherapy for Peritoneal Mesothelioma: Outcomes from a Tertiary Cancer Care Center in Northern India #PO 59

#### Oral communication

B. Pathak^1^, M. Ray^1^

^1^AIIMS - New Delhi (India)

**Abstract**

**Background:** Malignant peritoneal mesothelioma (MPM) is a rare and aggressive form of cancer originating from the peritoneum. The prognosis for MPM has historically been poor, and treatment options are limited. This study evaluated the impact of cytoreductive surgery (CRS) combined with hyperthermic intraperitoneal chemotherapy (HIPEC) as a treatment modality for MPM, although optimal management is still evolving.

**Materials and Methods:** This retrospective analysis included fifteen patients diagnosed with MPM between 2012 and 2023 at a tertiary referral cancer care center in North India. Patients underwent CRS followed by HIPEC. The study assessed outcomes based on overall survival (OS) and postoperative morbidity rates.

**Results:** Demographic analysis revealed a female preponderance (60%) and a majority of younger patients, 80% of whom were younger than the age of 50. Neoadjuvant chemotherapy was infrequent (13.33%), while the most common histopathological subtype was epithelioid (66.67%). The mean peritoneal cancer index (PCI) was 14.0, with 60% of patients having a PCI above the mean. The completeness of cytoreduction (CC) varied, with 40% achieving CC0, 33.33% CC1, and 26.67% CC2. Adjuvant chemotherapy was administered to 60% of the patients. The mean blood loss was 577 ml, and the mean operation duration was 350 minutes. Postoperative complications ranged from mild to life-threatening, with a mortality rate of 6.67%. The median follow-up period was 25 months, revealing an overall median survival of 27.0 months, with 1- and 3-year survival rates of 86.7% and 33.3%, respectively. On univariate analysis, only histological subtype emerged as a predictive factor for overall survival.

**Conclusion:** CRS combined with HIPEC is a viable and effective treatment option for patients with MPM and offers improved survival rates and an acceptable safety profile. These findings support the integration of this treatment modality into the management plan for select patients with MPM, although optimal management is still evolving.

### PO 60 Clinopathological outcomes of immunotherapy for malignant peritoneal mesothelioma patients--a 4-year review of national multidisciplinary team video-conference meeting #PO 60

#### Oral communication or poster

S.Y. Kok^1^, S. Westbrook^1^, T. Cecil^1^, S. Dayal^1^, A. Tzivanakis^1^, B. Moran^1^, F. Mohamed^1^, M. Remner^1^

^1^Peritoneal Malignancy Institute, Basingstoke and North Hampshire Hospital, Basingstoke - Basingstoke (United Kingdom)

**Abstract**

**Background:** Malignant peritoneal mesothelioma (MPM) is a rare disease. Cytoreductive Surgery and Hyperthemic Intraperitoneal Chemotherapy (CRS+HIPEC) is the mainstay of treatment for surgically resectable disease. For non-surgical candidates, new advancement especially immunotherapy (Nivolumab and ipilimumab) has proven improvement in overall survival in recent publication. This article aims to review the clinicopathological outcomes of MPM patients receiving immunotherapy from 2020-2023 retrospectively.

**Method:** Patients with MPM were discussed in a monthly national multidisciplinary team video-conferencing meeting (NMDT) between 4 tertiary referral centres in England and Ireland. Non-surgical patients were referred to oncologist for fitness for immunotherapy. Patients were rediscussed in NMDT for review of progress and feasibility of CRS+HIPEC in between or after Immunotherapy. Histology, treatment indication, type of immunotherapy used, side-effect, disease progression, proceeding to CRS+HIPEC and 1-year overall survival were evaluated.

**Result:** From 2020-23, 46 patients (M:F 20:26) with mean age 63 had immunotherapy. 32 (69.6%) had Nivolumab and ipilimumab whereas 13 (28.3%) had Nivolumab only.

93% (41) were Epithelioid. Immunotherapy was given due to post CRS+HIPEC recurrence: 6 (13%), disease progression on chemo: 13 (28%), as 1st line for non surgical disease: 24 (52%) and for pre & post CRS + HIPEC treatment: 3 (35%). 17 (37%) patients had side-effect and need to stop or change to chemotherapy. 19 (41.3%) had disease progression during immunotherapy. The median overall survival was 531 days and 1 year overall survival was 86.2%

4 patients (8.7%) proceeded to CRS+HIPEC after immunotherapy. CC1 (2), CC2 (1), CC3 (1). Median Peritoneal Cancer Index was 23. 100% has Clavien-Dindo Grade 1 complication with No postoperation mortality. 1 patient were referred for immunotherapy postoperation.

**Conclusion:** Immunotherapy has potential survival benefit and disease optimisation for MPM patients to CRS+HIPEC. Further study and long-term follow-up to evaluate its safety and efficacy is warranted

### PO 61 Outcomes of CRS/HIPEC fore peritoneal mesothelioma after conversion to surgery by bidirectional chmotherapy #PO 61

#### Oral communication or poster

I. Sourrouille^1^, P. Combari^1^, C. Smolenschi^1^, E. Fernandez De Sevilla^1^, V. Boige^1^, M. Gelli^1^

^1^Gustave Roussy - villejuif (France)

**Abstract**

**Background:** Curative treatment of peritoneal mesothelioma relies on complete cytoreductive surgery (CRS) with HIPEC. In case of extended disease, peritoneal intensification by bidirectional chemotherapy has been described with encouraging results, but unknown consequences on outcomes of CRS/HIPEC.

**Objectives:** We aimed to compare outcomes of upfront CRS/HIPEC with those of CRS/HIPEC performed after bidirectional chemotherapy (BDC).

**Results:** From 2012 to 2024, 68 patients had CRS/HIPEC for peritoneal mesothelioma, 39 upfront (HIPEC group) and 29 after BDC (BDC/HIPEC group), length of surgery was

Median PCI (20 [13;26] in BDC/HIPEC vs 15[10-23] in HIPEC group), length of surgery (540min [380;640] vs 500min [315;600]), intraoperative blood loss (400ml [200;1000] vs 300ml [150;700]) were similar between the two groups. Two patients had a CC2 surgery, all in BDC/HIPEC group. Major morbidity rates (41%,n=12 vs 48%,n=13) and mortality rates (0% vs 2.5%,n=1) were also similar.

**Conclusion:** CRS/HIPEC is feasible after bidirectional chemotherapy, with no negative impact on peroperative or postoperative outcomes.

### PO 62 Recurrence Patterns after CRS/HIPEC in Malignant Peritoneal Mesothelioma and Management Strategies: a Single-Institution Experience #PO 62

#### Poster

P.K. Koukoutsidi^1^, M.D. Goodman^1^

^1^Tufts Medical Center - Boston (United States)

**Abstract**

**Background:** Complete cytoreduction (CC0/CC1) combined with hyperthermic intraperitoneal chemotherapy (HIPEC) is the standard of care for malignant peritoneal mesothelioma (MPM). Despite optimal CC, disease recurrence remains a challenge. This study presents MPM recurrence patterns and recurrence management approaches for patients who underwent CC/HIPEC at our institution.

**Methods:** Retrospective analysis of a prospectively maintained database of patients treated at the Division of Surgical Oncology identified patients with a diagnosis of MPM treated with CC/HIPEC from 2007 to 2022. Ongoing surveillance for recurrence was conducted among alive patients. For deceased patients, follow-up data was thoroughly reviewed.

**Results:** 385 records were screened and 23 cases of epithelioid MPM were identified. CC/HIPEC was achieved in 20/23 cases. Two (2) patients died within 1 year of CC/HIPEC and 2 patients were lost to follow up postoperatively. Recurrence was radiographically diagnosed in 12/16 (75%), with mean time to recurrence of 24.2 months. Recurrence patterns included:

-6 patients with peritoneal recurrent disease, with pelvis being the most prevalent site,

-3 patients with combined peritoneal and pleural recurrent disease,

-2 patients with pleural recurrent disease,

-1 patient with liver recurrence.

**Figure 1** outlines the management strategies after recurrence was diagnosed.

**Conclusions:** Our single-institution experience indicates the lack of consensus in managing MPM recurrent disease. Given the rarity of MPM, available clinical information is limited and often extrapolated from patients with pleural mesothelioma. Management strategies are more personalized and usually guided by factors such as the site of recurrent disease, patient clinical status and the rate of disease progression.

Figure 1: Management Strategies in Recurrent MPM

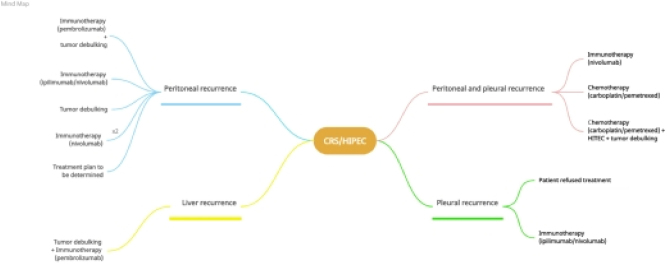



## Peritoneal metastases from colorectal cancer

### PO 91 Survival of patients with peritoneal versus non-peritoneal metastatic colorectal cancer given first-line palliative systemic therapy: a nationwide registry-based cohort study. #PO 91

#### Oral communication

T.M.E. Kerkhoff^1^, F.N. Van Erning^2^, S. Nienhuijs^3^, J.W.A. Burger^3^, I.E.G. Van Hellemond^4^, N.F.M. Kok^5^, C. Verhoef^6^, P.J. Tanis^6^, I.H.J.T. De Hingh^7^, K.P. Rovers^7^

^1^Departmen of Surgery, Catharina Hospital - Eindhoven (Netherlands),

^2^Department of Research and Development, Netherlands Comprehensive Cancer Organization - Utrecht (Netherlands),

^3^Department of Surgery, Catharina Hospital - Eindhoven (Netherlands),

^4^Department of Medical Oncology, Catharina Hospital - Eindhoven (Netherlands),

^5^Department of Surgery, Netherlands Cancer Institute - Amsterdam (Netherlands),

^6^Department of Surgery, Erasmus University Medical C|enter - Rotterdam (Netherlands),

^7^Department of Surgery, Catharina Hospitall - Eindhoven (Netherlands)

**Abstract**

**Background:** the prognostic impact of peritoneal metastases in systemically treated metastatic colorectal cancer (CRC) patients has been investigated in older first-line trials with underrepresentation of peritoneal metastases, limiting generalizability to the real-world population in current-day practice. This study investigated the association between peritoneal metastases and survival in a real-world population of patients receiving first-line palliative systemic therapy for metastatic CRC.

**Methods:** this nationwide Netherlands Cancer Registry-based cohort study included all Dutch patients who started with first-line palliative systemic therapy for synchronous metastatic CRC diagnosed from 2009 through 2021. The primary outcome was the survival difference between patients with peritoneal and non-peritoneal metastatic CRC. The association between peritoneal metastases and survival was assessed using Cox regression adjusting for clinicopathological covariates.

**Findings:** Of 12282 included patients, 2990 (24%) had peritoneal metastases. Compared to patients with non-peritoneal metastatic CRC, those with peritoneal metastatic CRC more frequently had right-sided tumors (54% versus 34%, p<0·0001), signet ring cell histology (6% versus 1%, p<0·0001), poorly differentiated tumors (35% versus 19%, p<0·0001), BRAF mutations (25% versus 13%, p<0·0001), and ≥3 metastatic sites (31% versus 11%, p<0·0001). Though patients with peritoneal metastatic CRC had worse survival than those with non-peritoneal metastatic CRC (median 10·8 [95% CI 10·2-11·1] versus 14·0 [95% CI 13·7-14·3] months, HR 1·35 [95% CI 1·29-1·41], p<0·0001), the presence of peritoneal metastases was not associated with survival after covariate adjustment (adjusted HR 1·04 [95% CI 0·99-1·09], p=0·1062).

**Interpretation:** in patients receiving first-line palliative systemic therapy for metastatic CRC, the survival difference between those with and without peritoneal metastases is related to unfavorable tumor characteristics that are more frequently present in patients with peritoneal metastases. These results challenge the widely accepted assumption that the poor prognosis of systemically treated patients with colorectal peritoneal metastases is due to the presence of peritoneal metastases itself.

### PO 92 Survival outcomes of patients with synchronous colorectal peritoneal metastases treated with “standard” therapy in Japan #PO 92

#### Poster

H. Nagata^1^, T. Kato^1^, Y. Takamizawa^1^, K. Moritani^1^, S. Tsukamoto^1^, A. Takashima^1^, Y. Kanemitsu^1^

^1^National Cancer Center Hospital - Tokyo (Japan)

**Abstract**

**Background:** Management of colorectal peritoneal metastasis (CRPM) is clinically challenging. While cytoreductive surgery has been proposed as a promising treatment, not a few surgeons still hesitate to adopt it partly because its superiority to state-of-the-art chemotherapy is not necessarily clear. This study aimed to clarify the latest outcomes of patients with synchronous PM treated with current “standard” therapy in Japan.

**Methods:** This historical cohort study included patients with CRPM actively treated in our hospital between 2017 and 2021 following Japanese guidelines, which basically recommend systemic chemotherapy and admit the excision of peritoneal nodules only when the disease is highly localized.

**Results:** CRPM was clinically diagnosed in 165 patients, and the peritoneum was the only site of metastases in 56 patients. Peritoneal cancer index (PCI) based on CT images before initial treatments was no more than 20 in 46 patients. R0 resection was performed in 17 patients (37.0%), and five patients achieved 5-year survival (30.0%), while the median OS of patients without R0 resection was 33.6 months, and none witnessed 5-year survival. OS was significantly better in patients with R0 resection than those without it (Hazard ratio 0.23, 95% confidence interval:0.08-0.65). Surgical PCI before treatment was available and no more than 20 in 43 patients. R0 resection was performed in 20 patients (46.5%), and seven patients achieved 5-year survival (60.2%). On the other hand, the median OS of patients without R0 resection was 35.8 months, and none had 5-year survival. OS was significantly better in patients with R0 resection than those without it (Hazard ratio 0.32, 95% confidence interval: 0.12-0.85).

**Conclusions:** This study reported the latest OS of CRPM without cytoreductive surgery. Median OS seemed better than previous reports, presumably due to advances in chemotherapy, whereas 5-year survival was shown to be hardly achievable without surgery.

### PO 93 First-line palliative systemic therapy alternated with oxaliplatin-based Pressurized Intraperitoneal Aerosol Chemotherapy for unresectable colorectal peritoneal metastases: a single-arm phase II trial (CRC-PIPAC-II). #PO 93

#### Oral communication

V.C.J. Van De Vlasakker^1^, P. Rauwerdink^2^, R.J. Lurvink^1^, E. Wassenaar^2^, D. Boerma^2^, I.H.J. De Hingh^1^

^1^Catharina Hospital Eindhoven - Eindhoven (Netherlands), ^2^St. Antonius Hospital - Nieuwegein (Netherlands)

**Abstract**

**Background:** Palliative systemic therapy alternated with electrostatic precipitation oxaliplatin-based pressurized intraperitoneal aerosol chemotherapy (ePIPAC) has never been prospectively investigated in patients with unresectable colorectal peritoneal metastases (CPM). The CRC-PIPAC-II study aimed to assess safety, feasibility and efficacy of such bidirectional therapy.

**Methods:** This two-center, single-arm, phase II trial enrolled chemotherapy-naïve patients to undergo three treatment cycles, consisting of systemic therapy (CAPOX, FOLFOX, FOLFIRI, or FOLFOXIRI, all with bevacizumab) and oxaliplatin-based ePIPAC (92mg/m2) with intravenous leucovorin (20mg/m2) and 5-fluorouracil (400mg/m2). Primary outcome were major treatment-related adverse events. Secondary outcomes included minor events, tumor response, progression-free survival (PFS) and overall survival (OS).

**Results:** Twenty patients completed 52 treatment cycles. Fifteen major events occurred in 7 patients (35%): 5 events (33%) related to systemic therapy; 5 (33%) related to ePIPAC; and 5 (33%) were biochemical events. No treatment-related deaths occurred. All patients experienced minor events, mostly abdominal pain, nausea and peripheral sensory neuropathy. After treatment, radiological, pathological, cytological, and biochemical response was observed in 0%, 88%, 38%, and 31% of patients respectively. Curative surgery was achieved in two patients, one of whom unexpectedly diagosed with pseudomyxoma peritonei. Median PFS was 10.0 months (95% confidence interval [CI] 8.0–13.0) and median OS was 17.5 months (95% CI 13.0–not reached).

**Conclusions:** Combining palliative systemic therapy with oxaliplatin-based ePIPAC in patients with unresectable CPM was feasible and showed an acceptable safety profile. Treatment-induced response and survival are promising, yet further research is required to determine the additional value of ePIPAC to systemic therapy.

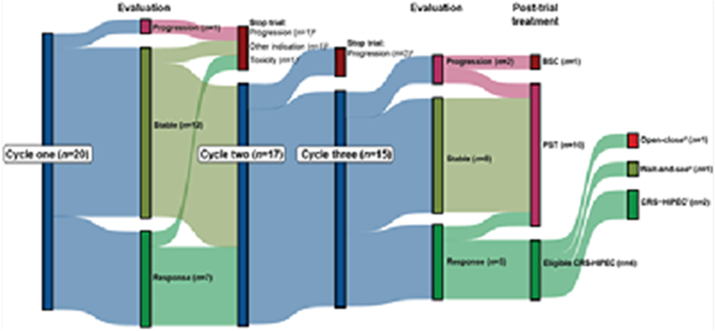



### PO 94 Prospective study of adjuvant oxaliplatin-based PIPAC with concurrent intravenous 5-fluorouracil and folinic acid after curative surgery for pT4a/b colon cancer (Clinicaltrials.gov NCT06091683) #PO 94

#### Oral communication

D. Baratti^1^, M. Guaglio^1^, S. Kusamura^1^, G. Fallabrino^1^, P. Proto^1^, G. Colletti^1^, T. Cavalleri^1^, M. Deraco^1^

^1^Fondazione IRCCS Istituto Nazionale dei Tumori - Milano (Italy)

**Abstract**

**BACKGROUND:** We conducted a prospective single center pilot study to assess feasibility and safety of adjuvant oxaliplatin-based Pressurized Intra-Peritoneal Aerosolized Chemotherapy (PIPAC) after curative surgery for pT4a/b colon cancer. This strategy takes advantage of pathological examination to optimize patient selection, and better drug diffusion and penetration to overcome the (potential) limitations of the postoperative time setting. We also hypothesized that concurrent intravenous 5-fluorouracil and folinic acid (FU/FA) could increase oxaliplatin effect without harm

**METHODS:** Ten patients with pT4a/b, N0-2, M0, R0 colon cancer were enrolled. PIPAC was performed within 4-8 weeks from primary surgery with oxaliplatin (92 mg/m2) and concurrent intravenous 5-FU/FA (400/20 mg/m2). Adjuvant PIPAC was considered feasible if the laparoscopic procedure can be completed in ≥9 patients, and postoperative stay will be ≤3 days in ≥6 patients. Adjuvant PIPAC was considered safe if maximunm one conversion to open surgery, one severe complication (NCI-CTCAE v.4 grade 3-5), and one readmission within 30 days occurred. The trial is registered with Clinicaltrials.gov.NCT06091683.

**RESULTS:** Median age was 59 years (range 41-80). Median interval between primary resection and PIPAC was 6 weeks (range 3-7). The procedure was completed in all patients. Postoperative stay was ≤3 days in all but one patient. One patient had mild (grade 2) transaminase increase. No conversion, severe complication, death, or readmission occurred in the remaining patients. Metachronous peritoneal metastases (undetected at primary surgery) were discovered during PIPAC in one patient. The remaining patients are free of disease after a median of 19 months (range 10-35). Adjuvant systemic chemotherapy was not indicated for two patients (including the one with metachoronous peritoneal metastases), and started within 12 week from primary surgery for the remaining patients.

**CONCLUSIONS:** Adjuvant oxaliplatin-based PIPAC with concurrent intravenous FU/FA after curative surgery for pT4a/b colon cancer is feasible and safe. Preliminary oncological results are promizing.

### PO 95 Peritoneal metastases from mucinous adenocarcinoma of appendiceal vs. colorectal origin. An Italian multicenter comparative study #PO 95

#### Oral communication

D. Baratti^1^, M. Valle^2^, A. Sommariva^3^, D. Marrelli^4^, P. Fugazzola^5^, C. Noventa^6^, F. Casella^7^, M. Tonello^8^, F.L. Nava^1^, M. Deraco^1^

^1^Fondazione IRCCS Istituto Nazionale dei Tumori - Milano (Italy),

^2^Regina Elena Cancer Institute - Roma (Italy),

^3^IRCCS Istituto Oncologico Veneto IOV - Padova (Italy),

^4^Unit of General Surgery and Surgical Oncology University of Siena - Siena (Italy),

^5^Fondazione IRCCS Policlinico San Matteo - Pavia (Italy),

^6^Department of Surgery, ASST Cremona - Cremona (Italy),

^7^Chirurgia Generale dell’Esofago e dello Stomaco, Azienda Ospedaliera Universitaria Integrata - Verona (Italy),

^8^IRCCS Istituto Oncologico Veneto IOVEsofago e dello Stomaco, Azienda Ospedaliera Universitaria Integrata - Padova (Italy)

**Abstract**

**Background:** Among mucinous peritoneal neoplasms, traditional (low-grade) pseudomyxoma peritonei represents a distinct clinical-pathological entity, but it is still unclear if peritoneal metastases (PM) from appendiceal or colorectal mucinous adenocarcinoma (MAC) are clinically and prognostically different. We compared baseline characteristics, outcomes and patterns of failure between patients undergoing cytoreductive surgery and hyperthermic intraperitoneal chemotherapy (CRS/HIPEC) for PM from appendiceal or colorectal MAC.

**Methods:** We collected data for 183 patients treated for PM from appendiceal MAC, and 121 patients treated for PM from colorectal MAC in 13 Italian centers, gathered in a collaborative group of the Italian Society of Surgical Oncology. The study end-points were overall survival (OS), and cumulative incidence of peritoneal recuurences and distant metastases.

**Results:** Appendiceal origin was associated with higher PCI (P<0.001), and higher rates of incomplete cytoreduction (P=0.01), signet ring cell histology (P=0.001), T4a/b primary (P=0.003), negative nodes (P<0.001), synchronous PM presentation (P<0.001), wild-type KRAS (P<0.001), wild-type BRAF (P=0.009), but not wild-type NRAS (P=0.170) and MSI (P=0.162). After a median follow-up of 50 months (95% confidence interval [CI] 40-72), 5-year OS was 60.7% (95% CI 52.7-67.2) for appendiceal origin and 40.1% (95% CI 29.7-54.9) for colorectal origin. The difference was statistically significant (p=0.002). Appendiceal origin correlated with better prognosis also at multivariate analysis (hazard rate 1.86; 95% CI 1.05-3.30; P=0.033). Between appendiceal and colorectal origin, 5-year cumulative incidences of peritoneal recurrences (60.0% vs. 56.3%; P=0.773) and distant metastases (27.8 % vs. 39.1%; P=0.101) were not significantly different.

**Conclusions:** As compared with their colorectal counterpart, PM from appendiceal MAC are associated with distinct clinical-biological features and better prognosis. Although there was a higher (but not significant) cumulative incidence of distant metastases in patients treated for PM of colorectal origin, the patterns of failure after CRS/HIPEC were not statistically different between groups.

### PO 96 Early postoperative immediate chemotherapy (EPIC) prevents diffuse peritoneal carcinomatosis recurrence. #PO 96

#### Oral communication

S. Berkane^1^

^1^Faculté De Médecine D’alger. Service De Chirurgie Viscérale Et Oncologique. Hôpital De Bologhine. Ibn Ziri. Alger - Alger (Algeria)

**Abstract**

**Introduction:** Diffuse peritoneal carcinomatosis recurrence is one treatment failure of advanced colon cancer. The aim of this prospective phase two trial is to evaluate while early postoperative immediate chemotherapy (EPIC) prevents peritoneal carcinomatosis recurrence. **Material and method:** patients with adenocarcinoma of the colon invading the serous layer and/or invading the neighboring viscera and operated on with curative intent (R0 resection) were included. After curative resection, EPIC is added and starts immediately after a closure of abdominal wound with adjunction of 2liters of isotonic saline serum associated with antimitotic drug. The protocol associates 10m/kg of Mitomycin on day one followed by 500mg/day of 5Fluorouracile for 4 days. This procedure is realized for five days. The mean period of follow-up is 120months (60-240months). **Results:** 73 patients, 37males and 36 females with mean age of 57years (24-75years), were included. Four, four and two patients had respectively hepatic, peritoneal and ovarian metastasis. The morbidity and mortality were respectively 26% and 02,7%. The morbidity was dominated by anastomosis fistulas. Complications related to chemotherapy drug were nil. During period follow-up, fourteen patients (10%) experienced recurrences. Only one patient (1,4%) presented diffuse peritoneal carcinomatosis recurrence (this rate is around 28% in our patients without EPIC- Data not shown). In 9 cases, sites of recurrence were liver in 3cases, lymphatic nodes in two cases, pulmonary in one case, bone in one case and surrounding anastomosis in 2cases. Seventy-eight percent of patients (59cases) are alive without disease at this time with mean duration of 96months (60-200months). The 3 and 5year survival rate are respectively 72% and 54%. The 3year survival is 90% and 75% for respectively stage II and stage III. **Conclusion:** It seems to reduce the rate of diffuse peritoneal carcinomatosis recurrence but there is a need to pay a lot attention for anastomosis fistulas.

### PO 97 Perioperative Bevacizumab in patients undergoing Cytoreductive Surgery and HIPEC for colorectal peritoneal metastases: results of a phase II trial (BEV-IP) #PO 97

#### Poster

D.C. Chia^1^, K. Van Der Speeten^2^, G. Liberale^3^, F. Vansteenkiste^4^, K. De Meuleneir^5^, S. Cosyns^6^, W. Willaert^6^, W.C. Ceelen^1^

^1^Department of Human Structure and Repair, Ghent University, Lab of Experimental Surgery - Gent (Belgium),

^2^Department of Surgical Oncology, Ziekenhuis Oost-Limburg - Genk (Belgium),

^3^Department of Surgical Oncology, Institut Jules Bordet, Université Libre de Bruxelles - Brussels (Belgium),

^4^Department of Digestive Surgery, Groeninge Hospital - Kortrijk (Belgium),

^5^Department of GI Surgery, Ghent University Hospital - Ghent (Belgium),

^6^Department of Human Structure and Repair, Ghent University, Lab of Experimental Surgery - Ghent (Belgium)

**Abstract**

**Introduction:** While adding bevacizumab(BEV) to systemic chemotherapy may improve outcomes in colorectal cancer peritoneal metastases(CR-PM),its impact on morbidity/mortality after CRS/HIPEC remains unknown.The BEV-IP study evaluated the safety/efficacy of perioperative BEV.(NCT02399410;EudraCTnumber:2015-001187-19)

**Methods:** Eligible CR-PM patients received standard neoadjuvant-therapy(NACT) and BEV(>6-biweekly administrations).Adjuvant BEV was recommended subject to the oncologist/patient.Primary-endpoints were 3-month morbidity/mortality(Clavien-dindo[CD]).Secondary-endpoints included treatment-related toxicity(CTCAE),NACT completion(%),progression-free survival(PFS),overall survival(OS) and patient-reported quality-of-life outcomes(PRQOL:[EORTC QLQ-C30,SF36]).From literature(major morbidity:27%,mortality:3.5%) and using effects methods;a sample size of n=45 yielded 95%-CIs of 14%-40% and 0.8%-14% respectively.

**Results:** Of 60 patients(33.3% synchronous,66.7% metachronous),baseline PCI(median,[IQR]):11(3-19),90%(54/60) received >1cycle of BEV.Before CRS/HIPEC, patients received a median(IQR) of 6(6-7) and 4(4-6) cycles of systemic chemotherapy and BEV;severe adverse events(SAEs;CTCAE > 3) occurred in 16.7%(9/54).CTCAE-G5 complications and disease-progression occurred in 3.7%(2/54) and 3.7%(2/54).Of 51 remaining patients,the median(IQR) PCI at laparotomy was 8(4-10).Major complications(CD>3B) occurred in 33.3%(17/51) including 5 anastomotic leaks (AL).The 90-day mortality after CRS/HIPEC was 5.9%(3/51).SAEs occurred in 15.7%(8/51) of patients receiving a median(IQR) 5(3-6) cycles of adjuvant systemic chemotherapy and 2(0-6) cycles of BEV.NACT was completed in 72.2%(39/54) of patients;nineteen patients(37.3%) received>6 cycles of BEV,with higher AL trend(21.1% vs. 3.1%,p=0.058).Over 71.4m follow-up,the median(IQR) PFS/OS was 14.9m(10.7-29.5) and 38.7m(17.3-90.0).Completing NACT did not improve the median PFS (Complete vs. incomplete NACT,months[IQR],14.1[11.4-29.5]vs.14.9[5.8-24.2],p=0.624) or OS (Complete vs. incomplete NACT,months[IQR],42.5[17.3-90.0]vs.29.2[12.4-47.1],p=0.751).Social-function scores(SF36) improved 6-months post-adjuvant chemotherapy compared to post-CRS/pre-NACT (88 vs. 39 vs. 62, p<0.05). All other SF36 domains/EORTCQLQ-C30 were comparable between baseline and 6-months post-CRS.

**Conclusion:** Perioperative oxaliplatin-based chemotherapy with BEV resulted in an acceptable morbidity rate.The combination resulted, however, in a higher AL rate, deserving further scrutiny in larger trials.

### PO 98 Feasibility and safety of neoadjuvant intravenous and intraperitoneal chemotherapy (NIPS) for colorectal peritoneal metastasis #PO 98

#### Poster

Y. Gohda^1^, H. Yano^2^

^1^National Center for Global Health and Medicine - Tokyo (Japan),

^2^University Hospital Southampton - Southampton (United Kingdom)

**Abstract**

**Background:** Intraperitoneal administration of Paclitaxel (PTX) is known to enhance antitumour activity against peritoneal metastasis by maintaining a high drug concentration in the peritoneal cavity over a long period in various types of cancer. Neoadjuvant intraperitoneal and systemic chemotherapy (NIPS) may be useful to facilitate cytoreductive surgery (CRS) combined with hyperthermic intraperitoneal chemotherapy (HIPEC) for colorectal peritoneal metastasis (CPM).

**Aim:** The aim of this study was to evaluate the feasibility and safety of NIPS for CPM.

**Patients and Methods:** Between 2013 and 2016, 24 patients with colorectal or appendiceal peritoneal metastasis were enrolled in this prospective single-centre study. Patients with pseudomyxoma peritoneai were excluded from this study. Diagnostic laparoscopy was performed in all patients to a) confirm the pathology, b) establish the Peritoneal Cancer Index (PCI) score, c) predict the likelihood of complete cytoreduction and d) place an intraperitoneal port. NIPS was given according to the predetermined regimen for three to six months and a further laparoscopy was performed. The primary endpoint was feasibility and the secondary endpoints included rate of response in PCI score, rate of complete cytoreduction and overall survival.

**Result:** There were 8 appendiceal and 16 colonic primaries. Completion rate of NIPS was 92%. Severe adverse events (CTCAE v.4.0 Grade3/4) were noted in 9 (38%). Following NIPS, the median PCI score dropped from 14 (range, 3-26) to 10 (range, 1-26). The response rate in PCI score was 42% with 10 PR, 8 SD and 5 PD. CRS+HIPEC was achieved in 21 patients (88%) with 5-year overall survival rate of 29%.

**Conclusion:** NIPS could be performed safely and might be a promising treatment modality in the management of CPM with high PCI patients.

### PO 99 Robust Prognostic Signature in Colon Cancer by Studying Ectopically Expressed Genes using Machine Learning #PO 99

#### Oral communication or poster

A. Spinelli^1^, E. Bourova-Flin^2^, A.L. Vitte^2^, M.H. Laverriere^1^, J. Abba^3^, S. Rousseaux^2^, S. Khochbin^2^, S. Valmary-Degano^4^

^1^Department of Pathology, Grenoble Alpes University Hospital, F-38000, Grenoble, France - Grenoble (France),

^2^Univ. Grenoble Alpes, INSERM U1209, CNRS UMR5309, Institute for advanced Biosciences, Grenoble, France - Grenoble (France),

^3^Department of Surgery, Grenoble Alpes University Hospital, F-38000, Grenoble, France - Grenoble (France),

^4^Univ. Grenoble Alpes, INSERM U1209, CNRS UMR5309, Institute for advanced Biosciences, Department of Pathology, Grenoble Alpes University Hospital, Grenoble, F-38000, France - Grenoble (France)

**Abstract**

**Background & Objective:** Colon cancer is the third most common cancer and the second leading cause of cancer deaths worldwide. Despite effective screening programs in most countries, diagnosis comes late, when treatment options are limited or under debate. Our team has demonstrated that epigenetic deregulations in tumors lead to aberrant gene activation, which can be associated with an unfavorable prognosis. The main aim of this study is to create a panel of prognostic biomarkers in colon cancer, based on ectopic activations, to better guide the therapeutic management of patients.

**Methods:** We analyzed off-context activations of tissue-specific genes as a source of novel prognostic biomarkers in colon cancer. Using our machine learning pipeline named “ectopy” on public datasets, we identified candidate biomarkers with a stable and significant impact on survival from gene expression data. Based on these findings, we created a new Gene Expression Classifier (GEC) that stratifies patients according to the number of aberrantly activated genes, and successfully tested it in an independent public colon cancer dataset.

**Results:** By applying the “ectopy” method to three public colon cancer cohorts (n=1141), we defined a GEC prognostic signature for 4 genes: ERFE, HOXC6, LAMP5 and ULBP2. Multivariate analyses demonstrated that the GEC is a strong predictive factor of unfavorable prognosis, all stages combined, as well as in each stage of colon cancer separately, including stages IV.

**Conclusion:** The GEC signature predicts survival prognosis in colon cancer independently of other prognostic criteria. This tool will help in the management of patients, particularly in stage IV, where surgical treatment with hyperthermic chemotherapy is discussed. Prospects for the study include RT-qPCR analysis of the Grenoble Alpes University Hospital colon cancer cohort, and development of an immunohistochemical test for easy routine use.

### PO 100 CRS with HIPEC in Colorectal peritoneal metastasis: A Clinical retrospective observational study from a tertiary cancer care center #PO 100

#### Oral communication

S. Sonvane^1^, M. Office^1^

^1^Aiims New Delhi - Delhi (India)

**Abstract**

**Background:** While a multicenter RCT PRODIGE 7 showed CRS with HIPEC was associated with increased postoperative morbidity without significant survival benefit. We present a single institutional experience of 53 patients of colorectal cancer with peritoneal metastasis, who underwent CRS with HIPEC at our center, in terms of perioperative outcomes and survival benefit.

**Methods:** We did retrospective analysis of patients with colorectal peritoneal metastasis (CRC-PM) disease registered under peritoneal surface malignancy (PSM) clinic who underwent CRS with HIPEC between the period of 2014 and 2020. Patients with synchronous peritoneal disease were selected. Patients with extraperitoneal disease were excluded from the study. Selected patients were retrospectively analyzed for perioperative morbidity, mortality and survival outcomes.

**Results:**

•A total 53 patients were found to have CRC-PM and underwent CRS with HIPEC.

•10 patients (18.8 %) initially received chemotherapy prior to surgery.

•43 patients (81.1%) were operated upfront and received adjuvant chemotherapy.

•Average PCI was 7.7.

•Rate of optimal CRS = 96.2%

**HIPEC:** All patients received intraperitoneal Mitomycin.

•Temp at 42ºc

•Duration: 60 to 90 minutes

•Postoperative morbidity: 3.8%

•Mortality: None

•Median follow up: 34months

•Recurrence free survival (RFS):

•1years: 77.7%

•3yeras: 27.7%

•5years: 16.6%

**•Median OS:** 38.8months

•**pRFS:** 23months

**Conclusion:** CRC-PM disease with low PCI <10, CRS with HIPEC is a feasible procedure with acceptable perioperative outcomes, OS and pRFS. A protocol-based multidisciplinary team approach, optimal patient selection, and surgical expertise are essential for achievement of optimal outcomes.

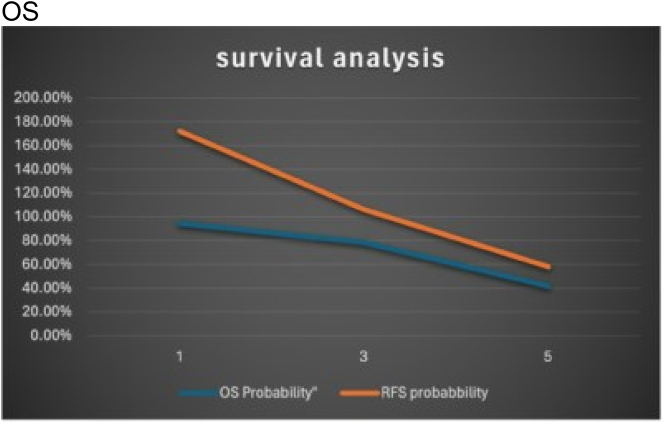



### PO 101 Effectivity of systemic CHEmotherapy on MUcnious Colon CAncer. CHEMUCCA study. (Part I: high risk stage II and stage III) #PO 101

#### Oral communication

I. Trinidad^1^, A. Arjona^1^

^1^IMIBIC - CORDOBA (Spain)

**Abstract**

Local advanced colon cancer is a high risk condition to develop tumor recurrences with poor survival. The current treatment is surgery and adjuvant chemotherapy based on fluoropyrimidines and oxaliplatin. This approach has got improvements in DFS and OS. Mucinous condition has been related to worse response to systemic chemotherapy but the evidence on this issue is weak. CHEMUCCA study arises to answer this question for mucinous colon cancer.

The objective was to compare the disease free survival for stages II and III mucinous colon cancer who receive surgery plus systemic adjuvant chemotherapy vs. surgery alone. A total of 1134 patients were collected from a retrospective cohort diagnosed with high-risk stage II (505 patients) and stage III (629 patients) colorectal cancer between the years 2010 and 2021 aged older than 18 years. Demographic variables were analyzed, included DFS (months) and OS(months). T student test and Kaplan Meier with log rank test analysis were used. Of 1134 patients with colorectal disease in stage II high risk and III, 206 (18,17%) had mucinous histology and 928 (81,83%) had non-mucinous histology. 708 patients who received adjuvant chemotherapy, 129 (62,62%) in mucinous group and 579 (62,39%) in non mucinous group. Adjuvant systemic chemotherapy in mucinous colorectal cancer stage II and III including rectum improved the DFS p=0,017. However, in a stratified analysis, patients with high risk stage mucinous colon cancer did not show any benefit with this approach (p=0.056).

Adjuvant chemotherapy seems to be useful in mucinous colorectal cancer. This benefit could be diminished in mucinous high risk stage II colon cancer patients. It is worth to evaluate this patient’s sub- group in further analysis in order to recommend adjuvant chemotherapy.

### PO 102 Risk of anastomotic leakage after cytoreductive surgery with multiple anastomoses and hyperthermic intraperitoneal chemotherapy in patients with colorectal peritoneal metastases #PO 102

#### Oral communication or poster

J. Tranberg^1^, E. Bexe Lindskog^1^, I. Syk^2^, G. Jansson Palmer^3^, P. Cashin^4^

^1^Sahlgrenska University Hospital - Gothenburg (Sweden),

^2^Skåne University Hospital - Malmö (Sweden),

^3^Karolinska University Hospital - Stockholm (Sweden),

^4^Uppsala University Hospital - Uppsala (Sweden)

**Abstract**

**Background:** While anastomotic leakage has been investigated in cytoreductive surgery (CRS) and hyperthermic intraperitoneal chemotherapy (HIPEC) in general, few studies have focused on patients with colorectal peritoneal metastases (PM) and multiple anastomoses. The aim of this study was to evaluate the risk of multiple anastomoses and prognostic value of postoperative complications.

**Methods:** Data for all patients with colorectal PM treated with CRS and HIPEC were extracted from the Swedish HIPEC registry for evaluation. Patients were categorized as having 0, 1, 2, or >2 gastrointestinal anastomoses. Clavien-dindo grade 3+ complications were evaluated as well as two specific complications: intraabdominal infection and reoperation for anastomotic leakage.

**Results:** A total of 664 patients were identified. There were 249 patients with no anastomosis, 314 with one anastomosis, 67 with two anastomoses, and 34 with more than two anastomoses (range 3-7). There was a 26% risk of Clavien-Dindo grade 3+ morbidity if no anastomosis was performed and 31% for one, 34% for 2, and 21% for >2, (p=n.s.). Similarly, 8% had an intraabdominal infection with no anastomosis, 10% for 1, 13% for 2, and 3% for >2, (p=n.s). Lastly, the reoperation rates for anastomotic leakage were 4% for 1 anastomosis, 7% for 2, 3% for >2, respectively (p=n.s.). Prognostically, patients with a Clavien-Dindo grade 3+ complication had a median OS of 29 months versus 44 months with Clavien-Dindo <3, p=0.00028. Likewise, intraabdominal infection demonstrated 30 months versus 42 months, p=0.0076. The number of anastomoses correlated to the PCI.

**Conclusions:** No increasing risk was found with multiple anastomoses. The need for multiple anastomoses should not deter the surgeon from continuing surgery and aiming for a complete cytoreduction. Postoperative complications affect oncological survival negatively but was not associated with number of anastomoses.

### PO 103 Prophylactic Hyperthermic Intraperitoneal Chemotherapy for Colon Cancer Patients at High Risk of Peritoneal Metastases: An Individual Patient Data Meta-Analysis #PO 103

#### Oral communication or poster

J. Hamm^1^, R. Van Den Berg^1^, E. Andrinopoulou^1^, E. Zwanenburg^2^, G. Musters^1^, P. Tanis^1^, A. Arjona-Sanchez^3^

^1^Erasmus University Medical Center - Rotterdam (Netherlands),

^2^Amsterdam University Medical Center - Amsterdam (Netherlands),

^3^Reina Sofia University Hospital - Cordoba (Spain)

**Abstract**

**Background:** Almost a quarter of patients with locally advanced colon cancer (pT4) develop locoregional recurrence, including peritoneal metastases, which leads to a poor prognosis. Individual randomized controlled trials have shown conflicting results of prophylactic hyperthermic intraperitoneal chemotherapy (HIPEC). This individual patient data meta-analysis (IPDMA) aimed to identify whether specific subgroups of patients might benefit from prophylactic HIPEC.

**Methods:** A systematic literature search was conducted to identify randomized controlled trials on prophylactic HIPEC in locally advanced colon carcinoma until August 1st, 2023. An IPDMA was performed with 3-year locoregional recurrence rate as primary endpoint and 3-year disease-free survival (DFS) and overall survival (OS) as secondary endpoints.

**Results:** The search revealed two randomized controlled trials (COLOPEC and HIPECT4). Individual patient data were pooled for 386 patients, of whom 189 patients received prophylactic HIPEC, and 197 patients constituted the control group. Median follow-up was 36 months (IQR 32 – 60). Modified intention to treat analysis resulted in an overall 3-year locoregional recurrence rate of 18% for HIPEC and 25% for control patients (HR 0.75, 95% CI 0.47 – 1.22). Predefined subgroup analyses revealed a significant reduction in 3-year locoregional recurrence after HIPEC in patients with right-sided tumors (HR 0.59; 95% CI 0.49 – 0.72), and patients with right-sided pT4 tumors (HR 0.47; 95% CI 0.29 – 0.77). No significant differences in DFS and OS between HIPEC and control groups were found for the overall study population.

**Conclusions:** In patients with locally advanced colon cancer, prophylactic HIPEC in addition to surgical resection and adjuvant systemic chemotherapy seems to significantly improve the locoregional recurrence rate in high-risk subgroups. This did not translate into an overall survival benefit, but follow-up is still immature.

### PO 104 A single cell atlas of colorectal peritoneal metastases #PO 104

#### Poster

J. Demuytere^1^, J. Haerinck^2^, J. De Coninck^3^, S. Ernst^1^, J. Taminau^3^, W. Ceelen^1^, G. Berx^2^

^1^Laboratory of experimental surgery, Department of human structure and repair, Ghent University - Ghent (Belgium),

^2^Molecular and Cellular Oncology Laboratory, Department of Biomedical Molecular Biology, Ghent University - Ghent (Belgium),

^3^Molecular and Cellular Oncology Laboratory, Department of Biomedical Molecular Biology, Ghent University - Ghent (Belgium) - Ghent (Belgium)

**Abstract**

**INTRODUCTION:** Notwithstanding the unmet clinical need for improved treatment, the tumor microenvironment (TME) of peritoneal metastases (PM) of colorectal cancer (CRC) has not been thoroughly characterized. Furthermore, insights into the TME composition might offer clues to the unique pathogenesis of peritoneal metastasis. Here, we detail a single-cell atlas of 10 patients with CRC PM.

**METHODS:** We prospectively collected fresh tissue samples from 10 patients with CRC PM at three distinct anatomic locations (the abdominal wall, the small bowel mesentery, and the greater omentum), along with primary tumor samples when available. Following quality control, we clustered the cells with the Seurat pipeline. We annotated all cells thoroughly and performed downstream analyses such as differential gene expression analysis and gene set enrichment.

**RESULTS:** We obtained 144,611 qualitative cells, from 29 multiplexed samples. We characterized the TME based on marker genes and signatures, across five main cellular compartments (epithelial cells, mesenchymal cells, endothelial cells, myeloid and lymphoid). The most prominent clusters encountered were T-cells, tumor-associated macrophages (TAMs) and cancer-associated fibroblasts (CAFs). Further subclustering revealed functionally diverse subclusters in both CAF and TAM clusters, demonstrating location-specific subtypes differentiating primary tumor from the metastatic TME. Analysis of the epithelial compartment showed, next to normal epithelial cells and mesothelial cells, five subclusters of cancerous cells identified by CNV analysis (with scATOMIC).

**CONCLUSIONS:** We characterized the microenvironment of colorectal PM, while demonstrating functional heterogeneity and differences between the primary and metastatic TME. Further integration of multiplex immunohistochemistry (MACSima), flow cytometry and secretomic data will complete our atlas.

### PO 105 Surgical treatment of Rectal Peritoneal Metastases in a primary tumor treated with neoadjuvant chemoradiotherapy: A Systematic Review. #PO 105

#### Oral communication or poster

R. Salcedo-Hernández^1^, L. Lino-Silva^2^

^1^National Cancer Institure of Mexico - mexico city (Mexico),

^2^Monterrey Institute of Technological Studies, Mexico City campus. - mexico city (Mexico)

**Abstract**

**Background.** Cytoreductive surgery (CRS) with or without hyperthermic intraperitoneal chemotherapy (HIPEC) is widely acknowledged as an effective treatment option for managing colorectal peritoneal metastases (CRPM). However, the incorporation of neoadjuvant chemoradiotherapy (NACRT) into this treatment approach remains a subject of debate.

**Methods.** To evaluate the impact of neoadjuvant chemotherapy on perioperative outcomes, mortality rates, and long-term survival in patients with CRPM undergoing CRS and HIPEC, a systematic review and meta-analysis were conducted.

**Results.** Fifteen studies, involving a total of 3012 patients, were analyzed. These comprised ten retrospective cohort studies, one prospective cohort study, and one prospective randomized trial. Patients who underwent NACRT followed by CRS and HIPEC experienced similar rates of perioperative complications and mortality compared to those who underwent surgery first (SF). Although there were no significant differences in overall survival at 3 years, patients who received NAC demonstrated better survival rates at 5 years (relative risk [RR] 1.25; 95% confidence interval [CI] 1.12-1.63, P < 0.001). The 1- and 3-year disease-free survival (DFS) were similar between the groups. No significant heterogenity was identified between groups.

**Conclusion.** Patients who underwent NACRT did not experience increased perioperative risks. While the potential improvement in 5-year overall survival among NACRT recipients is noted, caution is warranted due to data limitations. Nonetheless, these findings favors the use of NACRT. Until prospective, randomized evidence becomes available, the practice of incorporating NACRT in this setting will continue to vary and be influenced by retrospective data.

### PO 106 Cytoreductive surgery with hyperthermic intraperitoneal chemotherapy and liver resection is a treatment option for patients with peritoneal and liver metastases from colorectal cancer #PO 106

#### Oral communication or poster

V.J. Dagenborg^1^, K.W. Brudvik^2^, C. Lund-Andersen^3^, A. Torgunrud^3^, M. Lund-Iversen^4^, K. Flatmark^1^, S.G. Larsen^1^, S. Yaqub^2^

^1^Department of Surgical Oncology, Section of Abdominal Cancer Surgery - Oslo (Norway),

^2^Department of Hepatobiliary and Pancreatic Surgery, Oslo University Hospital - Oslo (Norway),

^3^Institute for Cancer Research, Oslo University Hospital - Oslo (Norway),

^4^Department of Pathology, Oslo University Hospital - Oslo (Norway)

**Abstract**

**Background:** Metastases from colorectal cancer (CRC) is a major cause of mortality, and are frequently found in liver (CLM) and peritoneum (PM-CRC). For selected patients with CLM or PM-CRC separately, surgery can offer long-term survival. In patients with CLM, treatment of PM-CRC with CRS-HIPEC has been a relative contraindication. Currently, small studies explore both short and long-term outcomes in patients with treated with liver resection and CRS-HIPEC, with conflicting data.

**Study design:** Patient with PM-CRC, treated at a national centre between 2007 and 2023 with CRS-HIPEC, and additional intervention for CLM, were included. The aim was to analyse complication by Clavien-Dindo, and overall and progression free survival (OS and PFS).

**Results:** We included 57 patients with advanced primary CRC (98% pT3/4, 84% lymph node metastases). Eighty-six percent had multiple sequential resections for metastatic CRC, and 86% had chemotherapy prior to CRS-HIPEC. At CRS-HIPEC median age was 59 with peritoneal cancer index (PCI) of 8. Thirteen patients had severe complications (Clavien-Dindo ≥3), but no 90-day mortality. Median OS was 48 months after CRS-HIPEC, and PFS was 6 months. PCI was a predictor of OS (Hazard ratio 1.11, P<0.001). The 57 patients were classified in three subgroups: CLM resection before CRS-HIPEC (n=11); CLM resected simultaneously with CRS-HIPEC (n=29); CLM after CRS-HIPEC (n=17), with no significant difference in short or long-term outcomes comparing subgroups.

**Discussion:** Patients treated with CRS-HIPEC and liver resection in this study, had similar OS to studies on only CRS-HIPEC, possibly due selected patients (low PCI and complexity of treatments). Simultaneous liver resection and CRS-HIPEC has similar complication rate and OS compared to other sub groups analysed.

**Conclusion:** In this national cohort, CRS-HIPEC and CLM intervention offer short and long-term survival comparable to CRS-HIPEC only, and could be offered to selected patients with PM-CRC and CLM.

### PO 107 Outcome of patients with a peritoneal surface malignancy referred to a tertiary center specialized in cytoreductive surgery and hyperthermic intraperitoneal chemotherapy #PO 107

#### Oral communication or poster

L. Van Der Snee^1^, A.G.J. Aalbers^1^, W.J. Van Eden^1^, P. Snaebjornsson^2^, R.J.A. Fijneman^2^, B.A. Grotenhuis^1^, G.L. Beets^1^, M.J. Lahaye^3^, N.F.M. Kok^1^

^1^Department of Surgical Oncology, Netherlands Cancer Institute - Amsterdam (Netherlands),

^2^Department of Pathology, Netherlands Cancer Institute - Amsterdam (Netherlands),

^3^Department of Radiology, Netherlands Cancer Institute - Amsterdam (Netherlands)

**Abstract**

**Background:** Patients with peritoneal surface malignancies (PSM) can be treated with curative intent with cytoreductive surgery and hyperthermic intraperitoneal chemotherapy (CRS-HIPEC). Patient selection remains crucial to prevent open-close procedures or overtreatment without survival benefit. It is crucial to conduct a thorough diagnostic work-up and discuss patients in a multidisciplinary team meeting in a specialized center. However, there is a lack of insight into the selection bias among patients referred for CRS-HIPEC. We aimed to report the patient flow and outcome of referrals to a tertiary CRS-HIPEC center for colorectal cancer (CRC) patients.

**Methods:** This retrospective, single center study included consecutive patients with PSM referred to a tertiary CRS-HIPEC center in 2016 and 2017. Baseline-, tumor- and treatment characteristics were collected. Date from referral to last follow-up or death was used for overall survival in peritoneal metastasized (PM) CRC patients.

**Results:** A total of 298 patients were identified, 239(80%) of whom were referred from other centers. The mean age was 63(±12) years, and 155(52%) were male. The primary tumor sites were the colorectum in 198(66%) patients, appendix in 66(23%), small bowel in 10(3%), mesothelioma in eight(3%), ovary in seven (2%), urachus in two (1%), and unknown in five (2%). From all 298 referred patients, 127(42%) underwent CRS-HIPEC. Of all 198 CRC patients, 70(35%) underwent diagnostic laparoscopy (DLS), resulting in 34 patients eligible for CRS-HIPEC. The median overall survival for PM-CRC in patients ineligible for CRS-HIPEC after DLS due to extensive disease was 12.9 months (n=29), 11.4 months for patients who had an open-close procedure (n=9) and 55.8 months for patients who underwent CRS-HIPEC successfully (n=74).

**Conclusions:** Most referred patients did not undergo CRS-HIPEC. Primary tumor origin of patients referred to our tertiary center was most frequently CRC, and among the patients who were ineligible for CRS-HIPEC, the overall survival remained poor.

### PO 108 Improving overall survival for patients with CRC-PM with dedicated care pathways; result of CRS-HIPEC, a Northwestern Netherlands, two center cohort #PO 108

#### Oral communication

L.J. Van Kesteren^1^, T.E. Buffart^2^, N.F.M. Kok^3^, J.B. Tuynman^1^, A.G.J. Aalbers^3^

^1^Amsterdam UMC location Vrije Universiteit Amsterdam, Department of Surgery, Cancer Center Amsterdam, De Boelelaan 1117, Amsterdam, The Netherlands. - Amsterdam (Netherlands),

^2^Amsterdam UMC location Vrije Universiteit Amsterdam, Department of Oncology, Cancer Center Amsterdam, De Boelelaan 1117, Amsterdam, The Netherlands. - Amsterdam (Netherlands),

^3^Department of Surgery, Antoni Van Leeuwenhoek-Netherlands Cancer Institute, PO Box 900203, 1006 BE, Amsterdam, The Netherlands. - Amsterdam (Netherlands)

**Abstract**

**Background:** Patients present with peritoneal metastasis (PM) of colorectal cancer (CRC) in 13% of the total CRC patients. Despite CRC-PM having a poor prognosis compared to patients with non-peritoneal metastasis, CRS-HIPEC can be a curative treatment for patients with limited disease. Since substantial variability in survival exist in literature this study aimed to evaluate overall survival of CRC-PM patients in dedicated collaborating centers.

**Methods:** All consecutive patients presented with CRC-PM between 2010 and 2021 eligible for CRS-HIPEC were included in this study. Analysis of OS was performed on patients after 2014 to compensate for different strategies due to different care pathways and different treatment approaches for recurrent disease. Descriptive and survival analysis was performed on these data.

**Results:** Since 2015, 348 patients received CRS-HIPEC per protocol and were included in the survival analysis.The overall median OS was 50 months (43.9-56.1 95%Confidence Interval), with a median DFS of 11 months (9.9-12.0 95%CI). The median OS was 62 months (49.1-74.6 95%CI) for the low PCI group (<8) and 23 months (43.5-56.5 95%CI) for the high PCI group (>15).

**Conclusion:** This study shows that in dedicated HIPEC centers CRS-HIPEC for patients with CRC-PM is associated with a median overall survival of 50+ months. It also shows that despite a poor DFS, the OS is substantially better compared to recent published trials and other cohorts. Multiple components of the dedicated HIPEC care pathway, including adequate patient selection, standardized CRS-HIPEC and dedicated follow-up, including therapy in case of recurrence contribute to good overall survival.

Figure 1: Survival overview

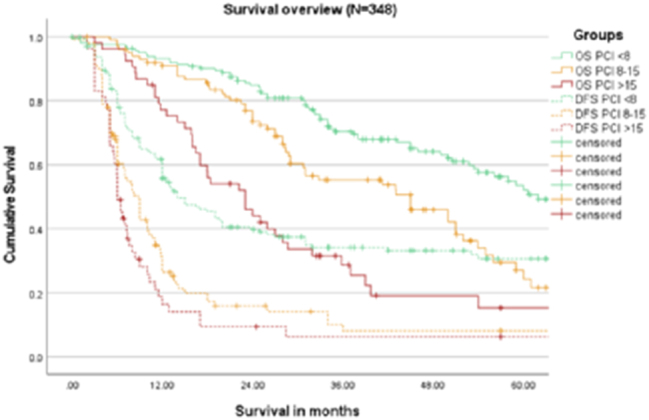



### PO 109 A single, tertiary cancer care centre experience of management of Colorectal Peritoneal Metastasis #PO 109

#### Oral communication

S.N. Vadisetti^1^, T. Vispute^1^, A. Sharma^1^, M. Kazi^1^, A. Desouza^1^, A. Saklani^1^

^1^Tata Memorial Centre - Mumbai (India)

**Abstract**

**Introduction:** Existing retrospective data suggest that untreated, CRC PM are associated with a survival of less than 9 months. In properly selected patients, CRS-HIPEC is associated with a median overall survival of 51 months.

**Aim:** To analyse the treatment and survival patterns of colorectal cancer patients who develop peritoneal disease.

**Materials and methods:** We have screened patients who registered with the Division of Colorectal surgery in the Department of Surgical oncology at Tata Memorial Hospital from 2020 to 2022. Colorectal cancer patients with or without peritoneal metastases were included. The demographic, treatment and survival details of patients with peritoneal disease were collected. All data entry and statistics were performed in SPSS software.

**Results:** 1600 patients were screened, of which 1474 patients has colorectal primary. Peritoneal disease occured in 302(20.4%) patients. Patients with baseline peritoneal disease were 43(2.9%). Among these, upfront CRS +/- HIPEC was offered to 32 patients who had an OS of 35.27 months. The patients who did not receive upfront surgery had an overall survival of 25.4 months only. In the setting of metachronous disease, 162 patients were treated with a curative intent. 67 patients who underwent CRS + /- HIPEC had an OS of 31 months(6,74). However, those patients who continued on neoadjuvant chemotherapy and did not become surgical candidates had an overall survival of only 21 months(2,68).

**Conclusion:** Our single centre experience shows that there is survival benefit when CRS +/- HIPEC is offered to patients with peritoneal metastases in both baseline and in metachronous setting.

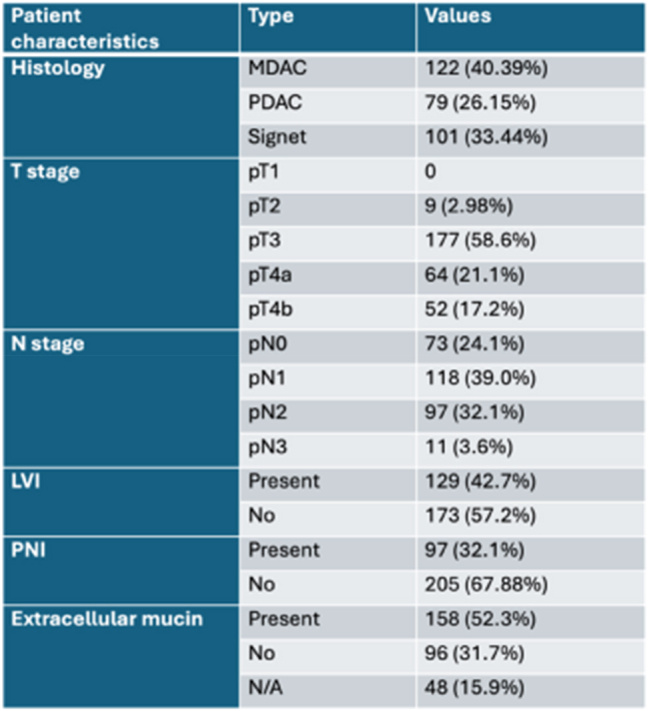



### PO 110 Appendiceal adenocarcinoma discovered during acute perforated appendicitis: a potential contribution of prophylactic CRS and HIPEC, a French BIGRENAPE group study #PO 110

#### Oral communication or poster

E. Leiritz^1^, I. Sourrouille^2^, V. Kepenekian^3^, J. Pinson^4^, A. Youcef^5^, F. Tidani^6^, E. Clément^7^, Y. Morel^8^, O. Glehen^3^, M. Pocard^1^

^1^Department of Digestive, Hepatobiliary Surgery and Liver Transplantation, Pitié-Salpétrière Hospital, Assistance Publique – Hôpitaux de Paris, Paris, France - Paris (France),

^2^Department of Visceral and Oncological Surgery, Gustave Roussy, Cancer Campus, Villejuif, France - Villejuif (France),

^3^Surgical Oncology Department, Lyon Civil Hospices, South Lyon University Hospital Center, Lyon, France - Lyon (France),

^4^Department of Digestive Surgery, Rouen University Hospital, Rouen, France - Rouen (France),

^5^Department of Digestive and Oncological Surgery, Claude Huriez University Hospital, Lille, France - Lille (France),

^6^Department of General and Digestive Surgery, Grenoble-Alpes University Hospital, Grenoble, France - Grenoble (France),

^7^epartment of Digestive Surgery and Liver Transplantation, University Hospital of Besançon, Besançon, France - Besançon (France),

^8^Department of General, Visceral and Endocrine Surgery, Pitié-Salpétrière Hospital, Assistance Publique – Hôpitaux de Paris, Paris, France - Paris (France)

**Abstract**

**Introduction:** Treatment of peritoneal metastasis from appendicular adenocarcinoma consists in cytoreductive surgery (CRS) and Hyperthermic IntraPEritoneal Chemotherapy (HIPEC). No specific recommendation exists in case of acute appendicular syndrome (AAS) with discovery of a perforated tumor. A monocentric retrospective study with few patients suggested a potential contribution of prophylactic CRS and HIPEC in that precise situation. We aim to investigate the results of that strategy, with a largest specimen of patients.

**Materials and methods:** We listed 166 patients, from 12 centers specialized in oncologic surgery, addressed for perforated appendiceal adenocarcinoma discovered during AAS, with no peritoneal metastasis at the time of diagnosis. We compare the overall-survival (OS) and the disease-free-survival (DFS) between carcinologic right colectomy and CRS with HIPEC.

**Results:** Carcinologic surgery was performed for 136 patients: 12 right colectomy, 42 prophylactic CRS and HIPEC, 76 CRS and HIPEC associated with the discovery of peritoneal metastasis during the time lapse between emergency surgery and specialized treatment, and 6 CRS performed for symptoms issues. DFS and OS are significatively better in the prophylactic CRS with HIPEC group compared to the peritoneal metastasis group (83,3% vs 47,4%, p<0,001 and 97,6% vs 64,5%, p<0,001). OS is significatively better in the prophylactic CRS with HIPEC group compared to the right colectomy group (97,6% vs 66,7%, p=0,001), whereas the difference regarding DFS is not significative (83,3% vs 66,7%, p=0,21). There is no statistical difference between the peritoneal metastasis group and the right colectomy group (DFS p=0,21, OS p=0,88).

**Conclusion:** Performing prophylactic CRS with HIPEC in case of perforated appendiceal adenocarcinoma discovered during AAS seems to be associated with a better OS compared to a carcinologic right colectomy.

We propose a prophylactic CRS with HIPEC as a standard treatment for perforated appendiceal adenocarcinoma, without peritoneal metastasis.

### PO 111 CRS-HIPEC in Colorectal Cancer: Experience from a Tertiary Cancer Care Centre in India #PO 111

#### Oral communication or poster

A. Sharma^1^, A. Mor^1^, M. K^1^, A.Z. Anwar^1^, C. Safi^1^, J. Ganesan^1^, A. Maheshwari^1^, M. Kazi^1^, A. Desouza^1^, A. Saklani^1^

^1^Tata Memorial Hospital - Mumbai (India)

**Abstract**

**Background:** Peritoneal disease can be found in upto 5% of newly diagnosed and 30% of operated patients with colorectal cancer. While there is debate regarding the role of CRS HIPEC in colorectal cancer, major consensus is that CRS-HIPEC is the standard therapy for patients with peritoneal metastasis. We present our experience of CRS-HIPEC in colorectal adenocarcinoma patients.

**Methods:** Retrospective analysis of a prospectively maintained database from March 2011 to April 2024 was done. . Demographic data, along with clinico-pathological variables were analysed. Disease burden and completeness of cyto-reduction (CC-score) were noted.

**Results:** 252 patients underwent CRS HIPEC at our centre between 2011 and 2024. 55% were male and 45% were female. Median age of presentation was 44yrs. 35% of the patients had undergone prior resection of a colorectal primary and 20 patients (9%) had undergone CRS- HIPEC before reporting to our centre. Most common site of the primary was rectum (36%) followed by right colon (29%), sigmoid colon (19%) and left colon (9%). Most common. Histology was MDAC (39%). PDAC/ signet accounted for 41%. 66% had received neo-adjuvant chemotherapy. Median PCI was 7 with CC0 and CC1 resection achieved in 83% and 6% respectively. HIPEC was given in 61% of cases. Median blood loss was 1500 ml and median hospital stay was 10 days . Grade 3 or higher complication on Clavien Dindo scale occurred in 12.6%. 30 day mortality occurred in 1.8%.Median follow up period was 15 months. Median DFS was 16 months. 2 yr OS was 89.8%. 105 patients had disease recurrence (42%) of which 35% were in the peritoneum and 17% had distant failure.

**Conclusion:** CRS HIPEC for colorectal peritoneal metastasis is safe and feasible. Majority of the patients undergo CC0/CC1 resection with acceptable long term survival outcomes.

### PO 112 It’s not just what you’ve got, it’s where you’ve got it: comparison of perioperative and oncological outcomes in CRS/HIPEC for Rectal vs. Colon cancer #PO 112

#### Oral communication or poster

D. Morezzi^1^, J.J. Tuech^2^, L. Ansaloni^3^, P. Fugazzola^3^, D. Perrina^1^, C. Vallicelli^1^, G. Vigutto^1^, A. Ghaly^3^, M. Stefano^1^, F. Catena^1^

^1^General, Emergency and Trauma Surgery Dept, Bufalini Hospital, Cesena, Italy. - Cesena (Italy),

^2^Department of Digestive Surgery, Rouen University Hospital, 1, rue de Germont, 76000 Rouen, France. - Roeun (France),

^3^Department of Surgery, Pavia University Hospital, 27100 Pavia, Italy. - Pavia (Italy)

**Abstract**

**Background:** Cytoreductive surgery (CRS) and hyperthermic intraperitoneal chemotherapy (HIPEC) are established treatments for selected patients with peritoneal metastases from colorectal cancer. However, data on the impact of the primary tumor location (colon vs. rectum) on outcomes are limited. This study aimed to investigate the influence of primary tumor location on perioperative and oncological outcomes in patients undergoing CRS/HIPEC for colorectal peritoneal metastases.

**Methods:** A retrospective analysis of a prospectively maintained database (2017-2023) from three centers was performed. Patients undergoing CRS/HIPEC (n=167) were propensity score-matched and analyzed using multivariate analysis.

**Results:** Among 167 patients, 126 had colon and 41 had rectal primary tumors. Propensity score matching ensured balanced groups with no significant differences in perioperative outcomes. A trend towards higher recurrence rate in rectal cancer was observed (p=0.22 vs. 0.40). Overall survival didn’t differ significantly. Disease-free survival showed a statistically significant trend favoring colon cancer (HR 1.57 vs. 1.80; p=0.057 vs. 0.049). Tumor location and a CC score > 0 were independent predictors of DFS (p=0.031 for both).

**Conclusions:** In this retrospective series, patients with rectal carcinoma undergoing CRS/HIPEC for peritoneal metastases exhibited a trend toward poorer DFS and higher recurrence rates compared to colon cancer patients, while OS was similar. These findings suggest a potential role for CRS/HIPEC in the management of rectal cancer peritoneal carcinomatosis. However, they also suggest that it may represent a distinct disease entity with different oncological outcomes. This hypothesis necessitates further investigation to determine the need for tailored treatment pathways, surgical procedures and perfusion strategies.

OS and DFS of rectal vs colon group

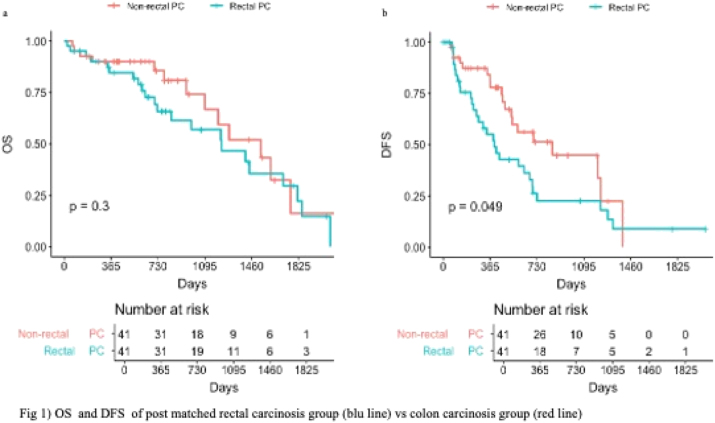



### PO 113 Early outcomes of a new colorectal peritoneal metastases service for Wales #PO 113

#### Oral communication or poster

J. Parker^1^, L. Davies^1^, J. Horwood^1^, J. Torkington^1^

^1^Cardiff and Vale University Health Board - Cardiff (United Kingdom)

**Abstract**

Five-year survival rates for colorectal cancer in Wales are some of the lowest across the continent. Until recently, access to cytoreductive surgery (CRS) and hyperthermic intraperitoneal chemotherapy (HIPEC) for peritoneal metastases was not available. A Moondance Cancer Initiative funded pilot programme has allowed the development of a Welsh service. This aimed to improve the diagnosis and assessment of colorectal peritoneal metastases through a multi-disciplinary team meeting approach and offer potential curative treatment to selected patients with CRS and HIPEC.

Prospectively maintained data regarding meetings and CRS cases was collected from May 2022. Referrals were accepted from across Wales and discussed in collaboration with the Peritoneal Malignancy Institute, Basingstoke.

Over 20 monthly meetings, 114 patients have been discussed. Referrals have been received from all health boards across the country. CRS and HIPEC has been performed in 18 patients with an average age of 65 years. Median intra-operative peritoneal carcinoma index was 8.5. Total parenteral nutrition was required for a median of 6 days and length of stay was 15 days. Six patients had complications of which three were Clavien-Dindo 2 and three were Clavien-Dindo 4. There have been no mortalities. A complete cytoreduction was achieved in all patients except one. Independent review of the service has demonstrated it is cost effective compared to our previous management approach.

This project has demonstrated a successful and safe introduction of a Welsh colorectal peritoneal metastasis service. We now aim to secure funding to commission this much needed service permanently for our Welsh patients.

### PO 114 Influence of lung metastasis on outcomes of curative management of peritoneal metastasis from colorectal cancer #PO 114

#### Oral communication

A. Pawar^1^, V. Kepenekian^2^, G. Olivier^2^

^1^The Gujarat Cancer and Research Institute - Ahmedabad (India),

^2^Lyon Sud University Hospital - Lyon (France)

**Abstract**

**Background:** The treatment of metastatic CRC have transitioned from palliative care to systemic therapy and now to surgical resection along with systemic therapy as the standard of care. Given the role of pulmonary metastectomy and CRS/HIPEC in the treatment colorectal pulmonary and peritoneal metastasis respectively, our centre decided to evaluate the role of combining these modalities in patients with CRC with peritoneal metastasis and pulmonary metastasis.

**Methods:** This was a retrospective study of prospectively maintained data base of patients of CRC peritoneal metastasis undergoing CRS and HIPEC with curative intent from 1st jan 2005 to 1st Aug 2008. Patients were divided into two groups of without pulmonary metastasis and with pulmonary metastasis. Patients were followed up for median 40.8 months.

**Results:** Of total 455 patients 19 had pulmonary metastasis. The median RFS and OS of all patients was14.26 months ( 95% CI:12.71-16.2) and 56.96 months (95% CI: 47.73-77.79) respectively. Median RFS and OS of patients with and without pulmonary metastasis was 12 & 49.8 months and 14.4 & 57.9 months respectively. On multivariate analysis, PCI, CC-0 rate, CEA, signet ring histology, retroperitoneal lymph node metastasis, N + stage after Neo-adjuvant chemotherapy and adjuvant chemotherapy significantly affected the OS. Presence of pulmonary metastasis did not significantly affect the RFS and OS.

**Conclusion:** There has always been a skepticism in the management of extra-peritoneal disease especially pulmonary metastasis with cytoreductive surgery and HIPEC in patients with colorectal peritoneal metastasis. Our study with promising results shows that this approach can be used in well selected patients and can have better survival outcomes.

### PO 115 Elevation of tumour markers is a risk factor for decreased survival and recurrence in patients with colorectal peritoneal metastastes treated by cytoreductive surgery and HIPEC #PO 115

#### Poster

N. Allievi^1^, S. Bayney^1^, M.V. Samuel^1^, A. Tzivanakis^1^, S. Dayal^1^, T. Cecil^1^, F. Mohamed^1^, B. Moran^1^

^1^Peritoneal Malignancy Institute - Basingstoke (United Kingdom)

**Abstract**

**Background:** Colorectal peritoneal metastases (CPM) pose a challenge in the management of patients with colorectal cancer. Good outcomes can be achieved with Cytoreductive surgery (CRS) and HIPEC. Aim of this study is to evaluate whether elevated tumour markers (CEA, CA19.9 and CA125) are independent risk factors for survival and recurrence in patients with CPM after complete cytoreduction.

**Material and methods:** Consecutive patients who underwent complete cytoreduction for CPM between 2000-2021 were identified from a prospectively-collected database and stratified on tumour markers status. The Kaplan-Meier method was used to evaluate overall survival (OS) and disease-free survival (DFS) and risk factors for decreased OS and DFS were studied with Cox regression.

**Results:** Overall, 376 patients (53% female, median age 57) with CPM underwent complete cytoreduction. Metastases were metachronous in 67% of cases and median PCI was 6. Elevated tumour markers were found in 220 patients (59%), with any one, two or all three markers elevated in 120 (55%), 79 (36%) and 21 (9%) patients, respectively. As compared to patients with normal tumour markers, patients with elevated tumour markers showed worse 5-year OS (62.6% vs 42.7, p<0.001) and DFS (46% vs 19.6%, p<0.001). Elevation in tumour markers was an independent risk factor for reduced OS and DFS at multivariable analysis, along with PCI>6 and pN+ at primary surgery (Table 1).

**Conclusions:** Pre-operative testing of tumour marker status, along with burden of peritoneal disease, predicts survival outcomes and provides additional benefit in decision making in patients with CPM undergoing complete cytoreduction.

**Table:** Table 1. Multivariable analysis for OS and DFS in

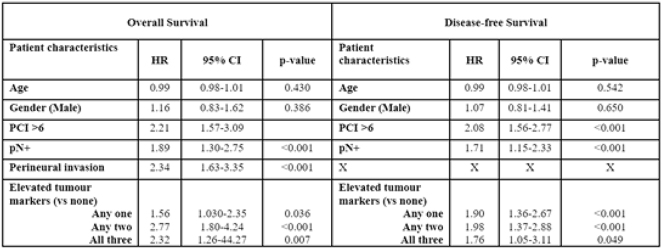



### PO 116 Postoperative leukopenia after CRS and HIPEC for carcinomatosis of colorectal adenocarcinoma – causes and implication on outcomes. A Swedish population-based study. #PO 116

#### Oral communication or poster

M. Lepsenyi^1^, V. Valdimarsson^1^, H. Thorlacius^1^, D. Asplund^2^, E. Bexe Lindskog^2^, G. Jansson Palmer^3^, P. Nilsson^3^, L. Ghanipour^4^, P. Cashin^4^, I.K. Syk^1^

^1^Lund University - Malmo (Sweden),

^2^Sahlgrenska University - Gothenburg (Sweden),

^3^Karolinska University - Stockholm (Sweden),

^4^Uppsala University - Uppsala (Sweden)

**Abstract**

**Background:** Leukocytes are reported to have a tumor stimulating effect in colorectal cancer, among others. In line with this, an earlier study (Cashin et.al, 2020) showed improved disease-free survival in patients with postoperative neutropenia compared to non-neutropenic patients after CRS+HIPEC.

This population-based study aimed to evaluate the impact on recurrence rate and survival of postoperative leukopenia after CRS+HIPEC. Further, any impact on complications and risk factors for leukopenia were evaluated.

**Methods:** All CRS+HIPEC-procedures for colorectal adenocarcinoma in the national Swedish HIPEC-registry (starting 2015) and local registries in Uppsala and Malmö (starting 2003) until December 31st 2021, were included (n=873). Cases with incomplete macroscopic cytoreduction (n=269) and cases lacking information on leukocyte count were excluded (n=20), resulting in 584 analyzed cases.

Primary outcome was overall recurrence rate. Secondary outcomes were overall survival, recurrence-free survival, and perioperative complications.

**Results:** Postoperative leukopenia (<1.5x10⁹/L) was registered in 54 (9,2%) cases, of which 31 had moderate-to-severe leukopenia (<1.0x10⁹/L). No differences in patient characteristics were noted between leukopen and non-leukopen patients.

Recurrence rate did not differ (65,4% vs 66,9%, p=0.840). Nor did overall survival or three-year recurrence-free survival. However, the subgroup with moderate-to-severe leukopenia showed a near significant worse 3-year overall survival (55.6 % vs 66,8 %, p=0.077), postoperative mortality excluded.

Risk estimates in multivariate analyses identified PCI-score (HR 1.05), severe postoperative complication (HR 1.73) and time period 2016-2018 as risk factors for 3-year mortality. Long operation time (HR 2.21) and combined oxaliplatin and irinotecan treatment (HR 10.9) were identified as risk factors for developing leukopenia. Long operation time was also a risk factor for severe postoperative complication (HR 1.80), whereas leukopenia was not.

**Conclusions:** This study showed no statistically significant impact on recurrence rate or long-term survival by leukopenia. Long operation-time and combined oxaliplatin and irinotecan treatment were risk factors for developing leukopenia.

### PO 43 Adjuvant Pressurized IntraPeritoneal Aerosol Chemotherapy (PIPAC) in resected high risk colon cancer patients - the PIPAC-OPC3 trial #PO 43

#### Oral communication or poster

M. Graversen^1^, S. Detlefsen^1^, A.P. Ainsworth^1^, P. Andersen^1^, C.W. Fristrup^1^, P.V. Pfeiffer^1^, S. Salomon^1^, L. Tarpgaard^1^, M.B. Mortensen^1^

^1^Odense PIPAC Center - Odense (Denmark)

**Abstract**

**Background:** Patients with locally advanced colon adenocarcinoma are at high risk of recurrence due to peritoneal metastasis (PM). Pressurized IntraPeritoneal Aerosol Chemotherapy with oxaliplatin (PIPAC Ox) is used in patients with non-resectable PM, but has not been evaluated in the adjuvant setting. We aimed to investigate the feasibility and efficacy of adjuvant PIPAC Ox in radically resected patients. Here we present data on feasibility and preliminary follow-up data.

**Methods:** Prospective, controlled, non-randomized, non-blinded, single center phase 2 study in 60 patients with radically resected locally advanced colon adenocarcinoma (perforated / pT4NanyM0/ pTanyNanyM1 with radically resected PM). Patients had two PIPAC Ox starting one month after resection or adjuvant chemotherapy (if indicated). Dose of oxaliplatin was 92 mg/m2, but was due to unacceptable abdominal pain lowered to 46 mg/m2 after treatment of 11 patients. Primary endpoint was three-year peritoneal recurrence free survival based on annual CT. We scored adverse reactions according to CTCAE version 4.0.

**Results:** Sixty-one patients (32 males) had 108 PIPACs from September 2017 to June 2024 at Odense PIPAC Center (OPC), Denmark. They had a performance status 0-1, 32 patients (52%) had a right sided cancer, 11 (18%) had emergency surgery, 49 patients (80%) had adjuvant chemotherapy before PIPAC. Patients were discharged within 24 hours of PIPAC in 90 procedures (83%), 47 patients (77%) completed both PIPACs. Three patients had mild surgical complications. All patients had moderate abdominal pain after PIPAC. Seven patients (11%) had severe adverse reactions (abdominal pain n=4, neuropathy n=1, allergic reaction n=1, atrial fibrillation n=1), no life threatening or fatal reactions. To date, 24 patients completed three years follow up. Two patients (3%) had recurrence due to PM.

**Conclusions:** PIPAC Ox is safe in the adjuvant setting, but the dose of oxaliplatin must be lowered to make it feasible.

### PO 118 Repeat Cytoreductive Surgery and Hyperthermic Intraperitoneal Chemotherapy (HIPEC) using Open and Closed abdomen techniques for Colorectal Peritoneal Metastases and Peritoneal Pseudomyxoma Recurrences: results from six French expert centers #PO 118

#### Oral communication

F. Tidadini^1^, C. Arvieux^1^, O. Glehen^2^, I. Sourrouille^3^, F. Marchal^4^, J.L. Quesada^1^, J. Abba^1^, B. Malgras^5^, M. Pocard^6^, A.C. Ezanno^5^

^1^Department of Digestive and Emergency Surgery, Grenoble-Alpes University Hospital, - Grenoble (France),

^2^Surgical Department, Lyon Sud University Hospital, - Lyon (France),

^3^Department of Surgical Oncology, Gustave Roussy Cancer Center, - Villejuif (France),

^4^Department of Digestive Surgery, Institut de Cancérologie de Lorraine, - Nancy (France),

^5^Department of digestive surgery, Begin Military Teaching Hospital, - Saint Mandé (France),

^6^Department of digestive surgery, La Pitié Salpêtrière Hospital, - Paris (France)

**Abstract**

**Background:** Standard treatment for peritoneal metastases (PM) combines cytoreduction surgery (CRS) with hyperthermic intraperitoneal chemotherapy (HIPEC). However, the rate of recurrence remains high and repeat CRS/HIPEC may be considered in well selected patients. We describe our postoperative and oncological outcomes.

**Methods:** Between 1994 and 2024, data from 132 repeat CRS/HIPEC were analyzed in this retrospective multi-center study. Morbi-mortality, overall survival (OS) and recurrence-free survival (RFS) were evaluated for colorectal peritoneal metastasis (CRPM) and peritoneal pseudomyxoma (PMP).

**Results:** 63 patients with PM (CRPM, n=55 (87.3%) and PMP, n=8 (12.7%)) underwent CRS/HIPEC (two, n=58 (92%), three, n=4 (6.3%) and four times, n=1 (1.6%)). PCI score, operating room occupancy, complication and readmission rates at day 90, length of intensive care and hospital stay, were similar between initial CRS/HIPEC and first repeat CRS/HIPEC. No 90 day postoperative mortality occurred. For CRPM: median OS were 82.3, 53.9 and 74.5 months from initial, first and second repeat CRS/HIPEC respectively, with median RFS of 22.0, 36.9 and 13.2 months respectively. For PMP: after median follow-up of 70.8 and 39.3 months from the initial and first repeat CRS/HIPEC, all patients are alive, with median RFS: 22.4 and 39.4 months; respectively. Multivariate analysis shown that no factor was significantly related to severe complications (Dindo-Clavien 3-4) or OS.

**Conclusions:** In selected patients with CRPM and PMP, CRS/HIPEC shows comparable results between initial and repeat procedures in terms of post-operative outcomes and appears to improve survival especially for PMP. Repeat CRS/HIPEC is an option to be considered in patients presenting CRPM or PMP.

### PO 119 Perioperative systemic therapy for resectable colorectal peritoneal metastases: A critical systematic review. #PO 119

#### Oral communication

T.B.M. Van Den Heuvel^1^, R.J. Lurvink^1^, K.P.B. Rovers^1^, I.E.G. Van Hellemond^1^, I.H.J. De Hingh^1^

^1^Catharina Hospital - Eindhoven (Netherlands)

**Abstract**

**Background:** In patients with resectable colorectal peritoneal metastases (CPM) it remains unknown whether perioperative systemic therapy in addition to CRS-HIPEC, improves overall survival (OS) and whether it should be used in a neo-adjuvant, adjuvant or perioperative setting. This systematic review summarizes the currently available evidence on this topic.

**Methods:** PubMed, Cochrane, and Embase were systematically searched to identify clinical studies assessing the association between neo-adjuvant, adjuvant, and perioperative systemic therapy and OS in patients with resectable CPM. The methodologic quality was assessed using the ROBIN’s-criteria.

**Results:** In total, eleven retrospective cohort studies describing 3423 patients who underwent CRS-HIPEC were included. Eight studies investigated OS between neoadjuvant (n=709) and no neoadjuvant systemic therapy (n=882): four reported an association between neoadjuvant systemic therapy and improved OS and four did not. Six studies investigated OS between adjuvant (n=957) and no adjuvant systemic therapy (n=707): four reported an association between adjuvant systemic therapy and improved OS and two did not. Three studies investigated OS between perioperative (n=235) and no perioperative systemic therapy (n=191): one found an association between perioperative systemic therapy and improved OS and two did not.

The ROBINS-I criteria were used to assess the risk of bias in included studies. All studies were considered to have at least moderate risk of bias due to their retrospective design.

**Conclusion:** In patients with resectable CPM, the currently available evidence on the association between neoadjuvant, adjuvant, and perioperative systemic therapy on OS solely consists of retrospective cohort studies with conflicting results and high probability of selection bias. The role of neo-adjuvant, adjuvant, or perioperative systemic therapy in patients with resectable CPM therefore remains unclear. Prospective, randomized trials are needed to investigate whether perioperative systemic therapy should be standard practice in patients with resectable CPM. This is the subject of the ongoing Dutch CAIRO6 RCT.

### PO 120 Risk Factors and Outcomes for Postoperative Evisceration within 30 Days of Cytoreductive Surgery and Hyperthermic Intraperitoneal Chemotherapy (HIPEC) using open and closed abdominal techniques for Peritoneal Carcinomatosis. #PO 120

#### Oral communication

F. Tidadini^1^, J. Fawaz^2^, J.L. Quesada^1^, J. Abba^1^, B. Malgras^3^, B. Trilling^1^, P. Sage^1^, M. Pocard^2^, C. Arvieux^1^, A.C. Ezanno^3^

^1^Department of Digestive and Emergency Surgery, Grenoble Alpes University Hospital, - Grenoble (France),

^2^Department of digestive surgery, La Pitié Salpêtrière Hospital, - Paris (France),

^3^Department of digestive surgery, Begin Military Teaching Hospital, - Saint Mandé (France)

**Abstract**

**Background:** Standard treatment for peritoneal metastasis (PM) combines Hyperthermic intraperitoneal chemotherapy (HIPEC) associated with CC0 excision. Postoperative evisceration is a rare but major complication after cytoreduction surgery (CRS) and HIPEC. This study aimed to identify the risk factors associated with evisceration after open and closed abdominal HIPEC procedures.

**Methods:** Between 2014 and 2023, medical, demographic and perioperative data of 233 patients with PM undergoing CRS/HIPEC (Open (OPEN_HIPEC), n = 110; Closed abdominal technique (CLOSED_HIPEC), n =123) were analyzed in this retrospective multi-center study. Patient factors associated with evisceration within 30 days of CRS/HIPEC were determined using univariate and multivariate Cox model analysis.

**Results:** Among 233 patients included, 129 (55.4%) were women. The median age was 60 [51; 67] years. The OPEN_HIPEC group was significantly younger than the CLOSED_HIPEC group (median 57 [47; 62] vs 63 [54; 70] years; p ≤0.001) with a higher PCI score (median 9.5 [5; 17] vs 6 [2; 11]; p≤0.001). Severe complications were similar between OPEN and CLOSED_HIPEC: 17 (15.5%) vs 15 (12.2%); p=0.471 with no mortality. Eight (3.4%) patients had postoperative evisceration with significantly more occurrences in the OPEN_HIPEC than in the CLOSED-HIPEC group (7/110 (6.4%) vs 1/123 (0.8%); p=0.028). Multivariate analysis identified a BPCO/respiratory pathology history (HR=7.39 [1.85-29.6], p=0.005) and Open-HIPEC (HR=8.37 [1.03-68.1], p=0.047) as risk factors of postoperative evisceration. Eviscerations in the OPEN group were observed following musculoaponeurotic closures using Vicryl 1 sutures. This issue was promptly identified by the team, leading to a swift switch to PDS sutures for closure.

**Conclusions:** Following CRS/HIPEC treatment, more than 3.4% patients had evisceration by day 30. A BPCO/respiratory pathology and Open-HIPEC technique were independent risk factors linked to evisceration and the need for reintervention.

### PO 121 Overall survival in colorectal peritoneal metastases after a discontinued CRS-HIPEC procedure: added value of palliative treatment. #PO 121

#### Oral communication

T.B.M. Van Den Heuvel^1^, L.J. Van Kesteren^2^, T.E. Buffart^2^, I.H.J. De Hingh^1^, J.B. Tuynman^2^

^1^Catharina Hospital - Eindhoven (Netherlands),

^2^Amsterdam University Medical Centre - Amsterdam (Netherlands)

**Abstract**

**Background:** CRS-HIPEC is performed in highly selected patients with resectable colorectal peritoneal metastases. Eligibility for this curative procedure may be limited by factors like poor overall condition or irresectability of disease due to systemic disease, extensive peritoneal disease (PCI-score >20) or excessive small intestine involvement. Despite strict patient selection, it may occur that intra-operatively complete tumor resection is not deemed feasible and the procedure is discontinued. This study aimed to evaluate the impact of a discontinued CRS-HIPEC, potentially followed by palliative systemic therapy, on overall survival (OS) and morbidity.

**Methods:** Retrospective data were collected from two tertiary hospitals in the Netherlands, focusing on patients planned for CRS-HIPEC, whose procedures were discontinued. Descriptive statistics were used to analyze clinicopathological characteristics and surgical, postoperative, and survival outcomes.

**Results:** In over ten years, 108 patients had a discontinued procedure, predominantly due to extensive peritoneal disease (56%). The majority (57%) did not undergo a diagnostic laparoscopy before the planned CRS-HIPEC. There were no significant OS differences between the aforementioned reasons for discontinuation. After an open-closed procedure, 46% of patients received palliative systemic therapy, while others received best supportive care. Severe postoperative complications (Clavien-Dindo grade ≥3) occurred in 9% of patients, with perforations and abscesses being most prevalent. Median OS for the total study population was 6.8 months (IQR 2.9 – 13.8). Median OS was significantly higher in patients with left-sided primary tumors (8.8 vs. 5.7 months) and patients receiving palliative systemic therapy (11.4 vs. 3.6 months).

**Conclusion:** Median OS of patients treated with palliative systemic therapy following a discontinued CRS-HIPEC procedure was conform literature. Nonetheless, open-closed procedures should be avoided due to the risk of surgical complications, while being a non-beneficial procedure. Preoperative diagnostic laparoscopy may decrease the risk of a discontinued CRS-HIPEC and should be considered for all patients scheduled for a CRS-HIPEC.

### PO 122 Prophylactic HIPEC in colon cancer patients with minimal serosal involvement: A pilot study #PO 122

#### Oral communication

I.S.H. Ahmed^1^

^1^nci, cairo university - cairo (Egypt)

**Abstract**

**Background:** In 10%- 35% of patients with recurrent colorectal cancer tumor recurrence is confined to the peritoneal cavity, leading ultimately to death from complications of loco regional tumoral widespread. **Aim:** To determine the oncological effectiveness of prophylactic HIPEC in preventing the development of peritoneal carcinomatosis in colorectal cancer patients having minimal serosal involvement. **Patients and methods:** This is a randomized control pilot study on an eligible group of 21 colorectal cancer patients undergoing a curative colectomy. In which prophylactic HIPEC was administered in the experimental arm, or adjuvant systemic chemotherapy alone in the standard treatment arm. HIPEC was mitomycin based; it was a 90-minute session and was applied simultaneously. The effectiveness of prophylactic HIPEC was determined by the peritoneal-recurrence free survival among both groups at 18 months. **Results:** The median peritoneal-recurrence free survival for the experimental arm was 17 months, while in the standard treatment arm it was 12 months (p-value 0.250). There were no perioperative mortalities among both groups, and only one patient in the experimental arm developed a deep surgical site infection. **Conclusion:** Prophylactic HIPEC did not seem to have a major role in the prevention of peritoneal carcinomatosis in colorectal cancer. This statement cannot be made with certainty before a full-scale randomized control trial is conducted. In addition, to the higher incidence of nodal capsular infiltration among the experimental arm; this negatively impacts survival and conceals the benefit of HIPEC among the experimental arm.

Keywords: Colorectal cancer, Minimal serosal involvement, peritoneal carcinomatosis, Prophylactic HIPEC.

### PO 123 Treatment of disseminated Appendiceal Goblet cell adenocarcinoma with Cytoreductive surgery and HIPEC. #PO 123

#### Poster

M. Pavlov^1^, S. Latincic^1^, M. Papovic^1^, M. Doskovic^1^, S. Bugarin^1^, O. Acimov^1^, J. Vasiljevic^1^

^1^University Clinical Center of Serbia - Belgrade (Serbia)

**Abstract**

**Introduction:** Goblet cell adenocarcinoma, also known as goblet cell carcinoid, is the rarest form of appendiceal tumour with an incidence of 0.05/100,000 per year in the USA. Given the rarity of the tumours, there is still no optimal strategy for their treatment. The retrospective 5-year survival rate for stage IV of the disease is 14%.

**Methods:** The treatment of 3 patients with disseminated Appendiceal Goblet cell adenocarcinoma with cytoreductive surgery and HIPEC in 2023. year at the First Surgical Clinic, University Clinical Center of Serbia.

**Results:** Three patients (men/women (1/2)) with a mean age of 50 years (46-54 years) underwent cytoreductive surgery with HIPEC (Mitomycin 12.5mgm2 and 5-Fu 650mg/m2) for disseminated goblet cell adenocarcinoma (mean PCI 21 (20-22)). Complete cytoreduction was achieved in all patients CC 0/1. The tumour marker Ca 125 was elevated in two patients with a mean value of 105 (reference range 0-35KU/L) and CEA in one patient 5.3 ( 0.0-5.0µg/L). During cytoreduction, two patients underwent total colectomy (one with ileo-rectal anastomosis, the other with end ileostomy), while in one patient a portion of the transverse colon was preserved at the middle colonic artery with ileo-colo and colo-recto TT anastomosis. All patients underwent a total omentectomy with peritonectomy of the paracolic spaces and pelvis (in both cases a hysterectomy with bilateral adnexectomy was performed during the pelvic peritonectomy). Positive lymph node metastases were present in all patients (mean 13.66 (13-14)). All patients underwent postoperative adjuvant chemotherapy with biologic therapy and are in follow-up with a mean of 9.33 months (7-13 months) with no verified recurrence of disease.

**Conclusion:** The use of cytoreductive surgery is safe and feasible in the Goblet cell patients with high PCI and disease-free in 6 months in all patients.

### PO 124 Study of the immune microenvironment in peritoneal metastases from colorectal cancer #PO 124

#### Oral communication or poster

T. Baron^1^, L. Le Bourhis^1^, T. Aparicio^1^, D. Goéré^1^

^1^Hopital Saint Louis - Paris (France)

**Abstract**

**Background:** Peritoneal metastases from colorectal cancer (PMCC) are the third most common site of recurrence with a 5-year overall survival around 30-40%. In the era of immunotherapy, there has been a growing interest in immune checkpoints that are not very efficient in CC. KLRG1 is an inhibitory molecule of the immune system that could be targeted in PMCC.

**Methods:** From January 2024 to June 2024, 14 patients with peritoneal metastases were enrolled prospectively. Both peritoneal liquid and cancerous nodes were collected directly during surgery and analyzed by flow cytometry. Spheroids were made out of nodes and assessed by confocal microscopy.

**Results:** We’ve showed a strong expression of KLRG1 on T cells in peritoneal liquid and nodules. In both ascite and nodes, KLRG1+ T cells were more present in the CD8 population than CD4. There was no significant difference between KLRG1+ CD8 T cells in nodules when compared to those in ascite. We also observed a co expression of KLRG1 and PD1 on T cells.

**Conclusions:** KLRG1 expression is increased in T cells extracted from peritoneal metastases. It might be a new promising target in patients with PMCC.

### PO 125 Cytoreductive surgery with HIPEC in colorectal cancer - results from an initial experience at a major cancer center in India #PO 125

#### Oral communication

M. Ray^1^, B. Pathak^1^

^1^All India Institute of Medical Sciences - New Delhi (India)

**Abstract**

**Introduction:** Despite its presence for three decades, the acceptance of cytoreductive surgery (CRS) with HIPEC has been slow due to the need for specialized skills, instruments, trained personnel, and the scarcity of high-level evidence coupled with high perioperative morbidity and mortality rates. However, advancements in perioperative care and literature have led to safer HIPEC procedures. Our institute performed its first CRS + HIPEC for mesothelioma in January 2015, and for colorectal carcinoma in April 2015. This study presents our initial experience with this procedure.

**Methods:** We included patients with colorectal, appendiceal, and pseudomyxoma peritonei of colorectal/appendiceal origin, treated at IRCH, AIIMS, New Delhi. Patient data were from a prospectively maintained electronic database. The semi-open technique for HIPEC was used, with chemotherapeutic agent choice based on surgeon preference and patient history. Complications were scored using Clavien-Dindo classification, disease load by PCI, and general condition by ECOG.

**Results:** Of 35 patients (65.7% females, median age 48), 85% had ECOG 1. Prior treatments included surgery (62.8%) and neoadjuvant chemotherapy (11%). Tumors were primarily in the appendix (48.57%). The median operating time was 200 minutes, and median blood loss was 300 ml. Cisplatin was the most commonly used drug. Complete cytoreduction (CC 0) was achieved in 28 cases. Postoperative complications occurred in 34% of patients, with no in-hospital or perioperative mortality. Recurrence was noted in 14.2% of patients during follow-up.

**Conclusion:** CRS + HIPEC shows promise in managing colorectal cancer with peritoneal metastases, though careful patient selection and perioperative care are crucial.

### PO 126 Seminal vesicles involvement is an unfavorable site of colorectal peritoneal metastases. A 10 year experience seminal vesicle resection as part of CRS and HIPEC #PO 126

#### Oral communication or poster

O. Albaqmi^1^, K. Chandrakumaran^2^, F. Mohamed^2^, S. Dayal^2^, A. Tzivanakis^2^, T. Cecil^2^, B. Moran^2^

^1^1- PMI Basingstoke - Basingstoke and North Hampshire Hospital Foundation Trust, Basingstoke, United Kingdom. 2- Prince Sultan Military Medical City, General Surgery Department. Riyadh, Saudi Arabia - Basingstoke (United Kingdom),

^2^PMI Basingstoke - Basingstoke and North Hampshire Hospital Foundation Trust, Basingstoke, United Kingdom - Basingstoke (United Kingdom)

**Abstract**

**Background:** Complete cytoreduction is the main predictor of outcome following cytoreductive surgery and HIPEC for colorectal peritoneal metastases (CPM). Pelvic involvement in CPM is common and in men may involve the seminal vesicles, an unfavorable site of disease. Resection of the vesicles is technically challenging and can lead to significant morbidity with limited published survival data. We report a single institution experience of seminal vesicle resection to achieve complete cytoreduction for CPM.

**Methods:** Retrospective review of a prospective database, Inclusion criteria were male patients with CPM, who had complete cytoreduction(CC:0-1), with pelvic peritonectomy and histologically confirmed resection of one or both seminal vesicles.

**Results:** Between 2013 and 2023, 2979 patients underwent CRS+HIPEC for peritoneal malignancy. Overall, 856/2979 had CPM of which 386 were male. In total, 14 patients met the inclusion criteria with a median age of 56 (IQR 45-65). The primary tumor site at index operation was right colon in 7, Left in 6 and rectum in 1. Median PCI was 9.5 (Range 3-15).CC0 was achieved in 13/14 patients and 1 had CC1. Grade 2 Clavien-Dindo complications occurred in 10 patients, with Grade 3B in 1 and Grade 4 in 2. median follow up was 12 months with a maximum of 70 months. At the time of analysis 7 patients (50%) had died, of whom 4 had documented evidence of either radiological and/or biopsy proven recurrence. Three recurrences were detected within 12 months of CRS+HIPEC. In the 7 patients still alive, 3/7 developed recurrence within 12 months of CRS+HIPEC.

**Conclusions:** Seminal vesicle resection as part of CRS+HIPEC for CPM can be performed with acceptable morbidity but may not improve survival and is likely to impair quality of life. Seminal vesicle involvement is an unfavorable site and resection should be carefully considered in highly selected cases to achieve complete cytoreduction.

### PO 127 Genetic Mutations in Colorectal Cancer Cases with Peritoneal Metastases and Liver Involvement with High Pre-cytoreductive CEA Value #PO 127

#### Oral communication or poster

A. Cristoudo^1^, S. Barat^2^, D. Morris^3^

^1^Colorectal Surgery - Sydney (Australia),

^2^Scientist Peritoneal metastases - Sydney (Australia),

^3^Professor of Surgery - Sydney (Australia)

**Abstract**

**Background:** High Carcinoma Embryonic Antigen (CEA) is known to be a precursor to Liver metastases for Colorectal Cancer Cases with Peritoneal involvement.

**Objectives:** The objective of this study was to determine the most frequently mutated genes in Colorectal Cancer cases with Peritoneal metastases and with Liver involvement.

**Methods:** Cases that were deemed suitable for Cytoreduction for Colorectal cancer with peritoneal metastases with a preoperative elevated CEA level greater than 5 microg/L and having liver involvement were identified and consented for this study. Out of the 10 cases whose tissue samples were processed at a specialized Genome research facility (Australian Genome Research Facility, AGRF) for identifying genetic mutations, 5 cases had liver metastases and were considered for further analysis.

**Results:** 5234 genes were filtered for a polyphenotypic of damaging and clinical variant marked with either potentiality or possibility of being pathogenic. Of these 2466 unique genes, identified to have muted at least once in at least one of the 5 identified cases. 601 of these genes mutated in at least 3 of the 5 cases. 64 of these genes mutated more than once in at least 3 of the 5 cases and 18 of these were Somatic variations.

In a further subset, 173 genes were mutated in all our 5 cases. With 12 genes mutated more than once in all the 5 cases. All of these were of Germline origins.

**Conclusions:** Further analysis will be needed to identify the phenotypic expressions of these mutations leading to future Diagnostic and Therapeutic advances.

ISS_PSOGI_2024_Genetic mutations in CRC with PM

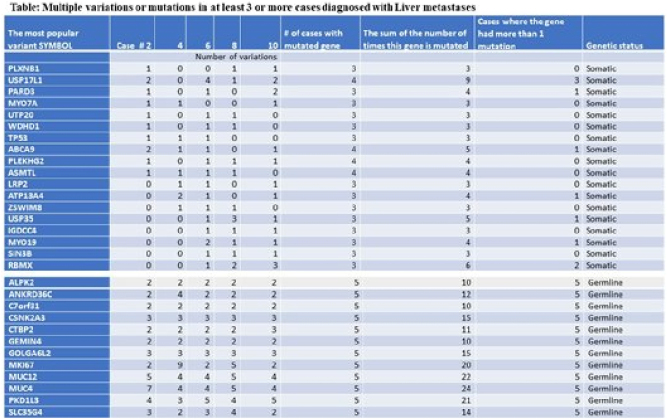



### PO 128 Cytoreductive surgery and HIPEC have a positive impact on the overall survival of patients with BRAF V600E-mutated peritoneal metastatic colorectal cancer #PO 128

#### Oral communication

S. Gül-Klein^1^, M. Atmaca^1^, A. Kunde^1^, S. Wegel^1^, M.E. Alberto Vilchez^1^, B. Rau^1^

^1^Charité - Universitätsmedizin Berlin - Berlin (Germany)

**Abstract**

**Background:** The BRAF gene mutation in colorectal cancer (CRC), with its most common mutation V600E, is a marker of poor prognosis, characterised by short survival, especially in metastasized (m)CRC. Additional surgical treatment approaches are depending on potential resectablity for mCRC. The benefit of surgery for mCRC remains unclear. This retrospective study describes the prognosis of patients with BRAF V600E-mutated CRC and mCRC and analyses the outcomes of different surgical approaches. Cytoreductive surgery (CRS) and hyperthermic intraperitoneal chemotherapy (HIPEC) are still under debate.

**Methods:** Patients with CRC registered at the Charité Comprehensive Cancer Centre were screened for BRAF mutation, and BRAF V600E mutated patients were specifically selected and analysed. Consecutively patients with mCRC, who received systemic treatment alone were distinguished from those who received additional surgery; according to primary tumour only without metastases, metastatic colorectal cancer and cytoreductive surgery combined with hyperthermic intraperitoneal chemotherapy. The primary endpoint was OS; secondary endpoints included progression-free survival (PFS), other distant metastasis-free survival (MFS) and safety.

**Results:** Between April 2012 and April 2024, 801 patients were discussed at our MDT board. 124 patients had a BRAF mutation and 112 patients had a specific BRAF V600E mutation. Only 10 patients received palliative chemotherapy; median overall survival (OS) was 7.5 months. The remaining 102 patients underwent surgery: Curative resections for primary tumour without metastases n=57, median overall survival (OS) 11 months, prog ression-free survival (PFS) 9.5 months; metastatic colorectal cancer n=27, median OS 18 months, (PFS) 9.5 months; cytoreductive surgery (CRS ) combined with hyperthermic intraperitoneal chemotherapy (HIPEC) n=18, OS 35.5 [months, (PFS) 12.5 months.

**Conclusions:** Treatment with CRS-HIPEC resulted in a comparably favourable median OS compared to patients who underwent curative surgery of the primary tumour.

### PO 129 Ovarian Involvement In Patients Undergoing Surgery For Peritoneal Metastases of Colorectal Cancer– A Peritoneal Oncology Referral Center Study #PO 129

#### Oral communication

M. Bosch Ramirez^1^, R. Narayanachary^2^, H. Fernandez^3^, D. Sabia^1^, A. Tejedor^4^, J. Tur Martinez^1^, L. Bijelic^1^

^1^Peritoneal Surface Malignancies Unit, CHU Moises Broggi - Barcelona (Spain),

^2^European Peritoneal Surface Oncology School - Barcelona (Spain),

^3^Resident, Department of Surgery, CHU Moises Broggi - Barcelona (Spain),

^4^Department of Anesthesiology, CHU Moises Broggi - Barcelona (Spain)

**Abstract**

**Background:** Ovarian involvement is considered frequent in patients undergoing cytoreduction for peritoneal metastases (PM) leading to bilateral oophorectomy in the majority. However, specific data on frequency and risk factors for ovarian metastases in colorectal cancer PM undergoing cytoreduction (CRS) is scarce.

**Methods:** We evaluated prior oophorectomy status, macroscopic appearance and microscopic involvement of ovaries in all consecutive female patients undergoing CRS in a high volume referral center from 2015 to 2020. CRS aimed at complete macroscopic disease resection with routine omentectomy and bilateral oophorectomy was the standard approach. Data was extracted from a prospectively maintained database.

**Results:** There were 141 patients with a mean age of 59.7. Of these, 28 (20%) were 50 or younger at the time of CRS. Twenty-nine had both ovaries removed prior to CRS, the vast majority (25/29, 86%) because of suspicion of malignant involvement. Eight patients (5.6%) had a prior unilateral oophorectomy while the majority (104/141, 74%) had both ovaries in place at the time of CRS. In all but 9 (6%), both ovaries were removed and histologically examined by bilateral oophorectomy either shortly before CRS or during it. A total of 62 (44%) of patients had at least one ovary involved. Normal ovaries during CRS (74/141, 52%), infrequently had occult metastases: 7/74 patients (9.4%). The overall frequency of involvement was higher among women aged 50 or less (19/28; 67%) as was the risk of occult metastases, 2/10 patients (20%).

**Conclusions:** Ovarian involvement is frequent among colorectal cancer patients undergoing CRS. The majority of involved ovaries were macroscopically abnormal while the risk of occult metastasis was low. However, the overall risk of involvement and occult involvement is higher in women younger than 50. Next steps include analyzing tumor factors predictive of ovarian involvement as well as the correlation of ovarian involvement and other peritoneal sites.

### PO 130 Impact of timing of cytoreductive surgery and HIPEC in prophylactic treatment for colorectal cancer at risk of peritoneal metastasis: report of a monocentric experience. #PO 130

#### Oral communication or poster

M. Tomassi^1^, A. Kefleyesus^2^, L. Villeneuve^1^, I. Bonnefoy^1^, N. Benzerdjeb^1^, O. Glehen^1^, V. Kepenekian^1^

^1^Department of General Surgery and Surgical Oncology, Centre Hospitalier Lyon-Sud, Hospices Civils de Lyon - Lyon (France),

^2^Department of Visceral Surgery, Lausanne University Hospital, - Lausanne (Switzerland)

**Abstract**

**Background:** Up to 5% of colorectal cancer (CRC) develops peritoneal metastasis (PM) after primary tumor resection (PTR). Adjuvant chemotherapy (CT) improved outcomes, without eliminating this risk of PM. Cytoreductive surgery (CRS) and hyperthermic intraperitoneal chemotherapy (HIPEC) combined with systemic CT are proposed with different timing regarding the PTR (synchronously or as second look). Efficacy to improve the locoregional control when used synchronously have been demonstrated.The aim of that study was to analyze the impact of CRS-HIPEC according to the different timings of administration.

**Methods:** This was a retrospective analysis including CRC patients at risk of PM or with confirmed PM, treated with CRS-HIPEC, in 2010-2021.We distinguished upfront, interval and second look prophylactic CRS-HIPEC subgroups. Patients treated for metachronous PM were the control group.Survival endpoints were:the time between the primary tumor diagnosis date and follow-up until death from any cause(OS),and disease recurrence(RFS).Propensity score adjusted for risk factors for early peritoneal progression was used.

**Results:** Prophylactic treatment was performed in 127 patients:16%has upfront CRS-HIPEC,61% interval and 23% closing CRS-HIPEC.32% of patients had PM diagnosed at prophylactic CRS-HIPEC.Matched analysis showed significative difference in OS and RFS for prophylactic treatment 101.6 months vs 59.9(p=0.012) and 81.9 vs 31.9 months(p=0.019), respectively.For upfront, interval and closure timing OS rates were significantly improved in the upfront setting.3-y OS rate was of 100% for the upfront group, 82.6% for the interval group and 83.8% for the closure timing.

**Conclusion:** Prophylactic strategies could improve the outcome of CRC at high risk of PM.The upfront subgroup seemed to offer the better long-term outcomes.

OS upfront, interval,closure prophylacticCRS-HIPEC

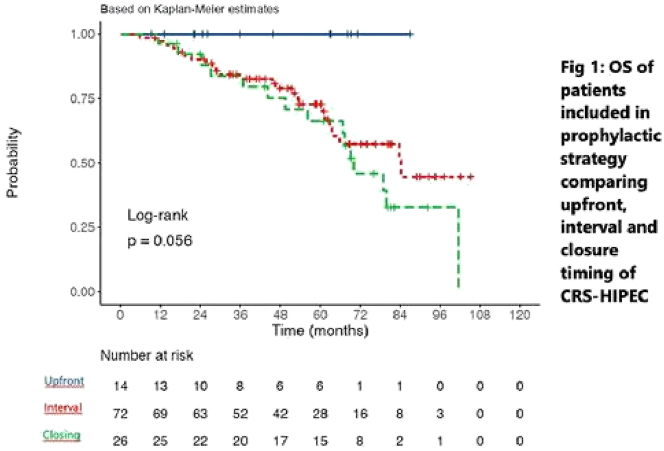



### PO 178 Synergestic effect of Oxaliplatin, ATR inhibitor and anti-PD1 combination, leads to colon cancer carcinogenesis eradication though deregulation of neutrophil homeostasis and increased PD1+ CD8+ Tcells population in mice. #PO 178

#### Oral communication or poster

A. Fauvre^1^, C. Ursino^1^, V. Garambois^1^, N. Vezzio-Vié^1^, L. Jeanson^1^, L. Milazzo^1^, E. Culerier^1^, O. Sgarbura^1^, J. Faget^1^, C. Gongora^1^

^1^Institut de recherche en cancérologie - Montpellier (France)

**Abstract**

**Background:** Colorectal cancer is the third most common type of cancer and one of the leading cause of cancer-related deaths worldwide. The treatment of advanced metastatic forms, including peritoneal metastases, is limited by the emergence of resistance mechanisms, including to oxaliplatin. In this context, we have recently shown that the combination of oxaliplatin and ATR inhibition is synergistic and may have a potential therapeutic effect in the treatment of peritoneal metastases from colorectal cancer.

**Methods:** We now studied the role of this combination (called VOX for VE-821 + Oxaliplatin) + anti-PD1 on the immune response on peritoneal carcinomatosis mouse models. Read outs are PCI scores and immunophenotyping of immune cells infiltrated in tumors, blodd, spleen and bone marrow.

**Results:** We have shown that VOX + anti-PD1 are reducing the tumor growth until no tumor is visible and protect 100% of cured animals from a rechallenge. VOX treatment is associated with a reduction of tumor-infiltrated neutrophils and CD206+ macrophages while inducing CD8+ T cell accumulation in the tumor. Indeed, we observed that Vox induced a deep depletion of both tumor and peripheric neutrophil which associate with an increased bone marrow neutropoiesis that failed compensating VOX mediated differentiated neutrophil depletion.

Moreover, Vox treatment led to increased PD1 expression on CD8+ T cells present in blood and spleen. This observation might further explain how combining VOX and anti-PD1 displays major anti-tumor effect. These PD1+ CD8+ T cells expressed EOMES and proliferate in spleen. They are containing a strong proportion of Ly6C+ CD62L+ cells (effector memory phenotype) which might allow long-term protection against tumor rechallenge.

**Conclusion:** VOX+anti-PD1 is able to eradicate peritoneal metastases and protect mice from rechallenge. This synergistic effect is accompanied with increase of new type of cytotoxic CD8 T cells, that may be responsible for the anti-tumor effect and long-term protection.

## Peritoneal metastases from gastric cancer

### PO 132 The Efficacy of Pressurised Intraperitoneal Aerosol Chemotherapy combined with Curative Intent Minimally Invasive Radical Resection in High-risk Gastric Cancer Patients – EPICURE: A multicentre, randomized study. #PO 132

#### Oral communication

J. Sanberg^1^, I. Rouvelas^2^, M. Graversen^1^, A.P. Ainsworth^1^, S. Detlefsen^3^, M. Nilsson^2^, P. Pfeiffer^4^, L. Tarpgaard^4^, A. Tsekrekos^2^, M.B. Mortensen^1^

^1^Odense PIPAC Center, Odense University Hospital. Department of Surgery, Odense University Hospital. Department of Clinical Research, Faculty of Health Sciences, University of Southern Denmark. - Odense (Denmark),

^2^Department of Upper Abdominal Diseases, Karolinska University Hospital and Division of Surgery and Oncology, CLINTEC, Karolinska Institutet. - Stockholm (Sweden),

^3^Odense PIPAC Center, Odense University Hospital, Odense, Denmark. Department of Clinical Research, Faculty of Health Sciences, University of Southern Denmark, Odense, Denmark. Department of Pathology, Odense University Hospital, Odense, Denmark. - Odense (Denmark),

^4^Odense PIPAC Center, Odense University Hospital. Department of Clinical Research, Faculty of Health Sciences, University of Southern Denmark. Department of Oncology, Odense University Hospital. - Odense (Denmark)

**Abstract**

**Background:** Standard treatment of gastric adenocarcinoma (GAC) with curative intent includes gastrectomy and perioperative chemotherapy. Nevertheless, the prognosis is poor, with frequent relapses in the peritoneum. We have recently shown in a pilot study that the combination of minimally invasive gastrectomy and synchronous PIPAC with cisplatin and doxorubicin (C/D) in resectable GAC is feasible and safe.

This planned, prospective, multicentre, randomized open-label phase-II study investigates whether PIPAC C/D delivered immediately after minimally invasive gastrectomy and repeated 6-8 weeks later results in a reduced incidence of peritoneal recurrences in patients with high-risk GAC.

**Methods:** A total of 264 patients with high-risk localized GAC (cT3+ or cT2+ if poorly cohesive histological type) deemed eligible for curative resection are randomized between minimally invasive gastrectomy (laparoscopic or robot-assisted) and perioperative chemotherapy (control arm) or minimally invasive gastrectomy and perioperative chemotherapy combined with PIPAC C/D (experimental arm).

A diagnostic laparoscopy is carried out 12 months after randomization unless a scheduled (PET) CT shows recurrence. The primary outcome is peritoneal disease-free survival (DFS) at 12 months. Secondary outcomes include overall DFS, overall survival, rate of positive peritoneal lavage cytology, patient-reported quality of life, postoperative complications, toxicity, and mortality.

Initially, patients will be recruited from Denmark and Sweden, followed by the inclusion of PIPAC centres in USA, Germany, and France. Further expansion may involve additional international high-volume, specialized centres. The study is registered with ClinicalTrials.gov (NCT06295094).

**Conclusion:** This study will investigate whether adjuvant PIPAC may reduce the rate of peritoneal recurrences in patients with high-risk GAC.

### PO 133 Patterns of aggravation in gastric cancer patients with peritoneal metastases who underwent paclitaxel based intraperitoneal therapy #PO 133

#### Oral communication or poster

Y. Tsuji^1^, K. Teramura^2^, A. Doi^1^, K. Shuji^2^

^1^Tonan Hospital Department of Medical Oncology - Sapporo (Japan),

^2^Tonan Hospital Department of Surgery - Sapporo (Japan)

**Abstract**

**Background:** We have showed promising results of intraperitoneal (ip) paclitaxel (PTX) in combination with systemic chemotherapy for gastric cancer (GC) with peritoneal metastases (PM) at the PSOGI in 2023 (P332). ipPTX could provide significantly longer survival for GC pts with PM than conventional chemotherapy, and there were even several patients with the potential to be cured eventually. We analyzed patterns of aggravation and death in pts who underwent ipPTX to achieve better understanding of the disease and its potential outcomes.

**Subjects:** We excluded cases with metastases other than peritoneal. There were GC 56 pts with PM only that received ipPTX regimens as first line chemotherapy.

**Results:** 36 pts (64%) out of 56 achieved conversion surgery, their PFS and MST were 19.1 and 30.3 mos., respectively. Out of the 36 pts, 22 (61%) recurred, and 14 (39%) died. The most frequent site of aggravation was the peritoneum: 16 pts (73%), followed by rectum (stenosis): 3 pts (14%). 2 pts died of meningitis carcinomatosa.

**Conclusion:** ipPTX in combination with systemic chemotherapy provided significantly longer survival than conventional chemotherapy for GC with PM. However, the majority of patients, including the conversion surgery cases, recurred and eventually died due to reaggravated peritoneal metastases. There were mainly two patterns of aggravation, one was resistance to ipPTX which caused multiple intestinal stenosis or obstruction, the other was limited distribution of ipPTX which in time allows for development of extra peritoneal metastases, causing mostly rectal stenosis and meningitis.

### PO 134 Intraperitoneal chemotherapy combined with Cell-free and concentrated Ascites Reinfusion Therapy (CART) for patients with massive malignant ascites due to peritoneal metastasis of gastric cancer #PO 134

#### Oral communication

R. Kanamaru^1^, H. Hideyo^1^, K. Kazuya^1^, K. Kentaro^1^, S. Shin^1^, H. Hideyuki^1^, H. Hironori^1^, Y. Yoshinori^1^, N. Naohiro^1^, J. Joji^1^

^1^Jichi Medical University - Shimotsuke (Japan)

**Abstract**

**BACKGROUND:** Cell-free and Concentrated Ascites Reinfusion Therapy (CART) is a form of apheresis therapy that involves intravenously reinfusing filtered and concentrated ascites, which contains albumin and globulin, to patients. We retrospectively investigated the outcome of gastric cancer patients with peritoneal metastasis (PM) who had massive ascites and required paracentesis for palliation. **METHODS:** We have experienced 12 patients (2016-2023). At the beginning of the treatment, paracentesis was performed using a percutaneous IP catheter to remove the ascites. In some patients, the ascites was filtered and concentrated with the CART system and reinfused through a peripheral vein. Subsequently, paclitaxel (PTX) was delivered through the catheter. As the ascites volume decreased, PTX was administered at a dose of 40 mg/m^2^ through an IP access port implanted in the subcutaneous space in conjunction with the triweekly systemic SOX regimen (100 mg/m^2^ Oxaliplatin and 80 mg/m^2^/day oral S-1). **RESULTS:** In 12 patients, average volume of ascites obtained from 12 patients was 3.6 (2.5-5.4) L. In 7/12 patients, CART procedures were performed. In those patients, average of 3.1 (2.5-4.3) L of ascites was concentrated to an average of 560 mL (2.5-1150 mL) containing 19.3g/dl (8.0-27.0g/dL) total protein, which were reinfused to the patients. An IP port was established in all patients, and IP-PTX was administered for 1-27 courses, with a median of 9 courses. The median survival time (MST) for the 7 patients who underwent IP chemotherapy in conjunction with the CART procedure was 12.4 months, with a 50% one-year survival rate. **CONCLUSIONS:** The combination of IP chemotherapy with PTX and CART has demonstrated high effectiveness and can be recommended as an initial treatment option for patients with symptomatic massive malignant ascites originating from PM of gastric cancer.

### PO 135 Confirmation of Radiological Peritoneal Carcinomatosis Index Score with Diagnostic Laparoscopy in Gastric Peritoneal Carcinomatosis #PO 135

#### Oral communication

P.O. Özcan^1^, O. Duzgun^2^

^1^Istanbul University Cerrahpasa Medical Faculty - istanbul (Turkey),

^2^Umraniye Research and Trainig Hospital - istanbul (Turkey)

**Abstract**

**Background:** In gastric cancer (GC) with peritoneal metastasis (PM), deciding on surgery based on the radiological Peritoneal Carcinomatosis Index (PCI) score remains a serious challenge. Patients with a PCI score of 7 or lower benefit from cytoreductive surgery and hyperthermic intraperitoneal chemotherapy (CRS+HIPEC). To avoid unnecessary surgery in these cases, it is necessary to perform PCI scoring with diagnostic laparoscopy (DL). In this study, we aimed to perform DL PCI scoring for cases with a radiological PCI score of 7 and below, and to compare both groups.

**Methods:** Data of patients diagnosed with GC with PM in our clinic and underwent staging laparoscopy (SL) due to a radiological PCI score of 7 or below, between May 2016 and March 2024, were retrospectively analyzed. Patients’ demographic data, PCI, and CC scores were collected from the clinical information system. Abdominal PCI staging was performed using MRI. Patients requiring emergency and palliative surgery, those with distant metastasis, and those with a radiological PCI score of 8 and above were excluded from the study.

**Results:** A total of 81 patients diagnosed with gastric peritoneal carcinomatosis (GPC) and with a radiological PCI score of 7 or below underwent DL. In 32 cases (39.5%), the PCI score was 8 or higher during laparoscopy, and the procedure was terminated without surgery. Forty-nine cases (61.5%) had a PCI score of 7 or below (range 3-7), and these patients underwent combined CRS+HIPEC with 60 minutes of cisplatin and doxorubicin. Among these 49 patients, 45 (55.5%) were male and 36 (44.4%) were female, with a mean age of 55 years (range 22-70). CC 0-1 score was achieved in all 49 cases.

**Conclusions:** SL is a more effective than radiological PCI scoring in detecting GPM cases with low PCI scores and in reducing the rate of unnecessary laparotomy with high accuracy.

### PO 136 Intraperitoneal paclitaxel combined with systemic chemotherapy for gastric cancer with peritoneal metastasis: Experience of 100 cases in a general hospital in Japan. #PO 136

#### Poster

T. Konishi^1^, H. Ishigami^2^, M. Asakage^1^, J. Kishikawa^1^, T. Inaba^1^, K. Kubota^1^

^1^Department of Surgery,Tohto Bunkyo Hospital - Bunkyo-ku (Japan),

^2^Depertment of Chemotherapy,Depertment of Gastrointestinal Surgery,The University of Tokyo - Bunkyo-ku (Japan)

**Abstract**

**Background:** Various types of intraperitoneal (IP) treatments have been developed to treat gastric cancer patients with peritoneal metastasis. IP paclitaxel (PTX) via a port combined with systemic chemotherapy has advantages in terms of sustained high IP concentration, frequency, and duration of the treatment. The efficacy and safety of IP PTX have been suggested by clinical trials performed mainly in Asia. In addition, the efficacy of gastrectomy after response to combination chemotherapy has been reported.

**Methods:** We retrospectively evaluated the safety and efficacy of combined IP and systemic chemotherapy in primary gastric cancer patients with peritoneal metastases treated at Tohto Bunkyo Hospital in collaboration with the University of Tokyo Hospital between 2014 and 2023.

**Results:** We treated 100 gastric cancer patients with IP chemotherapy. The median age was 56 years (range 18–85). The median peritoneal cancer index (PCI) was 13 (IQR 5–20). Forty-one patients had already received standard systemic chemotherapy at their previous hospitals, and five patients had undergone palliative gastrectomy before starting chemotherapy. The median time from initial diagnosis to initiation of IP chemotherapy was 1.9 months (range 0–15.0). The median overall survival (OS) was 19.9 months (95%CI 14.7–27.5), and the 3-year OS rate was 33.9% (95%CI 24.8%–44.3%) from the start of IP chemotherapy. We performed gastrectomy in 47 patients after response to IP chemotherapy, and the median OS was 41.7 months (95%CI 27.5–56.3). The 5-year OS rate was 31.9% (95%CI 18.8%–48.7%). The most common adverse events of chemotherapy were neutropenia and leukopenia, and IP port infections and catheter occlusions were observed as reported in clinical trials. There were no treatment-related deaths.

**Conclusions:** IP PTX in combination with systemic chemotherapy is safe and effective for gastric cancer with peritoneal metastasis.

### PO 137 Peritoneal pathologic response in patients with gastric cancer and carcinomatosis managed with chemotherapy with curative intent #PO 137

#### Oral communication or poster

S. Guerrero-Macías^1^, M.E. Manrique-Acevedo^1^, C. Bonilla^1^, M. Vargas^1^, P. Jimenez^1^

^1^Cancer Treatment and Research Center (CTIC) - Bogotá (Colombia)

**Abstract**

**Background:** The Peritoneal Regression Grade Score (PRGS) is a scoring system designed to objectively measure the extent of residual disease following systemic or intraperitoneal therapies. Higher grades (3-4) correspond to a poorer treatment response and prognosis, often leading to earlier recurrence. Conversely, lower grades (1-2) responses are linked to significantly longer overall and progression-free survival periods. This score is determined through histopathological examination of peritoneal biopsies taken laparoscopically before and after chemotherapy. Assessing this response and its potential correlation with the prognosis of patients with peritoneal carcinomatosis resulting from gastric cancer could prove valuable in determining the most appropriate therapeutic approach.

**Methods:** Prospective cohort study including patients with gastric cancer and peritoneal carcinomatosis with a Peritoneal Cancer Index (PCI) of less than 10, who initiated chemotherapy with the intent of undergoing subsequent cytoreductive surgery. Intervention: Precision laparoscopy after chemotherapy, performed 2-3 weeks after completion of the last cycle of the chemotherapy protocol.

Peritoneal cytology: At least 2 different quadrants, with or without prior irrigation with saline in the absence of free fluid.

Peritoneal biopsies: Two or three biopsies from different areas, taken with non-energy cutting instruments to avoid sample loss.

**Results:** This study is approved by ethics committee of the Luis Carlos Sarmiento Angulo Cancer Treatment and Research Center (CTIC) in Bogotá, Colombia and is in the recruitment phase.

**Conclusions:** This research aims to expand the application of PRGS to the curative scenario and to describe additional factors such as tumour biology and chemotherapy protocols as associated factors that could aid in identifying suitable candidates for adjuvant HIPEC among patients with gastric cancer and peritoneal carcinomatosis.

### PO 138 Molecular profiling of peritoneal metastatic gastric cancer #PO 138

#### Poster

J. Lee^1^, C. Charton^1^, S.H. Kang^1^, S.S. Kim^1^, Y.S. Park^1^, S.H. Ahn^1^, Y.S. Suh^1^, N.J. Kwon^2^, J. Kim^1^, H.H. Kim^3^

^1^Seoul National University Bundang Hospital - Seongnam-si (Korea, Republic of),

^2^Macrogen Inc. - Seoul (Korea, Republic of),

^3^Chung-Ang University Gwang Myeong Hospital - Gwangmyeong-si (Korea, Republic of)

**Abstract**

**Background:** Early diagnosis and appropriate treatment significantly reduce the mortality rate of gastric cancer, but metastatic gastric cancer has a remarkably low survival rate. Gastric cancer peritoneal metastasis (GCPM) is a distinct clinical entity with a poor prognosis, characterized by unique and aggressive features. Understanding the molecular biology of GCPM is a critical unmet need for effective treatment strategies.

**Methods:** We conducted comprehensive whole genome and RNA sequencing analyses on GCPM samples and paired primary gastric cancer (GC) tumor tissues from 14 and 26 patients for whole genome and RNA sequencing, respectively.

**Results:** Our findings revealed substantial variability in the mutational overlap between GCPM samples and their paired primary GC tissues, with some mutational signatures being unique to either the GCPM or the primary GC samples. Accordingly, tumor evolution analysis indicated the presence of distinct clones specific to GCPM or the primary tumors in most patients and might reveal a divergent evolution in primary tumor and / or GCPM after seeding. Notably, Molecular Functional Portraits (MFP) analysis of the tumor micro-environment showed no correlation between the primary tumor and the peritoneal seeding micro-environments. Surprisingly, desert and fibrotic micro-environments were predominant in primary tumor, while immune-enriched and immune-enriched fibrotic environments accounted for approximately half of the metastasis’s samples.

**Conclusions:** Our study uncovers significant intra-patient heterogeneity between GCPM and primary tumor tissues both at the molecular and the tumor micro-environment levels, underscoring the need for novel, molecular-guided therapies to effectively target GCPM.

Corresponding Author: Jinho Kim (jinho.kim@snubh.org) and Hyung-Ho KIM (lapakh2@gmail.com)

Molecular Functional Portrait (MFP) in GC and PM

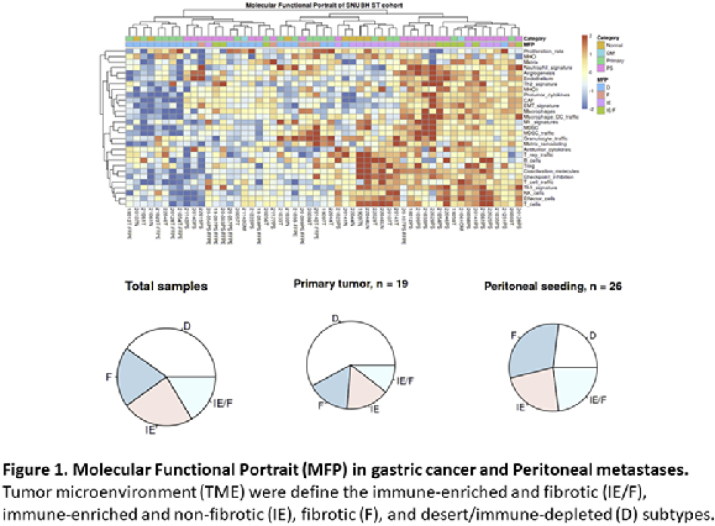



### PO 139 Acute immune activation, anti-tumor activity and survival benefit of intraperitoneal chimeric oncolytic virus, CF33-hNIS alone and combined with systemic anti-PD-L1 treatment in an immunocompetent mouse model of gastric cancer peritoneal metastasis #PO 139

#### Oral communication or poster

A. Yang^1^, Z. Zhang^1^, A. Park^1^, S. Chaurasiya^1^, S.I. Kim^1^, J. Lu^1^, H. Valencia^1^, C. Chen^1^, Y. Fong^1^, Y. Woo^1^

^1^City of Hope - Duarte (United States)

**Abstract**

**INTRODUCTION:** Peritoneal metastasis (PM) from gastric adenocarcinoma (GC) is fatal. We are developing a peritoneal-directed oncolytic viral strategy against GCPM, using CF33-hNIS, a novel orthopoxviral chimera.

**METHODS:** We treated five human (AGS, MKN-74, KATO-III, MKN-45, and SNU-16) and 2 murine (ACKPY3944 and ACKPY4113) GC cell lines at various MOIs of CF33-hNIS. We assessed viral replication, cytotoxicity and cell surface and intracellular PD-L1expression. ACKPY 3944 cells from transgenic mice (KRAS G12D mutant/CDH-1 attenuated) were transfected with ffluc to establish a PM model in C57BL/6 mice. Mice were divided into 4 cohorts: 1) IP PBS, 2) IV anti-PD-L1, 3) IP CF33-hNIS, 4) IP CF33-hNIS plus IV anti-PDL-1. Mice received IP CF33-hNIS at 3x107 PFU or PBS on days 3, 5, and 7. Starting on day 8, cohorts 2 and 4 were treated with three weekly doses of IV anti-PD-L1 antibody (200 µg/mouse). Peritoneal fluid/washings were collected on days 5, 9, and 21 and analyzed by flow cytometry for immune cell changes. Weekly bioluminescence imaging monitored peritoneal tumor burden.

**RESULTS:** CF33-hNIS infected, replicated in, and killed human and mouse GC cells in a time and dose-dependent manner. Greater antitumor efficacy was seen against human versus mouse GC cells. We confirmed endogenous PD-L1 expression in ACKPY3944 cells. Compared to PBS (average survival 18.7 days), IP CF33-hNIS improved survival to >50 days). IP CF33-hNIS combined with IV mouse anti-PD-L1 antibody also improved survival (>50.5 days with 4/8 mice alive). No significant difference was seen with addition of IV anti-PD-L1 to IP CF33-hNIS and between IV anti-PD-L1 compared to PBS. Also, IP CF33-hNIS transiently but significantly increased total CD45+ leukocytes, CD3+ and CD8+ T cells, and F4/80+ cells in the peritoneal cavity on day 9, but not on day 21.

**CONCLUSION:** CF33-hNIS exhibits robust anti-tumor activity against GCPM with transient PD-L1 upregulation and immune activation.

### PO 140 HIPEC for gastric cancer: A multicenter retrospective Canadian study #PO 140

#### Oral communication or poster

F. Bénard^1^, S. Marcil^2^, L. Mack^3^, A. Bouchard-Fortier^3^, F. Mercier^1^, E. Haase^4^, C. Boulanger-Gobeil^5^, G. Leblanc^2^, L. Sidéris^2^, M.K. Gervais^2^

^1^Centre Hospitalier de l’Université de Montréal (CHUM) - Montreal (Canada),

^2^Hôpital Maisonneuve-Rosemont - Montreal (Canada),

^3^University of Calgary - Calgary (Canada),

^4^University of Alberta - Edmonton (Canada),

^5^Hôtel-Dieu de Québec - Quebec (Canada)

**Abstract**

**Background:** Metastatic gastric cancer is associated with a poor prognosis, with a median survival estimated to less than 12 months. Palliative systemic therapies remain the standard of care for metastatic gastric cancer. Peritoneal carcinomatosis representing the most common site of recurrence, there is growing interest in cytoreductive surgery and hyperthermic intraperitoneal chemotherapy (CRS-HIPEC) for gastric cancer, with some encouraging results from Asian populations. However, Western studies remain scarce. This Canadian study reports oncologic outcomes and details the clinico-pathologic characteristics of patients who underwent CRS-HIPEC for gastric cancer.

**Methods:** This retrospective study included patients 18 years or older with a diagnosis of gastric adenocarcinoma associated with isolated peritoneal disease, who were treated with CRS-HIPEC in five academic centers in Canada between 2016 and 2022. Patients’ characteristics, previous treatments, operative data, and oncologic outcomes, including survival and recurrence, were collected and analyzed.

**Results:** Twenty patients, with a median age of 60 years old, underwent CRS-HIPEC for gastric cancer. Most presented with diffuse type (70%), poorly differentiated (90%) adenocarcinoma, and had synchronous peritoneal disease (95%). All but one patient (95%) received neoadjuvant chemotherapy. At the time of CRS-HIPEC, median peritoneal carcinomatosis index was 3 (range 0-13). Complete cytoreduction (CC0) was achieved for all patients. Various HIPEC agents were used, Mitomycin C being the most common (60%). Throughout the mean follow-up period of 16 months (range 2-44), 7 patients (35%) did not show any sign of recurrence. Median time to recurrence was 12 months, with an estimated median overall survival of 27 months. The 90-day morbidity rate in our cohort was 10%, with a 90-day mortality of 0%.

**Conclusions:** CRS-HIPEC was able to prolong survival in well-selected gastric cancer patients with limited peritoneal carcinomatosis or positive peritoneal cytology only. Future trials will further highlight the potential benefits of this treatment compared to traditional approaches.

### PO 141 The role of primary tumor resection in stage IV gastric cancer patients #PO 141

#### Poster

M. Welten^1^, F. Berben^1^, I. De Hingh^2^, I. Van Hellemond^1^, G.J. Creemers^1^, F. Van Erning^3^, R. Verhoeven^3^, H. Van Laarhoven^4^, M. Luyer^2^, G. Simkens^2^

^1^Oncology - Eindhoven (Netherlands),

^2^Surgery - Eindhoven (Netherlands),

^3^Research & Development - Eindhoven (Netherlands),

^4^Oncology - Amsterdam (Netherlands)

**Abstract**

**Introduction:** The role of primary tumor resection (PTR) for stage IV gastric cancer patients is still under debate. Hence, PTR is still performed in certain cases with unclear clinical benefit.

**Objectives:** The aim of this study is to provide insight in the patient population undergoing PTR and to identify specific patient and tumor characteristics in which PTR may be of added value.

**Methods:** All Dutch adult patients who received any tumor directed treatment for stage IV gastric adenocarcinoma between 2010 and 2021 were selected from the Netherlands Cancer Registry (NCR). Patients were excluded if they only received best supportive care, only hormone therapy, underwent emergency resection or cytoreduction and HIPEC. Subsequently, patients were categorized into a PTR and a non-PTR group. Median overall survival (mOS) was compared between both groups and multivariable Cox regression analyses were performed.

**Results:** A total of 4599 patients were included, of whom 555 (12%) underwent PTR and 4044 (88%) did not undergo PTR. Peritoneal metastases, primary tumor location in the distal stomach and only 1 metastasis were significantly more common in the PTR group. The mOS was 14.6 months (95% CI 13.5-17.5) in the PTR group compared to 7.8 months (95% CI 7.6-8.1) in non-PTR group (P < 0.001). Multivariable analyses showed that PTR was associated with improved mOS (aHR 0.46; 95% CI 0.41-0.53; P < 0.001). If PTR was combined with systemic therapy, mOS was 17.3 months (95% CI 14.9-19.6; P < 0.001).

**Conclusion:** In patients with stage IV gastric cancer, PTR appears to be associated with better survival compared with patients that did not undergo PTR. These results imply that, although residual bias might be present, a specific subgroup of metastatic patients seems to benefit from PTR.

### PO 142 Outcomes of Pressurized Intraperitoneal Aerosol Chemotherapy (PIPAC) in Gastric Cancer Patients- Five years of experience #PO 142

#### Oral communication or poster

H. Benvenisti^1^, D. Assaf^1^, E. Mor^1^, D. Zippel^1^, M. Adileh^1^, A. Nissan^1^, A. Ben Yaacov^1^

^1^The Department of General Surgery, The Chaim Sheba Medical Center, Ramat Gan, Israel. Affiliated with the Tel Aviv University School of Medicine - Ramat Gan (Israel)

**Abstract**

**Introduction:** Peritoneal metastases represent a significant challenge in gastric cancer management. Pressurized Intraperitoneal Aerosol Chemotherapy (PIPAC) offers a novel therapeutic approach. This study investigates the perioperative and oncological outcomes of PIPAC in 40 patients with gastric cancer.

**Methods:** A retrospective analysis of a prospectively maintained peritoneal surface malignancies database between 2019 and 2024 was performed. Patient demographics, tumor parameters, perioperative and oncological outcomes were analyzed.

**Results:** Forty gastric cancer patients underwent a total of 89 PIPAC procedures, with a median of 2 procedures per patient (range 1-7). Median age was 60.1 years (range 37-85), 55% were females and median Peritoneal Cancer Index (PCI) was 14.5 (range 2-39). Poorly differentiated signet-ring cells were documented in 29 of the patients (72.5%). One patient (2.5%) had intraoperative bleeding complication with no severe postoperative complications documented. During a median follow up of 8.25 months (range 1-30), 7 patients (17.5%) proceeded to cytoreductive surgery (CRS) with hyperthermic intraperitoneal chemotherapy (HIPEC), achieving complete cytoreduction in three cases (42.9%). Median progression free survival (PFS) was 3 months and overall survival (OS) was 5.5 months. Multivariate analysis showed that the number of PIPAC procedures performed (p=0.014), PCI (p=0.012) were independently significantly associated with OS. The number of PIPAC procedures performed (p=0.009) was independently significantly associated with PFS. Patients who underwent 4 or more PIPAC procedures had significantly better OS of median 13 months, compared with those with less than 4 sessions with median OS 5 months (p=0.045).

**Conclusions:** In our cohort, PIPAC has demonstrated safety and potential efficacy in gastric cancer patients with peritoneal metastases in conjunction with systemic chemotherapy. Combining PIPAC and CRS/HIPEC in a subset of patients highlights its’ potential role as part of multimodal treatment strategy. Moreover, the observed association between increased PIPAC sessions and prolonged OS warrants further investigation for future intervention avenues.

### PO 143 NIPS or neoadjuvant chemotherapy followed by CRS + HIPEC for patients with peritoneal carcinomatosis from gastric cancer #PO 143

#### Oral communication

M.L. Fernandez Vazquez^1^, P. Lozano Lominchar^2^, L. Martin Roman^1^, S. Hernandez Kakauridze^1^, M.J. Galindo Alins^3^, M. Lopez De Felipe Gumiel^3^, N. Santana Castaño^4^, N. Palencia Garcia^1^, L. Gonzalez Bayon^5^

^1^MD. Peritoneal Carcinomatosis Unit. Gregorio Marañón Hospital - Madrid (Spain),

^2^MD. PhD. Peritoneal Carcinomatosis Unit. Gregorio Marañón Hospital - Madrid (Spain),

^3^Peritoneal Carcinomatosis Unit. Gregorio Marañón Hospital - Madrid (Spain),

^4^Peritoneal Carcinomatosis Unit. Gregorio Marañón Hospital - Madrid (Spain) - Madrid (Spain),

^5^MD. PhD. Peritoneal Carcinomatosis Unit. Gregorio Marañón Hospital - Madrid (Spain) - Madrid (Spain)

**Abstract**

**Background:** Gastric cancer (GC) with peritoneal metastases (PM) has a poor prognosis. Cytoreductive surgery (CRS) + hyperthermic intraperitoneal chemotherapy (HIPEC) is a therapeutic option that improves survival in selected patients after neoadjuvant treatment. NIPS (neoadjuvant intraperitoneal and systemic chemotherapy) is an underexplored treatment option.

**Methods:** Retrospective analysis from prospective database of patients who were treated with CRS + HIPEC from GC with PM and/or positive cytology. These were treated with neoadjuvant treatment from august 2002 to November 2022 in Peritoneal Carcinomatosis Unit of the Gregorio Marañón Hospital, Madrid.

**Results:** 40 patients underwent CRS + HIPEC;10 received NIPS and the rest neoadjuvant chemotherapy. 29 (72.5%) were diffuse, 9 (22.5%) intestinal. Median PCI in whole serie was 6. Mean PCI in NIPS since diagnostic to surgery decreased from 15.1 to 11.6; in chemotherapy was almost the same (6.6 to 7.2). CRS was CC0 in 38 patients (95%) and CC1 in 2 (5%). Median OS was 21 months; OS rates at 1, 3, and 5 years were 82%, 28%, and 20%. OS in NIPS was 22 months; not significant differences between groups. Median OS was significantly higher in patients with PCI 0 to 6 Vs 7 to 12 (24 vs 17 months; P=0,016). Not significant differences were found in histology between intestinal and diffuse ( 41 Vs 20 months; P=0,154). Median DFS was 15 months, DFS rates at 1, 3, and 5 years were 72%, 11%, and 5%. Median DFS was significantly higher in intestinal vs diffuse (43 Vs 15 months; p=0,008). 19 patients (47%) had some postoperative complication, of which 12 were severe.

**Conclusions:** Neoadjuvant followed by CRS + HIPEC is the therapeutic strategy with the best results in PM of GC. NIPS is a little explored treatment strategy, and could have a greater effect on peritoneal disease that deserves further investigation.

### PO 144 Higher doses of cisplatin and doxorubicin increase objective regression rates (PRGS 1) and improve survival after pressurized intraperitoneal aerosol chemotherapy (PIPAC) for gastric cancer (GC) with peritoneal carcinomatosis (PC). #PO 144

#### Poster

V. Khomiakov^1^, A. Ryabov^1^, A. Utkina^1^, I. Kolobaev^1^, L. Bolotina^1^, S. Aksenov^1^, A. Chayka^1^, O. Kuznetsova^1^, A. Kaprin^1^

^1^P.A. Hertsen Moscow Research Oncological Institute – branch of the National Medical Research Center of Radiology, Moscow, Russia - Moscow (Russian Federation)

**Abstract**

**Background.** GC with PC is associated with poor survival. PIPAC has demonstrated the ability to induce an objective response in peritoneal lesions as palliative treatment of patients with carcinomatosis. Low doses of doxorubicin and cisplatin are used as standard; however, phase 1 studies have demonstrated the safety of higher doses.

**Aim.** To study the effect of increased doses of cisplatin and doxorubicin on the objective regression rate and survival of patients with GC and PC undergoing PIPAC

**Methods.** From August 2013 to November 2023, 1,007 PIPACs were performed in 401 patients with GC and PC (328 - synchronous PC) in combination with standard systemic chemotherapy. Low-dose doxorubicin (1.5-2.1 mg/m2) and cisplatin (7.5-10.5 mg/m2) were used in 196 patients, while high-dose doxorubicin and cisplatin (6 mg/m2 and 30 mg/m2, respectively) were used in 198 patients. Paclitaxel was used in 7 patients (were excluded). Cox Proportional Hazards Regression Analysis was performed in a group of patients with synchronous PC who received two or more PIPACs.

**Results.** Pathological response at the second PIPAC was evaluated in 107 patients in the low-dose group and 130 patients in the high-dose group using PRGS. In high-dose group, the rate of PRGS1 increased from 24.3% to 41.5%, while the rate of PRGS3 decreased from 28.9% to 20.8%. PRGS4 was observed in only 3.9% of patients in the high-dose group.

The median survival of patients with synchronous carcinomatosis who received 2 or more PIPACs was 15.0 [14.0; 16.0] months. Low-dose doxorubicin and cisplatin were independent predictor of poor survival in GC with synchronous PC (OR 2.21 (1.54 to 3.16), p<0.0001).

**Conclusion.** The use of higher doses of cisplatin and doxorubicin for PIPAC increases the pathological regression rate and improves survival in patients with GC and synchronous PC.

## Peritoneal metastases of ovarian cancer

### PO 207 Malignant ovarian goiter: a rare and particular tumor about a case, literature review and treatment methods #PO 207

#### Poster

A.Y. Bouayed^1^

^1^BOUAYED - tlemcen (Algeria)

**Abstract**

**Introduction:** Ovarian goiters are rare endocrine tumors of the ovary, representing less than 3% of ovarian teratomas and consisting mainly of thyroid tissue. Often benign, however, malignant transformation is possible but remains exceptional. Nous rapportons le cas d’une patiente chez laquelle le diagnostic de tumeur maligne de l’ovaire était hautement probable, l’objectif est de décrire les circonstances de découverte, le diagnostic positif et les modalités thérapeutiques des goitres ovariens malins.

**Observation:** 36-year-old patient, with no particular history, postmenopausal and not taking hormonal treatment, presenting for 4 months with diffuse pelvic pain which was the subject of radiological exploration revealing the presence of a suspicious-looking right ovarian mass. . Due to the age of the patient, a left adnexectomy is performed, the pathological study reveals an ovarian teratoma, the thyroid component of which has degenerated into a papillary carcinoma.

**Discussion:** Les cas de goitres ovariens malins sont rares, les métastases sont exceptionnelles. Le diagnostic est anatomopathologique. L’attitude thérapeutique est calquée sur celle du cancer de la thyroïde.

**Conclusion:** Ovarian goiter belongs to the group of monodermal teratomas. This is a rare variety of ovarian tumors. Histopathological examination is essential for the diagnosis of this entity. Ovarian goiter can cause abnormalities in the thyroid balance. Given the risk of malignant transformation, surgical treatment remains the only therapeutic alternative.

### PO 208 Isolated Lymphatic Spread in Ovarian Cancer: A Single-Center Retrospective Study #PO 208

#### Oral communication or poster

G. Ruggeri^1^, W. Solass^2^, E. Schütz^2^, J. Kubias^1^, J. Mathis^1^, F. Siegenthaler^1^, S. Imboden^1^, M. Mueller^1^, F. Saner^1^

^1^Department of Gynecology and Gyneco-Oncology, University of Bern and Bern University Hospital - Bern (Switzerland),

^2^University Bern, ITMP - Bern (Switzerland)

**Abstract**

**Introduction:** Extensive intraabdominal/ peritoneal spread of primary ovarian/fallopian tube cancer is standard. However, only a small tumor subgroup develops isolated retroperitoneal lymph node metastases, resulting in a FIGO stage IIIA1. This study aims to identify clinical and pathological determinants for isolated lymphatic spread in ovarian cancer. **Materials and Methods:** A single-center retrospective cohort study including patients with FIGO stage IIIA1 ovarian/ fallopian tube/peritoneal cancer treated at the UBern between 12/ 2014 -07/2023. Clinical /histopathological characteristics were analyzed. **Results:** 14 patients with FIGO stage IIIA1i (n=5) or IIIA1ii (n=9) disease, with a mean age of 62.6 years (range 44 to 81 years). High-grade serous histology was most prevalent (n=8, 57%), followed by low-grade serous carcinoma (n=2, 14%) and others (endometrioid, MMT, mixed high-grade serous/clear cell, carcinosarcoma; each n=1, 7%). CA-125 was elevated in 85% of patients at diagnosis. Peritoneal cytology was positive in 55% of cases. Average of 44 lymph nodes were extracted with a median of 3 positive nodes. Extracapsular extension was observed in 5/11 patients but was not associated with positive peritoneal cytology. Lymphovascular invasion was positive in 57% (n=8) of primary tumors and in 4/5 patients with ≥5 positive lymph nodes; vascular (n=2) or perineural (n=0) invasion was uncommon. Preoperative imaging showed no signs of LN involvement in 57% of patients and in 33% with stage IIIA1ii disease. **Conclusion:** Isolated lymphatic metastases remain a rare event in ovarian cancer. LVSI of primary tumors, peritoneal cytology and preoperative imaging were unreliable determinants for lymph node metastasis in this study, each being positive in only around half of all cases. Further molecular analyses of primary tumor tissue are necessary to identify marker for lymphatic spread in ovarian cancer, which would help guide treatment decision for apparent early stage ovarian cancer.

### PO 209 Role of CRS HIPEC in Carcinoma Ovary where we stand today- With our experience from a tertiary referral oncology centre, India #PO 209

#### Oral communication or poster

M.D. Ray^1^, D. Goel^1^, C. Solomi.v^1^

^1^All India Institute of Medical Sciences - New Delhi (India)

**Abstract**

**Background:** The recurrence rates in advanced epithelial ovarian cancer (EOC) after standard treatment are very high. The addition of HIPEC increases the survival by reducing peritoneal recurrence with acceptable morbidity. In this study we assessed disease free survival (DFS) in CRS and HIPEC in upfront, interval and secondary settings and compared the DFS in CRS and HIPEC in upfront and interval setting.

**Methods:** This is a single-centre retrospective study from prospectively maintained database from 2014-2022. Our study cohort includes 400 EOC patients who underwent upfront, interval and secondary CRS or CRS and HIPEC. The drug used was Cisplatin 75mg/m2 for 60 -90 minutes in upfront, interval setting and Cisplatin 75mg/m2 + Doxorubicin 15mg/m2 for 90 minutes in secondary setting.

**Results:** The median age of our study cohort was 52 years (19- 80 years). 81.7% were in stage III and 18.3% in stage IV. 21% underwent upfront, 54% interval and 25% underwent secondary 0nly CRS. CCO was achieved in 79%, CC1 in 15%, CC2 and CC3 in 6% of patients. 40.3% underwent CRS and HIPEC in the upfront group, 44% in interval group, 51% in secondary group and 2% had palliative HIPEC. Postoperative mortality was only (1.4%) with acceptable morbidity. The median follow-up period was 65 months. The DFS in CRS + HIPEC and CRS alone were 47 months vs 45 months in upfront group (p value 0.1465), 27 months vs 14 months in interval group, (p value <0.0001) and 22 months vs 16 months in secondary group, (p value <0.0001).

**Conclusion:** The addition of HIPEC to a complete cytoreductive surgery has improved DFS in all settings of Advanced EOC. Comparing the DFS of CRS+ HIPEC in upfront vs interval setting, it showed significant difference in HIPEC Arm but overall CRS plays the pivotal role in Adv EOC.

### PO 210 Cytoreductive Surgery (CRS) and Hyperthermic Intraperitoneal Chemotherapy (HIPEC) in Patients With Primary Ovarian Cancer: Morbidity and Outcomes in Hispanics Vs non-Hispanics #PO 210

#### Oral communication or poster

M. Montealegre^1^, A. Hernandez^1^, K. Gomez^1^, C. Cash^1^, S. Yadegarynia^2^, P. Joshi^1^, A. Sinno^1^, J. Pearson^1^, H. Bahna^2^, M. Moller^3^

^1^University of Miami - Miami (United States),

^2^University of Miami/ JFK Hospital - Miami (United States),

^3^University of Chicago/University of Miami - Miami (United States)

**Abstract**

**Introduction:** CRS/HIPEC has been associated with better survival in patients with advanced ovarian cancer vs CRS alone. Outcomes data on minority racial and ethnic groups are limited. We evaluated outcomes in a majority Hispanic population compared to non-Hispanic patients with peritoneal carcinomatosis of ovarian origin.

**Methods:** 276 patients treated with CRS/HIPEC, we identified 23 patients with ovarian primary. We evaluated patient, tumor, treatment data, post-operative complications and clinical outcomes for Hispanic vs. non-Hispanics. Clavien-Dindo classification (CDC) documented 30-day post-operative morbidity. Kaplan-Meir survival analysis was used to evaluate recurrence and overall survival (OS) between groups.

**Results:** Median age was 56 (13-70), Hispanic race was 52 % (n=12). In the overall cohort, Epithelial sup-type was the most common 91% (n=21), 78 % (n=18) presented as stage III disease. R0 resection was achieved in 79.2% (n=19). Median follow-up time was 18 months (IQR: 13-30). Overall, there was no significant difference between staging and PO morbidity between Hispanic vs non-Hispanic. Of the R0 patients, 50% of Hispanics recurred vs 45.5% of non-Hispanics. Recurrence free survival (RFS) was 43.9 months (IQR 14.5-72.3) vs. 73.2 months (IQR 21.4-120.7) respectively. Median OS in months for Hispanics was 37 (IQR 25.5-47.7) vs non-Hispanics 111 (IQR 0-234.29). When looking at Epithelial subtype only, median RFS and OS were worse in Hispanics vs non-Hispanics 73% vs 44 % (p=0.15) and 39 vs 111 months (p=0.76) respectively. 2-year and 5-year OS for the whole cohort was 50% vs 80% (p=0.051) and 25% vs 50 % for Hispanics vs non-Hispanics (p=0.035).

**Conclusion:** Despite no significant difference in staging, CRS or post-op morbidity, Hispanic patients treated with CRS/HIPEC had significantly shorter survival than non-Hispanics. Epithelial subtype has a trend for worse RFS and OS in Hispanics. These findings need to be validated in a larger cohort, focusing on underlying mechanisms driving survival differences.

### PO 212 CRS-HIPEC in potentially curative treatment of primary advanced high grade serous ovarian-, tubal- and peritoneal cancer (OC). A pilot phase 2 study. #PO 212

#### Oral communication

A. Dørum^1^, M. Spasojevic^1^, M.A. Goscinski^1^, K. Flatmark^2^, E. Rokkones^1^, S.G. Larsen^1^

^1^Dr - Oslo (Norway),

^2^Prof - Oslo (Norway)

**Abstract**

**Background:** OC is the 5th leading cause of cancer death in women. In Norway, 487 new cases(2022), 70% had high-grade serous carcinoma(HGS), two-thirds advanced stage and the 5-year survival rate was 37,9%. Standard treatment is CRS followed by chemotherapy(European guidelines) within four weeks after surgery. HIPEC is questioned in the primary treatment of OC.

**Methods:** A prospective cohort study including 17 HGS OC patients with CRS-HIPEC at Norwegian Radium Hospital were compared to 42 HGS-OC controls with complete CRS without HIPEC, all FIGO stage III-IV treated 2017-2021. The groups were well balanced, sensoring date February 29th,2024. HIPEC with Carboplatin, 800 mg/m^2^(max 2m^2^) for 90 minutes at 42.0-42.5°C, were given after CRS.

**Results:** The groups were well balanced regarding stage, age, ECOG, ASA, BRCA positivity and use of adjuvant PARP-inhibitors, as well as need for colorectal anastomoses, splenectomy and diverting stoma. Time of surgery is longer in the HIPEC group, median 458 minutes(336-754) vs 290 minutes(96-540)(p<0.001) among controls supporting more complex surgery in the HIPEC group. The extra time for the HIPEC procedure is 135-150 minutes. The PCI value was calculated in some of the controls group based on the operation report, the median was 13 in both groups(3-33 in the HIPEC group,1-23 among controls). Four Accordian 4 cases occurred in the HIPEC group(bowel perforation(1),bleeding(1),urinary bladder leakage(1) and intraabdominal infection(1) and four among the controls(bowel perforation(2),stomal necrosis(1),bowel obstruction(1). Overall survival(OC) and disease free survival(DFS) were not significantly different in the two groups. Median time to subsequent 2.line chemotherapy was 19 months among the controls and 30 months in the HIPEC group.

**Conclusions:** CRS-HIPEC was administered with acceptable complication rate compared to standard CRS in patients. The OS and DFS was not significantly different, however, the HIPEC treatment is more resource demanding because of increased time in the operating theatre.

### PO 213 Evaluation of predictive factors of delayed return to intended oncology treatment after interval cytoreductive surgery in advanced epithelial ovarian cancer #PO 213

#### Oral communication or poster

G. Poutrel^1^, B. Valenzuela^1^, F. Quenet^1^, S. Carrere^1^, S. Thezenas^1^, D. Zambrano^1^, O. Sgarbura^1^, P.E. Colombo^1^

^1^Institut régional du Cancer de Montpellier (ICM)-Val d’Aurelle, Montpellier, France. - Montpellier (France)

**Abstract**

**Background:** Early return to intended oncology treatment (RIOT) after cytoreductive surgery for advanced epithelial ovarian cancer (AEOC) improves survival outcomes. The aim of this study was to evaluate predictive factors of delayed initiation of adjuvant chemotherapy after interval cytoreductive surgery for AEOC.

**Methods:** In this retrospective study, we included all patients who underwent interval cytoreductive surgery (CRS) for AEOC after neoadjuvant chemotherapy between November 2012 and April 2023 in the Montpellier Cancer Institute. Logistic regression analysis was used to evaluate predictive factors of delayed RIOT (period of time between CRS and the first cycle of adjuvant chemotherapy > 42 days).

**Results:** A total of 157 patients with median PCI of 15 were included for analysis - 125 (80%) in the delayed group and 32 (20%) in the early group. One hundred twenty seven patients (81%) had a high Aletti score (≥ 8), with a bowel resection rate of 66%. In our study, we observed anastomotic leakage for 3% of patients and performed a protective ileostomy for 17%. Clavien-Dindo classification grade ≥ IIIa occurred in 19 (12%) patients. The mean time to RIOT was 60 days for the delayed group and 37 days for the early group. In univariate analysis, the high Aletti score was significantly associated with a delayed RIOT (p = 0.017). This association was not found in multivariate analysis (p = 0.964).

**Conclusions:** In our study, high Aletti score was associated with a delayed RIOT in univariate analysis. The loss of significance in multivariate analysis may be explained by the high rate of delayed RIOT (80%). Prospective studies are needed to evaluate the high Aletti score as a predictive factor of delayed RIOT. Additionally, time to RIOT may be improved in our center by optimizing the care of our patients.

### PO 214 Impact of HIPEC Introduction in interval debulking surgery for advanced ovarian cancer: A real-world population study #PO 214

#### Oral communication or poster

B. Valenzuela-Mendez^1^, F. Quénet^2^, S. Thezenas^2^, S. Carrère^2^, V. D’hondt^2^, M. Fabbro^2^, A. Mourregot^2^, P. Rouanet^2^, O. Sgarbura^2^, P.E. Colombo^2^

^1^Hôpital Européen Georges Pompidou - Paris,

^2^Institut Régional du Cancer de Montpellier (ICM) - Montpellier

**Abstract**

**Introduction:** Ovarian cancer, a major cause of cancer-related deaths among women, requires innovative treatments. Hyperthermic Intraperitoneal Chemotherapy (HIPEC) has been proposed as an adjunct to standard cytoreductive surgery (CRS) for advanced ovarian cancer. This study evaluates the impact of HIPEC by comparing outcomes before and after its introduction during interval debulking surgery (IDS) at the Montpellier Cancer Institute.

**Material and Methods:** This retrospective analysis used data from a prospective ovarian cancer database (BCB ovaire N° NCT03976999), including patients who underwent complete or near complete (CC0 or CC1) interval CRS for stage III and IV ovarian cancer from 2014 to December 2022. Since February 2018, HIPEC has been systematically integrated at IDS for stage III and occasionally for stage IV, based on the response to neoadjuvant chemotherapy (NACT).

**Results:** The study included 179 patients, divided into IDS+HIPEC (93) and IDS (86) groups. Patient and tumor characteristics were comparable, except for FIGO stages, with more stage IV cases in the non-HIPEC group (30.2% vs 8.7%). Maintenance treatment rates were similar, with higher PARP inhibitors +/- bevacizumab usage in the HIPEC group (18.6% vs 7.74%). The median overall survival (OS) was 44.7 months for IDS and not reached for IDS+HIPEC. Postoperative morbidity, hospital stay length, and time to adjuvant chemotherapy were not significantly different between groups. In multivariate analysis, we observed a significant protective and independent effect on OS and PFS1 from treatment with HIPEC (HR=0.59 and 0.64) and maintenance therapies with PARP inhibitors (HR=0.32 and 0.24).

**Conclusion:** The introduction of HIPEC in the treatment of advanced ovarian cancer has demonstrated promising improvements in survival outcomes. This study supports the incorporation of HIPEC into treatment protocols, advocating for further research to refine patient selection and treatment strategies.

### PO 215 Prognostic factors for survival in ovarian cancer after cytoreductive surgery and hyperthermic intraperitoneal chemotherapy: 11-year retrospective review #PO 215

#### Oral communication or poster

A. Vale Guimarães^1^, A. Varejão^2^, M. Peyroteo^1^, C. Ribeiro^1^, P. Ferreira Pinto^1^, M. Marques^1^, M.J. Madeira-Cardoso^1^, M. Pires^2^, J. Abreu De Sousa^1^

^1^Instituto Português de Oncologia - Porto, Surgical Oncology - Porto (Portugal),

^2^Instituto Português de Oncologia - Porto, Gynecology - Porto (Portugal)

**Abstract**

**Background:** Ovarian cancer is a leading cause of cancer-related death in women, with most cases diagnosed in advanced stages. Cytoreductive surgery and hyperthermic intraperitoneal chemotherapy(CRS-HIPEC) has been introduced as a potentially curative treatment. There is still uncertainty regarding patient selection and optimal timing for surgery, as well as the long-term outcomes.

The goal of this study was to assess the outcomes of patients with ovarian cancer submitted to CRS-HIPEC in our institution.

**Methods:** A retrospective observational study was conducted using data from patients who underwent CRS-HIPEC with cisplatin for ovarian cancer between 2012 and 2022.

**Results:** Our sample included 67 women, average age 57.5 years old at the time of surgery. The most common histological subtypes were high-grade serous carcinoma in 48 patients(71.6%), low-grade serous carcinoma in 8(11.9%) and mucinous carcinoma in 7(10.4%). Most patients were classified as FIGO stage IIIB(14.9%) or IIIC(77.8%). Sixteen(23.9%) had ascites at the time of surgery and the average Peritoneal Carcinomatosis Index(PCI) was 12.8. Upfront surgery was performed in 4 patients(6%), interval surgery in 31(46.3%) and CRS-HIPEC after disease recurrence in 32(47.8%). Complete cytoreduction was achieved in 63 patients(94%), with 28(41.8%) requiring gastrointestinal resection. Major complications(Clavien-Dindo III-IV) occurred in 7 patients(10.5%). Disease-free survival(DFS) and overall survival(OS) at 5 years were 20% and 52.5%, respectively. There was a negative correlation between the PCI and both DFS and OS(r=-0.381, p=0.002 and r=-0.463, p<0.001 respectively). Subgroup analysis revealed a worse DFS in patients with ascites(p=0.042), more advanced stages(p=0.002) and with high-grade serous carcinoma(p=0.006) in the CRS-HIPEC after recurrence group.

**Conclusions:** Our results indicate that CRS-HIPEC is an important treatment modality for ovarian cancer with peritoneal metastases, with acceptable morbidity rates and with outcomes similar to those previously described. The presence of ascites, high PCI and more advanced stages were associated with worse survival after surgery.

### PO 216 Role of RPLND in Carcinoma Ovary Analysis from Tertiary Care Centre in India #PO 216

#### Oral communication or poster

B. Bhukkal^1^, M.D. Ray^1^, D. Goyal^1^

^1^AIIMS - New Delhi (India)

**Abstract**

**Introduction:** Ovarian cancer is the most lethal gyanecologic cancer worldwide, and the median age at diagnosis is around 63 years in most developed countries. (1) Nearly three-quarters of new cases are diagnosed at advanced stages. (2) ovarian cancer (OC) is the 8th most common incident cancer and ranks eighth in cancer-related deaths globally. The standard treatment of ovarian cancer is maximal cytoreductive surgery followed by chemotherapy is the standard of care for advanced cancer. The surgical treatment of ovarian cancer aims to achieve maximal cytoreduction. (3)

As per the present concept, CRS includes removal of all macroscopic residual disease but the routine removal of the pelvic and retroperitoneal lymph nodes is not an integral part of cytoreduction. Lymph nodes status is an important prognostic factor in patients with ovarian cancer.

The retroperitoneal lymphatic spread has been reported to be a common feature both in early and advanced ovarian cancer patients, the rate of lymph node metastasis is about 20–41%, which can reach up to 50–80% in advanced patients (FIGO stage III-IV). (4) National Comprehensive Cancer Network (NCCN) recommend that systematic retroperitoneal lymphadenectomy (including pelvic and paraaortic lymphadenectomy) should be included in the primary surgery of early ovarian cancer patients. but in advanced stages of ovarian cancer Lymphadenectomy benefit still controversial some retrospective studied showed improve prognosis (4–6). Recently, differently from previous studies, the LION study, which was a well-designed randomized controlled trial, did not report any survival advantage for systematic lymphadenectomy in patients without bulky lymphadenopathy. In addition, retroperitoneal lymphadenectomy may increase intraoperative and postoperative complications, such as bleeding, vascular injury, lymphocysts, infection, intestinal fistula, and chylous fistula. (7)

In view of above controversial issue, Is RPLND associated with More postoperative complication with no survival benefit. We performed a retrospective analysis to investigate role of RPLND in advanced ovarian cancer.

## Pseudomyxoma peritonei

### PO 63 Cytoreductive Surgery and HIPEC for Patients with Pseudomyxoma Peritonei and Liver Hilum Involvement: A Single Institution Experience #PO 63

#### Oral communication or poster

B. Williams^1^, S. Kulkarni^1^, J. Shin^1^

^1^University of Southern California - Los Angeles (United States)

**Abstract**

**Background:** Cytoreductive surgery (CRS) and hyperthermic intraperitoneal chemotherapy (HIPEC) are the mainstay of treatment for patients with pseudomyxoma peritonei (PMP) from LAMN. Unfortunately, those with liver hilum involvement (LHI) may not be appropriate candidates. Most recent PSOGI statement regards LHI as a relative contraindication to treatment.

**Methods:** We conducted a retrospective review of all patients with PMP who underwent CRS and HIPEC at a single institution from 2016 to 2023. Patients were excluded if the etiology of peritoneal disease was due to causes other than appendiceal mucinous neoplasm, such as adenocarcinoma from colon or appendix. Relevant demographic, operative, and outcomes data were reviewed, and those with and without LHI were compared. Surgeries on patients with LHI were performed in combination with a hepatobiliary surgeon.

**Results:** Twenty-four patients were analyzed including 14 (58%) patients with LHI and 10 (41.7%) without. Demographics were similar between groups, however patients with LHI versus no LHI had higher peritoneal carcinoma index (PCI) scores (23.8 ± 4.6 vs. 12.1 ± 5.5; p < 0.001). Thirty-day major complication rate (Clavien-Dindo > II) was higher for patients with LHI without reaching statistical significance (29% vs. 0%, respectively; p=0.064). There was 1 (4%) mortality in the cohort, which occurred 12 months postoperatively in a patient with LHI.

Overall, 18 (75%) patients achieved complete cytoreduction (CC; CS 0/1) including 9 (64%) patients with LHI and 9 (90%) without (p=0.151). For patients with PCI < 20 (N=13), 5 (100%) patients with LHI and 7 (87.5%) without LHI achieved CC (p=0.411). Conversely, when PCI is > 20 (N=11), 2 (100%) patients without LHI and only 4 (44%) patients with LHI achieved CC (p=0.154).

**Conclusion:** CRS and HIPEC can be successful for patients with LHI with comparable perioperative outcomes. As such, CRS and HIPEC in patients with LHI should be considered when appropriate.

### PO 64 Using machine learning to detect at-risk patients of pulmonary embolisms after cytoreductive surgery and hyperthermic intraperitoneal chemotherapy #PO 64

#### Oral communication or poster

M. Alberto Vilchez^1^, L. Hoff^1^, S. Gül-Klein^1^, B. Weiß^1^, B. Rau^1^

^1^Charité - Univeristätsmedizin Berlin - Berlin (Germany)

**Abstract**

**Background:** A growing number of evidence supports cytoreductive surgery (CRS) with or without intraperitoneal chemotherapy (i.e. HIPEC) to palliate or even cure peritoneal surface malignancies. The complexity of the surgery is extended to the prevention and/or management of perioperative complications. Using K-Means Clustering, a conventional machine-Learning technique, we were able to identify clusters of factors associated with a higher risk of pulmonary embolisms (PE) following CRS and HIPEC. Scarce literature regarding this matter reports a prevalence of thromboembolic events around 13% with a significant quota of readmissions into the intensive care unit (ICU). We set out to look for plausible causes of thromboembolic events (TEE), with special interest in PE.

**Material and Methods:** This retrospective study analyzed data from 356 patients who underwent CRS and HIPEC between 2018 and 2023 in our high-volume center. The prevalence of PE was 3,3,%. A cluster exercise was performed utilizing K-Means Clustering. The best cluster found (Calisnki-Harabasz Score 639) had 9 variables and 3 groups: Age, Weight, OP Length, Cisplatin, Systolic Average Real Variability, Systolic Pressure AUC Index, Intraoperative Blood loss, Preoperative Creatinine, Preoperative Thrombocytes. After the model ran, we carried out a multivariable logistic regression using the cluster groups, important clinical variables as well as confounding factors to assess the relationship between these variables and the outcome.

**Results:** We found a non-statistically significant trend of a higher incidence of pulmonary embolisms (PE) in older patients (Mean 71.7), higher preoperative serum creatinine levels, and intraoperative systolic pressure variability. Remarkably, patients with Pseudomyxoma peritonei (PMP) are at a statistically significantly higher risk of developing PE.

**Conclusions:** Our study will facilitate risk management and exploration of new thromboembolic prophylactic measures to avoid PE and TEE in patients with peritoneal surface malignancies, primarily focused in PMP at-risk patients.

### PO 65 Complex Management of Umbilical Hernia Revealing Pseudomyxoma Peritoneal from Low-Grade Appendiceal Mucinous Neoplasm: A Case Study #PO 65

#### Oral communication or poster

L. Lino-Silva^1^, R. Salcedo-Hernánez^2^, P. Frías-Fernández^3^

^1^onterrey Institute of Technology and Higher Education, Mexico city campus - mexico city (Mexico),

^2^National Cancer Institute (Mexico) - Mexico city (Mexico),

^3^Oncology center, Tula general Hospital - Tula de Allende (Mexico)

**Abstract**

**Background:** Umbilical hernias are defined as pa defect localized at the midline in the center of the umbilical ring. The general trend has seen more frequent umbilical hernia repairs in men compared to women, with emergency repairs increasing over the last decade. Appendiceal tumors, are often identified in emergency settings and post-operatively, complicating management. The pathological subtype significantly affects the prognosis of patients with appendiceal tumors. Mucinous appendiceal neoplasms (MAN) are known for their copious mucus production and are a primary cause of pseudomyxoma peritoneal (PMP). Low-grade appendiceal mucinous neoplasms (LAMN) grow slowly, are generally well-differentiated, expansive rather than infiltrative, with high mucus content. Peritoneal spread through rupture, leading to diffuse mucus distribution, is common.

**Case Presentation:** A 36-year-old male, with no significant personal pathological history. He began experiencing increased supraumbilical region volume in December 2020, which intensified with effort and reduced at rest. Two weeks prior to consultation, he developed pain and fissures over the defect with serous liquid discharge, leading him to seek emergency care. An ultrasound identified a paraumbilical and umbilical region defect with protruding intestinal loops and fluid. During urgent surgical exploration for umbilical hernia repair, a supraumbilical hernial defect with abundant fluid discharge was noted. Extending the surgical approach revealed numerous gelatinous implants in the abdominal cavity and a hardened appendix with gelatinous implants, resulting in an appendectomy and partial omentectomy. Histopathological report confirmed low-grade mucinous neoplasia of the appendix and pseudomyxoma peritoneal across the omentum and hernial sac.

**Conclusions:** This case highlights the complexity in diagnosing and managing appendiceal tumors manifesting as emergency complications like hernia, emphasizing the urgency in surgical intervention and the potential for extensive abdomen involvement. It underlines the critical nature of histopathological analysis in confirming diagnoses such as PMP, stemming typically from LAMN, thus shaping targeted therapeutic interventions.

### PO 66 Realistic gap between surgical and pathological staging of PCI in cytoreductive surgery (CRS) for appendiceal and colo-rectal mucinous neoplasms: Ten-years single center experience #PO 66

#### Poster

F. Casella^1^, F. Meloni^1^, C. Puccio^1^, R. Rigoli^1^, I. Zacchi^1^, G. De Manzoni^1^

^1^General and Upper GI Surgery, University of Verona - Verona (Italy)

**Abstract**

**Background:** The peritoneal cancer index (PCI) is one of the main prognostic factors of patients with peritoneal carcinomatosis and represents a fundamental factor in defining the operability of some patients. The aim of this study is to define the accuracy of staging of surgical PCI by correlating it to the pathological PCI.

**Methods:** All 124 consecutive patients who underwent CRS at our center between 2013 and 2023 for peritoneal metastasis of appendiceal and colo-rectal mucinous neoplasms were retrospectively included in the study. We analyzed demographical and oncological features of these patients and compared sPCI and pPCI identifying the degree of concordance.

**Results:** The median age was 59,5 and sex ratio was 65:59 (F:M). 41 patients underwent CRS, and 73 underwent CRS and HIPEC with Cisplatin ad Mitomycin C. The primitive tumor was: LAMN in 49 patients (40%), HAMN in 11 patients (9%), appendiceal mucinous adenocarcinoma in 34 patients (27%) and mucinous adenocarcinoma of colon in 30 patients. (24%). CC-0 was achieved in 89% of the sample and CC-1 was achieved in the remaining 11%. Seventy patients (56%) were subjected to CRS for synchronous PM, while fifty-four patients (44%) for metachronous PM. 84 patients were subjected to primary CRS, 40 underwent to CRS as a second-look; of these 37 received previous chemotherapy.

The median sPCI index was 13 (mean 15) whilst the median pPCI was 9 (mean 11,5), with a median difference of 4. Overall pathological downstaging was found in 80,5%.

**Conclusions:** A difference between sPCI and pPCI can be found in patients who underwent to CRS after chemotherapy or a previous surgery, such as in second-look CRS and peritoneal recurrence. So pPCI may provide a more accurate evaluation of the peritoneal disease extent.

### PO 67 The prognostic role of KRAS and GNAS in the prediction of survival outcomes in PMP #PO 67

#### Oral communication

G. Colletti^1^, S. Kusamura^1^, F. Perrone^1^, D. Baratti^1^, F. Nava^1^, F. Pietrantonio^1^, K. Navin^1^, M. Guaglio^1^, M. Deraco^1^

^1^Fondazione IRCCS Istituto Nazionale Tumori Milano - Milano (Italy)

**Abstract**

**Background:** Traditional clinicopathological prognostic factors do not allow for an accurate prediction of long-run oncologic outcomes of patients affected by pseudomyxoma peritonei (PMP) after Cytoreductive Surgery (CRS) and HIPEC. Literature data on the prognostic role of molecular markers KRAS-mut and GNAS-mut is inconsistent and therefore, the endpoint of this study was to assess their link with PMP survival.

**Material and methods:** Data from patients with PMP from mucinous appendiceal neoplasms who underwent cytoreduction and hyperthermic intraperitoneal chemotherapy (CRS-HIPEC) in the National Cancer Institute of Milan were retrospectively investigated. Tumour samples were obtained after CRS-HIPEC and we carried out next-generation sequencing (NGS) of 50 gene’s hotspot regions contained in the Hotspot Cancer Panel v2 using the Ion Torrent Personal Genome Machine platform (Life Technologies). Survival analysis was conducted using multivariate Cox regression models.

**Results:** One hundred and twenty patients were included. KRAS and GNAS mutations were observed in 77 (64%) and 46 (38%) patients respectively. The percentages of allelic mutations were 8.4% and 5.4%, respectively for KRAS and GNAS. Patients with a KRAS-mut had significantly worse Progression-Free Survival (PFS) and Overall Survival (OS) compared to KRAS-wt cases (p=0.008). Also, patients with GNAS mutation had worse PFS and OS than GNAS-wt (p=0.2 and p=0.002, respectively). After multivariate Cox regression analysis, the independent predictors of shorter PFS were KRAS (HR: 2.45, CI95%: 1.02-5.90, p=0.045), and PCI (HR: 2.17, CI95%: 1.28-3.68, p=0.003). The independent predictors of OS were GNAS-mut (HR: 3.31, CI95%: 0.91-1.12, p=0.054), gender (HR: 0.13, CI95%: 0.025 -0.67, p=0.015), and age (HR: 2.75, CI95%: 1.14-6.65, p=0.024).

**Conclusions:** These findings confirmed the KRAS-mut and GNAS-mut as new independent prognosticators of PMP. Despite its retrospective nature, this study constitutes the most extensive case series of PMP assessing the prognostic significance of these molecular drivers of tumour progression.

### PO 68 The Microbiome of Pseudomyxoma Peritonei: A Scoping Review #PO 68

#### Poster

S. Portela^1^, A. Jain^1^, M. Flood^1^, A. Lavelle^2^, M. Patel^3^, O. Aziz^3^, S. Warrier^1^, A. Heriot^1^, H. Mohan^1^

^1^Peter MacCallum - Melbourne (Australia),

^2^University College Cork - Cork (Ireland),

^3^The Christie - Manchester (United Kingdom)

**Abstract**

**BACKGROUND:** In pseudomyxoma peritonei (PMP) prognosis is largely influenced by histological subtype and extent of disease. There is growing interest in the role of microbiome in carcinogenesis and tumour progression, but few studies have examined the microbiome of PMP. The aim of this scoping review was to determine what micro-organisms have been identified in PMP samples and examine the evidence of their role in disease outcome.

**METHODS:** The methodology was developed following The Preferred Reporting Items for Systematic reviews and Meta-Analyses extension for Scoping Reviews framework and checklist and subsequently registered with the Open Science Framework. PubMed, EMBASE and Scopus databases were used to identify potentially relevant studies.

**RESULTS:** Nine relevant studies were identified, including a total of 107 patients with histologically confirmed PMP.

Bacteria were identified from tumour and mucin specimens collected intra-operatively. At the phylum level, Proteobacteria, was consistently detected in greatest relative abundance in PMP tumour tissue and cellular and acellular mucin, followed by Actinobacteriota. Several species, including Pseudomonas plecoglossicida and the novel Parapseudoflavitalea muciniphila gen. nov., sp. nov., were also identified. High-grade specimens showed significantly higher bacterial density, H. pylori inclusive, than low-grade specimens and non-neoplastic non-perforated appendix specimens. The bacterial densities from the latter two cohorts were not significantly different. Microbial detection methods varied significantly between studies, and the use of laboratorial and microbiological control was inconsistent, clouding the significance of the results.

**CONCLUSIONS:** There is evidence of an altered bacterial profile in PMP samples more closely resembling the microbiome in inflammatory bowel disease than healthy gut, the significant of which is unclear. Significant methodological challenges remain in this field of study. This scoping review supports the need for further analysis of the bacterial profile of PMP, using methodologies that reduce the rate of contamination and that enable the comparison with an appropriate ‘control’ microbiome.

### PO 69 Two-stage surgery in pseudomyxoma peritonea #PO 69

#### Oral communication

M. Jiménez Monasterio^1^, M.L. Fernández Vázquez^1^, Á. Landeras López^1^, C.R. Martínez^1^, C. Morales García^1^, M. López De Felipe Gumiel^1^, N. Palencia García^1^, L.A. González Bayón^1^

^1^HGUGM - Madrid (Spain)

**Abstract**

**BACKGROUND:** Psedomyxoma peritonei (PMP) is characterized by an appendiceal origin, mucinous ascites, and peritoneal tumor redistribution. Recommended treatment includes cytoreductive surgery (CRS) and hyperthermic intraoperative chemotherapy (HIPEC). For massive peritoneal disease, a two-stage surgery can be considered.

**METHODS:** This retrospective descriptive study included all patients undergoing two-stage CRS + HIPEC for PMP from 2000 to 2024. Preoperative criteria included histology of LAMN or adenocarcinoma with massive low-grade peritoneal carcinomatosis and previous surgeries. Intraoperative criteria included hemodynamic instability, coagulopathy, massive transfusion, or prolonged surgical times (>10-14 hours).

**RESULTS:** Eight patients were included: 3 LAMN, 2 low-grade adenocarcinoma, and 3 moderate-grade adenocarcinoma.

The intraoperative Peritoneal Cancer Index (PCI) ranged from 29 to 39 (median 35).

The first surgery lasted 10-14 hours, focusing on the upper abdomen (mainly areas 0, 1, 2, and 3). Common procedures included major omentectomy with splenectomy and left diaphragmatic peritonectomy, right diaphragmatic peritonectomy with glissonectomy, and minor omentectomy with hepatoduodenal ligament and lesses sac peritonectomies. One patient underwent total gastrectomy. All patients had HIPEC with Mitomycine C during 90 minutes, but one with Oxaliplatin 30 minutes. Complete cytoreduction (CC0) in the operated areas was achieved in all patients in stage one.

The second stage occurred 4-8 months later, targeting the lower abdomen to achieve CC0-CC1. No disease was found in previously operated areas. Again, HIPEC with Mitomycine C or Oxaliplatin was performed. Two patients are pending the second stage.

Among those completing treatment, five are alive, one deceased. Five-year overall survival (OS) is 100%, and 10-year OS is 87.5%. one-year disease free survival (DFS) is 83.3%, and 3-year DFS is 50%. All patients experienced peritoneal recurrence, treated with new CRS +/- HIPEC.

**CONCLUSION:** In our experience, two-stage strategy for massive PMP is a feasible therapeutic strategy, with good oncological results, which can be considered in reference centers.

### PO 70 Clinical Value of Different Strategies of Hyperthermic Intraperitoneal Chemotherapy in Pseudomyxoma Peritonei of Appendiceal Origin: A Retrospective Clinical Study #PO 70

#### Oral communication or poster

Y. Fu^1^, R. Yang^1^, Y. Li^2^

^1^Beijing Shijitan Hospital - Beijing (China),

^2^Beijing Shijitan Hospital,Beijing Tsinghua Changgung Hospital - Beijing (China)

**Abstract**

**Objective:** To evaluate the effects of different Hyperthermic intraperitoneal chemotherapy (HIPEC) regimens on pseudomyxoma peritonei (PMP) of appendiceal origin.

**Methods:** The data of PMP patients undergoing cytoreductive surgery (CRS) plus HIPEC at our center from January 2008 to December 2023 were retrospectively analyzed. PMP patients were divided into cisplatin+docetaxel(CD) group and cisplatin+mitomycin C(CM) group according to different HIPEC regimens. The clinicopathological and prognostic data were compared between the two groups. Univariate and multivariate survival analyses were performed to determine independent prognostic factors for PMP. subgroup survival analysis were used to evaluate the survival differences between the two HIPEC regiments.

**Results:** A total of 564 PMP patients undergoing CRS+HIPEC were included in this study, including 503 patients(89.2%) in CD group and 61 patients(10.8%) in CM group. There were no significant differences between the two groups in general clinicopathological characteristics and CRS+HIPEC parameters(P>0.05). Survival analysis showed that the median overall survival(OS) of CD group was significantly longer than that of CM group(156.3 months vs.60.5 months,P=0.006). There was no statistically significant difference in the incidence of SAEs between the two groups(P>0.05). Multivariate survival analysis identified five independent prognostic factors affecting survival in PMP patients: HIPEC regimen, peritoneal cancer index(PCI), completeness of cytoreduction(CC), lymphatic metastasis and tumor marker. subgroup analysis showed that in patients with PCI≥27, CC2/3, high grade pathological diagnosis and without lymphatic metastasis, CD regimen can significantly prolonged OS compared with CM(P=0.005).

**Conclusions:** Following standard CRS, HIPEC with CD regimen could have better survival advantage than CM regimen for PMP patients.

Subgroup survival analysis of the two groups

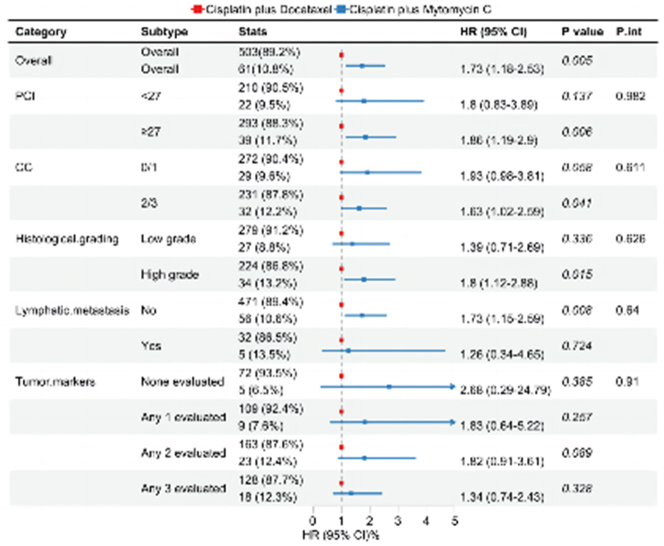



### PO 71 Laparoscopic radical appendectomy for appendicular mucocele, a video presentation #PO 71

#### Oral communication

A. Abdulrahem^1^, A. Alyami^1^, M. Alzamanan^1^, S. Almowallad^1^, M. Fagihi^1^, Q. Alqurashi^1^, R. Alwadai^1^, A. Saihb^1^, M. Alhajlan^1^, M. Alyami^2^

^1^king khalid hospital - najran (Saudi Arabia),

^2^king khalid hospital - NAJRAN (Saudi Arabia)

**Abstract**

**Background:** Appendicular mucocele may arise from benign of malignant changes and if left untreated or ruptured inside the abdomen increase the risk of seeding of cells with subsequent pseudomyxoma peritonei.

**Case summury:** This is a 26 years old male patient was diagnosed with appendicular cyst with suspesion for mucocele underwent laparoscopic radical appendectomy and hsitopathology report showed APPENDICULAR MUCINOUS NEOPLASM with negative margin. this is a demontration of how we do it ?

**Conclusion:** Laparoscopic radical appendectomy for appendicular mucocele with intraoperative careful handling of the tissue to prevent spillage of the appendicular content was effective and safe with a safety margin.

## Authors index

A

Aalbers A.G.J. PO 107, PO 108

Abatini C. PO 165

Abba J. PO 202, PO 99, PO 118, PO 120

Abboud K. PO 49

Abdel Gawad W. PO 196, PO 199

Abdul Kadir H. PO 192

Abdullah H.R. PO 192

Abdulrahem A. PO 174, PO 175, PO 176, PO 177, PO 71

Abid M. PO 188

Abreu De Sousa J. PO 26, PO 215

Acimov O. PO 123

Adileh M. PO 142

Agarwal K. PO 198

Ahmed I.S.H. PO 122

Ahn S.H. PO 138

Ahuja V. PO 198

Ainsworth A. PO 12

Ainsworth A.P. PO 43, PO 132

Akaraviputh T. PO 81

Akhter J. PO 27, PO 28, PO 55

Aksenov S. PO 144

Al Dokanny B. PO 199

Al Dokany B.A. PO 196

Al Ghamedi A. PO 199

Al Hamdani O. PO 163

Al Hosni M. PO 163

Al Mansor E. PO 199

Al Mutawa A.R. PO 199

Al Mutawa A. PO 196

Albaqmi O. PO 126

Alberto Vilchez M.E. PO 128

Alberto Vilchez M. PO 64

Aldayel F. PO 199

Alexandre S. PO 26

Alhajlan M. PO 174, PO 175, PO 176, PO 177, PO 71

Alhama J. PO 17

Allievi N. PO 115

Almog A. PO 51

Almowallad S. PO 174, PO 175, PO 176, PO 177, PO 71

Alonso-Casado O. PO 184

Alqurashi Q. PO 174, PO 175, PO 176, PO 177, PO 71

Alwadai R. PO 174, PO 175, PO 176, PO 177, PO 71

Alyami A. PO 174, PO 175, PO 176, PO 177, PO 71

Alyami M. PO 174, PO 175, PO 176, PO 177, PO 71

Alzamanan M. PO 174, PO 175, PO 176, PO 177, PO 71

Amira G. PO 148

Amroun K. PO 49

Andersen P. PO 43

Andrino Poulou E. PO 103

Ang A.J.Y. PO 02

Ansaloni L. PO 112

Antón Rodríguez C. PO 33

Anwar A.Z. PO 111

Aparicio T. PO 124

Aral M. PO 173

Aranaz V. PO 41, PO 42, PO 195

Arciuolo D. PO 164

Arjona A. PO 41, PO 42, PO 101

Arjona-Sanchez A. PO 103

Arjona-Sánchez Á. PO 17

Arvieux C. PO 202, PO 118, PO 120

Asakage M. PO 136

Ashwin K.R. PO 198

Asplund D. PO 116

Assaf D. PO 142

Atmaca M. PO 128

Attalla El Halabieh M. PO 165

Auger M. PO 145

Aulicino M. PO 165

Auris H. PO 34

Aziz Z. PO 148

Aziz O. PO 68

B

Babin S. PO 52

Babu Ramalingam M. PO 40

Badar S. PO 27, PO 28, PO 55

Badia-Ramentol J. PO 179

Bae J.K. PO 168

Bae S.K. PO 180, PO 15, PO 180

Bagalà C. PO 164

Bahna H. PO 82, PO 171, PO 172, PO 45, PO 210

Bakrin N. PO 80

Balasubramanian A. PO 200

Bang C. PO 180, PO 15, PO 180

Banzuela J.A. PO 54

Barat S. PO 27, PO 127

Baratti D. PO 48, PO 56, PO 94, PO 95, PO 67

Bardet S.M. PO 07, PO 16

Bardet S. PO 06

Baron E. PO 162

Baron T. PO 124

Bartlett D. PO 76, PO 77

Bassetti C. PO 06

Bau Mortensen M.I.C.H.A. PO 50

Bayney S. PO 115

Beets G.L. PO 107

Bek S. PO 191

Belkhadir Z.H. PO 187, PO 204

Bellosillo B. PO 179

Ben Yaacov A. PO 142

Bénard F. PO 140

Benchimol D. PO 78

Bencivenga M. PO 87

Benhaim L. PO 203

Benkabbou A. PO 187, PO 204

Benvenisti H. PO 142

Benzerdjeb N. PO 130

Berben F. PO 141

Berkane S. PO 29, PO 178, PO 96

Bertolucci A. PO 03

Berx G. PO 104

Beunon P. PO 203

Bexe Lindskog E. PO 102, PO 116

Bhukkal B. PO 151, PO 216

Biacchi D. PO 48

Bijelic L. PO 129

Bild A. PO 18

Bin Massuryono M.D. PO 189

Birgisson H. PO 73

Bishara I. PO 18

Bisquera Jr. O. PO 54

Blons H. PO 148

Boerma D. PO 93

Boige V. PO 61

Boissard A. PO 04

Bolotina L. PO 144

Bonilla C. PO 46, PO 137

Bonnefoy I. PO 130

Borghi F. PO 201

Borisov S.M. PO 86

Borowsky P. PO 82, PO 171, PO 172, PO 45

Bosch Ramirez M. PO 129

Bouayed A.Y. PO 207

Bouça-Machado T. PO 173

Bouchard-Fortier A. PO 140

Boudy V. PO 161

Boulanger-Gobeil C. PO 140

Bourova-Flin E. PO 99

Boutayeb S. PO 187

Bouvy N.D. PO 19

Braet H. PO 19

Branchu J. PO 13

Breakeit M. PO 27, PO 28

Brigand C. PO 49

Brudvik K.W. PO 106

Buffart T.E. PO 108, PO 121

Bugarin S. PO 123

Bura F.I. PO 17

Burger J.W.A. PO 91

Burton M. PO 12

C

Cabras A. PO 56

Cai M.Z. PO 40, PO 206

Cai M. PO 152, PO 83, PO 84, PO 38, PO 190, PO 192

Calon A. PO 179

Caravaca I. PO 41, PO 42, PO 195

Carbonell-Morote S. PO 41, PO 42

Carboni F. PO 48

Carlos R. PO 170

Carrere S. PO 213

Carrère S. PO 214

Cascales P. PO 41, PO 42

Casella F. PO 87, PO 95, PO 66

Cash C. PO 82, PO 171, PO 172, PO 45, PO 210

Cashin P. PO 147, PO 73, PO 102, PO 116

Castella-Bataller L. PO 184

Catena F. PO 112

Cavalleri T. PO 94

Cecil T. PO 60, PO 115, PO 126

Ceelen W. PO 19, PO 36, PO 104

Ceelen W.C. PO 97

Chandrakumaran K. PO 126

Chang S.J. PO 08, PO 09, PO 10

Chang S. PO 18, PO 181

Chardalias L. PO 88

Charton C. PO 138

Chaurasiya S. PO 139

Chayka A. PO 144

Cheah C.C.M. PO 206

Chen C. PO 139

Cheok S.H.X. PO 152, PO 38

Chia D. PO 74

Chia D.C. PO 97

Chia C. PO 84, PO 191

Chia S.C. PO 40, PO 192, PO 206

Chia C.S. PO 02, PO 152, PO 83, PO 189, PO 38, PO 190

Chin Jin S. PO 191

Chinswangwatanakul V. PO 81

Chong C.Y.L. PO 02

Clément E. PO 110

Colletti G. PO 56, PO 94, PO 67

Colombo P.E. PO 213, PO 214

Combari P. PO 61

Contis J. PO 88

Cooper L. PO 78

Cosgrove P. PO 18

Costa A. PO 173

Cosyns S. PO 97

Creemers G.J. PO 141

Cristoudo A. PO 127

Crocheray C. PO 13

Cruz C. PO 46, PO 47

Culerier E. PO 178

D

D’hondt V. PO 214

Dadgar N. PO 76, PO 77

Dagenborg V.J. PO 106

D’agostino L. PO 164

Dalle D. PO 205

Dani D. PO 155

Daniel D. PO 155

Dankers P.Y.W. PO 19

D’annibale G. PO 165

Das P. PO 200

Davies L. PO 113

Dayal S. PO 60, PO 115, PO 126

De Coninck J. PO 104

De Hingh I. PO 153, PO 154, PO 72, PO 141

De Hingh I.H.J.T. PO 19, PO 91

De Hingh I.H.J. PO 93, PO 119, PO 121

De Leon L.C. PO 54

De Manzoni G. PO 87, PO 66

De Meuleneir K. PO 97

De Simone M. PO 186

Debarbieux S. PO 205

Debbaut C. PO 36

Delgadillo X. PO 21, PO 34, PO 37

Delia D. PO 51

Dellinger T. PO 18, PO 181

Demuytere J. PO 104

Deraco M. PO 48, PO 56, PO 94, PO 95, PO 67

Desouza A. PO 109, PO 111

Detlefsen S. PO 12, PO 53, PO 158, PO 159, PO 160, PO 43, PO 132

Di Giorgio A. PO 164, PO 165

Dietz M. PO 27, PO 55

Dinh T. PO 181

Dmitriev R.I. PO 86

Doi A. PO 133

Dolnikov S. PO 78

Donnenberg A. PO 76, PO 77

Donnenberg V. PO 76, PO 77

Dørum A. PO 212

Doskovic M. PO 123

Dumas C. PO 01

Dumont F. PO 01, PO 145, PO 49

Durand Fontanier S. PO 06, PO 07, PO 16

Duzgun O.D. PO 182

Duzgun O. PO 135

E

Eckhoff L. PO 158, PO 159, PO 160

El Ahmadi B. PO 187, PO 204

El Fassi A. PO 204

Elferink M. PO 154

Emmanuel K. PO 31

Enblad M. PO 147, PO 73

Ennaceur F. PO 57

Ernst S. PO 104

Eveno C. PO 161

Ezanno A.C. PO 202, PO 118, PO 120

F

Fabbro M. PO 214

Faget J. PO 178

Fagihi M. PO 174, PO 175, PO 176, PO 177, PO 71

Fallabrino G. PO 94

Falla-Zuniga L.F. PO 39

Fauvre A. PO 178

Fawaz J. PO 120

Feriani N. PO 57

Fernandes M. PO 26

Fernandes A.M. PO 198

Fernandez H. PO 129

Fernandez De Sevilla E. PO 203, PO 61

Fernandez Vazquez M.L. PO 143

Fernández Vázquez M.L. PO 69

Ferracci F. PO 164, PO 165

Ferrari M. PO 03

Ferreira Pinto P. PO 215

Fijneman R.J.A. PO 107

Filippini F. PO 87

Flatmark K. PO 106, PO 212

Flood M. PO 68

Fong W.J. PO 84, PO 40, PO 190, PO 191, PO 192, PO 206

Fong Y. PO 139

Fourcaud L. PO 52

Fragulidis G. PO 88

Frankel P. PO 18, PO 181

Fransen P.P.K.H. PO 19

Frías-Fernández P. PO 65

Fristrup C. PO 12, PO 50

Fristrup C.W. PO 160, PO 43

Fu Y. PO 70

Fugazzola P. PO 95, PO 112

Futo Y. PO 157

G

Galanos L. PO 153, PO 154, PO 72

Galindo Alins M.J. PO 143

Ganesan J. PO 111

Garambois V. PO 178

García C. PO 170

García-García J.F. PO 184

Gazeau F. PO 13

Gelli M. PO 203, PO 61

Gelmini R. PO 186

Gervais M.K. PO 140

Ghaly A. PO 112

GhaniPO ur L. PO 147, PO 73, PO 116

Ghannam A. PO 204

Gil-Gómez E. PO 41, PO 42

Glehen O. PO 80, PO 186, PO 49, PO 205, PO 110, PO 118 , PO 130

Glehen O. PO 130

Goel D. PO 209

Goere D. PO 49

Goéré D. PO 124

Goh N. PO 149

Gohda Y. PO 98

Gomes I. PO 197

Gomez K. PO 82, PO 171, PO 172, PO 45, PO 210

Gómez-Dueñas G. PO 41, PO 42

Gonçalves F. PO 173

Gongora C. PO 178

Gonzalez Bayon L. PO 143

González Bayón L.A. PO 69

González Moreno S. PO 33

González-Gil A. PO 41, PO 42

González-Moreno S. PO 184

Goodman M.D. PO 185, PO 62

Goscinski M.A. PO 212

Goyal D. PO 216

Graf W. PO 147, PO 73

Granados-Rodríguez M. PO 17

Graversen M. PO 12, PO 53, PO 158, PO 159, PO 160, PO 43, PO 132

Gray B.D. PO 83

Greco A. PO 03

Grillo Marín C. PO 33

Grillo-Marin C. PO 184

Grotenhuis B.A. PO 107

Guaglio M. PO 56, PO 94, PO 67

Guerrero-Macías S. PO 46, PO 47, PO 137

Guillonneau F. PO 04

Guimarães A. PO 26

Gül-Klein S. PO 128, PO 64

Guo W.Y. PO 02

Gushchin V. PO 39

H

Haase E. PO 140

Haerinck J. PO 104

Hald Nielsen S. PO 50

Hamm J. PO 72, PO 103

Hansen P.S. PO 159, PO 160

Hansen P.S.H. PO 158

Harou O. PO 205

Hedfi M. PO 57

Hellemond I. PO 72

Henry C. PO 04

Heriot A. PO 68

Hernandez A. PO 82, PO 171, PO 172, PO 45, PO 210

Hernandez Kakauridze S. PO 143

Hideyo H. PO 134

Hideyuki H. PO 134

Hildebrandt M.G. PO 53

Hironori H. PO 24, PO 134

Hironori Y. PO 157

Hiroyuki I. PO 24

Hoff L. PO 64

Horwood J. PO 113

Hosoya Y. PO 157

Huang R. PO 20

Hübner M. PO 75

Hwang S. PO 15

Hytham A. PO 21

I

Iglesias M. PO 179

IliakoPO ulos K. PO 88

Imboden S. PO 208

Inaba T. PO 136

Isabelle B. PO 80

Ishigami H. PO 136

Iugai S. PO 39

J

Jäger T. PO 31

Jain A. PO 68

Jansson Palmer G. PO 102, PO 116

Javed T. PO 191

Jazon G.L. PO 25

Jeanson L. PO 178

Jelin-Klaric M. PO 158, PO 159, PO 160

Jensen K.M. PO 158, PO 159, PO 160

Jeon W.J. PO 155

Jimenez P. PO 137

Jiménez Monasterio M. PO 69

Jmal E. PO 57

Joji J. PO 134

Jørgensen M.S. PO 32

Joshi P. PO 82, PO 171, PO 45, PO 210

Joung B. PO 155

Juan W.K.D. PO 152

Jung Y. PO 180, PO 15, PO 180

K

K M. PO 111

Kaiser L. PO 31

Kanamaru R. PO 157, PO 134

Kaneko Y. PO 157

Kanemitsu Y. PO 92

Kang S.H. PO 138

Kaprin A. PO 144

Kashtan H. PO 78

Kato T. PO 92

Kazi M. PO 109, PO 111

Kazuya K. PO 134

Kefleyesus A. PO 130

Kemmel V. PO 161

Kentaro K. PO 134

Kepenekian V. PO 80, PO 110, PO 114, PO 130

Kepenekian V. PO 130

Kerkhoff T.M.E. PO 91

Kesseler V. PO 75

Khochbin S. PO 99

Khomiakov V. PO 144

Kim S.I. PO 139

Kim H.H. PO 180, PO 15, PO 180, PO 138

Kim H.S. PO 08, PO 09, PO 10

Kim S.S. PO 138

Kim J.H. PO 168

Kim U. PO 168

Kim J. PO 168, PO 138

Kim D.Y. PO 180, PO 15, PO 180

Kim G. PO 74

King M.C. PO 39

Kishikawa J. PO 136

Kitayama J. PO 157

Kiyomiddinovna M.S. PO 08, PO 09, PO 10

Koh H. PO 168

Koh P.L. PO 152, PO 38, PO 40, PO 190, PO 191

Kok N. PO 154, PO 72

Kok N.F.M. PO 91, PO 107, PO 108

Kok S.Y. PO 60

Kolobaev I. PO 144

Konishi T. PO 136

Kordeni K. PO 88

Koukoutsidi P.K. PO 185, PO 62

Kovalik V. PO 39

Kubias J. PO 208

Kubota K. PO 136

Kulkarni S. PO 63

Kumar A. PO 44

Kunde A. PO 128

Kurashina K. PO 157

Kusamura S. PO 56, PO 94, PO 67

Kuznetsova O. PO 144

Kwon N.J. PO 138

L

Lacueva F.J. PO 41, PO 42, PO 195

Lahaye M.J. PO 107

Lahnaoui O. PO 187, PO 204

Landeras López Á. PO 69

Lario-Pérez S. PO 195

Larsen S.G. PO 106, PO 212

Latincic S. PO 123

Laurent-Puig P. PO 148

Lavelle A. PO 68

Laverriere M.H. PO 99

Lay L. PO 37

Le Bourhis L. PO 124

Le Gall M. PO 04

Leblanc G. PO 140

Lee S. PO 08, PO 09, PO 10, PO 180, PO 15, PO 180

Lee S.H. PO 08, PO 09, PO 10

Lee D.K. PO 180, PO 15, PO 180

Lee J. PO 138

Leiritz E. PO 110

Lenaerts K. PO 19

Lepsenyi M. PO 116

Lewis C. PO 76, PO 77

Li Y. PO 58, PO 70

Liang X.L. PO 58

Liberale G. PO 97

Lillo-García C. PO 195

Lim M.C. PO 168

Lim M.G. PO 25

Linares J. PO 179

Lindegaard C.A.L. PO 158

Lino-Silva L. PO 105, PO 65

LipPO lis P.V. PO 03

Liu Y. PO 02

Lo Dico R. PO 89, PO 48

Lodoli C. PO 164, PO 165

Long Y. PO 36

Lopes J. PO 173

Lopez M.P. PO 25

Lopez De Felipe Gumiel M. PO 143

López De Felipe Gumiel M. PO 69

López-Rodríguez F. PO 195

López-Rojo I. PO 184

Lozano Lominchar P. PO 143

Lu J. PO 139

Luis C. PO 170

Lund-Andersen C. PO 106

Lund-Iversen M. PO 106

Lurvink R.J. PO 93, PO 119

Luyer M. PO 153, PO 141

M

Macia J.J. PO 195

Mack L. PO 140

Madeira-Cardoso M.J. PO 26, PO 215

Madsen E. PO 72

Maheshwari A. PO 111

Majbar M.A. PO 187, PO 204

Makni A. PO 188

Malgras B. PO 202, PO 118, PO 120

Manrique-Acevedo M.E. PO 46, PO 47, PO 137

Marchal F. PO 118

Marcil S. PO 140

Mariani A. PO 148

Marques M. PO 26, PO 215

Marrelli D. PO 95

Martelli V. PO 179

Martin M. PO 51

Martin Roman L. PO 143

Martínez C.R. PO 69

Martin-Romano L. PO 48

Massaras D. PO 88

Mathijssen R. PO 72

Mathis J. PO 208

Meireles S. PO 173

Mekkawy A.H. PO 27, PO 28

Meloni F. PO 87, PO 66

Mendes J. PO 26

Mercier F. PO 140

Methasate A. PO 81

Metheniti P. PO 88

Miao D. PO 20

Michán C. PO 17

Mignet N. PO 161

Milazzo L. PO 178

Millione M. PO 56

Mingzhe C. PO 191

Misad O. PO 34

Mkhinini W. PO 57

Mohamed F. PO 60, PO 115, PO 126

Mohammad M. PO 51

Mohan H. PO 68

Mohsine R. PO 187, PO 204

Moller M. PO 82, PO 171, PO 172, PO 45, PO 210

Montagut C. PO 179

Montealegre M. PO 82, PO 171, PO 172, PO 45, PO 210

Mor E. PO 142

Mor A. PO 111

Morales García C. PO 69

Moran B. PO 60, PO 115, PO 126

Morel Y. PO 110

Moreno-Serrano A. PO 17

Morezzi D. PO 112

Moritani K. PO 92

Morris D.L. PO 27, PO 28

Morris D. PO 55, PO 127

Mortensen M.B. PO 53, PO 158, PO 159, PO 160, PO 43, PO 132

Mortensen M. PO 12

Mouawad C. PO 161

Mourregot A. PO 214

Moya-Martínez A. PO 195

Mueller M. PO 208

Musco B. PO 03

Musters G. PO 103

N

Nagata H. PO 92

NamPO olsuksan C. PO 81

Naohiro N. PO 134

Narayanachary R. PO 129

Nath A. PO 18

Nava F.L. PO 56, PO 95

Nava F. PO 67

Navin K. PO 67

Neuberg M. PO 203

Neureiter D. PO 31

Ng A.T.I. PO 40

Ng G. PO 02

Nienhuijs S. PO 91

Nieroda C. PO 39

Nilsson M. PO 132

Nilsson P. PO 116

Nissan A. PO 142

Noe D. PO 82

Nogueiro J. PO 173

Noventa C. PO 95

Núñez-O’sullivan S. PO 184

O

Office M. PO 100

Oh S.H. PO 08, PO 09, PO 10

Ohzawa H. PO 157

Okkelman I.A. PO 86

Ølholm A.M. PO 50

Olivier G. PO 114

Omstead A. PO 76

Ong C.A.J. PO 02, PO 152, PO 83, PO 38, PO 40, PO 192

Ong J.C.A. PO 189, PO 190, PO 206

Ong W.S. PO 84

Ong X.Y.S. PO 83

Ong J. PO 84, PO 191

Orgad R. PO 78, PO 80

Orsini C. PO 165

Ortega Pérez G. PO 33

Ortega-Pérez G. PO 184

Osman O.S.M.A.N. PO 57

Özcan P.O. PO 182, PO 135

P

Pacelli F. PO 164, PO 165

Paik B. PO 84, PO 38

Pak K.Y. PO 83

Palencia Garcia N. PO 143

Palencia García N. PO 69

Pang A. PO 74

PaPO vic M. PO 123

Parakonthun T. PO 81

Park S.Y. PO 168

Park J.W. PO 08, PO 09, PO 10

Park A. PO 139

Park J. PO 180, PO 180

Park Y.S. PO 138

Park H.Y. PO 76, PO 77

Parker J. PO 113

Pascual M. PO 179

Patel M. PO 68

Pathak B. PO 59, PO 125

Patil D. PO 198

Patterson R. PO 162

Pavlov M. PO 123

Pawar A. PO 114

Pearson J. PO 210

Peleg D. PO 78

Perrin M.L. PO 06, PO 07, PO 16

Perrina D. PO 112

Perrone F. PO 67

Perrota F. PO 37

Peyroteo M. PO 26, PO 215

Pezzopane R. PO 17

Pfeiffer P. PO 12, PO 53, PO 132

Pfeiffer P.V. PO 43

Phalanusitthepha C. PO 81

Piccini L. PO 03

Pierre M. PO 46

Pietrantonio F. PO 67

Pillai K. PO 27, PO 28

Pimpie C. PO 161

Pinson J. PO 110

Pinto P. PO 26

Pires M. PO 215

Pocard M. PO 13, PO 161, PO 49, PO 202, PO 110, PO 118, PO 120

Politis D. PO 88

Portela S. PO 68

Poudevigne O. PO 202

Pouliquen D.L. PO 04

Poutrel G. PO 213

Presl J. PO 31

Prieto López L. PO 33

Proto P. PO 94

Puccio C. PO 87, PO 66

Q

Qentasi I.B. PO 34

Qin X. PO 20

Quenet F. PO 213

Quénet F. PO 214

Quesada J.L. PO 202, PO 118, PO 120

R

Rahardjo A.B. PO 01

Rahimi-Gorji M. PO 36

Rahman M.K. PO 27, PO 28

Ramia J. PO 41, PO 42

RamsPO tt J.P. PO 31

Raoof M. PO 18, PO 181

Rashed M. PO 73

Rau B. PO 128, PO 64

Rauwerdink P. PO 93

Ravi V. PO 151

Ray M.D. PO 11, PO 150, PO 151, PO 44, PO 209, PO 216

Ray M. PO 59, PO 125

Reis T. PO 197

Reis M. PO 197, PO 197

Remaut K. PO 19

Remner M. PO 60

Renape N. PO 49

Reymond M. PO 156

Reymond M.A. PO 22

Reynaud B. PO 205

Ribeiro C. PO 215

Ribereau-Gayon E. PO 205

Rigoli R. PO 87, PO 66

Rijken A. PO 153, PO 154

Robella M. PO 48, PO 201

Rodemund N. PO 31

Rodolfino E. PO 164

Roensholdt S. PO 53

Rohit Kumar C. PO 198

Rokkones E. PO 212

Romero-Ruiz A. PO 17

Rouanet P. PO 214

Rousseaux S. PO 99

Rouvelas I. PO 132

Rovers K.P. PO 91

Rovers K.P.B. PO 119

Roviello F. PO 186

Rozenbaum P. PO 13, PO 161

Rreka E. PO 03

Ruel N. PO 18, PO 181

Ruggeri G. PO 208

Ryabov A. PO 144

S

Sabia D. PO 129

Saeed N. PO 86

Safi C. PO 111

Sage P.Y. PO 202

Sage P. PO 120

Saihb A. PO 174, PO 175, PO 176, PO 177, PO 71

Saito S. PO 157

Saklani A. PO 109, PO 111

Salazar E. PO 206

Salcedo-Hernández R. PO 105

Salcedo-Hernánez R. PO 65

Saleh M. PO 78

Salomon S. PO 43

Salvans S. PO 179

Sammartino P. PO 48

Samuel M.V. PO 115

Samy M. PO 196, PO 199

Sanberg J. PO 132

Sánchez-Guillén L. PO 195

Sandoval J. PO 34, PO 37

Saner F. PO 208

Santana Castaño N. PO 143

Santullo F. PO 164, PO 165

Sardi A. PO 39

Sarmiento R. PO 54

Sautkin I. PO 22, PO 156

Schnelldorfer T.S. PO 185

Schoenfelder H. PO 156

Schredl P. PO 31

Schütz E. PO 208

Sedano C.A. PO 34

Seitenfuss R. PO 37

SemPO ux C. PO 75

Seo C.J. PO 152, PO 83, PO 84, PO 38, PO 40, PO 190, PO 192, PO 206

Seuter R. PO 153

Seye W. PO 145

Sgarbura O. PO 49, PO 178, PO 213, PO 214

Sgrabura O. PO 51

Shabbir A. PO 74

Shanbagh E. PO 198

Sharma A. PO 11, PO 109, PO 111

Sharma R. PO 162

Shim S.H. PO 08, PO 09, PO 10

Shimonovitz Moore M. PO 56

Shin J. PO 63

Shin S. PO 134

Shuji K. PO 133

Sidéris L. PO 140

Siegenthaler F. PO 208

Siew C.C.H. PO 149

Simkens G. PO 141

Singh G. PO 150

Sinno A. PO 210

Smolenschi C. PO 61

Snaebjornsson P. PO 107

So J. PO 186

So J.B.Y. PO 74

Soh N.H. PO 190

Solass W. PO 75, PO 208

Solomi.v C. PO 209

Somashekhar S.P. PO 198

Sommariva A. PO 201, PO 95

Sonvane S. PO 100

Souadka A. PO 187, PO 188, PO 188, PO 204

Sourrouille I. PO 203, PO 61, PO 110, PO 118

Sousa F. PO 26

Spasojevic M. PO 212

Spiliotis J. PO 23

Spinelli A. PO 99

Stefano M. PO 112

Subramani P. PO 200

Sugarbaker P.H. PO 184

Sugarbaker P. PO 183

Suh Y.S. PO 138

Sundar R. PO 74

Suwatthanarak T. PO 81

Swangsri J. PO 81

Syk I.S. PO 79

Syk I. PO 102

Syk I.K. PO 116

T

Taibi A. PO 06, PO 07, PO 16, PO 49

Takahara N. PO 24

Takahashi K. PO 157

Takamizawa Y. PO 92

Takashi S. PO 24

Takashima A. PO 92

Taminau J. PO 104

Tan Q.X. PO 02, PO 83

Tan H.L. PO 74

Tan M.V. PO 206

Tan J.K.T. PO 192

Tan M. PO 191

Tan J.W.S. PO 02, PO 83, PO 189

Tanis P.J. PO 91

Tanis P. PO 72, PO 103

Tarpgaard L. PO 12, PO 43, PO 132

Tavares N. PO 173

Tawantanakorn T. PO 81

Tedone F. PO 87

Teijo-Quintans A. PO 184

Teixeira Farinha H. PO 75

Tejedor A. PO 129

Teramura K. PO 133

Thaury M. PO 13

Theillac C. PO 205

Thezenas S. PO 213, PO 214

Thorlacius H. PO 116

Thouvenin A. PO 161

Tibaudeau E. PO 01

Tidadini F. PO 202, PO 118, PO 120

Tidani F. PO 110

Tomás Rodríguez M.I. PO 195

Tomassi M.O.N.I.C.A. PO 130

Tonello M. PO 201, PO 95

Torgunrud A. PO 106

Torkington J. PO 113

Torres M. PO 197

Torres-Martínez M. PO 17

Tortora G. PO 164

Trakarnsanga A. PO 81

Tranberg J. PO 102

Trilling B. PO 202, PO 120

Trinidad I. PO 101

Tschann P. PO 31

Tsekrekos A. PO 132

Tselikas L. PO 203

Tsuji Y. PO 133

Tsukamoto S. PO 92

Tuech J.J. PO 112

Tur Martinez J. PO 129

Tuynman J. PO 72

Tuynman J.B. PO 108, PO 121

Tzivanakis A. PO 60, PO 115, PO 126

U

Uribe M. PO 37, PO 170

Ursino C. PO 178

Utkina A. PO 144

V

Vadisetti S.N. PO 109

Vaira M. PO 186, PO 48, PO 201

Valdimarsson V.V. PO 79

Valdimarsson V. PO 116

Vale Guimarães A. PO 215

Valencia H. PO 139

Valenzuela B. PO 213

Valenzuela-Mendez B. PO 214

Valle M. PO 89, PO 48, PO 95

Valle S.J. PO 27, PO 28

Valle S. PO 55

Vallicelli C. PO 112

Valmary-Degano S. PO 99

Van Almen G.C. PO 19

Van De Vlasakker V.C.J. PO 93

Van Den Berg R. PO 103

Van Den Heuvel T.B.M. PO 119, PO 121

Van Der Snee L. PO 107

Van Der Speeten K. PO 97

Van Eden W.J. PO 107

Van Erning F.N. PO 91

Van Erning F. PO 153, PO 154, PO 141

Van Hellemond I. PO 141

Van Hellemond I.E.G. PO 91, PO 119

Van Kesteren L.J. PO 108, PO 121

Van Laarhoven H. PO 141

Vansteenkiste F. PO 97

Varejão A. PO 215

Vargas M. PO 137

Vasiljevic J. PO 123

Vázquez-Borrego M.C. PO 17

Verhoef C. PO 72, PO 91

Verhoeven R. PO 153, PO 141

Verrièle V. PO 04

Verwaal V.V. PO 79

Vezzio-Vié N. PO 178

Viamonte B. PO 173

Vidal J. PO 179

Vieira De Sousa J.P. PO 173

Vignaud T. PO 01

Vigutto G. PO 112

Vilcot L. PO 145

Villella J. PO 181

Villeneuve L. PO 130

Villeneuve L. PO 80, PO 130

Vispute T. PO 109

Vitte A.L. PO 99

Viveros-Carreño D. PO 46, PO 47

Volatron J. PO 13

W

Wagner P. PO 76, PO 77

Walsh R.J. PO 74

Wang H. PO 20

Wang E. PO 181

Wang Y.L. PO 149

Warrier S. PO 68

Wassenaar E. PO 93

Wasserberg N. PO 78

Wegel S. PO 128

Weinreich J. PO 22

Weiß B. PO 64

Weitzendorfer M. PO 31

Welten M. PO 141

Wernberg J.A. PO 162

Westbrook S. PO 60

Willaert W. PO 36, PO 97

Williams B. PO 63

Wintjens A.G.W.E. PO 19

Wong S.M.J. PO 38, PO 40, PO 192

Wong L.C.K. PO 152

Wong J. PO 84, PO 191

Wong J.S.M. PO 02, PO 152, PO 83, PO 190, PO 206

Woo E. PO 168

Woo Y. PO 168, PO 139

Wuthrich P. PO 21

X

Xiao K.K. PO 76, PO 77

Y

Yadegarina S. PO 171, PO 172

Yadegarynia S. PO 82, PO 45, PO 210

Yang R. PO 70

Yang A. PO 139

Yanghee Y. PO 155

Yano H. PO 98

Yap C. PO 74

Yaqub S. PO 106

Yardin C. PO 06, PO 07, PO 16

Yim G.W. PO 09, PO 10

Yim T.H.J. PO 189

Yonemura Y.Y.Y.Y. PO 05

Yong W.P. PO 74

Yoo M. PO 180, PO 15, PO 180

Yoon C. PO 180, PO 15, PO 180

Yoshinori Y. PO 134

Yost S. PO 18, PO 181

Youcef A. PO 110

Yousuke Y. PO 24

Z

Zacchi I. PO 87, PO 66

Zaidi A. PO 76, PO 77

Zambrano D. PO 213

Zamora M.A. PO 25

Zeweta A.S. PO 196, PO 199

Zhang Z. PO 139

Zhong X. PO 190

Zippel D. PO 142

Zwanenburg E. PO 103

